# Discovery of the 3-Amino-1,2,4-triazine-Based Library as Selective PDK1 Inhibitors with Therapeutic Potential in Highly Aggressive Pancreatic Ductal Adenocarcinoma

**DOI:** 10.3390/ijms24043679

**Published:** 2023-02-12

**Authors:** Daniela Carbone, Michele De Franco, Camilla Pecoraro, Davide Bassani, Matteo Pavan, Stella Cascioferro, Barbara Parrino, Girolamo Cirrincione, Stefano Dall’Acqua, Stefano Moro, Valentina Gandin, Patrizia Diana

**Affiliations:** 1 Department of Biological, Chemical, and Pharmaceutical Sciences and Technologies (STEBICEF), University of Palermo, Via Archirafi 32, 90123 Palermo, Italy; 2 Department of Pharmaceutical and Pharmacological Sciences, University of Padova, Via F. Marzolo 5, 35131 Padova, Italy; 3 Molecular Modeling Section (MMS), Department of Pharmaceutical and Pharmacological Sciences, University of Padova, Via F. Marzolo 5, 35131 Padova, Italy

**Keywords:** 3-amino-1,2,4-triazine derivatives, bis-indole derivatives, PDK inhibitors, Kras-mutated pancreatic ductal adenocarcinoma, antitumor agents, ligand-based homology modeling

## Abstract

Pyruvate dehydrogenase kinases (PDKs) are serine/threonine kinases, that are directly involved in altered cancer cell metabolism, resulting in cancer aggressiveness and resistance. Dichloroacetic acid (DCA) is the first PDK inhibitor that has entered phase II clinical; however, several side effects associated with weak anticancer activity and excessive drug dose (100 mg/kg) have led to its limitation in clinical application. Building upon a molecular hybridization approach, a small library of 3-amino-1,2,4-triazine derivatives has been designed, synthesized, and characterized for their PDK inhibitory activity using in silico, in vitro, and in vivo assays. Biochemical screenings showed that all synthesized compounds are potent and subtype-selective inhibitors of PDK. Accordingly, molecular modeling studies revealed that a lot of ligands can be properly placed inside the ATP-binding site of PDK1. Interestingly, 2D and 3D cell studies revealed their ability to induce cancer cell death at low micromolar doses, being extremely effective against human pancreatic KRAS mutated cancer cells. Cellular mechanistic studies confirm their ability to hamper the PDK/PDH axis, thus leading to metabolic/redox cellular impairment, and to ultimately trigger apoptotic cancer cell death. Remarkably, preliminary in vivo studies performed on a highly aggressive and metastatic Kras-mutant solid tumor model confirm the ability of the most representative compound **5i** to target the PDH/PDK axis in vivo and highlighted its equal efficacy and better tolerability profile with respect to those elicited by the reference FDA approved drugs, cisplatin and gemcitabine. Collectively, the data highlights the promising anticancer potential of these novel PDK-targeting derivatives toward obtaining clinical candidates for combatting highly aggressive KRAS-mutant pancreatic ductal adenocarcinomas.

## 1. Introduction

Cancer cells change their flow across different metabolic pathways to fulfill the increased bioenergy and biosynthetic demand needed for cell proliferation and survival [1]. 

One of the molecular mechanisms leading to the metabolic rewiring of cancer cells, which has been given attention as a candidate for cancer treatment, is the aerobic glycolysis pathway, often referred to as the “Warburg effect” [2]. This effect is a hallmark of cancer that refers to the preference of cancer cells to utilize aerobic glycolysis to produce energy, through a shift of glucose metabolism toward lactate production rather than glucose catabolism via the tricarboxylic acid (TCA) cycle, even with ample oxygen supply. The role of aerobic glycolysis in cancer growth and survival has been investigated in different cancer types, including pancreatic ductal adenocarcinoma (PDAC) [3].

The PDAC microenvironment is characterized by the presence of dense fibroblast stromal cells and large areas of hypoxic regions, which may be due to poor perfusion of blood and dense stroma. In response to the reduced oxygen uptake, the tumor cells undergo metabolic reprogramming to promote the Warburg effect metabolism, which involves increased levels of glycolysis. Moreover, PDAC cells in hypoxic areas tend to undergo epithelial-to-mesenchymal transformation (EMT), acquiring a high tendency to metastasize [4]. With hypoxia, in PDAC, the expression of HIF-1a is increased, as is the expression of glucose metabolic enzymes, such as pyruvate dehydrogenase kinases (PDKs). 

In PDAC, PDKs are the major regulatory enzymes of glucose metabolism as they hold a negative role in the regulation of the pyruvate dehydrogenase complex (PDC) by phosphorylating its specific serine residues and subsequently deactivating the system if present in excess. Among PDK isoforms, PDKs 1–3 interact with various signaling factors enhancing cancer progression and functioning as oncogenes, whereas PDK4 only acts as an oncogene [4].

In PDAC, the knockdown of HIF-1a under hypoxic conditions inhibited lactate production and the expression of PDK1, suppressing the growth of the pancreatic cells BxPC-3 along with the induction of apoptosis. Indeed, in the PDAC cells with constitutive HIF-1a expression, PDK1 was also more widely expressed, thereby facilitating increased anaerobic metabolism and apoptosis resistance under conditions of hypoxia and glucose deprivation. This is probably due to the HIF1-mediated upregulation of PDK1 and inactivation of the pyruvate dehydrogenase complex, with subsequent loss of pyruvate oxidation, and induces cancer senescence through increased oxygen consumption and mitochondrial oxidative stress [5,6]. 

In addition, PDAC is primarily characterized by the Κirsten rat sarcoma viral oncogene homolog (KRAS) mutation, with substantial experimental evidence that mutant KRAS is an essential requirement for PDAC growth and progression [7]. Accordingly, the NCI recently identified targeting KRAS as one of four major priorities for pancreatic cancer research in the next 10 years. The main past and current strategies for developing therapeutics to block mutant KRAS function have focused on indirect approaches, which encompass selective targeting of proteins that support KRAS function and promote KRAS-driven cancer growth. A recent pioneering study by Trinidad et al. made a clear connection between PDK4 inhibition and induction of cell death in KRAS mutant cancer cells [8]. They reported that PDK4 knockdown by specific small interfering (si)RNAs downregulates mutant (KRAS) expression and consequently strongly suppresses the growth of cancer cells. Similarly, recent finding clearly highlights that PDAC cells dependent on KRAS-driven metabolism are more sensitive to cell death induction via inhibition of enzyme regulation involved in glucose metabolism, such as PDKs. Altogether, this finding highlights that PDK4 and, possibly, all the PDK isoforms represent promising targets for cancer therapy of highly aggressive KRAS mutant cancers. This marks the attractive possibility of selectively targeting PDAC by the inhibition of one or more obligate glucose-metabolizing pathways, highlighting the key role of PDKs in PDAC and suggesting that targeting PDKs would present an exciting therapeutic possibility for KRAS mutant PDAC [9].

**Figure 1:**
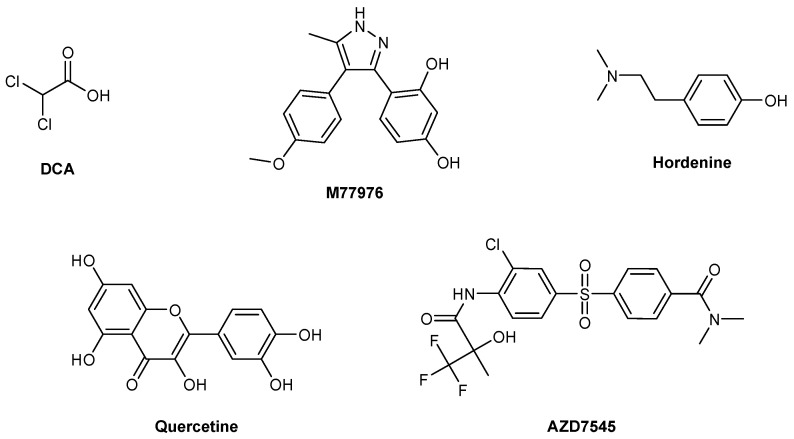
Structures of some representative PDK inhibitors.

Presently, some potent small molecules targeting PDKs are important milestones in the historical development of cancer research; for example, dichloroacetic acid (DCA) and its derivatives, the dihydroxyphenyl pyrazole M77976, hordenine, quercetin, and the sulfone AZD7545 (Figure 1) showed potent antiproliferative activity in different cancer cell lines and mouse models. Unfortunately, none of them has successfully overcome clinical trials due to off-target effects, low potency, or poor selectivity [10,11,12].

DCA is the first PDK inhibitor that has entered phase II clinical trials (NCT05120184), but its side effects, as well as weak anticancer activity and excessive drug dose (100 mg/kg), limited its clinical application [13]. 

Since PDK inhibitors known to date, do not possess structural similarities to each other [14], we thought to use a molecular hybridization approach for developing new compounds with improved efficacy and safety, by combining two or more pharmacophores of bioactive scaffolds in a single structure.

A plethora of heterocyclic moieties-based anticancer derivatives has been explored recently keeping in mind the brighter potential of heterocyclic compounds, which can occupy the nucleotide-binding pocket, forming hydrogen bonds with the residues of the kinase hinge region. Considering that the 1,2,4-triazine nucleus and indole ring have been identified as privileged scaffolds for the development of molecules with a wide range of pharmacological activities, especially antitumor, through different mechanisms of action involving inhibition of protein kinases [15,16,17,18,19,20,21,22,23,24,25,26,27,28,29]. Herein we report the synthesis of new hybrid scaffolds combining indole and 1,2,4-triazine moieties, in order to obtain new PDK inhibitors that are able to meet the demand for effective PDAC therapeutics. 

## 2. Results and Discussion

### 2.1. Synthesis

**Scheme 1:**
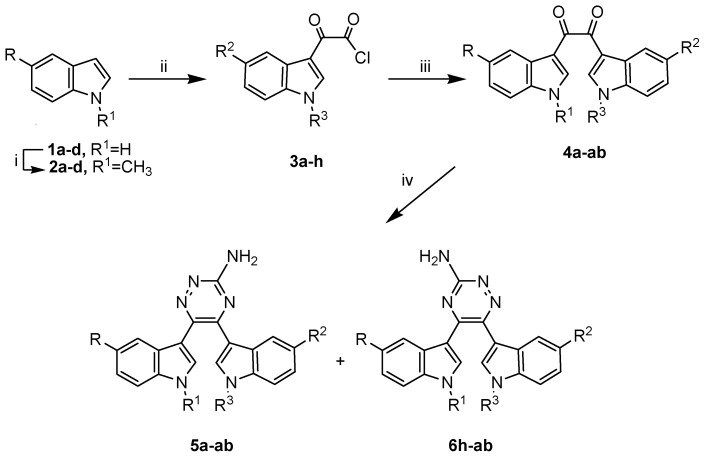
Synthesis of the 5,6-di(1*H*-indol-3-yl)-1,2,4-triazin-3-amines **5** and **6**. Reagents and conditions: (i) *t*-BuOK, TDA-1, toluene, RT, 1–24 h, then CH_3_I, RT, 1–2 h, 97–99%; (ii) oxalyl chloride, diethyl ether, 0 °C, 3 h, then RT, 1 h, 86–96%; (iii) **1a–d** or **2a–d**, AlCl_3_, DCM/heptane 2:1, RT, 2 h, 60–85%; (iv) aminoguanidine bicarbonate, *n*-butanol, reflux, 3 h, **5a–g:** 55–98%, **5h–ab**: 33–65%, **6h–ab**: 29–57%.

The synthesis of 3-amino-1,2,4-triazine derivatives **5a–ab**, **6h–ab** follows a common synthetic procedure shown in Scheme 1. 

The Friedel–Crafts acylation of indoles **1a–d** or **2a–d** with indolyl-oxo-acetyl chlorides **3a-h**, using AlCl_3_ as a strong Lewis acid catalyst, proceeded via electrophilic aromatic substitution to form the 1,2-diones **4a–ab**. (60–85%), useful key intermediates to obtain derivatives **5** and **6**. The 1-methyl-1*H*-indoles **2ad** and indolyl-oxo-acetyl chlorides **3a,c–h** were prepared from their corresponding indoles as previously reported [30,31,32,33]. The new indolyl-oxo-acetyl chloride **3b** was prepared from the commercially available 1*H*-indole **1b** which was subjected to acylation using an excess of oxalyl chloride in diethyl ether to afford the desired chloride in excellent yield (96%).

The ring closure reaction of bis(1*H*-indol-3-yl)ethane-1,2-diones **4a–ab** with aminoguanidine bicarbonate in refluxing *n*-butanol, led to the desired heterocyclic nitrogen system, giving a mixture of the two isomeric triazines **5** and **6**. All the synthesized derivatives and their relative yields are summarized in Table S1 in Supplementary Materials. The experimental data, the NMR spectra and the MS chromatograms are likewise reported in the Supplementary materials (Figures S1–S98).

### 2.2. Enzyme Inhibition Activity

**Figure 2:**
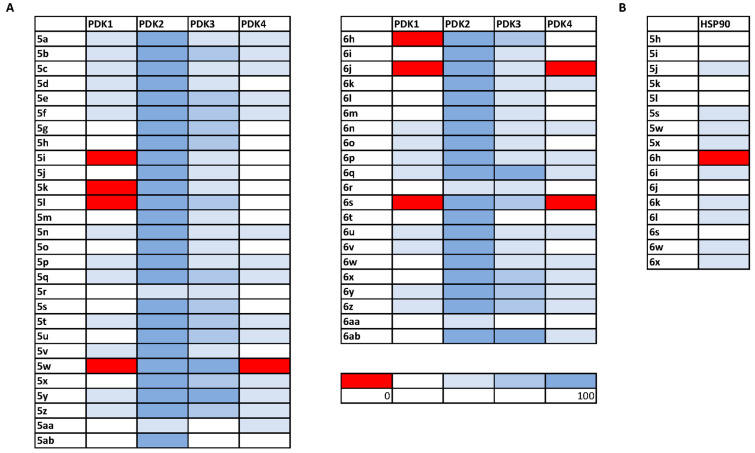
Inhibition of isolated enzyme isoforms. Heat-map showing the relative inhibition of tested compounds (at 1.5 µM) on PDK 1–4 (**A**) and HSP90 (**B**) enzymes. Results are expressed as enzyme residual activity. Data are the means of three independent experiments.

The ability of tested compounds to inhibit the activity of PDK 1–4 isoforms was primarily assessed in cell-free experiments by a chemioluminescent assay, the ADPGlo™ Kinase Assay kit from Promega (Promega Corporation, Madison, WI, USA). The assay was performed by using all four PDK 1–4 isoforms incubated for 30 min at room temperature with 1.5 µM of tested compounds, and results are reported in Figure 2A as a heat-map summary of the inhibition percentage induced by tested compounds on PDK 1–4 catalytic activity. All novel compounds, although to a different extent, were able to inhibit the pyruvate dehydrogenase kinases, with selectivity against the PDK1 and PDK4 isoforms. Among the new derivatives, compounds **5i**, **5k**, **5l**, **5w**, **6h**, **6j** and **6s** totally suppressed the enzymatic activity of PDK1 at 1.5 µM. Derivatives, **5w**, **6j** and **6s** also completely blocked the consumption of ATP by inhibiting PDK4 activity. In contrast, all compounds were barely effective in inhibiting the activity of PDK2 and only partially effective against PDK3 isoforms at 1.5 μM, thus highlighting their preferential activity against PDK1 and PDK4 isoforms. Interestingly, the most potent compounds **5i**, **5k**, **5l**, **5w**, **6h**, **6j** and **6s** were also subjected to IC_50_ value determination on the PDK1 isoform. As shown in Table 1, all 1,2,4-triazine derivatives dramatically decreased PDK1 catalytic activity at sub-micromolar/nanomolar concentrations, with IC_50_ values ranging from 0.01 to 0.1 µM, prominently outperforming the well-known PDK reference inhibitors DAP and DCA. Based on the obtained results, it is possible to draw some rational conclusions about structure–activity relationships (SARs) on PDK1 inhibition. In general, the presence of a methoxyl group in position 5 of one indole portion plays an important role in the inhibition of kinase activity, as well as the introduction of a methyl group at the indole N-atoms. In addition, the concomitant presence of the methoxyl group at position 5 of one indole unit and the halogen atom at the same position of the other indole portion led to an increase in the enzyme inhibitory activity.

Since the ATP binding pockets in PDKs and HSP90 are similar and taking into consideration that these 1,2,4-triazine derivatives conserved, a chemical scaffold resembling that of the well-known Hsp90 inhibitor Ganetespib, the most performant PDK inhibitors **5i**, **5k**, **5l**, **5w**, **6h**, **6j** and **6s** as well as derivatives **5s**, **5x**, **6i**, **6k**, **6l**, **6w** and **6x**, were also differentially screened for their ability to target HSP90. As reported in Figure 2B, only compound **6h** was extremely effective against HSP90, being able to completely abolish the enzyme activity at 1.5 µM. Conversely, derivatives **5i**, **5k**, **5l**, **5w**, **6j** and **6s** act as more selective PDK1 inhibitors, being able to decrease HSP90 catalytic activity by only 60–80% at equivalent doses.

### 2.3. Molecular Modeling Studies

All the diverse crystallographic structures analyzed in the present work, both from HSP90 and for the different PDK1, were downloaded from the Protein Data Bank and imported into the main window of the MOE suite. The specific experimental structure for each biological entity was chosen based on several factors, which comprise the crystallographic resolution, the eventual absence of protein portions, and the similarity between the scaffold of the co-crystallized ligand and one of the compounds of type **5** and **6** under evaluation in this study. In the end, the chosen systems for computational analyses were the ones with PDB code: 5J64 (method: X-ray diffraction, resolution: 1.38Å) [34] for HSP90, and 2Q8F (method: X-ray diffraction, resolution: 2.03Å) [35] for PDK1 (Supplementary Materials, Figure S99). Each of them was then subjected to proper preparation for computational handling. First, the MOE Structure preparation tool was used in order to rebuild the small missing loops in the structures, then each amino acid was assigned its dominant protonation state at pH 7.4 with the MOE Protonate 3D application. In the end, all the added hydrogen atoms were energetically minimized under the Amber10:EHT force field implemented in MOE [36].

The small molecules composing the triazine database under evaluation were also subjected to a proper preparation procedure, exploiting the tools of the QUACPAC package of the OpenEye suite. Specifically, the dominant tautomeric form for each compound was retrieved with the tautomers tool, then the three-dimensional coordinates were built with the program Omega. Then, the appropriate partial charges for each molecule were computed by exploiting the MolCharge application, and the proper protonation state was assigned to each molecular entity with the FixpKa tool (setting a pH value of 7.4).

With both proteins and ligands appropriately prepared, the molecular docking calculations were executed. The program used was PLANTS, and Ant Colony Optimization algorithm that has already proven to perform well in several conditions and scenarios, refs. [37,38] and the scoring function was set to PLANTS_CHEMPLP_. For each ligand, 50 poses were produced, which were then filtered in order to eliminate the ones presenting unfavorable electrostatic interaction with the target, or which were involved in steric clashes with the pocket. Moreover, in the case of HSP90, the remaining conformations were then passed through a 3D pharmacophore created with a consensus strategy, keeping the most common features of the different orthosteric crystallographic ligands of HSP90. The poses able to pass the pharmacophore were then visually inspected and the best for each molecule was chosen. 

**Figure 3:**
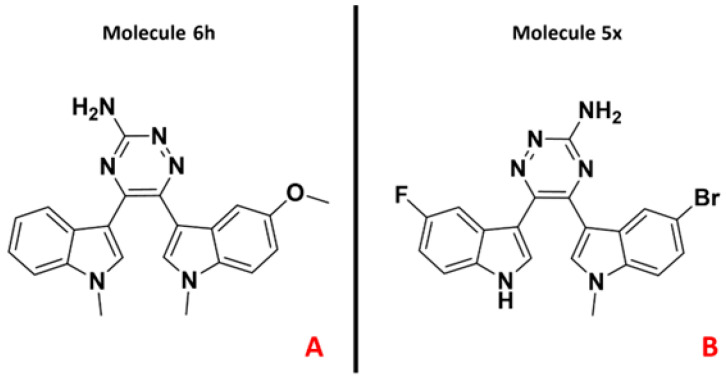
Representation of the two-dimensional structures of the two molecules chosen as references for the computational evaluation: **6h**, a potent ligand for both PDK1 and HSP90 (**A**), and **5x**, a weak binder, also in this case, for both HSP90 and PDK1 (**B**).

For simplicity, our computational analysis will focus on two specific molecules, one showing a very good inhibitory profile of both HSP90 (IC_50_ = 0.2 µM) and PDK1 (IC_50_ = 0.08 µM), which is compound **6h**, and one displaying worse potency for both the cited targets (IC_50_ > 1.5 µM for HSP90 and 0.7 µM for PDK1), which is the molecule with code **5x**. Both the molecules’ 2D structures are represented in Figure 3, while their best-bound conformation to HSP90 is depicted in Figure S100 of the Supplementary Materials.

**Figure 4:**
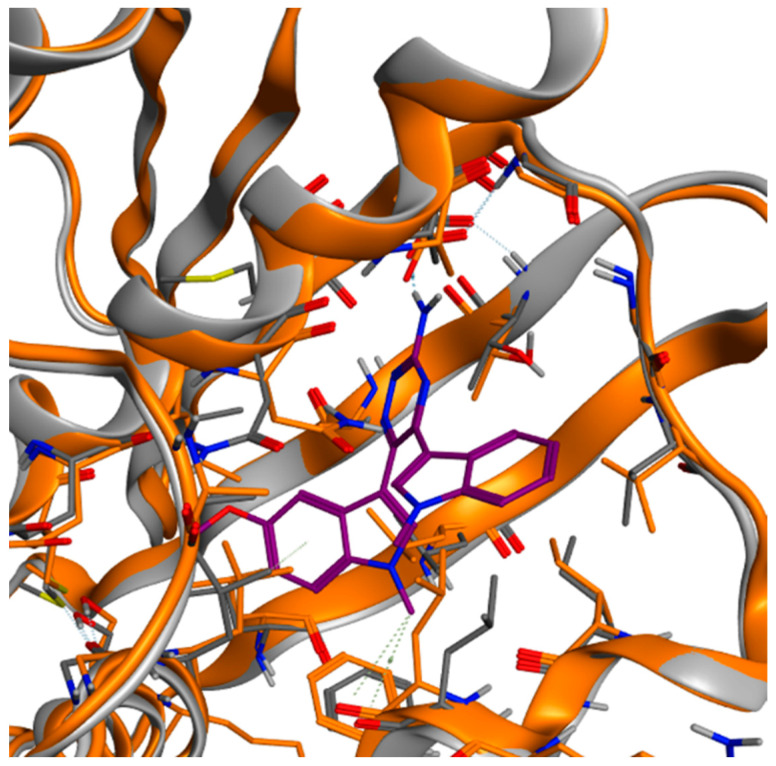
Depiction of the changes in the ATP-binding site resulting from the ligand-based homology model created on the crystallographic structure having PDB code 2Q8F. The potent ligand **6h** was kept in the binding pocket during the modeling run. The original crystal is colored orange, while the model obtained is colored grey. The manually placed ligand **6h** is colored purple. As can be seen, the small shifts in the lateral chains of some amino acids allow the formation of a larger volume inside the ATP-binding pocket, which has proved to be decisive to the ligand-binding of the molecules under examination. The image was created and rendered with MOE.

In the case of PDK1, the triazine database under evaluation underwent a molecular docking experiment with the very same procedure as for HSP90. Unfortunately, in the case of PDK1, the lack of experimental structures comprising a drug-like small-molecule ATP-competitive ligand bound to PDK1, made impossible the creation of a structure-based pharmacophore model. For this reason, the poses were just filtered in order to eliminate the ones presenting steric clashes or electrostatically unfavorable interactions with the pocket, and the results were carefully visually inspected. In this case, the outcomes of the docking experiment were much less encouraging, with the great majority of the molecules showing any conformation able to avoid steric clashes with the protein pocket. This is of course addressable to the fact that the protein considered is in its apo form, so the pocket is not in a conformation able to accept ligands of drug-like dimensions such as the ones in our database. It is important to say that at the present time (last update: 22 July 2022) no crystal structure of PDK1 with a drug-like small molecule in the ATP-binding site is present in the PDB. One of the strategies that can be adopted in this case, which is also the one that we chose, is the so-called “ligand-based homology modeling” [39]. This method consists of modeling the sequence of a protein (in our case, PDK1) on a template (which in our case is the apo form of PDK1 itself) in which the ligand of interest is placed inside the desired pocket before the modeling operation starts. This allows the protein to rebuild taking into account the presence of the ligand, and this mainly is reflected in the disposition of the side chains of the amino acids surrounding it. This way, the necessary volume in the binding site to accommodate the ligands is created, and some reasonable docking poses can be generated. In our case, we modeled the sequence of PDK1 using the crystal with PDB code 2Q8F as a template, in which the potent inhibitor **6h** was manually placed following a preliminary SAR hypothesis (also obtained from the inspection of the bound conformations of the ATP-competitive inhibitors of the other PDK isoforms, available in the PDB), with the aromatic amine moiety towards Asp318, in the upper portion of the ATP-binding pocket. A depiction of the comparison between the original protein structure and the one coming from homology modeling is reported in Figure 4. As can be seen, the major changes are related to the amino acid side chains around the ligand, as assessed by the very high level of superimposability with the initial crystallographic system (the measured RMSD between the a-carbons of the two structures is 0.33 Å).

With the ligand-based homology model, we re-executed the molecular docking calculations, keeping the same parameters as the first run. This time the results were much more appreciable, with a lot of ligands able to be properly placed inside the ATP-binding site. Of course, as we did for HSP90, we filtered the poses presenting steric clashes and unfavorable electrostatic interactions with the protein pocket.

The next operation was the validation of our binding hypothesis regarding **6h**. If we superpose the best pose coming from our protocol to the manually placed one, we notice that the two conformations are very similar in the protein binding site (Figure S101 of the Supplementary Materials). Indeed, the RMSD between the two was calculated to be 0.87 Å, giving credit to our preliminary binding hypothesis. 

Examining the best pose for compound **5x**, we notice that the interaction pattern is maintained, even if the on-target potency has proven to be lower. This does not affect our binding hypothesis, because it is reasonable that **5x** having a very similar structure with respect to compound **6h**, (as represented in Figure S102 of the Supplementary Materials), can bind following the same rules, to the ATP-binding site. Of course, the lower potency has to be addressed to factors that are not related merely to the in-site binding pose.

**Figure 5:**
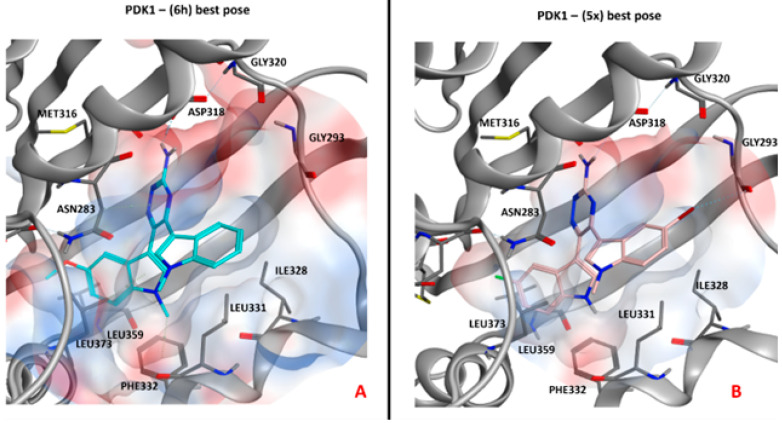
Depiction of the binding modes of the two compounds chosen as the reference for our study, located in the PDK1 ATP-binding site. (**A**) Representation of the binding pocket’s electrostatic surface for the selected pose produced by molecular docking of the ligand **6h** (colored cyan), which has passed through the steric and electrostatic filtering procedures. (**B**) Picture showing the binding pocket’s electrostatic surface for the selected pose produced by molecular docking of the ligand **5x** (colored in pink), which has passed through the steric and electrostatic filtering procedures. The images were created and rendered with MOE.

Focusing on PDK1, the reasons beneath the differences in potency are hardly addressable to some properties which can be retrieved from the binding poses. Even if it is true that the presence of the methoxyl group of compound **6h**, which is bigger than the simple fluorine or bromine atoms of compound **5x**, can help the molecule to properly occupy the volume of the small hydrophobic pocket nearby Met276, this is probably just a marginal effect to explain such a difference in the on-target activity. Figure 5 represents the binding poses already discussed, also highlighting the electrostatic surface of the binding pocket.

**Figure 6:**
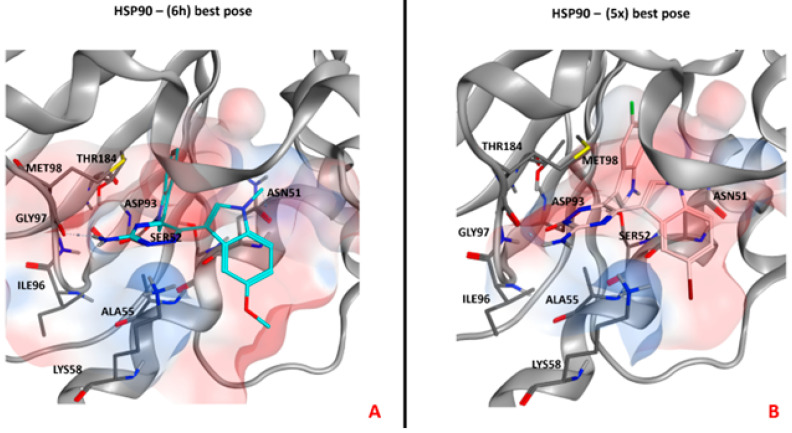
Depiction of the binding mode of the two compounds chosen as the reference for our study in the orthosteric HSP90 binding site, which also has the electrostatic surface highlighted. (**A**) Representation of the binding pocket’s electrostatic surface for the selected pose produced by molecular docking of the ligand **6h** (colored cyan), which has passed through the steric, electrostatic, and pharmacophoric filtering procedures. (**B**) Representation of the binding pocket’s electrostatic surface for the selected pose produced by molecular docking of the ligand **5x** (colored in pink), which has passed through the steric, electrostatic, and pharmacophoric filtering procedures. The images were created and rendered with MOE.

A very similar argument can be made for HSP90. Indeed, both the ligands considered in our study can properly allocate one of the two indole moieties in the inner pocket of the binding site, keeping the substituted one on the outer side. Moreover, both are able to establish a hydrogen bond with Gly97 in a very similar manner (Figure 6), making it very hard to depict the reasons behind the difference in potency in this case. A comprehensive comparison of the docking pose of each molecule present in the database able to produce a bound conformation with both proteins after the filtration procedures is reported in the file “Video_docking_HSP90_PDK1.mp4” (Video S1), available in the Supplementary Materials.

Some very important aspects to consider when evaluating the potency of a ligand with respect to a target are tightly bound to its stability in the protein pocket but, to an even higher extent, to the recognition path between ligand and receptor. Indeed, the dynamic events during the ligand approaching the binding site can be very crucial in the determination of the outcomes of the recognition with the protein, also making similar ligands show distinct potencies. One of the computational techniques able to capture these events is supervised molecular dynamics (SuMD) [40]. Indeed, this can be efficiently used to analyze the recognition pathway of small molecule ligands with a protein of interest, as already demonstrated in different scenarios on very distinct targets [41,42,43,44]. 

Currently, many experiments exploiting both classical and supervised molecular dynamics are ongoing in our lab for the investigation of this class of triazine ligands. 

**Figure 7:**
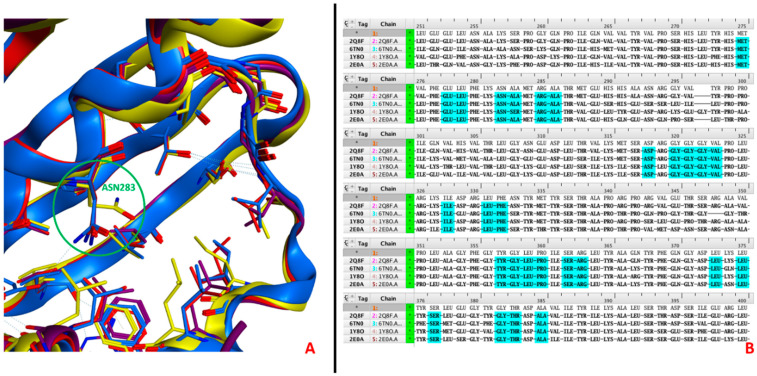
(**A**). The ATP-binding pocket of the four PDK isoforms aligned and superimposed to one PDK1. Looking into the detail, the crystal structures used were: 2Q8F for PDK1 (colored in yellow), 6TN0 for PDK2 (method: X-ray diffraction, resolution: 1.91 Å, colored in blue) [45], 1Y8O for PDK3 (method: X-ray diffraction, resolution: 2.48 Å, colored in red) [46], and 2E0A for PDK4 (method: X-ray diffraction, resolution: 1.86 Å, colored in purple) [47]. For each crystallographic structure, the very same preparation procedure described at the beginning of the section for PDK1 and HSP90 was executed. The residues depicted in sticks are helpful in giving a representation of the very high level of similarity among the ATP-binding pockets of the different PDK isoforms, which becomes absolute looking at the amino acids closest to the ligand. The residue Asn283 from PDK1 (labeled in green and highlighted in the image with the green circle), inversely to the other asparagine residues in that region, is oriented inwards to the pocket. This is because for this superimposition procedure we considered only crystal structures (and not models) and, as already said, no PDK1 crystal structure with a ligand in the ATP-binding pocket is present at the present date (last check: 22 July 2022). This obliged us to use the structure which did not have any ligand inside this pocket (2Q8F), which has the Asn283 side chains oriented in the inner part of the pocket. (**B**). Representation of the protein sequences of the four PDK isoforms aligned on PDK1. The residues highlighted in cyan are the ones closer to the ligand in the ATP-binding pocket. This depiction allows us to appreciate the very noticeable conservation of the residues located in this specific protein site. Both images were created and rendered with MOE. * List of PDB codes used for the analyses.

The results of such studies will be very important for addressing the problem of the selectivity of some of the ligands of the present database for specific isoforms of PDK (mainly PDK1 and PDK4). Indeed, from a structural point of view, the ATP-binding sites of the four PDK isoforms are almost identical to one another, as represented in Figure 7. 

The outcomes of the MD and SuMD experiments which are now ongoing will be very helpful in elucidating the reasons behind the peculiar shifts in activity of this class of ligands, and they will be extensively discussed in a future scientific work.

### 2.4. 2D and 3D Cytotoxicity Studies 

All newly synthesized compounds were then screened for their cytotoxic activity against two human PDAC cell lines, namely a KRAS-wild-type PDAC cell line (BxPC-3 cells) and a KRAS-mutant PDAC cell line (PSN-1 cells). For comparison purposes, the efficacy of the PDAC gold-standard chemotherapeutic gemcitabine [48], as well as of the well-known PDK inhibitors DCA and DAP were assessed under the same experimental conditions. The cytotoxicity parameters, expressed in terms of IC_50_ and obtained after 72 h of drug exposure by MTT assay, are listed in the Supplementary Materials (Table S2). The antiproliferative activities of the most representative compounds are reported in Table 2. 

As expected, DCA was completely ineffective at micromolar concentrations in determining a reduction in cancer cell viability (IC_50_ values > 1 μM). On the contrary, all tested compounds showed a very promising cytotoxic activity, with IC_50_ values in the low- or sub-micromolar range, being equally effective against KRAS wild-type and mutant cancer cells. In particular, all derivatives were also significantly more active than DAP in decreasing cancer cell proliferation. On the other hand, all derivatives were less cytotoxic compared with the reference chemotherapeutic drug gemcitabine, compound **6v** being the unique derivative showing a cytotoxicity profile resembling that of the reference clinically approved drug. The newly developed 1,2,4-triazine compounds were also screened against 3D spheroids of PDAC RAS wild-type and mutant cells, to further evaluate their anticancer potential in a more predictive environment. Three-dimensional cell cultures possess several features that more closely mimic the complex in vivo tumor architecture and physiology, consequently being majorly predictive of in vivo effectiveness [49]. In particular, glucose metabolism in 3D spheroids differs significantly from 2D cultures, being enormously more representative of the in vivo tumor metabolic conditions, thus emphasizing the need to use 3D cell models for investigating new putative drugs acting on tumor metabolism and on potential metabolic therapeutic targets [50]. The PDAC cancer spheroids were treated with tested compounds for 72 h, and cell viability was assessed by means of the acid phosphatase (APH) assay (Table 3). As for 2D cell studies, the efficacy of gemcitabine as well as of DCA and DAP were assessed under the same experimental conditions. All compounds were much more effective than gemcitabine and DAP against the three-dimensional models. Actually, gemcitabine was proven to be scarcely effective against 3D PDAC spheroids, eliciting IC_50_ values in the high-micromolar range. Similarly, the reference PDK inhibitors DAP showed average IC_50_ values of 78.2 and 87.4 µM against PSN-1 and BxPC-3 cancer cells, respectively. Notably, against 3D PDAC cell models, all 1,2,4-triazine compounds were, on average, much more effective against KRAS mutant PSN-1 cells compared with KRAS wild-type BxPC-3 cells. Among tested compounds, only a few derivatives were less active than DAP, whereas all the other compounds showed lower IC_50_ values (Supplementary materials, Table S3). In particular, derivatives **5g**, **5i** and **6l** proved to be the most efficacious against PNS-1 cells, with IC_50_ values of 4.9, 5.8 and 8.0 μM, respectively (Table 3). 

### 2.5. In Cell Mechanistic Studies

Based on PDK inhibitory activity and 3D cell cytotoxicity screenings, we selected compounds **5i**, **5k**, **5l**, **5w**, **6h**, **6j**, and **6s** as representative derivatives for further investigations aimed at deeply investigating their potential as PDK inhibitors on KRAS mutant PSN-1 PDAC cells. 

**Figure 8:**
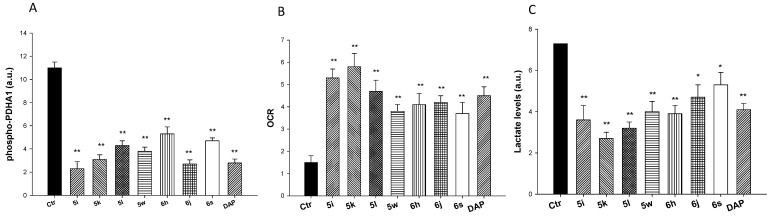
Effects on PDH phosphorylation, oxygen consumption and glycolytic metabolism in PSN-1 cells treated for 24 h with test compounds. (**A**) Phosphorylation levels of S232 PDH E1 alpha protein (PDHA1) were assessed by ELISA Kit (ab115343). (**B**) Changes in cellular oxygen consumption were assessed by the Mito-ID® Extracellular O_2_ Sensor Kit. Fluorescence was estimated at 360 nm (excitation) and 650 nm (emission). (**C**) Changes in lactate production were assessed by a lactate assay kit (Merk). Error bars indicate S.D. Data are the means ± SD of three independent experiments. * *p* < 0.05, ** *p* < 0.01.

As a first step, the ability of representative derivatives to target PDK in intact cancer cells was assessed by evaluating their effect on PDH phosphorylation of PSN-1 cancer cells. PSN-1 cells were treated for 24 h with IC_50_ concentrations (calculated after 24 h treatment of tested compounds and the phosphorylation levels of S232 PDH E1 alpha protein (PDHA1) were quantified by an ELISA Kit. Results reported in Figure 8A, clearly underline that tested compounds dramatically reduced PDH phosphorylation in treated PSN-1 cells. Interestingly, the reduction in phospo-PDHA1 levels was very similar to that exerted by the reference PDK inhibitor DAP, especially when considering **5i**, **5k** and **6j** derivatives. 

It is well-known that inhibition of PDKs activates PDC, leading to a switch of pyruvate metabolism from lactate production to oxidative phosphorylation [11]. On this basis, to prove the in-cell inhibition of PDKs by tested compounds, we evaluated the lactate formation and oxygen consumption (OCR) changes in KRAS mutant PSN-1 cancer cells.

Cells were treated with DAP or representative compounds at IC_50_ concentrations and the changes in OCR and in the intracellular lactate formation were monitored. As shown in Figure 8B, all tested derivatives significantly increased cellular oxygen consumption. In particular, derivatives **5i** and **5k** were more effective than DAP in increasing the OCR. Similarly, **5i**, **5k** and **5l** were more potent than DAP in decreasing lactate production in PSN-1 cancer cells (Figure 8C). It is important to note, however, that all the tested 1,2,4-triazine compounds induced a significant alteration of glycolytic and oxidative fluxes, as an important indication of their ability to target glycolytic metabolism in cancer cells. 

To confirm the metabolic shift towards oxidative pathways induced by tested compounds, we also evaluated their effects in terms of ROS formation and thiol redox state. PDK inhibitors promoting mitochondrial pyruvate oxidation determine a restoration of mitochondrial OXPHOS function, thus leading to cellular oxidative damage via an increase in ROS production and mitochondrial membrane potential decrease in Warburg-dependent cancer cells [51]. 

**Figure 9:**
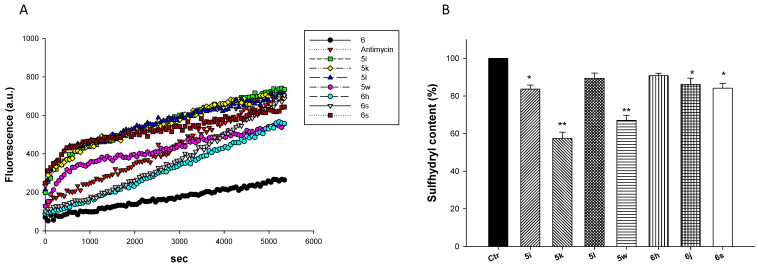
Effect on cellular redox homeostasis. (**A**) Effect of tested compounds on hydrogen peroxide formation in PSN-1 cells. Cells were pre-incubated in PBS/10 μM glucose medium for 20 min at 37 C in the presence of 10 nM CM-H_2_DCFDA and then treated with 5µM of test compounds or antimycin (3 µM). (**B**) Cellular thiol redox status. Sulfhydryl content in PSN1-treated cancer cells incubated for 24 h with test compounds. The sulfhydryl group amount was determined by the DTNB assay. Error bars indicate S.D. Data are the means ± SD of three independent experiments. * *p* < 0.05, ** *p* < 0.01.

PSN-1 cancer cells treated with representative compounds **5i**, **5k**, **5l**, **5w**, **6h**, **6j**, and **6s** showed an increase in the ROS basal level production (Figure 9A). Notably, treatment with derivatives **5i**, **5k**, **5l**, and **6j** determined an increase in basal hydrogen peroxide formation which was even higher than that provoked by antimycin, a classic inhibitor of the mitochondrial respiratory chain at the level of complex III. Consistently, tested compounds, although to different extents, were effective in reducing total cellular sulfhydryl contents in PSN-1-treated cells (Figure 9B). 

Overall, these results demonstrate that the newly developed 1,2,4-triazine compounds induced an oxidative shift in the redox status of PSN-1 cancer cells. These results are coherent with those highlighting their ability to target PDK and support the hypothesis that PDK represents major cellular targets for this class of compounds.

A persistent increase in the rate of ROS production and the induction of thiol redox stress can in turn prompt the collapse of mitochondrial membrane potential (ΔΨ) as well as loss of mitochondrial shape and integrity (swelling), possibly leading to the induction of cell apoptosis [52]. We hence evaluated the effect induced by test compounds in terms of modification of mitochondrial pathophysiological characteristics, such as mitochondrial membrane potential and morphological changes, as well as induction of cell death through apoptosis.

**Figure 10:**
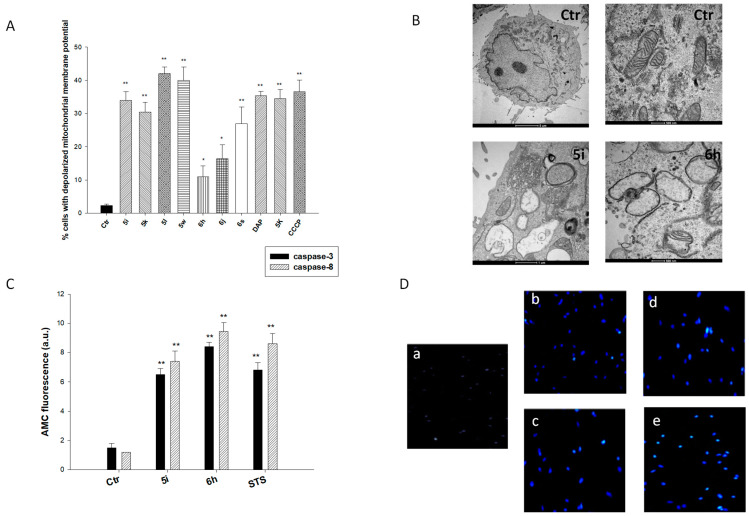
Antimitochondrial effect and induction of caspase-dependent apoptosis. (**A**). Effects of tested compounds on mitochondrial membrane potential. PSN-1 cells were treated with IC_50_ concentrations of tested compounds or CCCP (3 µM). The percentage of cells with hypopolarized mitochondrial membrane potential was determined by the Mito-ID^®^ Membrane Potential Kit. Fluorescence intensity was estimated at 490 (excitation) and 590 nm (emission). Error bars indicate S.D. * *p* < 0.05, ** *p* < 0.01. (**B**). TEM analysis of PSN-1 control cells or cells treated for 24 h with compounds **5i** and **6h**. (**C**). Effects of tested compounds on caspase activity. PSN-1 cells were incubated for 24 h with tested compounds or staurosporine and processed for caspase -3 and -8 activity. Error bars indicate S.D. ** *p* < 0.01. (**D**). Apoptosis induction. Hoechst staining of PSN-1 cells incubated for 48 h with IC_50_ doses of **5i** (**b**,**c**) and **6h** (**d**,**e**). (**a**) represents untreated PSN-1 cells as control.

Evident in results depicted in Figure 10A, the percentage of cells with hypopolarized mitochondrial membrane potential significantly increased in PSN-1 cells treated with representative compounds **5i**, **5k**, **5l**, **5w**, **6h**, **6j**, and **6s**. Remarkably, treatment with compounds **5l** and **5w** resulted in an increase of about 50% in the cell population with depleted ΔΨ, similar to that induced by the reference compound carbonyl cyanide-m-chlorophenylhydrazone (CCCP), tested under the same experimental conditions. 

In addition, TEM analyses were performed on PSN-1 cancer cells after 24 h treatment with IC_50_ concentrations of the representative compounds **5i** and **6h**. Images in Figure 10B clearly show that, with respect to control cells, **5i** and **6h** induced a dramatic swelling of the mitochondria, associated with decreased electron density of the inner membrane and matrix regions as well as disruption of cristae, as clear signs of their anti-mitochondrial effects. Altogether these data confirm that the newly developed PDK inhibitors elicited a substantial modification of mitochondria physiology and functioning. 

Apoptotic cell death can be triggered by a mitochondria-mediated or mitochondrial-independent pathways. In the former, caspase-8 and caspase-3 play an essential role [53]. Hence, to confirm the involvement of mitochondrial-driven apoptosis induced by tested compounds, we determined the activation of caspase-3 and -8 in PSN-1 cells. Staurosporine (STS), an alkaloid inducing apoptosis through caspase-3 and -8 activation was used as a positive control [54]. Interestingly, both caspases resulted in being significantly activated by treatment with selected compounds **5i** and **6h**. Caspase-3 and -8 cleavage reached values similar to that induced by the well-known caspase-dependent apoptosis inducer staurosporine (Figure 10C).

As a last mechanistic evaluation, the ability of selected compounds **5i** and **6h** to induce cancer cell death by means of apoptosis, was also assessed. Figure 10D shows the results obtained upon monitoring cellular morphological changes in PSN-1 cells treated for 48 h with IC_50_ doses of **5i** and **6h** and stained with the Hoechst 33258 fluorescent probe. Compared with control cells, cells treated with the tested compounds presented brightly stained nuclei and morphological features typical of cells undergoing apoptosis, such as chromatin condensation, thus confirming the ability of 1,2,4-triazine derivatives to induce cancer cell death by means of apoptosis.

### 2.6. In Vivo Studies

Finally, as in vitro cytotoxicity does not predict the ability of the compound to reach the tumor site and to kill cancer cells in the tumor mass, we also carried out preliminary in vivo studies comparing cisplatin, gemcitabine, and **5i** in the murine Lewis Lung Carcinoma (LLC) solid tumor model, obtained by implanting the LLC cell line intramuscularly as a 2 × 10^6^ cell inoculum into the right hind leg of 8-week-old male and female C57BL mice (24 ± 3 g body weight). The LLC is a highly aggressive and metastatic Kras-mutant (Kras^G12C^) syngeneic carcinoma model often used in preclinical trials for its reproducibility and, mostly because it entails the injection of immunologically compatible cancer cells into immunocompetent mice [55,56]. 

**Figure 11:**
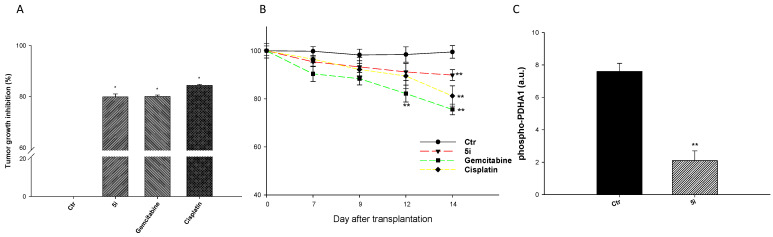
(**A**) Inhibition of tumor growth; days 7−14: animals received daily i.p. of **5i** (20 mg kg^−1^ in vehicle solution), gemcitabine (60 mg kg^−1^ in 0.9% saline solution) or cisplatin (3 mg kg^−1^ in saline solution). On day 15, animals were sacrificed. (**B**) Body weight changes of LLC-bearing C57BL mice treated with the vehicle or the tested compounds. (**C**) Evaluation of p-PDHA1 in tumor samples of treated mice. Phosphorylation level of S232 PDH E1 alpha protein (PDHA1) assessed by ELISA Kit (ab115343). Error bars indicate SD. * *p* < 0.05, ** *p* < 0.01.

Seven days after tumor inoculation the tumor-bearing mice were randomized into vehicle control and treatment groups. Control mice received the vehicle (1% DMSO (*v/v*) and 99% of a saline solution (*v*/*v*)), whereas treated groups received daily i.p. doses of **5i** (20 mg kg^−1^ in vehicle solution), gemcitabine (60 mg kg^−1^ in 0.9% saline solution) or cisplatin (3 mg kg^−1^ in saline solution). The tumor growth was evaluated at day 15, and the results are summarized in Figure 11A. As an estimation of the adverse side effects, changes in the body weights were monitored every two days (Figure 11B).

Although most potent in vitro, gemcitabine in vivo induced a reduction in tumor growth compared to the control group of about 80%, substantially equal to that induced by administration of **5i**, and only slightly lower than that resulting from cisplatin administration (~84% tumor inhibition). Remarkably, the time course of body weight changes indicated that treatment with **5i** induced a body weight loss lower than that induced by cisplatin and gemcitabine, both determining a significant anorexia, with a body weight loss ~20% (Figure 11B). As a proof-of-concept of PDK inhibitory activity of the newly developed 3-amino 1,2,4-triazines, we also assessed the phosphorylation level of S232-PDHA1 in tumor samples of animals treated with **5i**. Tumors of treated animals collected on day 15 were homogenized and phopho-S232-PDHA1 levels were quantified by an ELISA Kit. Results reported in Figure 11C, undoubtedly showed that **5i** dramatically reduced PDH phosphorylation in LLC-bearing mice, thus confirming the ability of the new derivative to target PDK in vivo, in a highly aggressive KRAS mutant animal model. 

## 3. Materials and Methods

### 3.1. Chemistry

#### 3.1.1. General Methods

The anhydrous solvents used for organic synthesis (butanol, heptane and diethyl ether) and the reagents were purchased from Sigma-Aldrich Co (St. Louis, MO, USA), Alfa Aesar (Haverhill, MA, USA), VWR International (Radnor, PA, USA), and Acros Organics (Waltham, MA, USA). Dichloromethane and toluene were purified and dried using calcium hydride and stored over 4 Å molecular sieves. All air- or moisture-sensitive reactions were performed using oven-dried glassware under an inert dry nitrogen atmosphere. Analytical thin-layer chromatography (TLC) was performed on silica gel 60 F254 plates (0.25 mm thickness) and the developed plates were examined under ultraviolet (UV) light. All melting points were taken on a Buchi–Tottoly capillary apparatus and were uncorrected. IR spectra were determined in bromoform with a Shimadzu FT/IR 8400S spectrophotometer and peaks were reported in wavenumber (cm^−1^). 1H and 13C NMR spectra were measured at 200 and 50 MHz, respectively, on DMSO-d6 solution, using a Bruker Avance II series 200 MHz spectrometer. Chemical shifts were described in parts per million (δ), coupling constants (J) were expressed in Hertz (Hz), and splitting patterns were reported as singlet (s), doublet (d), triplet (t), quartet (q), multiplet (m), doublet of doublets (dd) and triplet of doublets (td). Chromatography was performed with a column of MERK silica gel 230–400 mesh ASTM or FLASH40i Biotage chromatography or with Buchi Sepacore chromatography module (prepacked cartridge reference). Elementary analyses (C, H, N) were within ±0.4% of the theoretical values. 

The LC–MS was performed using an Agilent 1260 Infinity system (Agilent Technologies, Waldbronn, Germany), quaternary pump and autosampler in association with a Varian MS 500 Ion Trap Mass Spectrometer (Agilent Technologies, Waldbronn, Germany). The analysis was performed using an eclipse plus C18, 4.6 × 150 mm column using the following gradient: 0.0 min 80% A, 20% B; 10 min 20% A, 80% B, 14 min 20% A, 80% B, 15 min 80% A, 20% A (A: MilliQ water 1% formic acid, B: acetonitrile). The analysis lasted 15 min with a 0.750 mL/min flow. The compounds were detected by electrospray ionization ion trap mass spectrometry source under positive-ion conditions in turbo TDDS in the acquisition range *m*/*z* 300–550. The following parameters were used: capillary voltage 95.0 V, Needle Voltage +/− 5500 V, RF loading 81%.

#### 3.1.2. General Procedure for the Synthesis of (5-Bromo-1*H*-indol-3-yl)oxo-acetyl Chloride (**3b**)

To a solution of the commercially available 1*H*-indoles **1b** (10 mmol) in anhydrous diethyl ether (20 mL), oxalyl chloride (11.16 mmol, 0.95 mL) was added dropwise at 0 °C. The reaction mixture was left to stir at 0 °C for 3 h and then at room temperature for 1 h. The resulting solid product was collected by vacuum filtration and crystallized from diethyl ether. Yield: 96%; yellow solid; m.p.: 214.4 °C; IR (cm^−1^): 3212 (NH), 1755 (CO); 1639 (CO); ^1^H NMR (DMSO-*d_6_,* 200 MHz,) d: 7.43 (dd, 1H, *J* = 8.6, 2.0 Hz), 7.54 (d, 1H, *J* = 8.6 Hz), 8.30 (d, 1H, *J* = 2.0 Hz), 8.49 (d, 1H, *J* = 3.3 Hz), 12.62 (s, 1H); ^13^C{^1^H} NMR (DMSO-*d_6_*, 50 MHz) δ: 110.8, 114.8, 116.0, 123.2, 126.2, 127.8, 136.2, 139.0, 165.0, 180.2; Anal. calculated for C_10_H_5_BrClNO_2_ (MW: 286.51): C, 41.92; H, 1.76; N, 4.89%. Found: C, 41.83; H, 1.81; N, 4.97%.

#### 3.1.3. General Procedure for the Preparation of Bis(1*H*-indol-3-yl)ethane-1,2-diones (**4a–ab**)

To a suspension of acyl chloride **3a–h** (2.41 mmol) in a mixture of DCM/heptane 2:1 (7.2 mL), under a nitrogen atmosphere, aluminum trichloride (10.84 mmol) was added in portions in a time interval of 3–5 min. The reaction mixture was stirred at room temperature for a few minutes and then a solution of the proper indole **1a–d** or **2a–d** (3.98 mmol) in DCM (2.4 mL) was added dropwise. The reaction mixture was left to stir at room temperature for 2 h, until completion of the reaction. The resulting solution was poured into crushed ice, until the destruction of the aluminum trichloride, which is manifested by the cessation of effervescence. The resulting solid precipitate was collected by vacuum filtration, washed with water, dried under *vacuum* for 24 h, and purified by column chromatography using DCM/ethyl acetate 9:1 as eluent to give the desired products **4a–ab**. The experimental data are reported in the Supplementary materials.

#### 3.1.4. General Procedure for the Preparation of 5,6-di(1*H*-Indol-3-yl)-1,2,4-triazin-3-amine (**5**–**6**)

A suspension of the appropriate derivatives **4a–ab** (5 mmol) and aminoguanidine bicarbonate (10 mmol) in anhydrous *n*- butanol (20 mL) was heated under reflux for 3 h. The precipitate, which formed upon cooling, was collected by vacuum filtration, washed with n-butanol, and dried under vacuum for 24 h. The residue was obtained as a mixture of two diastereomers and the purification of the single enantiomers was afforded by column chromatography (CC) using dichloromethane/methanol 98:2 and 97:3 as eluent for the isolation of enantiomers **5** and **6**, respectively. The experimental data are reported in the Supplementary materials.

### 3.2. Software Overview

All the basic molecular modeling tasks were executed within the Molecular Operating Environment (MOE) suite [57]. The proteins which were analyzed in the present study were downloaded from the Protein Data Bank [58] and properly prepared with the MOE-dedicated tools. The homology modeling operations have also been carried out exploiting the MOE homology modeling application. On the other hand, the small molecule protagonists of the present work have been properly prepared for computational manipulation and docking calculation using the tools of the QUACPAC (OpenEye Scientific Software 2016) [59] package belonging to the OpenEye suite (OpenEye Scientific Software Inc. OEChem; OpenEye Scientific Software Inc.: Santa Fe, NM, USA, 2016). The molecular docking experiments were executed with the program PLANTS 1.2 [60], which is based on an Ant Colony Optimization algorithm (the tool was developed by Tübingen University and is free for academics). The hardware exploited for all the computational operations was a 20 CPU Linux Workstation (Inteli9-9820X).

### 3.3. PDK 1–4 Kinase Assay

The in vitro inhibitory activity of tested compounds on recombinant human PDK1-4 isoforms (Abcam, Cambridge, MA, USA) was evaluated by using the ADPGlo™ Kinase Assay kit from Promega (Promega Corporation, Madison, WI, USA) following the instructions of the manufacturer. Briefly, the assay was performed in 384-well plates by using PDK1-4 isoforms incubated for 30 min at room temperature with 1.5 µM of tested compounds. The reaction was then started by the addition of substrate/ATP for 60 min at room temperature. Subsequently, ADP-Glo™ reagent was added and incubated for 40 min prior to kinase detection reagent addition. After 30 min, the luminescence was measured with a Tecan plate reader (Tecan Infinite^®^ M200). Raw data were normalized to the values of control wells and luminescence values for each drug dose were expressed as a percentage of the luminescence of control untreated wells. The IC_50_ values, the drug concentrations that reduce the mean luminescence by 50% with respect to control untreated wells, were calculated by the four-parameter logistic (4-PL) model. The evaluation was based on means from at least three independent experiments.

### 3.4. Heat Shock Protein 90 (HSP90) Inhibition Assay

The in vitro inhibitory activity of tested compounds on recombinant human HSP90 was assessed by The Transcreener™ ADP kit (Bellbrook Labs) according to the manufacturer’s protocol. Human recombinant HSP90 protein was incubated with tested compounds in the presence of 250 μM ATP at 37 °C for 30 min, 4°C for 30 min and ATPase activity was stopped *via* the addition of the stop and detect buffer. Following this, samples were incubated for 1 h at room temperature with the ADP2 Antibody and the ADP Alexa594 Tracer. The fluorescence intensity polarization of samples was measured with a Tecan plate reader (Tecan Infinite^®^ M200). The evaluation was based on means from at least three independent experiments.

### 3.5. Experiments with Cultured Human Cancer Cells

All tested compounds were dissolved in DMSO just before the experiment, and a calculated amount of drug solution was added to the cell growth medium to a final solvent concentration of 0.5%, which had no detectable effects on cell viability. Dichloroacetate (DCA), 2,2-dichloroacetophenone (DAP) and gemcitabine hydrochloride were purchased by Merck KGaA (Darmstadt, Germany).

### 3.6. Cell Cultures

Human pancreatic PSN-1 and BxPC-3 carcinoma cell lines were obtained from American Type Culture Collection (ATCC, Rockville, MD, USA). Cell lines were maintained in the logarithmic phase at 37°C in a 5% carbon dioxide atmosphere using RPMI-1640 medium (EuroClone) containing 10% fetal calf serum (EuroClone, Milan, Italy), antibiotics (50 units/mL penicillin and 50 μg/mL streptomycin) and 2 mM l-glutamine.

### 3.7. MTT Assay

The growth inhibitory effect on tumor cells was evaluated by means of the MTT assay. Briefly, 3–8 × 10^3^ cells/well, dependent upon the growth characteristics of the cell line, were seeded in 96-well microplates in growth medium (100 μL). After 24 h, the medium was removed and replaced with a fresh one containing the compound to be studied at the appropriate concentration. Triplicate cultures were established for each treatment. After 72 h, each well was treated with 10 μL of a 5 mg/mL MTT saline solution, and following 5 h of incubation, 100 μL of a sodium dodecyl sulfate (SDS) solution in HCl 0.01 M was added. After overnight incubation, cell growth inhibition was detected by measuring the absorbance of each well at 570 nm using a Bio-Rad 680 microplate reader. The mean absorbance for each drug dose was expressed as a percentage of the control untreated well absorbance and plotted vs. drug concentration. IC_50_ values, the drug concentrations that reduce the mean absorbance at 570 nm to 50% of those in the untreated control wells, were calculated by the four-parameter logistic (4-PL) model. The evaluation was based on means from at least four independent experiments.

### 3.8. Spheroid Cultures

Spheroid cultures were obtained by seeding 2.5 × 10^3^ PSN-1 cancer cells/well in a round-bottom non-treated tissue culture 96-well plate (Greiner Bio-one, Kremsmünster, Austria) in phenol red-free RPMI-1640 medium (Sigma Chemical Co., St. Louis, MO, USA) containing 10% fetal calf serum and supplemented with 20% methylcellulose stock solution.

### 3.9. Acid Phosphatase (APH) Assay

An APH-modified assay was used for determining cell viability in 3D spheroids, as previously described [61]. Briefly, the pre-seeded spheroids were treated with fresh medium containing the compound to be studied at the appropriate concentration (range 2.5–200 μM). Triplicate cultures were established for each treatment. After 72 h, each well was treated with 100 μL of the assay buffer (0.1 M sodium acetate, 0.1% Triton-X-100, supplemented with ImmunoPure p-nitrophenyl phosphate; Sigma Chemical Co., Ltd.) and, following 3 h of incubation, 10 μL of 1 M NaOH solution was added. The inhibition of the cell growth induced by the tested compounds was detected by measuring the absorbance of each well at 405 nm, using a Bio-Rad 680 microplate reader. The mean absorbance for each drug dose was expressed as a percentage of the control untreated well absorbance (T/C) and plotted vs. drug concentration. IC_50_ values, the drug concentrations that reduce the mean absorbance at 405 nm 50% of those in the untreated control wells, were calculated by the four-parameter logistic (4-PL) model. The evaluation was based on means from at least four independent experiments.

### 3.10. Cellular PDH Phosphorylation

PSN-1 cells (2 × 10^5^) were seeded in a six-well plate in growth medium (4 mL). After 24 h, cells were incubated for 24 h with IC_50_ concentrations of tested compounds. Subsequently, the phosphorylation level of S232 PDH E1 alpha protein (PDHA1) was assessed by ELISA Kit (Abcam) following the manufacturer’s instructions. The amount of phosphorylated S232 PDHA1 protein was detected by measuring the absorbance of each well at 600 nm, using a Bio-Rad 680 microplate reader.

### 3.11. Cellular Oxygen Consumption

PSN-1 cells (2 × 10^5^) were seeded in a six-well plate in growth medium (4 mL). After 24 h, cells were incubated for 60 min with IC_50_ concentrations of tested compounds. Subsequently, oxygen consumption was assessed by Mito-ID^®^ Extracellular O_2_ Sensor Kit according to manufacturer’s instructions. Fluorescence was measured using a Tecan microplate reader (Ex 340–400 nm, Em 630–680 nm).

### 3.12. Cellular Lactate Production

PSN-1 cells (2 × 10^5^) were seeded in a six-well plate in a growth medium (4 mL). After 24 h, cells were incubated for 60 min with IC_50_ concentrations of tested compounds. Changes in lactate production were assessed via colorimetric microplate assay (Lactate assay kit^®^, Merk, St. Louis, MO, USA) according to the manufacturer’s instructions. The intracellular lactate concentration was detected by measuring the absorbance of each well at 570 nm, using a Bio-Rad 680 microplate reader.

### 3.13. Mitochondrial Membrane Potential (ΔΨ)

The ΔΨ was assayed using the Mito-ID^®^ Membrane Potential Kit according to the manufacturer’s instructions (Enzo Life Sciences, Farmingdale, NY, USA) as previously described. Briefly, PSN-1 cells (8 × 10^3^ per well) were seeded in 96-well plates; after 24 h, cells were washed with PBS and loaded with Mito-ID Detection Reagent for 30 min at 37 °C in the dark. Afterward, cells were incubated with tested compounds for 60 min. CCCP (Carbonyl cyanide 3-chlorophenyl-hydrazone) was used as the positive control. Fluorescence intensity was estimated using a VICTOR X3 (PerkinElmer, Waltham, MA, USA) plate reader at 490 (excitation) and 590 nm (emission).

### 3.14. ROS Production

The production of ROS was measured in PSN-1 cells (10^4^ per well) grown for 24 h in a 96-well plate in RPMI medium without phenol red (Sigma Aldrich, Germany). Cells were then washed with PBS and loaded with 10 μM 5-(and-6)-chloromethyl-2′,7′-dichlorodihydrofluorescein diacetate acetylester (CM–H_2_DCFDA) (Molecular Probes-Invitrogen, Eugene, OR, USA) for 45 min, in the dark. Afterward, cells were washed with PBS and incubated with tested compounds. Fluorescence increase was estimated utilizing the wavelengths of 485 nm (excitation) and 527 nm (emission) in a VICTOR X3 (PerkinElmer, USA) plate reader. Antimycin (3 μM, Sigma Aldrich), a potent inhibitor of Complex III in the electron transport chain, was used as the positive control.

### 3.15. Quantification of Thiols

PSN-1 cells (2 × 105) were seeded in a six-well plate in growth medium (4 mL). After 24 h, cells were incubated for 24 h with IC_50_ concentrations of tested compounds. Subsequently, the thiol content was measured as previously described [62].

### 3.16. TEM Analysis

About 10^6^ PSN-1 cancer cells were seeded in 24-well plates and, after 24 h incubation, were treated with IC_50_ concentrations of tested compounds and incubated for an additional 24 h. Cells were then washed with cold PBS, harvested, and directly fixed with 1.5% glutaraldehyde buffer with 0.2 M sodium cacodylate, pH 7.4. After washing with buffer and postfixation with 1% OsO_4_ in 0.2 M cacodylate buffer, specimens were dehydrated and embedded in epoxy resin (Epon Araldite). Sagittal serial sections (1 μm) were counterstained with toluidine blue; thin sections (90 nm) were given contrast by staining with uranyl acetate and lead citrate. Micrographs were taken with a Hitachi H-600 electron microscope (Hitachi, Tokyo, Japan) operating at 75 kV. All photos were typeset in Corel Draw 11.

### 3.17. Caspase Activity

PSN-1 cells (1 × 10^6^) were treated for 24 h with the IC_50_ doses of tested compounds, harvested and homogenized in a lysis buffer (1% Triton X-100, 320 nM sucrose, 5 mM EDTA, 10 mM Tris–HCl and 2 mM DTT (1,4-dithio-DL-threitol) buffer (pH 7.6)). Protein aliquots (100 μg) were stained at 37 °C for 60 min with fluorescent caspase-3 (N-acetyl-Asp-Glu-Val-Asp-AMC, AMC = 7-amino-4-methylcoumarin) or caspase-8 (Ac-Val-Glu-Thr-Asp-AMC) substrates. Substrate hydrolysis was measured after 60 min by monitoring the release of AMC using a a Tecan microplate reader (excitation at 370 nm, emission at 460 nm).

### 3.18. Cell Death Induction

PSN-1 cells were seeded into 8-well tissue-culture slides (BD Falcon, Bedford, MA, USA) at 5 × 10^4^ cells/well (0.8 cm^2^). After 24 h, the cells were washed twice with PBS and, following 48 h of treatment with IC_50_ doses of the tested compound, cells were stained for 5 min with 10 µg/mL of Hoechst 33258 (20-(4-hydroxyphenyl)-5-(4-methyl-1-piperazinyl)-2,50-bi-1H-benzimidazole trihydrochloride hydrate, Merk) in PBS. Samples were examined at 5× and 40× magnification in a Zeiss LSM 800 confocal microscope using the Zeiss ZEN 2.3 software system (Zeiss, Oberkochen, Germany).

### 3.19. In Vivo Anticancer Activity

All studies involving animal testing were carried out in accordance with ethical guidelines for animal research acknowledging the European Directive 2010/63/UE as to the animal welfare and protection and the related codes of practice. The mice were purchased from Charles River, housed in steel cages under controlled environmental conditions (constant temperature, humidity, and 12 h dark/light cycle), and alimented with commercial standard feed and tap water ad libitum. The Lewis Lung Carcinoma (LLC) cell line was purchased from ECACC, United Kingdom. The LLC cell line was maintained in DMEM (Euroclone) supplemented with 10% heat-inactivated fetal bovine serum (Euroclone), 10 mM L-glutamine, 100 U mL^−1^ penicillin, and 100 μg mL^−1^ streptomycin in a 5% CO_2_ air incubator at 37 °C. The LLC was implanted intramuscularly (i.m.) as a 2 × 10^6^ cell inoculum into the right hind leg of 8-week-old male and female C57BL mice (24 ± 3 g body weight). After 7 days from tumor implantation (visible tumors), mice were randomly divided into 4 groups (8 animals per group) and subjected to daily i.p. administration. Control mice received the vehicle (1% PEG2000 (*v*/*v*) and 99% of a saline solution (*v*/*v*)), whereas treated groups received daily doses of **5i** (20 mg kg^−1^ in vehicle solution), gemcitabine (60 mg kg^−1^ in 0.9% saline solution, iv) or cisplatin (3 mg kg^−1^ in saline solution, iv). At day 15, animals were sacrificed, the legs were amputated at the proximal end of the femur, and the inhibition of tumor growth was determined from the difference in the weights of the tumor-bearing leg and the healthy leg of the animals, expressed as a percentage referenced to the control animals. Body weight was measured at day 0 and from day 7 onwards every 2 days and was taken as a parameter for systemic toxicity. All presented values are the means ± SD of no less than three measurements.

### 3.20. PDH Phosphorylation in Mice Tumor Tissues

The phosphorylation level of S232 PDH E1 alpha protein (PDHA1) in LLC tissue homogenates was assessed by ELISA Kit (Abcam) following the manufacturer’s instructions. The amount of phosphorylated S232 PDHA1 protein was detected by measuring the absorbance of each well at 600 nm, using a Bio-Rad 680 microplate reader.

### 3.21. Statistical Analysis

All values are the means ± SD of no less than three measurements. Multiple comparisons were made by ANOVA followed by the Tukey–Kramer multiple comparison test (* *p* < 0.05, ** *p* < 0.01), using GraphPad software 9.0.

## 4. Conclusions

Pyruvate dehydrogenase kinases (PDKs), due to their key role in regulating metabolic flux linking the glycolytic pathway and tricarboxylic acid (TCA) cycle, represent relevant pharmacological targets for the design of a new class of anticancer drugs, especially against pancreatic ductal adenocarcinoma (PDAC), in which PDK1 was more widely expressed, facilitating increased anaerobic metabolism and apoptosis resistance under conditions of hypoxia and glucose deprivation.

Despite PDK inhibition being validated as an antitumor mechanism, no drug is currently available in the clinic. The only PDK inhibitor that has entered phase II clinical, is DCA; however, several side effects associated with weak anticancer activity and excessive drug dose (100 mg/kg), have led to its limitation in clinical application. Building upon a molecular hybridization approach, a library of forty-nine 3-amino-1,2,4-triazine derivatives has been designed, synthesized, and characterized for their PDK inhibitory activity using in silico, in vitro, and in vivo assays. Biochemical screening showed that most synthesized compounds are potent and subtype-selective inhibitors of PDK, totally suppressing the enzymatic activity of PDK1 at 1.5 µM. Since no crystal structure of PDK1 with a drug-like small molecule in the ATP-binding site is present in the PDB, through the “ligand-based homology modeling” method, the sequence of PDK1 was rebuild, with the most potent inhibitors docked inside the desired ATP-binding pocket. According to biochemical screening, molecular modeling studies revealed that a lot of the ligands are able to be properly placed inside the ATP-binding site of PDK1. Interestingly, 2D and 3D cell studies revealed their ability to induce human cancer cell death in the low micromolar range, being extremely effective against KRAS mutated cancer cells with a better cytotoxicity profile than that shown by DCA and resembling that of the reference clinically approved drug gemcitabine. Detailed cellular mechanistic studies performed with the most promising derivatives confirmed their ability to hamper the PDC/PDK axis, to determine mitochondrial damage, to increase cellular oxygen consumption, and decrease lactate production. Additionally, cell death studies revealed their ability to induce cancer cell death by apoptosis. The in vivo treatment of the KRAS-mutated LLC model with the most representative compound **5i** proved that this compound was effective in inducing about 80% reduction in the tumor mass, similar to cisplatin and gemcitabine, but with a body weight loss significantly lower than that induced by reference drugs. As a further proof-of-concept of molecular mechanism, the ELISA assay performed in LLC-bearing mice tumor samples revealed a significant inhibition of S232-PDHA1 phosphorylation, confirming PDK as a downstream target of the newly developed 3-amino-1,2,4-triazines. Collectively, the data highlights the promising anticancer potential of these novel derivatives, targeting PDK, towards obtaining clinical candidates for combatting highly aggressive KRAS-mutant pancreatic ductal adenocarcinoma. 

## Figures and Tables

**Table 1:** Inhibition of isolated PDK isoforms. IC_50_ values were calculated by a four-parameter analyzed logistic model (*p* < 0.05). DCA and DAP were reported as reference compounds. Data represent mean values from three independent experiments. S.D. = standard deviation.

Cpd	IC_50_ (µM) ±S.D. PDK1
**5i**	0.06 ± 0.02
**5k**	0.01 ± 0.003
**5l**	0.03 ± 0.004
**5w**	0.1 ± 0.04
**6h**	0.08 ± 0.01
**6j**	0.07 ± 0.02
**6s**	0.09 ± 0.04
**DCA**	>100
**DAP**	>100

**Table 2:** Antiproliferative activity of the most representative compounds against PSN-1 and BxPC3 cancer cells evaluated in a 2D cytotoxicity assay.

Cpd	IC_50_ (µM) ±S.D. PSN-1	IC_50_ (µM) ±S.D. BxPC-3	Cpd	IC_50_ (µM) ±S.D. PSN-1	IC_50_ (µM) ±S.D. BxPC-3
**5b**	0.5 ± 0.2	0.4 ± 0.1	**6j**	0.6 ± 0.1	0.20 ± 0.04
**5d**	0.30 ± 0.04	0.4 ± 0.1	**6o**	0.40 ± 0.04	0.30 ± 0.03
**5j**	0.4 ± 0.1	0.10 ± 0.04	**6v**	0.20 ± 0.03	0.10 ± 0.02
**5o**	0.5 ± 0.1	0.20 ± 0.03	**6x**	0.30 ± 0.04	0.3 ± 0.1
**5s**	0.5 ± 0.2	2.2 ± 0.6	**6y**	0.60 ± 0.04	0.3 ± 0.1
**5t**	0.4 ± 0.1	1.4 ± 0.2	**Gemcitabine**	0.10 ± 0.04	0.020 ± 0.003
**5v**	0.20 ± 0.04	0.20 ± 0.03	**DCA**	>1000	>1000
**5x**	0.3 ± 0.1	0.20 ± 0.04	**DAP**	10.2 ± 1.6	1.06 ± 0.9

Cells (3 × 10^3^) were treated for 72 h with increasing concentrations of tested compounds. Cytotoxicity was assessed by the MTT test. IC_50_ values were calculated by a four-parameter logistic model (*p* < 0.05). Gemcitabine, DCA and DAP were reported as reference compounds. Data represent mean values from at least three independent experiments. S.D. = standard deviation.

**Table 3:** Antiproliferative activity of the most representative compounds against PSN-1 and BxPC3 cancer cells evaluated in a 3D cytotoxicity assay.

Cpd	IC_50_ (µM) ±S.D. PSN-1	IC_50_ (µM) ±S.D. BxPC-3	Cpd	IC_50_ (µM) ±S.D. PSN-1	IC_50_ (µM) ±S.D. BxPC-3
**5g**	5.8 ± 0.8	19.5 ± 2.1	**6h**	24.3 ± 1.4	33.4 ± 3.6
**5i**	4.9 ± 0.8	34.1 ± 1.5	**6j**	18.1 ± 2.3	41.2 ± 3.6
**5j**	10.4 ± 1.6	16.7 ±2.0	**6k**	9.4 ± 0.2	22.3 ± 2.6
**5k**	15.9 ± 2.3	67.9 ± 3.7	**6l**	8.0 ± 0.1	8.8 ± 0.4
**5l**	8.7 ± 0.5	52.1 ± 3.3	**6s**	22.3 ± 2.3	29.2 ± 1.4
**5q**	8.4 ± 0.5	8.6 ± 0.3	**Gemcitabine**	159.5 ± 5.6	102.6 ± 6.3
**5u**	9.2 ± 0.7	16.6 ± 0.02	**DCA**	>1000	>1000
**5w**	15.3 ± 2.8	12.2 ± 1.3	**DAP**	78.2 ± 6.8	87.4 ± 8.9

Spheroids (2.5 × 10^3^ cells/well) were treated for 72 h with increasing concentrations of tested compounds. The growth-inhibitory effect was evaluated by means of the acid phosphatase (APH) test. IC_50_ values were calculated from the dose–survival curves using a four-parameter logistic model (*p* < 0.05). S.D. = standard deviation.

## Data Availability

Not applicable.

## Supplementary Materials

The supporting information can be downloaded at: https://www.mdpi.com/article/10.3390/ijms24043679/s1.
