# Supplementary material for: Discovery of the 3-Amino-1,2,4-triazine-Based Library as Selective PDK1 Inhibitors with Therapeutic Potential in Highly Aggressive Pancreatic Ductal Adenocarcinoma
Source: Int J Mol Sci. 2023 Feb 12;24(4):3679. doi: 10.3390/ijms24043679 (PMC9959349; doi:10.3390/ijms24043679)
Supplement: Supplementary file 1 [file ijms-24-03679-s001.zip › SI (ijms-2166785).pdf]

Supplementary Materials

Discovery of the 3-Amino-1,2,4-triazine-based Library as Selective PDK1 Inhibitors with Therapeutic Potential in Highly Aggressive Pancreatic Ductal Adenocarcinoma

Daniela Carbone[1†], Michele De Franco[2†], Camilla Pecoraro[1], Davide Bassani[3], Matteo Pavan[3], Stella Cascioferro[1], Barbara Parrino[1], Girolamo Cirrincione[1], Stefano Dall'Acqua[2], Stefano Moro[3], Valentina Gandin[2*] and Patrizia Diana[1*]

[1] Department of Biological, Chemical, and Pharmaceutical Sciences and Technologies (STEBICEF), University of Palermo, Via Archirafi 32, 90123, Palermo, Italy

[2] Department of Pharmaceutical and Pharmacological Sciences, University of Padova, Via F. Marzolo 5, 35131, Padova, Italy

[3] Molecular Modeling Section (MMS), Department of Pharmaceutical and Pharmacological Sciences, University of Padova, Via F. Marzolo 5, 35131 Padova, Italy

* Correspondence: patrizia.diana@unipa.it (P.D.); valentina.gandin@unipd.it (V.G.)

† These authors contributed equally to this work.

Table of Contents

Contents	Page	Table/Figure
Experimental data for synthesized compounds **4a-ab, 5a-ab, 6h-ab**	1-12	-
3-Amino-1,2,4-triazine derivatives **5,6** and their relative yields	13	Table S1
^{1}H NMR spectra of synthesized compounds **5a-ab, 6h-ab**	14-62	Figures S1-S49
Chromatograms and MS spectra of synthesized compounds **5a-ab, 6h-ab**	63-87	Figures S50-S98
Antiproliferative activity of compounds **5a-ab** and **6h-ab** against PSN-1 and BxPC3 cancer cells evaluated in a 2D cytotoxicity assay.	88	Table S2
Antiproliferative activity of compounds **5a-ab** and **6h-ab** against PSN-1 and BxPC3 cancer cells evaluated in a 3D cytotoxicity assay.	89	Table S3
Molecular modeling supplementary images	90-91	Figures S99-S102

Experimental data

*Bis(1H-indol-3-yl)ethane-1,2-dione (**4a**).* Yield: 62% from indole **1a** (R=H) and acyl chloride **3a** (R^2=H, R^3=H); yellow solid; m.p.: 273.3 °C; IR (cm^{-1}): 3301 (NH), 1601 (CO); ^{1}H NMR (DMSO-*d*$_6$, 200 MHz) δ: 7.32-7.38 (m, 4H), 7.58-7.62 (m, 2H), 8.28-8.35 (m, 4H), 12.32 (s, 2H); ^{13}C{^{1}H} NMR (DMSO-*d*$_6$, 50 MHz) δ: 112.4, 112.5,121.3, 122.4, 123.4, 125.6, 136.7, 137.3, 188.8; *Anal.* Calculated for $C_{18}H_{12}N_2O_2$ (MW: 288.30) C, 74.99; H, 4.20; N, 9.72%. Found: C, 74.71; H, 4.45; N, 9.59%.

*Bis(5-bromo-1H-indol-3-yl)ethane-1,2-dione (**4b**).* Yield: 66% from indole **1b** (R=Br) and acyl chloride **3b** (R^2=Br, R^3=H); yellow solid; m.p. 273.3 °C; IR (cm^{-1}): 3173 (NH), 1625 (CO); ^{1}H NMR (DMSO-*d*$_6$, 200 MHz) δ: 7.44 (dd, 2H, *J*=8.6, 1.9 Hz), 7.53 (d, 2H, *J*=8.6 Hz), 8.35 (s, 2H), 8.43 (d, 2H, *J*=1.9 Hz), 12.47 (s, 2H); ^{13}C{^{1}H} NMR (DMSO-*d*$_6$, 50 MHz) δ: 111.7, 114.7, 115.2, 123.4, 126.1, 127.4, 135.5, 138.6, 187.8; *Anal.* Calculated for $C_{18}H_{10}Br_2N_2O_2$ (MW: 446.09) C, 48.46; H, 2.26; N, 6.28%. Found: C, 48.64; H, 2.09; N, 6.47%.

*Bis(5-fluoro-1H-indol-3-yl)ethane-1,2-dione (**4c**).* Yield: 61% from indole **1c** (R=F) and acyl chloride **3c** (R^2=F, R^3=H); brown solid; m.p.: 392.0 °C; IR (cm^{-1}): 3207 (NH), 1608 (CO); ^{1}H NMR (DMSO-*d*$_6$, 200 MHz,) δ: 7.17 (td, 2H, *J*=9.3, 9.1, 2.6 Hz), 7.57 (dd, 2H, *J*=9.1, 4.5 Hz), 7.96 (dd, 2H, *J*=9.8, 2.6 Hz), 8.40 (d, 2H, *J*=3.2 Hz), 12.40 (s, 2H); ^{13}C{^{1}H} NMR (DMSO-*d*$_6$, 50 MHz) δ: 106.2 (d, *J*= 24.9 Hz), 111.6 (d, *J*= 26.2 Hz), 112.4 (d, *J*= 4.4 Hz), 113.9 (d, *J*= 10.1 Hz), 126.3 (d, *J*= 11.1 Hz), 133.3, 138.9, 158.9 (d, *J*= 235.6 Hz), 188.0; *Anal.* Calculated for $C_{18}H_{10}F_2N_2O_2$ (MW: 324.28) C, 66.67; H, 3.11; N, 8.64%. Found: C, 66.47; H, 3.31; N, 8.83%.

*Bis(5-methoxy-1H-indol-3-yl)ethane-1,2-dione (**4d**).* Yield: 67% from indole **1d** (R=OCH_3) and acyl chloride **3d** (R^2=OCH_3, R^3=H); gray solid; m.p.: 303.3 °C; IR (cm^{-1}): 3186 (NH), 1608 (CO); ^{1}H NMR (DMSO-*d*$_6$, 200 MHz) δ: 3.84 (s, 6H), 6.92 (dd, 2H, *J*=8.8, 2.5 Hz), 7.44 (d, 2H, *J*=8.8 Hz), 7.80 (d, 2H, *J*=2.5 Hz), 8.18 (s, 2H), 12.15 (s, 2H); ^{13}C{^{1}H} NMR (DMSO-*d*$_6$, 50 MHz) δ: 55.2, 103.0, 112.3, 113.2, 113.3, 126.5, 131.4, 137.3, 155.8, 188.7; *Anal.* Calculated for $C_{20}H_{16}N_2O_4$ (MW: 348.35) C, 68.96; H, 4.63; N, 8.04%. Found: C, 68.74; H, 4.81; N, 8.18%.

Bis(1-methyl-1*H*-indol-3-yl)ethane-1,2-dione *(**4e**).* Yield: 81% from indole **2a** (R=H) and acyl chloride **3e** (R^2=H, R^3=CH_3); yellow solid; m.p.: 267.0 °C; IR (cm^{-1}): 1625 (CO); ^{1}H NMR (DMSO-*d*$_6$, 200 MHz,) δ: 3.89 (s, 6 H),7.31- 7.41 (m, 4H), 7.58-7.64 (m, 2H), 8.29- 8.33 (m, 4 H); ^{13}C{^{1}H} NMR (DMSO-*d*$_6$, 50 MHz) δ: 33.2, 111.0, 111.2, 121.4, 122.8, 123.5, 126.0, 137.4, 140.8, 188.2; *Anal.* Calculated for $C_{20}H_{16}N_2O_2$ (MW: 316.35) C, 75.93; H, 5.10; N, 8.86%. Found: C, 76.04; H, 5.25; N, 8.94%.

*Bis(5-fluoro-1-methyl-1H-indol-3-yl)ethane-1,2-dione (**4f**).* Yield: 72% from indole **2c** (R=F) and acyl chloride **3g** (R^2=F, R^3=CH_3); yellow solid; m.p.: 312.5 °C; IR (cm^{-1}): 1621 (CO); ^{1}H NMR (200 MHz, DMSO-*d*$_6$) δ: 3.90 (s, 6 H), 7.24 (td, 2H, *J* = 9.2, 9.2, 2.6 Hz), 7.66 (dd, 2H, *J* = 9.2, 4.5 Hz), 7.96 (dd, 2H, *J* = 9.7, 2.6 Hz), 8.40 (s, 2H); ^{13}C{^{1}H} NMR (DMSO-*d*$_6$, 50 MHz) δ: 34.1, 106.9 (d, *J*= 24.7 Hz), 111.6 (d, *J*= 3.9 Hz), 112.1 (d, *J*= 26.0 Hz), 113.2 (d, *J*= 10.1 Hz), 127.3 (d, *J*= 11.0 Hz), 134.6, 142.6, 159.8 (d, *J*= 236.3 Hz), 187.9; *Anal.* Calculated for $C_{20}H_{14}F_2N_2O_2$ (MW: 352.33): C, 68.18; H, 4.01; N, 7.95%. Found: C, 68.32; H, 4.25; N, 7.76%.

*Bis(5-methoxy-1-methyl-1H-indol-3-yl)ethane-1,2-dione (**4g**).* Yield: 73% from indole **2d** (R=OCH_3) and acyl chloride **3e** (R^2= OCH_3, R^3=CH_3); yellow solid; m.p.: 244.1 °C; IR (cm^{-1}): 1612 (CO); ^{1}H NMR (DMSO-*d*$_6$, 200 MHz,) δ: 3.85 (s, 6 H), 3.86 (s, 6H), 6.99 (dd, 2H, *J*=8.9, 2.5 Hz), 7.52 (d, 2H, *J*=8.9 Hz), 7.82 (d, 2H, *J*=2.5 Hz), 8.25 (s, 2H); ^{13}C{^{1}H} NMR (DMSO-*d*$_6$, 50 MHz) δ: 33.4, 55.4, 103.1, 111.0, 111.9, 113.1, 127.0, 132.3, 140.7, 156.2, 188.0; *Anal.* Calculated for $C_{22}H_{20}N_2O_4$ (MW: 376.41) C, 70.20; H, 5.36; N, 7.44%. Found: C, 70.35; H, 5.14; N, 7.68%.

*1-(5-Bromo-1H-indol-3-yl)-2-(5-methoxy-1-methyl-1H-indol-3-yl)ethane-1,2-dione (**4h**).* Yield: 66% from indole **2d** (R=OCH_3) and acyl chloride **3b** (R^2= Br, R^3=H); yellow solid; m.p.: 273.8 °C; IR (cm^{-1}): 3210 (NH), 1629 (CO), 1601 (CO); ^{1}H NMR (DMSO-*d*$_6$, 200 MHz) δ: 3.85 (s, 3H), 3.86 (s, 1H), 6.99 (dd, 1H, *J*=8.9, 2.5 Hz), 7.41-7.55 (m, 3H), 7.81 (d, 1H, *J*=2.6 Hz), 8.26 (s, 1H), 8.33 (s, 1H), 8.44 (d, 1H, *J*= 1.6 Hz), 12.45 (s, 1H); ^{13}C{^{1}H} NMR (DMSO-*d*$_6$, 50 MHz) δ: 34.1, 55.3, 103.2, 110.8, 111.9, 112.0, 113.1, 114.7, 115.2, 123.4, 126.0, 127.0, 127.4, 132.3, 135.4, 138.4, 141.0, 156.3, 187.4, 188.5; *Anal.* Calculated for $C_{20}H_{15}BrN_2O_3$ (MW: 411.25) C, 58.41; H, 3.68; N, 6.81%. Found: C, 58.54; H, 3.82; N, 6.94%.

*1-(5-Bromo-1-methyl-1H-indol-3-yl)-2-(5-bromo-1H- indol-3-yl)ethane-1,2-dione (**4i**).* Yield: 73% from indole **2b** (R=Br) and acyl chloride **3b** (R^2= Br, R^3=H); yellow solid; m.p.: 336.6 °C; IR (cm^{-1}): 3317 (NH), 1622 (CO), 1611 (CO); ^{1}H NMR (DMSO-d_6, 200 MHz,) δ: 3.89 (s, 3H), 7.44 (dd, 1H, *J* = 8.6, 1.9 Hz), 7.48-7.55 (m, 2H), 7.62 (d, 1H, *J* = 8.7 Hz), 8.35 (d, 1H, *J* = 3.2 Hz), 8.41-8.44 (m, 3H), 12.49 (s, 1H); ^{13}C{^{1}H} NMR (DMSO-d_6, 50 MHz) δ: 33.5, 110.5, 111.7, 113.3, 114.7, 115.2, 115.8, 123.4, 123.5, 126.0, 126.1, 127.4, 127.8, 135.5, 136.2, 138.7, 142.0, 187.3, 187.8; *Anal.* Calculated for $C_{19}H_{12}Br_2N_2O_2$ (MW: 460.12) C, 49.60; H, 2.63; N, 6.09%. Found: C, 49.74; H, 2.51; N, 6.25%.

*1-(5-Bromo-1H-indol-3-yl)-2-(1-methyl-1H- indol-3-yl)ethane-1,2-dione (**4j**).* Yield: 75% from indole **2a** (R=H) and acyl chloride **3b** (R^2= Br, R^3=H); yellow solid; m.p.: 289.8 °C; IR (cm^{-1}): 3318 (NH), 1624 (CO), 1608 (CO); ^{1}H NMR (DMSO-d_6, 200 MHz) δ: 3.88 (s, 3H), 7.31-7.38 (m, 2H), 7.43 (dd, 1H, *J*= 8.6, 2.0 Hz), 7.5 (d, 1H, *J* = 8.6 Hz), 7.60 (dd, 1H, *J* = 6.3, 2.4 Hz), 8.28- 8.33 (m, 3H) 8.43 (d, 1H, *J* = 2.0 Hz) 12.42 (s, 1H); ^{13}C{^{1}H} NMR (DMSO-d_6, 50 MHz) δ: 34.0, 106.7, 107.1, 112.2, 112.8, 113.1, 121.8, 122.9, 124.0, 126.1, 127.2, 134.6, 137.2, 138.0, 142.4, 158.2, 161.3, 188.4, 188.7; *Anal.* Calculated for $C_{19}H_{13}BrN_2O_2$ (MW: 381.22) C, 59.86; H, 3.44; N, 7.35%. Found: C, 59.74; H, 3.61; N, 7.15%.

*1-(5-Bromo-1H-indol-3-yl)-2-(5-fluoro-1-methyl-1H- indol-3-yl)ethane-1,2-dione (**4k**).* Yield: 60% from indole **2c** (R=F) and acyl chloride **3b** (R^2= Br, R^3=H); yellow solid; m.p.: 225.0 °C; IR (cm^{-1}): 3319 (NH), 1636 (CO), 1610 (CO); ^{1}H NMR (DMSO-d_6, 200 MHz,) δ: 3.89 (s, 3H), 7.22 (td, 1H, *J* = 9.2, 9.2, 2.6 Hz), 7.43 (dd, 1H, *J* = 8.6, 2.0 Hz), 7.52 (d, 1H, *J* = 8.6 Hz), 7.64 (dd, 1H, *J* = 9.0, 4.4 Hz), 7.95 (dd, 1H, *J* = 9.6, 2.6 Hz), 8.33 (s, 1H), 8.42 (m, 2H), 12.44 (s, 1H); ^{13}C{^{1}H} NMR (DMSO-d_6, 50 MHz) δ: 34.1, 106.9 (d, *J* = 24.9 Hz), 111.6 (d, *J*= 4.0 Hz), 112.1 (d, *J* = 26.0 Hz), 112.3, 113.2 (d, *J*= 9.9 Hz), 115.2, 115.7, 123.9, 126.6, 127.3 (d, *J*= 11.0 Hz), 127.9, 134.6, 136.0, 139.0, 142.7, 159.8 (d, *J*=236.6 Hz), 187.6, 188.5; *Anal.* Calculated for $C_{19}H_{12}BrFN_2O_2$ (MW: 399.21) C, 57.16; H, 3.03; N, 7.02%. Found: C, 57.33; H, 3.30; N, 6.85%.

*1-(5-Bromo-1-methyl-1H-indol-3-yl)-2-(1H-indol-3-yl)ethane-1,2-dione (**4l**). Yield*: 69% from indole **2b** (R=Br) and acyl chloride **3a** (R^2= H, R^3=H); yellow solid; m.p.: 262.0 °C; IR (cm^{-1}): 3204 (NH), 1629 (CO), 1607 (CO); ^{1}H NMR (DMSO-d_6, 200 MHz) δ: 3.89 (s, 3H), 7.25-7.34 (m, 2H), 7.48-7.64 (m, 3H), 8.26-8.30 (m, 2H), 8.38 (s, 1H), 8.42 (d, 1H), 12.31 (s, 1H); ^{13}C{^{1}H} NMR (DMSO-d_6, 50 MHz) δ: 33.4, 110.7, 112.2, 112.3, 113.3, 115.7, 121.2, 122.5, 123.5, 125.6, 126.0, 126.1, 127.8, 136.2, 137.7, 136.7, 141.7, 188.0, 188.1; *Anal.* Calculated for $C_{19}H_{13}BrN_2O_2$ (MW: 381.22): C, 59.86; H, 3.44; N, 7.35%. Found: C, 59.98; H, 3.57; N, 7.11%.

*1-(1H-Indol-3-yl)-2-(1-methyl-1H-indol-3-yl)ethane-1,2-dione (**4m**).* Yield: 77% from indole **1a** (R=H) and acyl chloride **3e** (R^2= H, R^3=CH_3); yellow solid; m.p.: 226.9 °C; IR (cm^{-1}): 3410 (NH), 1624 (CO), 1607 (CO); ^{1}H NMR (DMSO-d_6, 200 MHz) δ: 3.89 (s, 3H), 7.27-7.41 (m, 4H), 7.53-7.64 (m, 2H), 8.23-8.32 (m, 4H), 12.28 (s, 1H); ^{13}C{^{1}H} NMR (DMSO-d_6, 50 MHz) δ: 33.3, 99.5, 111.0, 111.3, 112.6, 121.2, 121.4, 122.4, 122.5, 122.8, 123.5, 125.5, 126.0, 137.0, 137.3, 137.4, 140.7, 188.2, 188.7; *Anal.* Calculated for $C_{19}H_{14}N_2O_2$ (MW: 302.33) C, 75.48; H, 4.67; N, 9.27%. Found: C, 75.33; H, 4.76; N, 9.12%.

*1-(1H-Indol-3-yl)-2-(5-methoxy-1-methyl-1H-indol-3-yl)ethane-1,2-dione (**4n**).* Yield: 72% from indole **2d** (R=OCH_3) and acyl chloride **3a** (R^2= H, R^3=H); yellow solid; m.p.: 243.8 °C; IR (cm^{-1}): 3182 (NH), 1622 (CO), 1600 (CO); ^{1}H NMR (DMSO-d_6, 200 MHz,) δ: 3.85 (m, 6H), 6.98 (dd, 1H, *J* = 8.9, 2.4 Hz), 7.28 – 7.31 (m, 2H), 7.49 - 7.56 (m, 2H), 7.82 (d, 1H, *J* = 2.4 Hz), 8.22 – 8.30 (m, 3H), 12.22 (s, 1H); ^{13}C{^{1}H} NMR (DMSO-d_6, 50 MHz) δ: 33.8, 55.8, 103.8, 111.5, 112.4, 112.9, 113.0, 113.5, 121.8, 122.9, 123.9, 126.1, 127.5, 132.8, 137.2, 137.8, 141.1, 156.7, 188.6, 189.2; *Anal.* Calculated for $C_{20}H_{16}N_2O_2$ (MW: 332.35) C, 72.28; H, 4.85; N, 8.43%. Found: C, 72.48; H, 4.73; N, 8.12%.

*1-(5-Fluoro-1-methyl-1H-indol-3-yl)-2-(1H-indol-3-yl)-ethane-1,2-dione (**4o**).* Yield: 79% from indole **1a** (R=H) and acyl chloride **3f** (R^2= F, R^3=CH_3); yellow solid; m.p.: 235.2 °C; IR (cm^{-1}): 3219 (NH), 1622 (CO), 1604 (CO); ^{1}H NMR (DMSO-d_6, 200 MHz) δ: 3.89 (s, 3H), 7.3- 7.39 (m, 2H), 7.44 (dd, 1H, *J* = 8.6, 1.9 Hz), 7.53 (d, 1H, *J* = 8.6 Hz), 7.59-7.62 (m, 1H), 8.28 – 8.33 (m, 3H), 8.43- 8.44 (m, 1H), 12.43 (1H, s); ^{13}C{^{1}H} NMR (DMSO-d_6, 50 MHz) δ: 34.0, 106.9 (d, *J* = 23.9 Hz), 111.7 (d, *J* = 3.9 Hz), 112.0 (d, *J* = 26.0 Hz), 112.8, 113.0 (d, *J* = 10.1 Hz), 113.1, 121.8, 122.9, 124.0, 126.1, 127.3 (d, *J* = 11.3 Hz), 134.6, 137.2, 138.0, 142.4, 159.7 (d, *J*= 236.3 Hz), 188.4, 188.8; *Anal.* Calculated for $C_{19}H_{13}FN_2O_2$ (MW: 320.32) C, 71.24; H, 4.09; N, 8.75%. Found: C, 71.38; H, 4.23; N, 8.52%.

*1-(5-Methoxy-1H-indol-3-yl)-2-(1-methyl-1H-indol-3-yl)ethane-1,2-dione (**4p**).* Yield: 80% from indole **2a** (R=H) and acyl chloride **3d** (R^2= OCH_3, R^3=H); yellow solid; m.p.: 245.8 °C; IR (cm^{-1}): 3153 (NH), 1618 (CO), 1602 (CO); ^{1}H NMR (DMSO-*d*$_6$, 200 MHz) δ: 3.83 (s, 3H), 3.89 (s, 3 H), 6.92 (dd, 1H, *J* = 8.8, 2.5 Hz), 7.28 – 7.40 (m, 2H), 7.44 (d, 1H, *J* = 8.8 Hz), 7.59- 7.62 (m, 1H), 7.80 (d, 1H, *J* = 2.5 Hz), 8.16 (d, 1H, *J* = 3.3 Hz), 8.29 -8.31 (m, 2H), 12.14 (s, 1H); ^{13}C{^{1}H} NMR (DMSO-*d*$_6$, 50 MHz) δ: 33.7, 55.8, 103.6, 111.5, 111.8, 112.8, 113.7, 113.8, 121.9, 123.3, 123.9, 126.6, 127.0, 132.0, 137.8, 137.9, 141.2, 156.4, 188.7, 189.0; *Anal.* Calculated for $C_{20}H_{16}N_2O_2$ (MW: 332.35) C, 72.28; H, 4.85; N, 8.43%. Found: C, 72.48; H, 4.99; N, 8.62%.

*1-(5-Methoxy-1H-indol-3-yl)-2-(5-methoxy-1-methyl-1H-indol-3-yl)ethane-1,2-dione (**4q**).* Yield: 69% from indole **2d** (R=OCH_3) and acyl chloride **3d** (R^2= OCH_3, R^3=H); yellow solid; m.p.: 302.6 °C; IR (cm^{-1}): 3238 (NH), 1608 (CO b.s.); ^{1}H NMR (DMSO-*d*$_6$, 200 MHz) δ: 3.83 (s, 9H), 6.91 (dd, 1H, *J* = 8.8, 2.5 Hz), 6.97 (dd, 1H, *J* = 8.9, 2.5 Hz), 7.43 (d, 1H, *J* = 8.8 Hz), 7.49 (d, 1H, *J* = 8.9 Hz), 7.79-7.81 (m, 2H), 8.16 (s, 1H), 8.23 (s, 1H), 12.13 (s, 1H). ^{13}C{^{1}H} NMR (DMSO-*d*$_6$, 50 MHz) δ: 33.8, 55.8, 55.9, 103.6, 103.8, 111.6, 112.4, 112.8, 113.5, 113.7, 113.8, 127.0, 127.5, 131.9, 132.8, 137.9, 141.1, 156.3, 156.7, 188.6, 189.1; *Anal.* Calculated for $C_{21}H_{18}N_2O_2$ (MW: 362.38) C, 69.60; H, 5.01; N, 7.73%. Found: C, 69.48; H, 4.83; N, 7.52%.

*1-(5-Fluoro-1-methyl-1H-indol-3-yl)-2-(5-methoxy-1-methyl-1H-indol-3-yl)ethane-1,2-dione (**4r**).* Yield: 76% from indole **2c** (R=F) and acyl chloride **3d** (R^2= OCH_3, R^3=H); yellow solid; m.p.: 308.2 °C; IR (cm^{-1}): 3196 (NH), 1620 (CO), 1600 (CO); ^{1}H NMR (DMSO-*d*$_6$, 200 MHz) δ: 3.84 (s, 3H), 3.90 (s, 3H), 6.93 (dd, 1H, *J* = 8.8, 2.5 Hz), 7.22 (td, 1H, *J* = 9.2, 9.1, 2.6 Hz), 7.45 (d, 1H, *J* = 8.8 Hz), 7.64 (dd, 1H, *J* = 8.9, 4.4 Hz), 7.81 (d, 1H, *J* = 2.5 Hz), 8.04 – 7.91 (m, 1H), 8.20 (d, 1H, *J* = 3.3 Hz), 8.40 (s, 1H), 12.17 (s, 1H); ^{13}C{^{1}H} NMR (DMSO-*d*$_6$, 50 MHz) δ: 34.0, 55.8, 103.6, 106.9 (d, *J* = 23.9 Hz), 111.8 (d, *J* = 4.2 Hz), 112.5 (d, *J* = 26.2 Hz), 112.7, 113.0 (d, *J*= 10.1 Hz), 113.8, 127.0, 127.3 (d, *J*= 11.3 Hz), 132.0, 134.6, 137.9, 138.0, 142.4, 156.4, 159.8 (d, *J* = 236.3 Hz), 188.4, 188.6. *Anal.* Calculated for $C_{20}H_{15}FN_2O_3$ (MW: 350.34) C, 68.57; H, 4.32; N, 8.00%. Found: C, 68.38; H, 4.53; N, 7.72%.

*1-(5-Bromo-1-methyl-1H-indol-3-yl)-2-(5-methoxy-1H-indol-3-yl)ethane-1,2-dione (**4s**).* Yield: 78% from indole **2b** (R=Br) and acyl chloride **3d** (R^2= OCH_3, R^3=H); yellow solid; m.p.: 320.7 °C; IR (cm^{-1}): 3184 (NH), 1627 (CO), 1600 (CO); ^{1}H NMR (DMSO-*d*$_6$, 200 MHz) δ: 3.84 (s, 3H), 3.89 (s, 3H), 6.93 (dd, 1H, *J* = 8.8, 2.4 Hz), 7.44 (d, 1H, *J* = 8.8), 7.50 (dd, 1H, *J* = 8.7, 1.8 Hz), 7.61 (d, 1H, *J* = 8.7 Hz), 7.80 (d, 1H, *J* = 2.4 Hz), 8.20 (d, 1H, *J* = 3.3 Hz), 8.39 (s, 1H), 8.44 (d, 1H, *J* = 1.8 Hz), 12.18 (s, 1H); ^{13}C{^{1}H} NMR (DMSO-*d*$_6$, 50 MHz) δ: 33.9, 55.8, 103.6, 103.7, 111.3, 112.6, 113.7, 113.8, 116.2, 124.1, 126.5, 127.0, 128.3, 132.0, 136.7, 138.1, 142.2, 156.4, 188.4, 188.5; *Anal.* Calculated for $C_{20}H_{15}BrN_2O_3$ (MW: 411.25) C, 58.41; H, 3.68; N, 6.81%. Found: C, 58.68; H, 3.53; N, 6.75%.

*1-(5-Bromo-1-methyl-1H-indol-3-yl)-2-(5-fluoro-1H-indol-3-yl)ethane-1,2-dione (**4t**).* Yield: 77% from indole **2b** (R=Br) and acyl chloride **3c** (R^2= F, R^3=H); orange solid; m.p.: 294.3 °C; IR (cm^{-1}): 3183 (NH), 1640 (CO), 1606 (CO); ^{1}H NMR (DMSO-*d*$_6$, 200 MHz,) δ: 3.89 (s, 3H), 7.16 (td, 1H, *J* = 9.4, 9.1, 2.6 Hz), 7.48-7.64 (m, 3H), 7.95 (dd, 1H, *J* = 9.7, 2.6 Hz), 8.35-8.44 (m, 3H), 12.34 (s, 1H); ^{13}C{^{1}H} NMR (DMSO-*d*$_6$, 50 MHz) δ: 33.5, 106.2 (d, *J*= 24.5 Hz), 110.6, 111.6 (d, *J*= 26.2 Hz), 112.3 (s, *J*= 4.6 Hz), 113.3, 113.9 (d, *J*= 10.4 Hz), 115.7, 123.5, 126.0, 126.3 (d, *J*= 11.1 Hz), 127.8, 133.3, 136.2, 139.0, 141.8, 158.9 (d, *J*= 235.7 Hz), 187.5, 187.8; *Anal.* Calculated for $C_{19}H_{12}BrFN_2O_2$ (MW: 399.21): C, 57.16; H, 3.03; N, 7.02%. Found: C, 57.26; H, 3.12; N, 7.33%.

*1-(5-Fluoro-1H-indol-3-yl)-2-(5-methoxy-1-methy-1H-indol-3-yl)ethane-1,2-dione (**4u**).* Yield: 78% from indole **2d** (R=OCH_3) and acyl chloride **3c** (R^2= F, R^3=H); yellow solid; m.p.: 226.9 °C; IR (cm^{-1}): 3427 (NH), 1646 (CO), 1612 (CO); ^{1}H NMR (DMSO-*d*$_6$, 200 MHz,) δ: 3.85 (s, 3H), 3.86 (s, 3H), 6.99 (dd, 1H, *J* = 8.9, 2.5 Hz), 7.16 (td, 1H, *J* = 9.0, 9.0, 2.3 Hz), 7.50-7.60 (m, 2H), 7.81 (d, 1H, *J* = 2.5 Hz), 7.95 (dd, 1H, *J* = 9.8, 2.3 Hz), 8.25 (s, 1H), 8.33 (d, 1H, *J* = 3.1 Hz), 12.35 (s, 1H); ^{13}C{^{1}H} NMR (DMSO-*d*$_6$, 50 MHz) δ: 33.9, 55.8, 103.8, 106.7 (d, *J*= 24.5 Hz), 111.4, 112.0 (d, *J*= 25.6 Hz), 112.4, 113.0 (d, *J*= 4.2 Hz), 113.6, 114.4 (d, *J*= 9.9 Hz), 126.8 (d, *J*= 11.2 Hz), 127.5, 132.8, 133.8, 139.2, 141.3, 156.8, 159.4 (s, *J*= 235.5 Hz), 188.1, 189.0; *Anal.* Calculated for $C_{20}H_{15}FN_2O_3$ (MW: 350.34): C, 68.57; H, 4.32; N, 8.00%. Found: C, 68.51; H, 4.47; N, 7.84%

*1-(5-Fluoro-1H-indol-3-yl)-2-(1-methy-1H-indol-3-yl)ethane-1,2-dione (**4v**).* Yield: 72% from indole **2a** (R=H) and acyl chloride **3c** (R^2= F, R^3=H); yellow solid; m.p.: 260.9 °C; IR (cm^{-1}): 3321 (NH), 1635 (CO), 1614 (CO); ^{1}H NMR (DMSO-*d*$_6$, 200 MHz) δ: 3.89 (s, 3H) , 7.16 (td, 1H, *J* = 9.2, 9.2, 2.3 Hz), 7.25-7.44 (m, 2H), 7.49-7.68 (m, 2H), 7.96 (dd, 1H, *J* = 9.7, 2.3 Hz), 8.29-8.32 (m, 3H), 12.37 (s, 1H); ^{13}C{^{1}H} NMR (DMSO-*d*$_6$, 50 MHz) δ: 33.7, 106.7 (d, *J*= 24.1 Hz), 111.5, 111.8 (d, *J*= 4.3 Hz), 112.0 (d, *J*= 26.0 Hz), 114.4 (d, *J*= 9.2 Hz), 121.9, 123.3, 124.0, 126.6, 126.8

(d, *J*= 11.5 Hz), 130.2, 133.8, 137.9, 139.2, 141.4, 159.4 (d, *J*= 235.3 Hz), 188.3, 188.9. *Anal.* Calculated for $C_{19}H_{13}FN_2O_3$ (MW: 320.32): C, 71.24; H, 4.09; N, 8.75%. Found: C, 69.96; H, 4.27; N, 8.87%.

*1-(5-Fluoro-1H-indol-3-yl)-2-(5-fluoro-1-methy-1H-indol-3-yl)ethane-1,2-dione (**4w**).* Yield: 68% from indole **2c** (R=F) and acyl chloride **3c** (R^2= F, R^3=H); yellow solid; m.p.: 303.2 °C; IR (cm^{-1}): 3352 (NH), 1627 (CO), 1616 (CO); ^{1}H NMR (DMSO-d_6, 200 MHz) δ: 3.90 (s, 3H), 7.13-7.26 (m, 2H), 7.57 (dd, 1H, *J* = 8.8, 4.4 Hz), 7.65 (dd, 1H, *J* = 8.9, 4.4 Hz), 7.93-7.99 (m, 2H), 8.34 (dd, 1H, *J* = 3.3 Hz), 8.40 (s, 1H), 12.39 (s, 1H); ^{13}C{^{1}H} NMR (DMSO-d_6, 50 MHz) δ: 34.0, 106.7 (d, *J*= 24.8 Hz), 106.9 (d, *J*= 24.4 Hz), 111.6 (d, *J*= 4.4 Hz), 111.9 (d, *J*= 26.2 Hz), 112.2 (d, *J*= 25.8 Hz), 112.9 (d, *J*= 4.3 Hz), 113.0 (d, *J*= 9.9 Hz), 114.4 (d, *J*= 9.8 Hz), 126.8 (d, *J*= 11.1 Hz), 127.3 (d, *J*= 11.1 Hz), 133.8, 134.6, 139.3, 142.5, 158.1 (d, *J*= 235.5 Hz), 161.2 (d, *J*= 236.22 Hz), 188.0, 188.4. *Anal.* Calculated for $C_{19}H_{12}F_2N_2O_2$ (MW: 338.31): C, 67.45; H, 3.58; N, 8.28%. Found: C, 67.71; H, 3.27; N, 8.44%.

*1-(5-Methoxy-1-methy-1H-indol-3-yl)-2-(1-methyl-1H-indol-3-yl)ethane-1,2-dione (**4x**).* Yield: 81% from indole **2d** (R=OCH_3) and acyl chloride **3e** (R^2= H, R^3=CH_3); orange solid; m.p.: 196.3 °C; IR (cm^{-1}): 1613 (CO), 1520 (CO); ^{1}H NMR (DMSO-d_6, 200 MHz,) δ: 3.84 (s, 6H), 3.85 (s, 3H), 3.89 (s, 3H),6.99 (dd, 1H, *J* = 8.9, 2.5 Hz), 7.33-7.41 (m, 2H), 7.52 (d, 1H, *J* = 8.9 Hz), 7.59-7.63 (m, 1H), 7.82 (d, 1H, *J*= 2.5 Hz), 8.23 (s, 1H), 8.28-8.32 (m, 2H); ^{13}C{^{1}H} NMR (DMSO-d_6, 50 MHz) δ: 33.2, 33.4, 55.3, 103.2, 110.9, 111.0, 111.2, 111.9, 113.1, 121.4, 122.8, 123.5, 126.0, 127.0, 132.3, 137.4, 140.7, 140.8, 156.2, 188.0, 188.2; *Anal.* Calculated for $C_{21}H_{18}N_2O_3$ (MW: 346.38) C, 72.82; H, 5.24; N, 8.09%. Found: C, 72.96; H, 5.04; N, 7.98%.

*1-(5-Fluoro-1-methy-1H-indol-3-yl)-2-(5-methoxy-1-methyl-1H-indol-3-yl)ethane-1,2-dione (**4y**).* Yield: 84% from indole **2c** (R=F) and acyl chloride **3h** (R^2= OCH_3, R^3=CH_3); orange solid; m.p.: 202.4 °C; IR (cm^{-1}): 1622 (CO), 1516 (CO);^{1}H NMR (DMSO-d_6, 200 MHz,) δ: 3.83 (s, 3H), 3.85 (s, 3H), 3.89 (s, 3H), 6.98 (dd, 1H, *J*= 8.9, 2.5 Hz), 7.22 (td, 1H, *J* = 9.6, 9.2, 2.6 Hz), 7.50 (d, 1H, *J* = 8.9 Hz), 7.64 (dd, 1H, *J* = 9.0, 4.4 Hz), 7.81 (d, 1H, *J*= 2.5 Hz), 7.96 (dd, 1H, *J* = 9.6, 2.6 Hz), 8.24 (s, 1H), 8.39 (s, 1H); ^{13}C{^{1}H} NMR (DMSO-d_6, 50 MHz) δ: 33.9, 34.0, 55.8, 103.7, 106.9 (d, *J*= 24.8 Hz), 111.4, 111.7 (d, *J* = 4.1 Hz), 112.0 (d, *J* = 25.6 Hz), 112.4, 113.1 (d, *J* = 9.6 Hz), 113.6, 127.3 (d, *J* = 11.0 Hz), 127.5, 132.8, 134.6, 141.3, 142.5, 156.8, 159.8 (d, *J* = 235.3 Hz), 188.0, 188.4; *Anal.* Calculated for $C_{21}H_{17}FN_2O_3$ (MW: 364.37) C, 69.22; H, 4.70; N, 7.69%. Found: C, 69.50; H, 4.54; N, 7.84%.

*1-(5-Bromo-1-methy-1H-indol-3-yl)-2-(5-methoxy-1-methyl-1H-indol-3-yl)ethane-1,2-dione (**4z**).* Yield: 85% from indole **2b** (R=Br) and acyl chloride **3h** (R^2= OCH_3, R^3=CH_3); orange solid; m.p.: 203.1 °C; IR (cm^{-1}): 1647 (CO), 1624 (CO); ^{1}H NMR (DMSO-d_6, 200 MHz,) δ: 3.83 (s, 3H), 3.85 (s, 3H), 3.88 (s, 3H), 6.98 (dd, 1H, *J*= 8.9, 2.5 Hz), 7.49-7.52 (m, 2H), 7.61 (d, 1H, *J* = 9.1 Hz), 7.80 (d, 1H, *J* = 2.5 Hz), 8.25 (s, 1H), 8.38 (s, 1H) 8.43 (d, 1H, *J* = 1.9 Hz); ^{13}C{^{1}H} NMR (DMSO-d_6, 50 MHz) δ: 33.9 (x 2), 55.9, 103.8, 111.2, 111.3, 112.5, 113.6, 113.8, 116.2, 124.0, 126.5, 127.5, 128.3, 132.9, 136.7, 141.4, 142.2, 156.8, 187.8, 188.4; *Anal.* Calculated for $C_{21}H_{17}BrN_2O_3$ (MW: 425.28) C, 59.31; H, 4.03; N, 6.59%. Found: C, 59.50; H, 4.19; N, 6.78%.

*1-(5-Fluoro-1-methyl-1H-indol-3-yl)-2-(1-methyl-1H-indol-3-yl)ethane-1,2-dione (**4aa**).* Yield: 81% from indole **2c** (R=F) and acyl chloride **3e** (R^2= H, R^3=CH_3); yellow solid; m.p.: 273.5 °C; IR (cm^{-1}): 1616 (CO), 1607 (CO); ^{1}H NMR (DMSO-d_6, 200 MHz) δ: 3.95 (s, 6H), 7.24- 7.46 (m, 3H), 7.65- 7.75 (m, 2H), 8.02 (dd, 1H, *J* = 9.7, 2.6 Hz), 8.33- 8.44 (m, 3H); ^{13}C{^{1}H} NMR (DMSO-d_6, 50 MHz) δ: 33.7, 34.0, 106.9 (d, *J* = 24.8 Hz), 111.6, 111.7, 111.8 (d, *J* = 4.1 Hz), 112.0 (d, *J*= 26.0 Hz), 113.1 (d, *J* = 9.6 Hz), 121.9, 123.3, 124.0, 126.6, 127.3 (d, *J* = 11.0 Hz), 134.6, 138.0, 141.4, 142.4, 159.8 (d, *J* = 236.0.3 Hz), 188.2, 188.4; *Anal.* Calculated for $C_{20}H_{15}FN_2O_2$ (MW: 334.34) C, 71.85; H, 4.52; N, 8.38%. Found: C, 71.64; H, 4.67; N, 8.14%.

*1-(5-Bromo-1-methyl-1H-indol-3-yl)-2-(1-methyl-1H-indol-3-yl)ethane-1,2-dione (**4ab**).* Yield: 69% from indole **2a** (R=H) and acyl chloride **3f** (R^2= Br, R^3=CH_3); yellow solid; m.p.: 274.5 °C; IR (cm^{-1}): 1628 (CO), 1610 (CO); ^{1}H NMR (DMSO-d_6, 200 MHz) δ: 3.89 (s, 6H),7.31-7.41 (m, 2H), 7.51 (dd, 1H, *J* = 8.7, 1.9 Hz), 7.60-7.65 (m, 2H), 8.28-8.34 (m, 2H), 8.38 (s, 1H), 8.45 (d, 1H, *J* = 1.9 Hz); ^{13}C{^{1}H} NMR (DMSO-d_6, 50 MHz) δ: 33.3, 33.5, 99.7, 110.6, 111.0, 111.1, 113.3, 115.7, 121.4, 122.9, 123.3, 123.6, 126.0, 126.0, 127.7, 136.2, 137.4, 141.1, 187.5, 187.9; *Anal.* Calculated for $C_{20}H_{15}BrN_2O_2$ (MW: 395.25): C, 60.78; H, 3.83; N, 7.09%. Found: C, 60.89; H, 3.69; N, 7.23%.

*5,6-Di(1H-indol-3-yl)-1,2,4-triazin-3-amine (**5a**).* Yield: 55%; yellow solid; m.p.: 288.2 °C; IR (cm^{-1}): 3453/3400 (NH_2), 3285 (NH), 3184 (NH); ^{1}H NMR (DMSO-d_6, 200 MHz) δ: 6.86-6.95 (m, 4H), 7.08-7.22 (m, 4H), 7.39-7.34 (m, 1H), 7.51-7.47 (m, 1H), 7.60 (d, 1H, *J* = 2.5 Hz), 8.65 (m, 1H), 11.41-11.47 (m, 2H); ^{13}C{1H} NMR (DMSO-d_6, 50 MHz) δ: 110.4, 111.8. 111.9, 112.8, 119.3, 119.6, 120.7, 121.4, 122.4, 123.1, 125.2, 125.7, 126.4, 130.6, 136.1, 136.1, 143.1, 153.7, 160.8. LC-MS: *m/z* [M + H]$^+$ calcd for $C_{19}H_{15}N_6^+$ 327.13; found, 327.35. *Anal.* Calculated for $C_{19}H_{14}N_6$ (MW: 326.35) C, 69.92; H, 4.32; N, 25.75%. Found: C, 69.72; H, 4.43; N, 25.60%.

*5,6-Bis(5-methoxy-1H-indol-3-yl)-1,2,4-triazin-3-amine (**5b**).* Yield: 78%; yellow solid; m.p.: 197.2 °C; IR (cm^{-1}): 3383/3298 (NH_2), 3219 (NH), 3145 (NH); ^{1}H NMR (DMSO-d_6, 200 MHz) δ: 3.46 (s, 3H), 3.73 (s, 3H), 6.60 (d, 1H, *J*=2.2 Hz), 6.74 (dd, 1H, *J*=8.6, 2.4 Hz), 6.81 (d, 1H, *J*=2.6 Hz,), 6.90 (s, 2H), 6.97 (m, 1H), 7.25 (d, 1H, *J*=8.8 Hz), 7.35 (d, 1H, *J*=8.8 Hz,), 7.50 (d, 1H, *J*=2.6 Hz), 7.91 (d, 1H, *J*=2.3 Hz), 11.27 (d, 1H, *J*=1.7 Hz), 11.31 (d, 1H, *J*=1.5 Hz). ^{13}C{1H} NMR (DMSO-d_6, 50 MHz) δ: 55.5, 55.8, 101.6, 105.2, 110.9, 112.2, 112.7, 112.9 (x 2), 113.0, 126.3, 126.6, 127.4, 131.5, 131.6, 131.7, 143.9, 154.0, 154.4, 155.1, 160.8. LC-MS: *m/z* [M + H]$^+$ calcd for $C_{21}H_{19}N_6O_2^+$ 387.15; found, 387.39. *Anal.* Calculated for $C_{21}H_{18}N_6O_2$ (MW: 386.41) C, 65.27; H, 4.70; N, 21.75%. Found: C, 65.46; H, 4.59; N, 21.54%.

*5,6-Bis(5-fluoro-1H-indol-3-yl)-1,2,4-triazin-3-amine (**5c**).* Yield: 61%; yellow solid; m.p.: 178.1°C; IR (cm^{-1}): 3420/3414 (NH_2), 3169 (2 x NH b.s.); ^{1}H NMR (DMSO-d_6, 200 MHz,) δ: 6.86 (dd, 1H, *J* =9.9, 2.4 Hz,), 7.04-7.09 (m, 5H), 7.40 (dd, 1H, *J* = 4.7 Hz,), 7.19 (dd, 1H, *J* = 8.8, 4.6 Hz), 7.70 (d, 1H, *J* =2.4 Hz), 8.37 (dd, 1H, *J* =10.7, 2.6 Hz), 11.53 (s, 1H), 11.60 (s, 1H); ^{13}C{1H} NMR (DMSO-d_6, 50 MHz) δ: 104.6 (d, *J*=24.4 Hz), 108.3, 108.4 (d, *J*=26.7 Hz), 110.2 (d, *J*=26.2 Hz), 110.9 (d, *J*=4.6 Hz), 111.1 (d, *J*=22.2 Hz), 113.3 (d, *J*=4.6 Hz), 113.4 (d, *J*=9.8 Hz), , 113.5 (d, *J*=9.3 Hz), 126.5 (d, *J*=10.2 Hz), 127.4 (d, *J*=11.3 Hz), 127.8, 133.2, 133.3 143.1, 153.6, 157.5 (d, *J*=232.4 Hz), 158.5 (d, *J*=232.8 Hz), 161.4. LC-MS: *m/z* [M + H]$^+$ calcd for $C_{19}H_{13}F_2N_6^+$ 363.11; found, 363.35. *Anal.* Calculated for $C_{19}H_{12}F_2N_6$ (MW: 362.34) C, 62.98; H, 3.34; N, 23.19%. Found: C, 63.28; H, 3.23; N, 23.05%.

*5,6-Bis(5-bromo-1H-indol-3-yl)-1,2,4-triazin-3-amine (**5d**).* Yield: 70%; yellow solid; m.p.: 208.2 °C; IR (cm^{-1}): 3447/3420 (NH_2), 3318 (NH), 3275(NH); ^{1}H NMR (200 MHz, DMSO-d_6) δ: 7.02 (d, 1H, *J*=1.8 Hz,), 7.07 (s, 2H), 7.22-7.32 (m, 2H), 7.36-7.38 (m, 2H), 7.47 (d, 1H, *J*=8.6 Hz), 7.62 (d, 1H, *J*=1.6 Hz), 8.65 (s, 1H), 11.61 (s, 1H), 11.65 (s, 1H). ^{13}C{1H} NMR (DMSO-d_6, 50 MHz): δ: 105.3, 110.6, 112.5, 112.6, 114.2, 114.4, 122.2, 124.5, 125.2, 125.5, 127.4, 128.0, 128.4, 131.8, 135.3, 135.4, 143.1, 153.4, 161.4. LC-MS: *m/z* [M + H]$^+$ calcd for $C_{19}H_{13}Br_2N_6^+$ 482.95; found, 483.21. *Anal.* Calculated for $C_{19}H_{12}Br_2N_6$ (MW: 484.15) C, 47.14; H, 2.50; N, 17.36%. Found: C, 47.28; H, 2.70; N, 17.10%.

*5,6-Bis(1-methyl-1H-indol-3-yl)-1,2,4-triazin-3-amine (**5e**).* Yield: 85%; yellow solid; m.p.: 274.4 °C; IR (cm^{-1}): 3458/3265 (NH_2); ^{1}H NMR (DMSO-d_6, 200 MHz) δ: 3.51 (s, 3H), 3.88 (s, 3H), 6.90-6.97 (m, 3H), 7.07 (s, 1H), 7.15-7.29 (m, 4H), 7.42-7.56 (m, 2H), 7.62 (s, 1H), 8.56-8.60 (m, 1H); ^{13}C{1H} NMR (DMSO-d_6, 50 MHz) δ: 32.6, 32.9, 109.5, 110.1, 110.2, 111.6, 119.3, 120.0, 120.9, 121.4, 122.4, 123.1, 125.9, 126.8, 129.5, 134.1, 136.6, 136.8, 143.0, 152.8, 160.8. LC-MS: *m/z* [M + H]$^+$ calcd for $C_{21}H_{19}N_6^+$ 355.16; found, 355.36. *Anal.* Calculated for $C_{21}H_{18}N_6$ (MW: 354.41) C, 71.71; H, 5.12; N, 23.71%. Found: C, 71.47; H, 5.23; N, 23.58%.

*5,6-Bis(5-methoxy-1-methyl-1H-indol-3-yl)-1,2,4-triazin-3-amine (**5f**).* Yield: 98%; yellow solid; m.p.: 199.5 °C; IR (cm^{-1}): 3458/3273 (NH_2); ^{1}H NMR (DMSO-d_6, 200 MHz) δ: 3.43 (s, 3H), 3.55 (s, 3H), 3.72 (s, 3H), 3.82 (s, 3H), 6.71 (d, 1H, *J* = 2.2 Hz), 6.79-6.90 (m, 2H), 7.23-7.26 (m, 3H), 7.37 (d, 1H, *J* = 8.9 Hz), 7.43 (d, 1H, *J* = 8.9 Hz) 7.57 (s, 1H), 7.86 (d, 1H, *J* =2.4 Hz). ^{13}C{1H} NMR (DMSO-d_6, 50 MHz) δ: 32.8, 33.2, 55.0, 55.3, 100.1, 101.6, 104.8, 109.4, 110.5. 110.0, 111.1, 111.2, 111.7, 111.8, 112.3, 126.3, 127.3, 131.9, 132.0, 143.1, 153.7, 154.1, 155.1. *Anal.* Calculated for $C_{23}H_{22}N_6O_2$ (MW: 414.46) C, 66.65; H, 5.35; N, 20.28%. Found: C, 66.76; H, 5.17; N, 20.11%.

*5,6-Bis(5-fluoro-1-methyl-1H-indol-3-yl)-1,2,4-triazin-3-amine (**5g**).* Yield: 77%; yellow solid; m.p.: 236.5 °C; IR (cm^{-1}): 3485/3275 (NH_2); ^{1}H NMR (DMSO-d_6, 200 MHz) δ: 3.56 (s, 3H), 3.88 (s, 3H), 6.93 (dd, 1H, *J*=10.0, 2.5 Hz Hz,), 6.99-7.12 (m, 4H), 7.16 (s, 1H), 7.50 (dd, 1H, *J*=8.9, 4.6 Hz), 7.57 (dd, 1H, *J* =8.9, 4.6 Hz), 7.70 (s, 1H), 8.37 (dd, 1H, *J*= 10.7, 2.5 Hz); ^{13}C{1H} NMR (DMSO-d_6, 50 MHz) δ: 33.4, 33.7, 105.0 (d, *J*=24.2 Hz), 108.6 (d, *J*=25.6 Hz), 109.6 (d, *J*=4.5 Hz), 109.9 , 110.2 (d, *J*=25.6 Hz), 111.1 (d, *J*=26.3 Hz), 111.9 (d, *J*=4.1 Hz), 112.0, 112.2 (d, *J*=8.7 Hz), 126.7 (d, *J*=10.1 Hz), 127.8 (d, *J*=11.9 Hz), 131.9, 134.0 (d, *J*=12.8 Hz), 135.9, 142.9, 152.8, 157.5 (d, *J*=233.0 Hz), 158.8 (d, *J*=236.8 Hz), 161.2. LC-MS: *m/z* [M + H]$^+$ calcd for $C_{21}H_{17}F_2N_6^+$ 391.14; found, 391.42. *Anal.* Calculated for $C_{21}H_{16}F_2N_6$ (MW: 390.39) C, 64,61; H, 4.13; N, 21.53%. Found: C, 64.83; H, 3.98; N, 21.40%.

5-(5-Methoxy-1-methyl-1H-indol-3-yl)-6-(1-methyl-1H-indol-3-yl)-1,2,4-triazin-3-amine (***5h***). Yield: 41% (overall yield: 98%); yellow solid; m.p.: 272.5 °C; IR (cm^{-1}): 3374/3335 (NH_2); ^{1}H NMR (DMSO-*d*$_6$, 200 MHz) δ: 3.51 (s, 3H), 3.70 (s, 3H), 3.85 (s, 3H), 6.83-6.98 (m, 4H), 7.10 (s, 1H), 7.16-7.34 (m, 3H), 7.51-7.56 (m, 2H), 7.86 (s, 1H); ^{13}C{1H} NMR (DMSO-*d*$_6$, 50 MHz) δ: 33.1, 33.5, 55.7, 105.3, 109.8, 110.6, 111.4, 112.2, 112.7, 119.9, 120.5, 121.9, 126.5, 127.7, 130.0, 132.4, 134.9, 137.1, 143.5, 153.3, 155.3, 161.2. LC-MS: *m/z* [M + H]$^+$ calcd for $C_{22}H_{20}N_6O^+$ 385.17; found, 385.45. *Anal.* Calculated for $C_{22}H_{20}N_6O$ (MW: 384.43) C, 68.73; H, 5.24; N, 21.86%. Found: 68.53; H, 5.37; N, 21.53%.

6-(5-Methoxy-1-methyl-1H-indol-3-yl)-5-(1-methyl-1H-indol-3-yl)-1,2,4-triazin-3-amine (***6h***). Yield: 57% (overall yield: 98%); yellow solid; m.p.: 250.2 °C; IR (cm^{-1}): 3428/3304 (NH_2); ^{1}H NMR (DMSO-*d*$_6$, 200 MHz) δ: 3.41 (s, 3H), 3.54 (s, 3H), 3.83 (s, 3H), 6.58 (m, 1H), 6.79-6.90 (m, 3H), 7.11 (s, 1H), 7.14-7.27 (m, 2H), 7.40-7.47 (m, 2H), 7.56 (s, 1H), 8.47-8.50 (m, 1H); ^{13}C{1H} NMR (DMSO-*d*$_6$, 50 MHz) δ: 32.8, 33.0, 55.0, 101.6, 109.8, 110.3, 111.0, 111.2, 111.7, 120.9, 122.4, 122.8, 126.3, 126.8, 130.0, 132.0, 134.1, 136.9, 143.5, 152.7, 153.7, 160.8. LC-MS: *m/z* [M + H]$^+$ calcd for $C_{22}H_{20}N_6O^+$ 385.17; found, 385.45. *Anal.* Calculated for $C_{22}H_{20}N_6O$ (MW: 384.43) C, 68.73; H, 5.24; N, 21.86%. Found: C, 68.57; H, 5.42; N, 21.58%.

5-(5-Fluoro-1-methyl-1H-indol-3-yl)-6-(1-methyl-1H-indol-3-yl)-1,2,4-triazin-3-amine (***5i***). Yield: 34% (overall yield: 73%); yellow solid; m.p.: 279.4 °C ; IR (cm^{-1}): 3462/3266 (NH_2); ^{1}H NMR (DMSO-*d*$_6$, 200 MHz,) δ: 3.57 (s, 3H), 3.86 (s, 3H), 6.93 (s, 2H), 6.99 (dd, 1H, *J*= 10.5, 2.1 Hz), 7.05 (dd, 1H, *J*=9.6, 2.0 Hz), 7.15-7.28 (m, 3H), 7.44-7.47 (m, 1H), 7.55 (dd, 1H, *J*=8.9, 4.5 Hz), 7.66 (s, 1H), 8.48-8.50 (m, 1H); ^{13}C{1H} NMR (DMSO-*d*$_6$, 50 MHz) δ: 33.3, 33.4, 105.1 (d, *J*=23.7), 110.1 (d, *J*=25.6), 110.8, 111.9 (d, *J*=9.9 Hz), 112.0, 112.1 (d, *J*=4.9 Hz), 121.4, 122.9, 123.4, 126.7 (d, *J*=10.8 Hz), 127.2, 131.9, 133.9, 134.4, 137.3, 143.2, 153.1, 157.7 (d, *J*=232.4 Hz), 161.3. LC-MS: *m/z* [M + H]$^+$ calcd for $C_{21}H_{18}FN_6O^+$ 373.15; found, 373.39. *Anal.* Calculated for $C_{21}H_{17}FN_6$ (MW: 372.40) C, 67.73; H, 4.60; N, 22.57%. Found: C, 67.50; H, 4.72; N, 22.39%.

6-(5-Fluoro-1-methyl-1H-indol-3-yl)-5-(1-methyl-1H-indol-3-yl)-1,2,4-triazin-3-amine (***6i***). Yield: 39% (overall yield: 73%); yellow solid; m.p.: 254.4 °C; IR (cm^{-1}): 3387/3366 (NH_2); ^{1}H NMR (DMSO-*d*$_6$, 200 MHz) δ: 3.50 (s, 3H), 3.89 (s, 3H), 6.92-6.99 (m, 3H), 7.07 (s, 1H), 7.12 (dd, 1H, *J*=9.2, 2.4 Hz), 7.16-7.22 (m, 2H), 7.46 (dd, 1H, *J*=8.9, 4.6 Hz) 7.53-7.56 (m, 1H), 7.63 (s, 1H), 8.38 (dd, 1H, *J*=10.7, 2.4 Hz); ^{13}C{1H} NMR (DMSO-*d*$_6$, 50 MHz) δ: 32.7, 33.3, 108.2 (d, *J*=24.5 Hz), 109.5 (d, *J*=4.6 Hz) 110.3, 110.6 (d, *J*=26.4 Hz), 111.5, 111.6, 111.7 (d, *J*=9.7 Hz), 119.5, 119.9, 121.6, 126.0, 127.4 (d, *J*=11.3 Hz), 129.6, 135.7, 136.7, 142.8, 152.5, 159.9 (d, *J*=232.9 Hz), 160.8. LC-MS: *m/z* [M + H]$^+$ calcd for $C_{21}H_{18}FN_6O^+$ 373.15; found, 373.40. *Anal.* Calculated for $C_{21}H_{17}FN_6$ (MW: 372.40) C, 67.73; H, 4.60; N, 22.57%. Found: C, 67.53; H, 4.79; N, 22.68%.

5-(5-Bromo-1-methyl-1H-indol-3-yl)-6-(1-methyl-1H-indol-3-yl)-1,2,4-triazin-3-amine (***5j***). Yield: 62% (overall yield: 97%); yellow solid; m.p.: 257.3 °C; IR (cm^{-1}): 3428/3336 (NH_2); ^{1}H NMR (DMSO-*d*$_6$, 200 MHz) δ: 3.50 (s, 3H), 3.88 (s, 3H), 6.90-6.95 (m, 1H), 7.02 (s, 2H), 7.10 (s, 1H), 7.13-7.20 (m, 2H), 7.37 (dd, 1H, *J*=8.7, 1.7 Hz), 7.43 (d, 1H, *J*=8.7 Hz), 7.54 (m, 1H), 7.62 (s, 1H), 8.65 (m, 1H); ^{13}C{1H} NMR (DMSO-*d*$_6$, 50 MHz) δ: 33.1, 33.6, 109.7, 110.6, 111.9, 112.9, 114.6, 119.9, 120.4, 122.0, 125.4, 125.5, 126.3, 128.8, 130.0, 135.7, 136.1, 137.2, 143.5, 152.7, 161.3. LC-MS: *m/z* [M + H]$^+$ calcd for $C_{21}H_{18}BrN_6^+$ 433.07; found, 433.30. *Anal.* Calculated for $C_{21}H_{17}BrN_6$ (MW: 433.30) C, 58.21; H, 3.95; N, 19.40%. Found: C, 58.45; H, 3.72; N, 19.33%.

6-(5-Bromo-1-methyl-1H-indol-3-yl)-5-(1-methyl-1H-indol-3-yl)-1,2,4-triazin-3-amine (***6j***). Yield: 35% (overall yield: 97%); yellow solid; m.p.: 266.0 C; IR (cm^{-1}): 3405/328 (NH_2); ^{1}H NMR (DMSO-*d*$_6$, 200 MHz) δ: 3.60 (s, 3H), 3.84 (s, 3H), 6.95 (s, 2H), 7.13-7.18 (m, 1H), 7.21-7.27 (m, 2H), 7.31 (dd, 1H, *J*=8.7, 1.8 Hz), 7.45-7.50 (m, 2H), 7.54 (d, 1H, *J*=8.7 Hz), 7.60 (s, 1H), 8.42-8.44 (m, 1H); ^{13}C{1H} NMR (DMSO-*d*$_6$, 50 MHz) δ: 33.3, 33.4, 110.1, 110.8, 111.5, 112.7, 112.8, 121.4, 122.7, 122.9, 123.3, 124.4, 127.1, 128.3, 131.5, 134.4, 135.9, 137.4, 143.1, 153.2, 161.2. LC-MS: *m/z* [M + H]$^+$ calcd for $C_{21}H_{18}BrN_6^+$ 433.07; found, 433.34. *Anal.* Calculated for $C_{21}H_{17}BrN_6$ (MW: 433.30) C, 58.21; H, 3.95; N, 19.40%. Found: C, 58.55; H, 3.74; N, 19.54%.

6-(5-Fluoro-1-methyl-1H-indol-3-yl)-5-(5-methoxy-1-methyl-1H-indol-3-yl)-1,2,4-triazin-3-amine (***5k***). Yield: 49% (overall yield: 96%); yellow solid; m.p.: 239.5 °C; IR (cm^{-1}): 3483/3292 (NH_2); ^{1}H NMR (DMSO-*d*$_6$, 200 MHz) δ: 3.44 (s, 3H), 3.53 (s, 3H), 3.85 (s, 3H), 6.58 (d, 1H, *J*=2.1 Hz), 6.81 (dd, 1H, *J*=8.8, 2.1 Hz), 6.97 (s, 2H), 7.10 (dd, 1H, *J*=9.3, 2.5 Hz) 7.12 (s, 1H), 7.42-7.49 (m, 2H), 7.59 (s, 1H), 8.30 (dd, 1H, *J*=10.6, 2.4 Hz); ^{13}C{1H} NMR (DMSO-

d$_6$, 50 MHz) δ: 33.3, 33.7, 55.5, 102.0, 108.4 (d, *J*=25.0 Hz), 110.1 (d, *J*=4.8 Hz), 111.0 (d, *J*=26.1 Hz), 111.4, 111.5, 112.0 (d, *J*=10.1 Hz), 112.2, 126.7, 127.8 (d, *J*=11.1 Hz), 130.4, 132.4, 134.0, 136.0, 143.5, 152.8, 154.1, 158.7 (d, *J*=233.2 Hz), 161.2. LC-MS: *m/z* [M + H]$^+$ calcd for $C_{22}H_{20}FN_6O^+$ 403.16; found, 403.49. *Anal.* Calculated for $C_{22}H_{19}FN_6O$ (MW: 402.42) C, 65.66; H, 4.76; N, 20.88%. Found: C, 65.71; H, 4.49; N, 20.69%.

*5-(5-Fluoro-1-methyl-1H-indol-3-yl)-6-(5-methoxy-1-methyl-1H-indol-3-yl)-1,2,4-triazin-3-amine (**6k**).* Yield: 47% (overall yield: 96%); yellow solid; m.p.: 251.2 °C; IR (cm^{-1}): 3460/3285 (NH_2); ^{1}H NMR (DMSO-*d*$_6$, 200 MHz) δ: 3.56 (s, 3H), 3.69 (s, 3H), 3.84 (s, 3H), 6.85 (dd, 1H, *J*=2.5 Hz, 8.9 Hz), 6.93 (m, 2H), 6.99 (dd, 1H, *J*=10.0, 2.4 Hz), 7.05 (dd, 1H, *J*=9.3, 2.5 Hz), 7.17 (s, 1H), 7.35 (d, 1H, *J*= 8.9 Hz), 7.54 (dd, 1H, *J*=8.8, 4.4 Hz), 7.62 (s, 1H), 7.79 (d, 1H, *J*=1.6 Hz); ^{13}C{1H} NMR (DMSO-*d*$_6$, 50 MHz) δ: 33.4, 33.6, 55.7, 105.4 (d, *J*=25.8 Hz), 109.8, 109.9, 110.6 (d, *J*=26.3 Hz), 111.5, 111.8 (d, *J*=10.5), 112.2 (d, *J*=4.6 Hz), 112.7, 126.8 (d, *J*=10.2 Hz), 127.6, 131.8, 132.5, 133.9, 134.7, 143.2, 153.2, 155.3, 157.7 (d, *J*=232.4 Hz), 161.2. LC-MS: *m/z* [M + H]$^+$ calcd for $C_{22}H_{20}FN_6O^+$ 403.16; found, 403.45. *Anal.* Calculated for $C_{22}H_{19}FN_6O$ (MW: 402.42) C, 65.66; H, 4.76; N, 20.88%. Found: C, 65.82; H, 4.47; N, 20.64%.

*6-(5-Bromo-1-methyl-1H-indol-3-yl)-5-(5-methoxy-1-methyl-1H-indol-3-yl)-1,2,4-triazin-3-amine (**5l**).* Yield: 65% (overall yield: 94%); yellow solid; m.p.: 233.9 °C; IR (cm^{-1}): 3404/3282 (NH_2); ^{1}H NMR (DMSO-*d*$_6$, 200 MHz) δ: 3.41 (s, 3H), 3.54 (s, 3H), 3.84 (s, 3H), 6.54 (d, 1H, *J*=2.1 Hz), 6.79 (dd, 1H, *J*=8.8, 2.1 Hz), 7.0 (s, 2H), 7.15(s, 1H), 7.34-7.46 (m, 3H), 7.58 (s, 1H), 8.56 (m, 1H); ^{13}C{1H} NMR (DMSO-*d*$_6$, 50 MHz) δ: 33.3, 33.6, 55.4, 101.9, 109.9, 111.3, 111.4, 112.2, 112.8, 114.5, 125.1, 125.4, 126.6, 128.7, 130.4, 132.4, 135.6, 136.1, 143.8, 152.5, 154.1, 161.2; LC-MS: *m/z* [M + H]$^+$ calcd for $C_{22}H_{20}BrN_6O^+$ 463.08; found, 463.54. *Anal.* Calculated for $C_{22}H_{19}BrN_6O$ (MW: 463.33) C, 57.03; H, 4.13; N, 18.14%. Found: C, 56.78; H, 3.81; N, 17.81%.

*5-(5-Bromo-1-methyl-1H-indol-3-yl)-6-(5-methoxy-1-methyl-1H-indol-3-yl)-1,2,4-triazin-3-amine (**6l**).* Yield: 29% (overall yield: 94%); yellow solid; m.p.: 260.5 °C; IR (cm^{-1}): 3479/3292 (NH_2); ^{1}H NMR (DMSO-*d*$_6$, 200 MHz) δ: 3.59 (s, 3H), 3.68 (s, 3H), 3.82 (s, 3H), 6.85 (d, 1H, *J*=8.7 Hz), 6.96 (s, 2H), 7.22 (s, 1H), 7.29-7.36 (m, 2H), 7.51-7.57 (m, 3H), 7.74 (m, 1H); ^{13}C{1H} NMR (DMSO-*d*$_6$, 50 MHz) δ: 33.3, 33.6, 55.7, 105.1, 109.8, 111.5, 111.6, 112.6, 112.7, 112.8, 122.7, 124.4, 127.6, 128.3, 131.4, 132.5, 134.7, 135.9, 143.0, 153.3, 155.2, 161.2. LC-MS: *m/z* [M + H]$^+$ calcd for $C_{22}H_{20}BrN_6O^+$ 463.08; found, 463.43. *Anal.* Calculated for $C_{22}H_{19}BrN_6O$ (MW: 463.33) C, 57.03; H, 4.13; N, 18.14%. Found: C, 56.82; H, 3.92; N, 17.87%.

*6-(1H-indol-3-yl)-5-(1-methyl-1H-indol-3-yl)-1,2,4-triazin-3-amine (**5m**).* Yield: 43% (overall yield: 94%); yellow solid; m.p.: 246.6 °C; IR (cm^{-1}): 3634/3468 (NH_2), 3290 (NH); ^{1}H NMR (DMSO-*d*$_6$, 200 MHz) δ: 3.51 (s, 3H), 6.88-6.95 (m, 3H), 7.01 (s, 1H), 7.09-7.22 (m, 3H), 7.28-7.25 (m, 1H), 7.42-7.50 (m, 2H), 7.56 (d, 1H, *J*=2.5 Hz), 8.51-8.54 (m, 1H) 11.42 (s, 1H); ^{13}C{1H} NMR (DMSO-*d*$_6$, 50 MHz) δ 33.4, 110.3, 110.7, 112.3, 112.9, 119.7, 120.3, 121.4, 121.9, 122.9, 123.5, 125.8, 126.2, 127.2, 134.5, 136.6, 137.3, 143.9, 153.4, 161.3. LC-MS: *m/z* [M + H]$^+$ calcd for $C_{20}H_{17}N_6^+$ 341.14; found, 341.39. *Anal.* Calculated for $C_{20}H_{16}N_6$ (MW: 340.38): C, 70.57; H, 4.74; N, 24.69%. Found: C, 70.77; H, 4.62; N, 24.39%.

*5-(1H-Indol-3-yl)-6-(1-methyl-1H-indol-3-yl)-1,2,4-triazin-3-amine (**6m**).* Yield: 51% (overall yield: 94%); yellow solid; m.p.: 311.3 °C; IR (cm^{-1}): 3445/3404 (NH_2), 3262 (NH); ^{1}H NMR (DMSO-*d*$_6$, 200 MHz) δ: 3.89 (s, 3H), 6.88-6.98 (m, 4H), 7.08-7.21 (m, 4H), 7.37 (s, 1H), 7.52-7.56 (m, 1H), 7.65 (s, 1H), 8.65-8.70 (m, 1H), 11.39 (s, 1H); ^{13}C{1H} NMR (DMSO-*d*$_6$, 50 MHz) δ 32.6, 110.2, 110.3, 111.8, 112.0, 119.4, 119.8, 120.7, 121.4, 121.5, 122.4, 122.5, 123.1, 125.9, 126.4, 129.4, 130.7, 136.1, 136.6, 160.7. LC-MS: *m/z* [M + H]$^+$ calcd for $C_{20}H_{17}N_6^+$ 341.14; found, 341.35. *Anal.* Calculated for $C_{20}H_{16}N_6$ (MW: 340.38): C, 70.57; H, 4.74; N, 24.69%. Found: C, 70.66; H, 4.52; N, 24.51%.

*5-(5-Fluoro-1-methyl-1H-indol-3-yl)-6-(1H-indol-3-yl)-1,2,4-triazin-3-amine (**5n**).* Yield: 42% (overall yield: 80%); yellow solid; m.p.: 254.3 °C; IR (cm^{-1}): 3460/3400 (NH_2), 3260 (NH); ^{1}H NMR (DMSO-*d*$_6$, 200 MHz) δ: 3.88 (s, 3H), 6.83-6.86 (m, 1H), 6.95 (s, 2H), 7.00-7.04 (m, 2H), 7.22-7.12 (m, 2H), 7.38-7.40 (m, 1H), 7.56 (dd, 1H, *J*= 9.8, 5.2 Hz), 7.70 (s, 1H), 8.59-8.61 (m, 1H), 11.41 (s, 1H); ^{13}C{1H} NMR (DMSO-*d*$_6$, 50 MHz) δ: 33.4, 105.0 (d, *J*=23.8 Hz), 110.2 (d, *J*=26.1 Hz), 110.8, 111.9 (d, *J*=9.8 Hz), 112.3, 112.6 (d, *J*=4.8 Hz), 121.2, 122.9, 123.5, 126.7 (d, *J*=10.4 Hz), 126.8, 130.8, 131.8, 133.9, 136.7, 143.0, 153.7, 157.7 (d, *J*=232.9 Hz), 161.4. LC-MS: *m/z* [M + H]$^+$ calcd for $C_{20}H_{16}FN_6^+$ 359.13; found, 359.25. *Anal.* Calculated for $C_{20}H_{15}FN_6$ (MW: 358.37) C, 67.03; H, 4.22; N, 23.45%. Found: C, 67.22; H, 3.88; N, 23.30%.

*6-(5-Fluoro-1-methyl-1H-indol-3-yl)-5-(1H-indol-3-yl)-1,2,4-triazin-3-amine (**6n**).* Yield: 38% (overall yield: 80%); yellow solid; m.p.: 277.3 °C; IR (cm^{-1}): 3493/3287 (NH_2), 3138 (NH); ^{1}H NMR (DMSO-*d$_6$*, 200 MHz) δ: 3.50 (s, 3H), 6.90-7.00 (m, 4H), 7.07-7.22 (m, 3H), 7.44-7.51 (m, 2H), 7.59 (s, 1H), 8.32-8.35 (m, 1H), 11.43 (s, 1H); ^{13}C{1H} NMR (DMSO-*d$_6$*, 50 MHz) δ: 33.7, 108.61 (d, *J*=25.2 Hz), 110.0 (d, *J*=4.6 Hz), 111.0 (d, *J*=26.3 Hz), 112.0, (d, *J*=10.1 Hz), 112.4, 112.8, 119.8, 120.1, 122.0, 125.8, 126.1, 127.8 (d, *J*=11.2 Hz), 134.0, 136.0, 136.6, 143.5, 153.1, 160.3 (d, *J*=233.2 Hz), 161.2. LC-MS: *m/z* [M + H]$^+$ calcd for $C_{20}H_{16}FN_6^+$ 359.13; found, 359.27. *Anal.* Calculated for $C_{20}H_{15}FN_6$ (MW: 358.37) C, 67.03; H, 4.22; N, 23.45%. Found: C, 67.18; H, 3.94; N, 23.20%.

*5-(5-Bromo-1-methyl-1H-indol-3-yl)-6-(1H-indol-3-yl)-1,2,4-triazin-3-amine (**5o**).* Yield: 60% (overall yield: 91%); yellow solid; m.p.: 271.0 °C; IR (cm^{-1}): 3472/3367 (NH_2), 3314 (NH); ^{1}H NMR (DMSO-*d$_6$*, 200 MHz) δ: 3.86 (s, 3H), 6.97 (s, 2H), 7.06 (s, 1H), 7.12-7.22 (m, 2H), 7.30 (dd, 1H, *J*=8.7, 1.5 Hz), 7.37-7.41 (m, 2H), 7.54 (d, 1H, *J*=8.7 Hz), 7.65 (s, 1H), 8.56-8.58 (m, 1H), 11.41 (s, 1H); ^{13}C{1H} NMR (DMSO-*d$_6$*, 50 MHz) δ: 33.3, 110.8, 112.1, 112.4, 112.7, 112.9, 121.2, 122.5, 122.9, 123.4, 124.5, 126.8, 128.3, 130.8, 131.4, 135.9, 136.7, 142.8, 153.8, 161.4; LC-MS: *m/z* [M + H]$^+$ calcd for $C_{20}H_{16}BrN_6^+$ 419.05; found, 419.30. *Anal.* Calculated for $C_{20}H_{15}BrN_6$ (MW:419.28) C, 57.29; H, 3.61; N, 24.04%. Found: C, 57.17; H, 3.39; N, 23.82%.

*6-(5-Bromo-1-methyl-1H-indol-3-yl)-5-(1H-indol-3-yl)-1,2,4-triazin-3-amine (**6o**).* Yield: 31% (overall yield: 91%); yellow solid; m.p.: 291.0 °C; IR (cm^{-1}): 3485/3308 (NH_2), 3198 (NH); ^{1}H NMR (DMSO-*d$_6$*, 200 MHz) δ: 3.50 (s, 3H), 6.89-6.94 (m, 1H), 7.03 (m, 2H), 7.10-7.15 (m, 1H), 7.18-7.21 (m, 1H), 7.34-7.38 (m, 2H), 7.43 (d, 1H, *J*=8.7 Hz), 7.47-7.50 (m, 1H), 7.57 (d, 1H, *J*=1.7 Hz), 8.64 (s, 1H), 11.43 (s, 1H); ^{13}C{1H} NMR (DMSO-*d$_6$*, 50 MHz) δ: 33.6, 109.8, 112.4, 112.6, 112.9, 114.6, 119.8, 120.1, 122.0, 125.4, 125.5, 125.9, 126.0, 128.8, 135.6, 136.1, 136.6, 143.8, 153.0, 161.2; LC-MS: *m/z* [M + H]$^+$ calcd for $C_{20}H_{16}BrN_6^+$ 419.05; found, 419.29. *Anal.* Calculated for $C_{20}H_{15}BrN_6$ (MW: 419.28) C, 57.29; H, 3.61; N, 24.04%. Found: C, 57.41; H, 3.43; N, 23.95%.

6-(1H-Indol-3-yl)-5-(5-methoxy-1-methyl-1H-indol-3-yl)-1,2,4-triazin-3-amine (***5p***). Yield: 47% (overall yield: 81%); yellow solid; m.p.: 288.4 °C; IR (cm^{-1}): 3499/3368 (NH_2), 3190 (NH); ^{1}H NMR (DMSO-*d$_6$*, 200 MHz) δ: 3.39 (s, 3H), 3.85 (s, 3H), 6.50-6.51 (m, 1H), 6.79 (dd, 1H, *J*=8.8, 2.2 Hz), 6.95 (s, 2H), 7.04 (d, 1H, *J*=2.8 Hz), 7.13-7.21 (m, 2H), 7.37-7.43 (m, 2H), 7.59 (s, 1H), 8.60 (m, 1H), 11.39 (s, 1H); ^{13}C{1H} NMR (DMSO-*d$_6$*, 50 MHz): δ: 33.3, 55.4, 101.8, 110.9, 111.5, 112.0, 112.1, 112.3, 121.2, 122.9, 123.3, 126.7, 126.9, 130.4, 131.1, 132.3, 136.6, 143.6, 154.0, 154.1, 161.1. LC-MS: *m/z* [M + H]$^+$ calcd for $C_{21}H_{19}N_6O^+$ 371.15; found, 371.28. *Anal.* Calculated for $C_{21}H_{18}N_6O$ (MW: 370.41) C, 68.09; H, 4.90; N, 22.69%. Found: C, 68.25; H, 4.75; N, 22.84%.

5-(1H-Indol-3-yl)-6-(5-methoxy-1-methyl-1H-indol-3-yl)-1,2,4-triazin-3-amine (***6p***). Yield: 34% (overall yield: 81%); yellow solid; m.p.: 279.6 °C; IR (cm^{-1}): 3480/3397 (NH_2), 3119 (NH); ^{1}H NMR (DMSO-*d$_6$*, 200 MHz) δ: 3.51 (s, 3H), 3.63 (s, 3H), 6.82 (dd, 1H, *J*=8.8, 2.5 Hz), 6.92-6.97 (m, 3H), 7.08-7.15 (m, 2H), 7.30-7.34 (m, 2H), 7.46-7.48 (m, 2H), 7.74 (d, 1H, *J*=2.4 Hz), 11.36 (s, 1H); ^{13}C{1H} NMR (DMSO-*d$_6$*, 50 MHz) δ: 33.5, 55.6, 105.1, 110.1, 111.4, 112.3, 112.6, 112.9, 119.8, 120.3, 121.9, 125.8, 126.2, 127.6, 132.4, 134.7, 136.6, 143.9, 153.4, 155.2, 161.2; LC-MS: *m/z* [M + H]$^+$ calcd for $C_{21}H_{19}N_6O^+$ 371.15; found, 371.30. *Anal.* Calculated for $C_{21}H_{18}N_6O$ (MW: 370.41) C, 68.09; H, 4.90; N, 22.69%. Found: C, 68.21; H, 5.04; N, 22.95%.

*6-(5-Bromo-1H-indol-3-yl)-5-(1-methyl-1H-indol-3-yl)-1,2,4-triazin-3-amine (**5q**).* Yield: 40% (overall yield: 74%); yellow solid; m.p.: 230.0 °C; IR (cm^{-1}): 3466/3372 (NH_2), 3181 (NH); ^{1}H NMR (DMSO-*d$_6$*, 200 MHz) δ: 3.89 (s, 3H), 6.89-6.94 (m, 1H), 7.01-7.08 (m, 4H), 7.14-7.19 (m, 1H), 7.30 (dd, 1H, *J*=8.6, 1.8 Hz), 7.35 (d, 1H, *J*=8.6 Hz), 7.52-7.54 (m, 1H), 7.66, (s, 1H), 8.76 (d, 1H, *J*=1.5 Hz), 11.55 (d, *J*=2.1 Hz, 1H); ^{13}C{1H} NMR (DMSO-*d$_6$*, 50 MHz): δ: 33.1, 110.5, 110.7, 112.3, 114.2, 114.3, 120.0, 120.2, 122.0, 125.4, 125.5, 126.3, 128.5, 129.9, 132.2, 135.4, 137.1, 143.3, 153.4, 161.3. LC-MS: *m/z* [M + H]$^+$ calcd for $C_{20}H_{16}BrN_6^+$ 419.05; found, 419.32. *Anal.* Calculated for $C_{20}H_{15}BrN_6$ (MW: 419.28) C, 57.29; H, 3.61; N, 20.04%. Found: C, 57.38; H, 3.85; N, 20.19%.

*5-(5-Bromo-1H-indol-3-yl)-6-(1-methyl-1H-indol-3-yl)-1,2,4-triazin-3-amine (**6q**).* Yield: 34% (overall yield: 74%); yellow solid; m.p.: 304.0 °C; IR (cm^{-1}): 3483/3275 (NH_2), 3134 (NH); ^{1}H NMR (DMSO-*d$_6$*, 200 MHz) δ: 3.60 (s, 3H), 6.97 (s, 2H), 7.11-7.13 (m, 1H), 7.17 (s, 1H), 7.21-7.27 (m, 2H), 7.44-7.47 (m, 2H), 7.53-7.57 (m, 2H), 8.36 (d, 1H, *J*=7.9 Hz), 11.58 (d, 1H, *J*= 1.7 Hz,); ^{13}C{1H} NMR (DMSO-*d$_6$*, 50 MHz): δ: 33.4, 110.3, 110.8, 112.3, 112.4, 114.3, 121.3, 122.6, 122.9, 123.2, 124.5, 127.0, 127.4, 128.1, 134.2, 135.3, 137.4, 143.6, 153.3, 161.3; LC-MS: *m/z* [M

+ H]+ calcd for $C_{20}H_{16}BrN_6^+$ 419.05; found, 419.29. *Anal.* Calculated for $C_{20}H_{15}BrN_6$ (MW: 419.28) C, 57.29; H, 3.61; N, 20.04%. Found: C, 57.43; H, 3.87; N, 20.15%.

*6-(5-Bromo-1H-indol-3-yl)-5-(5-bromo-1-methyl-1H-indol-3-yl)-1,2,4-triazin-3-amine (**5r**).* Yield: 42% (overall yield: 90%); yellow solid; m.p.: 239.0 °C; IR (cm^{-1}): 3360/3317 (NH_2), 3254 (NH); ^1H NMR (DMSO-d_6, 200 MHz) δ: 3.62 (s, 3H), 7.09 (s, 2H), 7.20 (s, 1H), 7.26-7.29 (m, 1H), 7.38-7.41 (m, 1H), 7.47-7.59 (m, 4H), 8.52 (m, 1H), 11.63 (s, 1H); $^{13}C\{1H\}$ NMR (DMSO-d_6, 50 MHz) δ: 33.6, 109.9, 112.2, 112.5, 113.0, 114.4, 114.6, 122.5, 124.5, 125.2, 125.5, 127.5, 128.1, 128.7, 135.3, 135.4, 136.2, 143.4, 152.8, 161.3; LC-MS: *m/z* [M + H]+ calcd for $C_{20}H_{15}Br_2N_6^+$ 496.96; found, 497.30. *Anal.* Calculated for $C_{20}H_{14}Br_2N_6$ (MW: 498.17) C, 48.22; H, 2.83; N, 16.87%. Found: C, 48.35; H, 2.60; N, 16.61%.

*5-(5-Bromo-1H-indol-3-yl)-6-(5-bromo-1-methyl-1H-indol-3-yl)-1,2,4-triazin-3-amine (**6r**).* Yield: 48% (overall yield: 90%); yellow solid; m.p.: 282.4 °C; IR (cm^{-1}): 3472/3275(NH_2), 3184 (NH); ^1H NMR (DMSO-d_6, 200 MHz) δ: 3.90 (s, 3H), 7.11-7.13 (m, 3H), 7.31-7.36 (m, 3H), 7.41 (d, 1H, *J*=8.4 Hz), 7.57 (d, 1H, *J*=8.6 Hz), 7.69 (s, 1H), 8.69 (m, 1H), 11.63 (s, 1H), $^{13}C\{1H\}$ NMR (DMSO-d_6, 50 MHz) δ: 33.4, 110.5, 111.8, 112.8, 112.9, 114.2, 114.4, 122.5, 124.5, 125.3, 125.6, 128.2, 128.5, 131.5, 132.0, 135.4, 135.9, 142.8, 153.3, 161.4. LC-MS: *m/z* [M + H]+ calcd for $C_{21}H_{18}BrN_6O^+$ 498.96; found, 499.17. *Anal.* Calculated for $C_{20}H_{14}Br_2N_6$ (MW: 498.17) C, 48.22; H, 2.83; N, 16.87%. Found: C, 48.37; H, 2.64; N, 16.71%.

*6-(5-Bromo-1H-indol-3-yl)-5-(5-methoxy-1-methyl-1H-indol-3-yl)-1,2,4-triazin-3-amine (**5s**).*Yield: 40% (overall yield: 78%); yellow solid; m.p.: 201.8 °C; IR (cm^{-1}): 3474/3277 (NH_2), 3179 (NH); ^1H NMR (DMSO-d_6, 200 MHz) δ: 3.38 (s, 3H), 3.85 (s, 3H), 6.46 (d, 1H, *J*=1.9 Hz), 6.78 (dd, 1H, *J*= 8.9, 2.4 Hz), 7.03 (s, 2H), 7.07 (d, 1H, *J*=2.8 Hz,), 7.30 (dd, 1H, *J*=1.9 Hz, 8.6 Hz), 7.37(d, 1H, *J*=8.6 Hz), 7.42 (d, 1H, *J*=8.9 Hz), 7.61 (s, 1H), 8.69 (d, 1H, *J*=1.6 Hz,), 11.56 (m, 1H); $^{13}C\{1H\}$ NMR (DMSO-d_6, 50 MHz) δ: 33.3, 55.1, 101.8, 110.6. 111.5, 111.8, 112.1, 114.2, 114.3. 125.1, 125.5, 126.6, 128.5, 130.4, 132.2. 132.4, 135.4, 143.6, 153.3, 154.1, 161.1; LC-MS: *m/z* [M + H]+ calcd for $C_{21}H_{18}BrN_6O^+$ 449.06; found, 449.30. *Anal.* Calculated for $C_{21}H_{17}BrN_6O$ (MW: 449.30) C, 56.14; H, 3.81; N, 18.70%. Found: C, 55.81; H, 3.62; N, 18.95%.

*5-(5-Bromo-1H-indol-3-yl)-6-(5-methoxy-1-methyl-1H-indol-3-yl)-1,2,4-triazin-3-amine (**6s**).* Yield: 38% (overall yield: 78%); yellow solid; m.p.: 207.7 °C; IR (cm^{-1}): 3428/3315 (NH_2), 3169 (NH); ^1H NMR (DMSO-d_6, 200 MHz) δ: 3.60 (s, 6H), 6.82 (dd, 1H, *J*=8.8, 2.4 Hz), 6.95 (s, 2H), 7.20 (s, 1H), 7.25 (dd, 1H, *J*=8.6, 1.8 Hz), 7.35 (d, 1H, *J*=8.8 Hz), 7.45 (d, 1H, *J*=8.6 Hz), 7.48 (d, 1H, *J*=2.4 Hz), 7.59-7.60 (m, 2H), 11.53 (m, 1H); $^{13}C\{1H\}$ NMR (DMSO-d_6, 50 MHz) δ: 33.6, 55.6. 104.8, 110.1, 111.5, 112.3, 112.4, 112.7, 114.3, 122.5, 124.5, 127.3, 127.4, 128.2. 132.5, 134.6, 135.3, 143.5, 153.4, 155.2, 161.1; LC-MS: *m/z* [M + H]+ calcd for $C_{21}H_{18}BrN_6O^+$ 449.06; found, 449.32. *Anal.* Calculated for $C_{21}H_{17}BrN_6O$ (MW: 449.30) C, 56.14; H, 3.81; N, 18.70%. Found: C, 55.88; H, 3.72; N, 18.93%.

*6-(5-Bromo-1H-indol-3-yl)-5-(5-fluoro-1-methyl-1H-indol-3-yl)-1,2,4-triazin-3-amine (**5t**).* Yield: 44% (overall yield: 86%); yellow solid; m.p.: 266.9 °C; IR (cm^{-1}): 3487/3275 (NH_2), 3132 (NH); ^1H NMR (DMSO-d_6, 200 MHz,) δ: 3.89 (s, 3H), 6.82 (dd, 1H, *J*=9.4, 2.4 Hz), 6.99-7.10 (m, 4H), 7.32 (dd, 1H, *J*=8.6, 1.7 Hz), 7.38 (d, 1H, *J*=8.6 Hz), 7.56 (dd, 1H, *J*=8.9, 4.4 Hz), 7.74 (m, 1H), 8.69 (s, 1H, *J*=1.2 Hz), 11.60 (s, 1H); $^{13}C\{1H\}$ NMR (DMSO-d_6, 50 MHz) δ: 33.4, 104.9 (d, *J*=23.7 Hz), 110.2 (d, *J*=26.6 Hz), 110.5, 110.6 (d, *J*=4.2 Hz) 111.9 (d, *J*=9.9 Hz), 114.3, 114.4, 125,3, 125.6, 126.5 (d, *J*=10.4 Hz), 128.4, 131.9, 132.1, 133.9, 135.4, 142.9, 153.3, 157.7 (d, *J*=232.9 Hz), 161.2; LC-MS: *m/z* [M + H]+ calcd for $C_{20}H_{15}BrFN_6^+$ 437.04; found, 437.30. *Anal.* Calculated for $C_{20}H_{14}BrFN_6$ (MW: 437.27) C, 54.94; H, 3.23; N, 19.22%. Found: C, 54.80; H, 3.11; N, 19.01%.

*5-(5-Bromo-1H-indol-3-yl)-6-(5-fluoro-1-methyl-1H-indol-3-yl)-1,2,4-triazin-3-amine (**6t**).* Yield: 42% (overall yield: 86%); yellow solid; m.p.: 314.3 °C; IR (cm^{-1}): 3499/3300 (NH_2), 3150 (NH); ^1H NMR (DMSO-d_6, 200 MHz) δ: 3.58 (s, 3H), 7.03-7.14 (m, 4H), 7.26 (dd, 1H, *J*=8.5, 1.9 Hz), 7.45-7.51 (m, 3H), 7.60 (d, 1H, *J*=2.3 Hz), 8.21 (dd, 1H, *J*=10.7, 2.6 Hz), 11.61-11.62 (m, 1H); $^{13}C\{1H\}$ NMR (DMSO-d_6, 50 MHz) δ: 33.8, 108.5 (d, *J*=24.9 Hz), 110.0 (d, *J*=4.5 Hz), 111.2 (d, *J*=26.4 Hz), 112.0, 112.2 (d, *J* =10.3 Hz), 112.5, 114.4, 122.4, 124.6, 127.5, 127.7 (d, *J*=11.2 Hz), 128.1, 134.1. 135.4, 136.1, 143.1, 153.5, 160.3 (d, *J*=233.3 Hz), 160.8. LC-MS: *m/z* [M + H]+ calcd for $C_{20}H_{15}BrFN_6^+$ 437.04; found, 437.35. *Anal.* Calculated for $C_{20}H_{14}BrFN_6$ (MW: 437.27): C, 54.94; H, 3.23; N, 19.22%. Found: C, 54.83; H, 3.31; N, 19.03%.

*5-(5-Fluoro-1-methyl-1H-indol-3-yl)-6-(5-fluoro-1H-indol-3-yl)-1,2,4-triazin-3-amine (**5u**).* Yield: 37% (overall yield: 79%); yellow solid; m.p.: 276.0 °C; IR (cm^{-1}): 3491/3285 (NH_2), 3138 (NH); ^{1}H NMR (DMSO-d_6, 200 MHz) δ: 3.58 (s, 3H), 6.98-7.12 (m, 6H), 7. 48-7.53 (m, 2H), 7.68 (m, 1H), 8.30 (dd, 1H, *J*=10.5, 2.1 Hz), 11.57 (s, 1H); ^{13}C{1H} NMR (DMSO-d_6, 50 MHz) δ: 33.8, 104.8 (d, *J*=23.7 Hz), 108.6 (d, *J*=25.0 Hz), 110.2 (d, *J*=5.0 HZ), 110.3 (d, *J*=26.2 Hz), 111.0 (d, *J*=27.0 Hz), 112.1 (d, *J*=9.9 Hz), 113.0 (d, *J*=4.7 Hz), 113.4 (d, *J*=9.8 Hz), 126.5 (d, *J*=10.1 Hz), 127.7 (d, *J*=11.2 Hz), 127.9, 133.3, 134.1, 135.8, 143.3, 152.9, 157.6 (d, *J*=232.0 Hz), 158.7 (d, *J*=233.2 Hz). 161.3. LC-MS: *m/z* [M + H]$^+$ calcd for $C_{20}H_{15}F_2N_6^+$ 377.12; found, 377.25. *Anal.* Calculated for $C_{20}H_{14}F_2N_6$ (MW:376.36): C, 63.83; H, 3.75; N, 22.33%. Found: C, 63.61; H, 3.82; N, 22.11%.

*6-(5-Fluoro-1H-indol-3-yl)-5-(5-fluoro-1-methyl-1H-indol-3-yl)-1,2,4-triazin-3-amine (**6u**).* Yield: 42% (overall yield: 79%); yellow solid; m.p.: 259.5 °C; IR (cm^{-1}): 3468/3298 (NH_2), 3192 (NH); ^{1}H NMR (DMSO-d_6, 200 MHz) δ: 3.89 (s, 3H), 6.84 (dd, 1H, *J*=9.9, 2.4 Hz), 7.00-7.08 (m, 5H), 7.40 (dd, 1H, *J*=8.9, 4.8 Hz), 7.54 (dd, 1H, *J*= 8.9, 4.5 Hz), 7.73 (s, 1H), 8.40 (dd, 1H, *J*=10.7, 2.4 Hz) 11.51 (s, 1H); ^{13}C{1H} NMR (DMSO-d_6, 50 MHz): δ: 33.4, 104.9 (d, *J*=21.9 Hz), 108.5 (d, *J*=25.0 Hz), 110.2 (d, *J*=26.1 Hz), 110.8 (d, *J*=4.7 Hz), 111.1 (d, *J*=26.3 Hz), 112.0 (d, *J*=9.8 Hz), 112.5 (d, *J*=4.7 Hz), 113.4 (d, *J*=9.8 Hz), 126.6 (d, *J*=10.2 Hz), 127.4 (d, *J*=11.2 Hz), 131.9, 132.5, 133.3, 133.9, 142.7, 153.4, 157.8 (d, *J*=222.3 Hz), 158.6 (d, *J*=232.1 Hz). 161.4; LC-MS: *m/z* [M + H]$^+$ calcd for $C_{20}H_{15}F_2N_6^+$ 377.12; found, 377.26. *Anal.* Calculated for $C_{20}H_{14}F_2N_6$ (MW: 376.36) C, 63.83; H, 3.75; N, 22.33%. Found: C, 63.65; H, 3.94; N, 22.15%.

*6-(5-Fluoro-1H-indol-3-yl)-5-(1-methyl-1H-indol-3-yl)-1,2,4-triazin-3-amine (**5v**).* Yield: 38% (overall yield: 84%); yellow solid; m.p.: 285.9 °C; IR (cm^{-1}): 3443/3294 (NH_2), 3144 (NH); ^{1}H NMR (DMSO-d_6, 200 MHz) δ: 3.60 (s, 3H), 6.97-7.29 (m, 7H), 7.47-7.50 (m, 2H), 7.62 (s, 1H), 8.44-8.47 (m, 1H), 11.52 (s, 1H). ^{13}C{1H} NMR (DMSO-d_6, 50 MHz) δ: 33.4, 104.9 (d, *J*=23.7 Hz), 110.2 (d, *J*=26.7 Hz), 110.3, 110.8, 113.3 (d, *J*=4.8 Hz), 113.4 (d, *J*=9.4 Hz), 121.4, 122.9, 123.4, 126.5 (d, *J*=10.5 Hz), 127.1, 127.9, 133.3, 134.3, 137.3, 143.7, 153.2, 157.5 (d, *J*=232.7 Hz), 161.3; LC-MS: *m/z* [M + H]$^+$ calcd for $C_{20}H_{16}FN_6^+$ 359.13; found, 359.31. *Anal.* Calculated for $C_{20}H_{15}FN_6$ (MW: 358.37) C, 67.03; H, 4.22; N, 23.45%. Found: C, 66.83; H, 4.35; N, 23.12%.

*5-(5-Fluoro-1H-indol-3-yl)-6-(1-methyl-1H-indol-3-yl)-1,2,4-triazin-3-amine (**6v**).* Yield: 46% (overall yield: 84%); yellow solid; m.p.: 295.8 °C; IR (cm^{-1}): 3468/3265 (NH_2), 3173 (NH); ^{1}H NMR (DMSO-d_6, 200 MHz) δ: 3.93 (s, 3H), 6.94-7.23 (m, 7H), 7.41 (dd, 1H, *J*=8.5, 4.7 Hz), 7.56-7.58 (m, 1H), 7.67-7.69 (m, 1H), 8.46-8.49 (m, 1H), 11.49 (s, 1H); ^{13}C{1H} NMR (DMSO-d_6, 50 MHz) δ: 33.1, 108.6 (d, *J*=24.3 Hz), 110.7, 110.8 (d, *J*=4.7 Hz), 111.1 (d, *J*=26.2 Hz), 112.5, 113.4 (d, *J*=10.0 Hz), 120.0, 120.3, 122.0, 126.4, 127.5 (d, *J*=11.5 Hz), 129.9, 133.3, 137.1, 143.1, 153.6, 156.9, 158.5 (d, *J*=231.8 Hz), 161.4; LC-MS: *m/z* [M + H]$^+$ calcd for $C_{20}H_{16}FN_6^+$ 359.13; found, 359.24. *Anal.* Calculated for $C_{20}H_{15}FN_6$ (MW: 358.37) C, 67.03; H, 4.22; N, 23.45%. Found: C, 66.93; H, 4.36; N, 23.23%.

*6-(5-Fluoro-1H-indol-3-yl)-5-(5-methoxy-1-methyl-1H-indol-3-yl)-1,2,4-triazin-3-amine (**5w**).* Yield: 40% (overall yield: 84%); yellow solid; m.p.: 276.0 °C; IR (cm^{-1}): 3482/3368 (NH_2), 3144 (NH); ^{1}H NMR (DMSO-d_6, 200 MHz) δ: 3.60 (s, 3H), 3.65 (s, 3H), 6.86 (dd, 1H, *J*=8.7, 1.7 Hz), 6.96 (s, 2H), 7.01-7.12 (m, 2H), 7.19 (s, 1H), 7.37 (d, 1H, *J*=8.7 Hz), 7.48-7.57 (m, 2H), 7.68 (s, 1H), 11.48 (s, 1H). ^{13}C{1H} NMR (DMSO-d_6, 50 MHz) δ: 33.6, 55.6, 104.8, 104.9 (d, *J*=23.7 Hz), 110.1, 110.2 (d, *J*=26.0 Hz), 111.5, 112.7, 113.1 (d, *J*=4.2 Hz), 113.3 (d, *J*=9.8 Hz), 126.5 (d, *J*=10.2 Hz), 127.4, 127.9, 132.4, 133.3, 134.6, 143.6, 153.2, 155.2, 157.6 (d, *J*=232.1 Hz), 161.2; LC-MS: *m/z* [M + H]$^+$ calcd for $C_{21}H_{18}FN_6O^+$ 389.14; found, 389.30. *Anal.* Calculated for $C_{21}H_{17}FN_6O$ (MW: 388.40) C, 64.94; H, 4.41; N, 21.64%. Found: C, 64.72; H, 4.09; N, 21.51%.

*5-(5-Fluoro-1H-indol-3-yl)-6-(5-methoxy-1-methyl-1H-indol-3-yl)-1,2,4-triazin-3-amine (**6w**).* Yield: 44% (overall yield: 84%); yellow solid; m.p.: 251.5 °C; IR (cm^{-1}): 3482/3348 (NH_2), 3125 (NH); ^{1}H NMR (DMSO-d_6, 200 MHz) δ: 3.44 (s, 3H), 3.89 (s, 3H) 6.51 (m, 1H), 6.81-6.84 (m, 1H), 7.02-7.06 (m, 4H), 7.39-7.47 (m, 2H), 7.64 (s, 1H), 8.39-8.43 (m, 1H), 11.52 (s, 1H). ^{13}C{1H} NMR (DMSO-d_6, 50 MHz) δ: 33.3, 55.4, 101.8, 108.3 (d, *J*=24.6 Hz), 110.8, 110.9 (d, *J*=4.6 Hz), 111.4 (d, *J*=21.2 Hz), 112.0, 112.1, 113.3 (d, *J*=9.8 Hz), 126.7, 127.4 (d, *J*=11.2 Hz), 130.3, 132.4, 132.6, 133.2, 143.3, 153.4, 154.1, 158.5 (d, *J*=232.5 Hz), 161.4; LC-MS: *m/z* [M + H]$^+$ calcd for $C_{21}H_{18}FN_6O^+$ 389.14; found, 389.33. *Anal.* Calculated for $C_{21}H_{17}FN_6O$ (MW: 388.40) C, 64.94; H, 4.41; N, 21.64%. Found: C, 64.81; H, 4.29; N, 21.41%.

*5-(5-Bromo-1-methyl-1H-indol-3-yl)-6-(5-fluoro-1H-indol-3-yl)-1,2,4-triazin-3-amine (**5x**).* Yield: 43% (overall yield: 82%); yellow solid; m.p.: 267.6 °C; IR (cm^{-1}): 3489/3265 (NH_2), 3173 (NH); ^{1}H NMR (DMSO-d_6, 200 MHz) δ: 3.88 (s, 3H), 7.01-7.08 (m, 4H), 7.31 (d, 1H, *J*=8.6, 1.7 Hz), 7.35 (d, 1H, *J*=1.4 Hz), 7.41 (d, 1H, *J*=8.8, 4.7 Hz),

7.55 (d, 1H, *J*=8.7 Hz), 7.68 (s, 1H), 8.37 (dd, 1H, *J*=10.7, 2.5 Hz), 11.52 (s, 1H); $^{13}C\{1H\}$ NMR (DMSO-d_6, 50 MHz) δ: 33.3, 108.4 (d, *J*=27.3 Hz), 110.8 (d, *J*=4.6 Hz), 111.1 (d, *J*=25.2 Hz), 112.0, 112.7, 112.9, 113.4 (d, *J*=8.6 Hz), 122.4, 124.5, 127.4 (d, *J*=11.1 Hz), 128.3, 131.5, 132.4, 133.3, 135.9, 142.5, 153.5, 158.5 (d, *J*=232.8 Hz), 161.4; LC-MS: *m/z* $[M + H]^+$ calcd for $C_{20}H_{15}BrFN_6^+$ 437.04; found, 437.24. *Anal.* Calculated for $C_{20}H_{14}BrFN_6$ (MW: 437.27) C, 54.94; H, 3.23; N, 19.22%. Found: C, 55.02; H, 3.42; N, 19.36%.

*6-(5-Bromo-1-methyl-1H-indol-3-yl)5-(5-fluoro-1H-indol-3-yl)-1,2,4-triazin-3-amine (**6x**).* Yield: 39% (overall yield: 82%); yellow solid; m.p.: 270.2 °C; IR (cm^{-1}): 3489/3383 (NH_2), 3138 (NH); ^1H NMR (DMSO-d_6, 200 MHz) δ: 3.56 (s, 3H), 6.95-7.03 (m, 4H), 7.12 (s, 1H), 7.36 (d, 1H, *J*=8.7, 1.9 Hz), 7.43 (s, 1H), 7.48 (dd, 1H, *J*=9.6, 4.6 Hz), 7.61-7.62 (m, 1H), 8.52 (d, 1H, *J*=1.8 Hz), 11.52 (s, 1H); $^{13}C\{1H\}$ NMR (DMSO-d_6, 50 MHz) δ: 33.6, 104.8 (d, *J*=25.7 Hz), 109.9, 110.2 (d, *J*=26.3 Hz), 112.8 (d, *J*=4.7 Hz), 112.9, 113.4 (d, *J*=9.5 Hz), 114.5, 125.2, 125.5, 126.4 (d, *J*=10.1 Hz), 127.8, 128.7, 133.3, 135.3, 135.4, 136.1, 143.5, 152.7, 160.2 (d, *J*=232.0 Hz), 161.3; LC-MS: *m/z* $[M + H]^+$ calcd for $C_{20}H_{15}BrFN_6^+$ 437.04; found, 437.10. *Anal.* Calculated for $C_{20}H_{14}BrFN_6$ (MW: 437.27) C, 54.94; H, 3.23; N, 19.22%. Found: C, 55.05; H, 3.41; N, 19.39%.

*5-(5-Methoxy-1-methyl-1H-indol-3-yl)-6-(5-methoxy-1H-indol-3-yl)-1,2,4-triazin-3-amine (**5y**).* Yield: 33% (overall yield: 68%); yellow solid; m.p.: 205.5 °C; IR (cm^{-1}): 3485-3300 (NH_2), 3181 (NH); ^1H NMR (DMSO-d_6, 200 MHz,) δ: 3.51-3.64 (m, 9H), 6.75-6.78 (m, 2H), 6.83 (dd, 1H, *J*=8.8, 2.4 Hz), 6.92 (s, 2H), 7.12 (s, 1H), 7.32-7.37 (m, 2H), 7.45 (d, 1H, *J*=2.5 Hz), 7.68-7.69 (m, 1H), 11.23 (s, 1H); $^{13}C\{1H\}$ NMR (DMSO-d_6, 50 MHz) δ: 33.6, 55.5, 55.6, 101.7, 104.9, 110.2, 111.5, 112.3, 112.5, 112.7, 113.0, 126.4, 126.6, 127.6, 131.7, 132.4, 135.0, 144.1, 153.6, 154.0, 155.2, 160.8; LC-MS: *m/z* $[M + H]^+$ calcd for $C_{22}H_{21}N_6O_2^+$ 401.16; found, 401.35. *Anal.* Calculated for $C_{22}H_{20}N_6O_2$ (MW: 400.43) C, 65.99; H, 5.03; N, 20.99%. Found: C, 65.75; H, 5.22; N, 21.10%.

*6-(5-Methoxy-1-methyl-1H-indol-3-yl)-5-(5-methoxy-1H-indol-3-yl)-1,2,4-triazin-3-amine (**6y**).* Yield: 35% (overall yield: 68%); yellow solid; m.p.: 263.3 °C; IR (cm^{-1}): 3465/3315 (NH_2), 3173 (NH); ^1H NMR (DMSO-d_6, 200 MHz) δ: 3.43 (s, 3H), 3.77 (s, 3H), 3.84 (s, 3H), 6.53 (s, 1H), 6.82-6.79 (m, 2H), 6.93-7.02 (m, 3H), 7.25-7.28 (m, 1H), 7.40-7.43 (m, 1H), 7.57 (s, 1H), 7.99 (s, 1H), 11.30 (s, 1H); $^{13}C\{1H\}$ NMR (DMSO-d_6, 50 MHz) δ: 33.2, 55.4, 55.8, 101.9, 105.1, 110.7, 111.4, 112.1, 112.2, 112.7, 112.8, 126.8, 127.4, 130.3, 131.3, 131.5, 132.3, 143.6, 153.8, 154.1, 155.0, 161.3; LC-MS: *m/z* $[M + H]^+$ calcd for $C_{22}H_{21}N_6O_2^+$ 401.16; found, 401.36. *Anal.* Calculated for $C_{22}H_{20}N_6O_2$ (MW: 400.43) C, 65.99; H, 5.03; N, 20.99%. Found: C, 65.71 H, 5.21; N, 21.15%.

*6-(5-Methoxy-1H-indol-3-yl)-5-(1-methyl-1H-indol-3-yl)-1,2,4-triazin-3-amine (**5z**).* Yield: 35% (overall yield: 67%); yellow solid; m.p.: 249.4 °C; IR (cm^{-1}): 3447-3404 (NH_2), 3150 (NH); ^1H NMR (DMSO-d_6, 200 MHz) δ: 3.78 (s, 3H), 3.88, (s, 3H), 6.81 (dd, 1H, *J*=8.7, 2.2 Hz), 6.91-6.98 (m, 4H), 7.12-7.20 (m, 2H), 7.26 (d, 1H, *J*=8.7 Hz), 7.52-7.54 (m, 1H), 7.62 (s, 1H), 8.06 (d, 1H, *J*=1.9 Hz), 11.28 (s, 1H); $^{13}C\{1H\}$ NMR (DMSO-d_6, 50 MHz) δ: 33.1, 55.9, 105.5, 110.6, 110.7, 112.6, 112.8, 112.9, 120.0, 120.4, 122.0, 126.4, 127.5, 129.9, 131.5, 131.6. 137.1, 143.3, 154.1, 155.1, 161.2; LC-MS: *m/z* $[M + H]^+$ calcd for $C_{21}H_{19}N_6O^+$ 371.15; found, 371.33. *Anal.* Calculated for $C_{21}H_{18}N_6O$ (MW: 370.41) C, 68.09; H, 4.90; N, 22.69%. Found: C, 68.36; H, 5.01; N, 22.72%.

*5-(5-Methoxy-1H-indol-3-yl)-6-(1-methyl-1H-indol-3-yl)-1,2,4-triazin-3-amine (**6z**).* Yield: 32% (overall yield: 67%); yellow solid; m.p.: 243.3 °C; IR (cm^{-1}): 3483/3275 (NH_2), 3171 (NH); ^1H NMR (DMSO-d_6, 200 MHz) δ: 3.45 (s, 3H), 3.54 (s, 3H), 6.65 (s, 1H), 6.74-6.77 (m, 1H), 6.91 (s, 2H), 7.06 (s, 1H), 7.13-7.26 (m, 2H), 7.37 (d, 1H, *J*=8.8 Hz), 7.45-7.50 (m, 2H), 8.44-8.46 (m, 1H), 11.26 (s, 1H); $^{13}C\{1H\}$ NMR (DMSO-d_6, 50 MHz) δ: 33.5, 55.4, 101.7, 110.4, 110.8, 112.3, 112.4, 113.0, 121.5, 122.9, 123.3, 126.4, 126.5, 127.2, 131.7, 134.7, 137.3, 144.2, 153.7, 153.9, 160.9. LC-MS: *m/z* $[M + H]^+$ calcd for $C_{21}H_{19}N_6O^+$ 371.15; found, 371.35. *Anal.* Calculated for $C_{21}H_{18}N_6O$ (MW: 370.41) C, 68.09; H, 4.90; N, 22.69%. Found: C, 68.28; H, 5.06; N, 22.82%.

5-(5-Bromo-1-methyl-1H-indol-3-yl)-6-(5-methoxy-1H-indol-3-yl)-1,2,4-triazin-3-amine (***5aa***). Yield: 37% (overall yield: 78%); yellow solid; m.p.: 267.8 °C; IR (cm^{-1}): 3404/3291 (NH_2), 3179 (NH); ^1H NMR (DMSO-d_6, 200 MHz) δ: 3.45 (s, 3H), 3.53 (s, 3H), 6.61 (m, 1H), 6.76 (dd, 1H, *J*=9.0, 1.7 Hz), 7.01, (s, 2H), 7.03 (s, 1H), 7.35-7.38 (m, 2H), 7.45 (d, 1H, *J*=8.7 Hz), 7.53 (d, 1H, *J*=2.1 Hz), 8.58 (d, 1H, *J*=1.2 Hz), 11.29 (s, 1H); $^{13}C\{1H\}$ NMR (DMSO-d_6, 50 MHz) δ: 33.6, 55.4, 101.6, 110.0, 112.3, 112.4, 112.9, 113.0, 114.5, 125.1, 125.4, 126.3, 126.4, 128.7, 131.7, 135.5, 136.0, 144.1, 152.7, 153.9, 161.3; LC-MS: *m/z* $[M + H]^+$ calcd for $C_{21}H_{18}BrN_6O^+$ 449.06; found, 449.30. *Anal.* Calculated for $C_{21}H_{17}BrN_6O$ (MW: 449.30) C, 56.14; H, 3.81; N, 18.70%. Found: C, 56.35; H, 3.69; N, 18.66%.

6-(5-Bromo-1-methyl-1H-indol-3-yl)-5-(5-methoxy-1H-indol-3-yl)-1,2,4-triazin-3-amine (***6aa***). Yield: 41% (overall yield: 78%); yellow solid; m.p.: 241.8 °C; IR (cm^{-1}): 3422/3325 (NH_2), 3298 (NH); ^1H NMR (DMSO-d_6, 200 MHz) δ: 3.76 (s, 3H), 3.86 (s, 3H), 6.79-6.83 (m, 1H), 6.99-7.06 (m, 3H), 7.26-7.31 (m, 2H), 7.39 (s, 1H), 7.53 (dd, 1H, *J*=8.7, 2.1 Hz), 7.63 (d, 1H, *J*=1.9 Hz), 7.95 (s, 1H), 11.35 (s, 1H); $^{13}C\{1H\}$ NMR (DMSO-d_6, 50 MHz) δ: 33.3, 55.8, 105.3, 110.6, 112.1, 112.7, 112.8 (x 2), 112.9, 122.5, 124.5, 127.3, 128.3, 131.2, 131.4, 131.6, 135.9, 142.7, 154.0, 155.1, 161.2; LC-MS: *m/z* $[M + H]^+$ calcd for $C_{21}H_{18}BrN_6O^+$ 449.06; found, 449.37. *Anal.* Calculated for $C_{21}H_{17}BrN_6O$ (MW: 449.30) C, 56.14; H, 3.81; N, 18.70%. Found: C, 56.37; H, 3.79; N, 18.70%.

5-(5-Fluoro-1-methy-1H-indol-3-yl)-6-(5-methoxy-1H-indol-3-yl)-1,2,4-triazin-3-amine (***5ab***). Yield: 39% (overall yield: 71%); yellow solid; m.p.: 250.3°C; IR (cm^{-1}): 3464/3424 (NH_2), 3130 (NH); ^1H NMR (DMSO-d_6, 200 MHz) δ: 3.76 (s, 3H), 3.87 (s, 3H), 6.82 (dd, 1H, *J*=8.8, 2.4 Hz), 6.87 (dd, 1H, *J*=9.9, 2.3 Hz), 6.95 (s, 2H), 6.99-7.06 (m, 2H), 7.28 (d, 1H, *J*=8.8 Hz), 7.54 (dd, 1H, *J*=9.9, 4.4 Hz). 7.68 (s, 1H), 7.98 (d, 1H, *J*=2.4 Hz), 11.31 (s, 1H); $^{13}C\{1H\}$ NMR (DMSO-d_6, 50 MHz) δ: 33.4, 55.8, 105.0 (d, *J*=23.6 Hz), 105.3, 110.1 (d, *J*=26.2 Hz), 110.6, 111.9 (d, *J*=9.9 Hz), 112.7 (d, *J*=4.8 Hz), 112.8, 112.9, 126.7 (d, *J*=10.2 Hz), 127.3, 131.2, 131.6, 131.8, 133.9, 143.0, 153.8, 155.1, 157.7 (d, *J*=232.6 Hz), 161.3; LC-MS: *m/z* $[M + H]^+$ calcd for $C_{21}H_{18}FN_6O^+$ 389.14; found, 389.36. *Anal.* Calculated for $C_{21}H_{17}FN_6O$ (MW: 388.40) C, 64.94; H, 4.41; N, 21.64%. Found: C, 64.70; H, 4.75; N, 21.55%.

6-(5-Fluoro-1-methyl-1H-indol-3-yl)-5-(5-methoxy-1H-indol-3-yl)-1,2,4-triazin-3-amine (***6ab***). Yield: 32% (overall yield: 71%); yellow solid; m.p.: 227.9 °C; IR (cm^{-1}): 3491/3289 (NH_2), 3180 (NH); ^1H NMR (DMSO-d_6, 200 MHz) δ: 3.47 (s, 3H), 3.53 (s, 3H), 6.62 (d, 1H, *J*=2.1 Hz), 6.77 (dd, 1H, *J*=8.7, 2.4 Hz), 6.97 (s, 2H), 7.05-7.13 (m, 2H), 7.38 (d, 1H, *J*=8.8 Hz), 7.47 (dd, 1H, *J*=8.9, 4.6 Hz), 7.54 (d, 1H, *J*=2.5 Hz), 8.27 (dd, 1H, *J*= 10.6, 2.5 Hz), 11.31 (s, 1H); $^{13}C\{1H\}$ NMR (DMSO-d_6, 50 MHz): δ: 33.8, 55.5, 101.6, 108.3 (d, *J*=25.0 Hz), 110.2 (d, *J*=4.6 Hz), 111.0 (d, *J*=26.2 Hz), 112.0 (d, *J*=10.0 Hz), 112.3, 112.5, 113.0, 126.3, 126.5, 127.7 (d, *J*=11.1 Hz), 131.7, 134.0, 135.9, 143.8, 152.9, 153.9, 158.7 (d, *J*=233.1 Hz), 161.3; LC-MS: *m/z* $[M + H]^+$ calcd for $C_{21}H_{18}FN_6O^+$ 389.14; found, 389.44. *Anal.* Calculated for $C_{21}H_{17}FN_6O$ (MW: 388.40) C, 64.94; H, 4.41; N, 21.64%. Found: C, 64.73; H, 4.65; N, 21.72%.

Table S1. 3-Amino-1,2,4-triazine derivatives **5,6** and their relative yields.

Cpd	R	R^1	R^2	R^3	Overall yield (%)	5(%) 6(%)
**5a**	H	H	H	H	55%	-
**5b**	OCH_3	H	OCH_3	H	78%	-
**5c**	F	H	F	H	61%	-
**5d**	Br	H	Br	H	70%	-
**5e**	H	CH_3	H	CH_3	85%	-
**5f**	OCH_3	CH_3	OCH_3	CH_3	98%	-
**5g**	F	CH_3	F	CH_3	77%	-
**5h** **6h**	H	CH_3	OCH_3	CH_3	98%	41% 57%
**5i** **6i**	H	CH_3	F	CH_3	73%	34% 39%
**5j** **6j**	H	CH_3	Br	CH_3	97%	62% 35%
**5k** **6k**	F	CH_3	OCH_3	CH_3	96%	49% 47%
**5l** **6l**	Br	CH_3	OCH_3	CH_3	94%	65% 29%
**5m** **6m**	H	H	H	CH_3	94%	43% 51%
**5n** **6n**	H	H	F	CH_3	80%	42% 38%
**5o** **6o**	H	H	Br	CH_3	91%	60% 31%
**5p** **6p**	H	H	OCH_3	CH_3	81%	47% 34%

Cpd	R	R^1	R^2	R^3	Overall yield (%)	5(%) 6(%)
**5q** **6q**	Br	H	H	CH_3	74%	40% 34%
**5r** **6r**	Br	H	Br	CH_3	90%	42% 48%
**5s** **6s**	Br	H	OCH_3	CH_3	78%	40% 38%
**5t** **6t**	Br	H	F	CH_3	86%	44% 42%
**5u** **6u**	F	H	F	CH_3	79%	37% 42%
**5v** **6v**	F	H	H	CH_3	84%	38% 46%
**5w** **6w**	F	H	OCH_3	CH_3	84%	40% 44%
**5x** **6x**	F	H	Br	CH_3	82%	43% 39%
**5y** **6y**	OCH_3	H	OCH_3	CH_3	68%	33% 35%
**5z** **6z**	OCH_3	H	H	CH_3	67%	35% 32%
**5aa** **6aa**	OCH_3	H	Br	CH_3	78%	37% 41%
**5ab** **6ab**	OCH_3	H	F	CH_3	71%	39% 32%

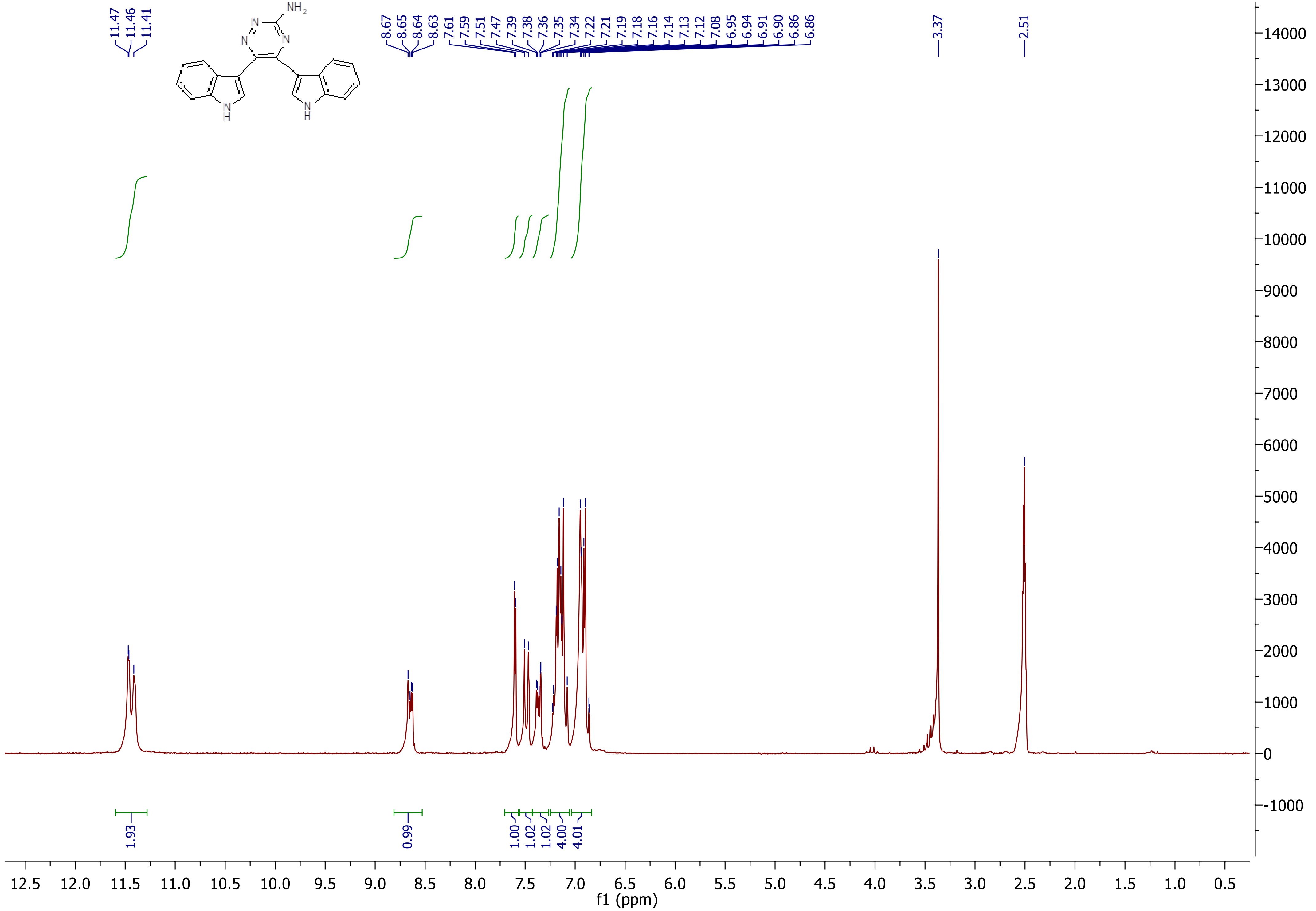

Figure S1: ^{1}H NMR spectrum of compound **5a**

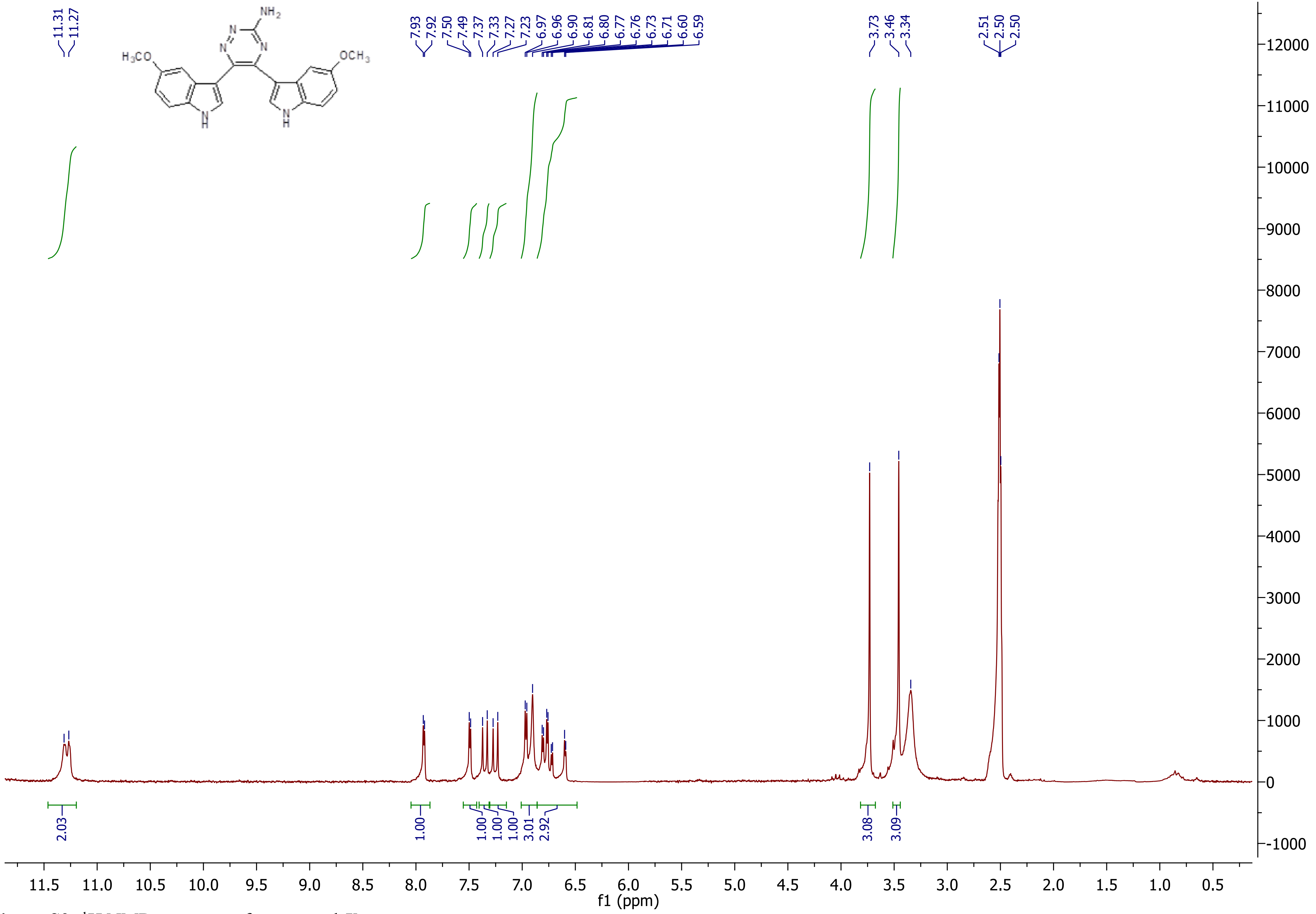

Figure S2: ^{1}H NMR spectrum of compound **5b**

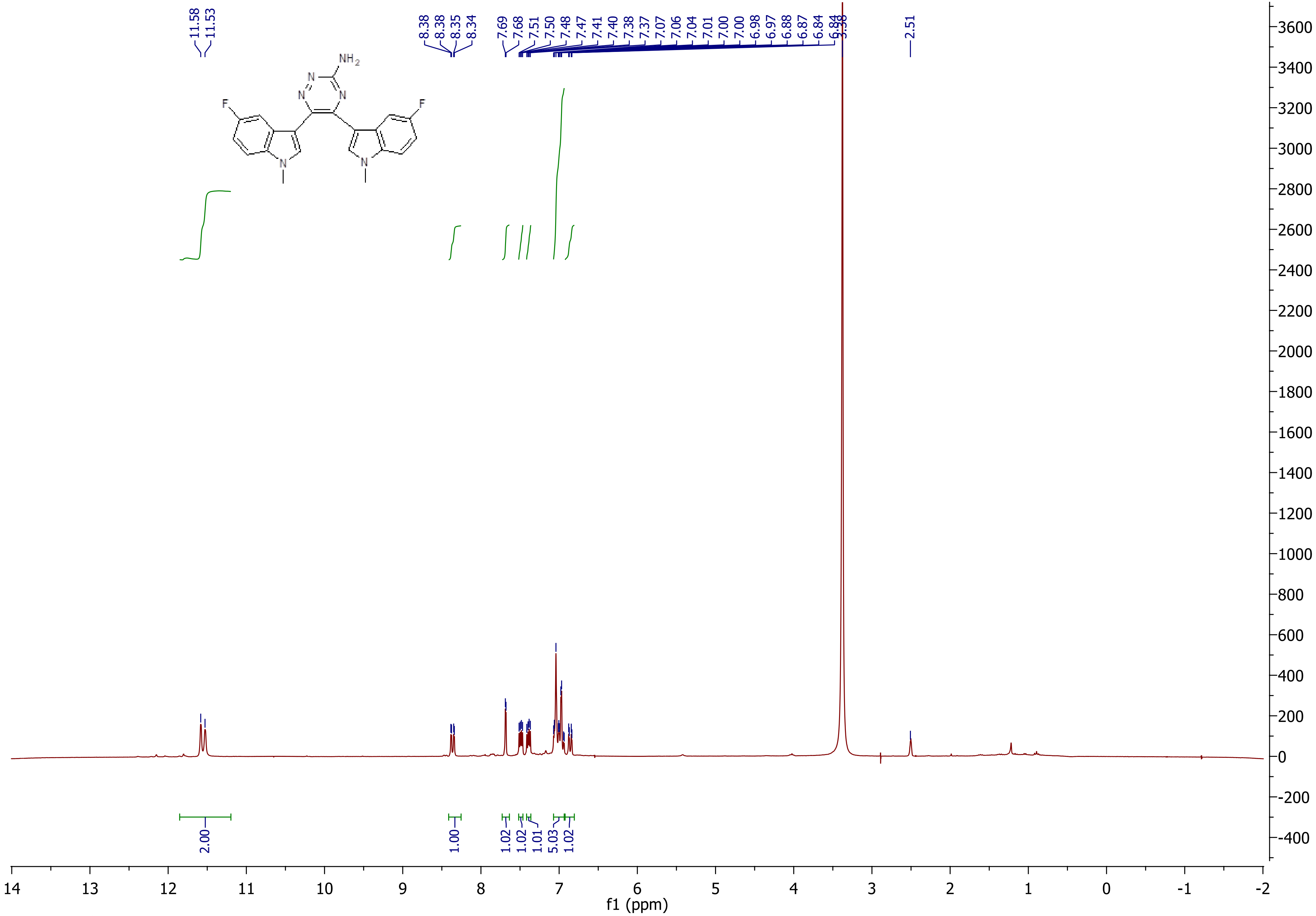

Figure S3: ^{1}H NMR spectrum of compound **5c**

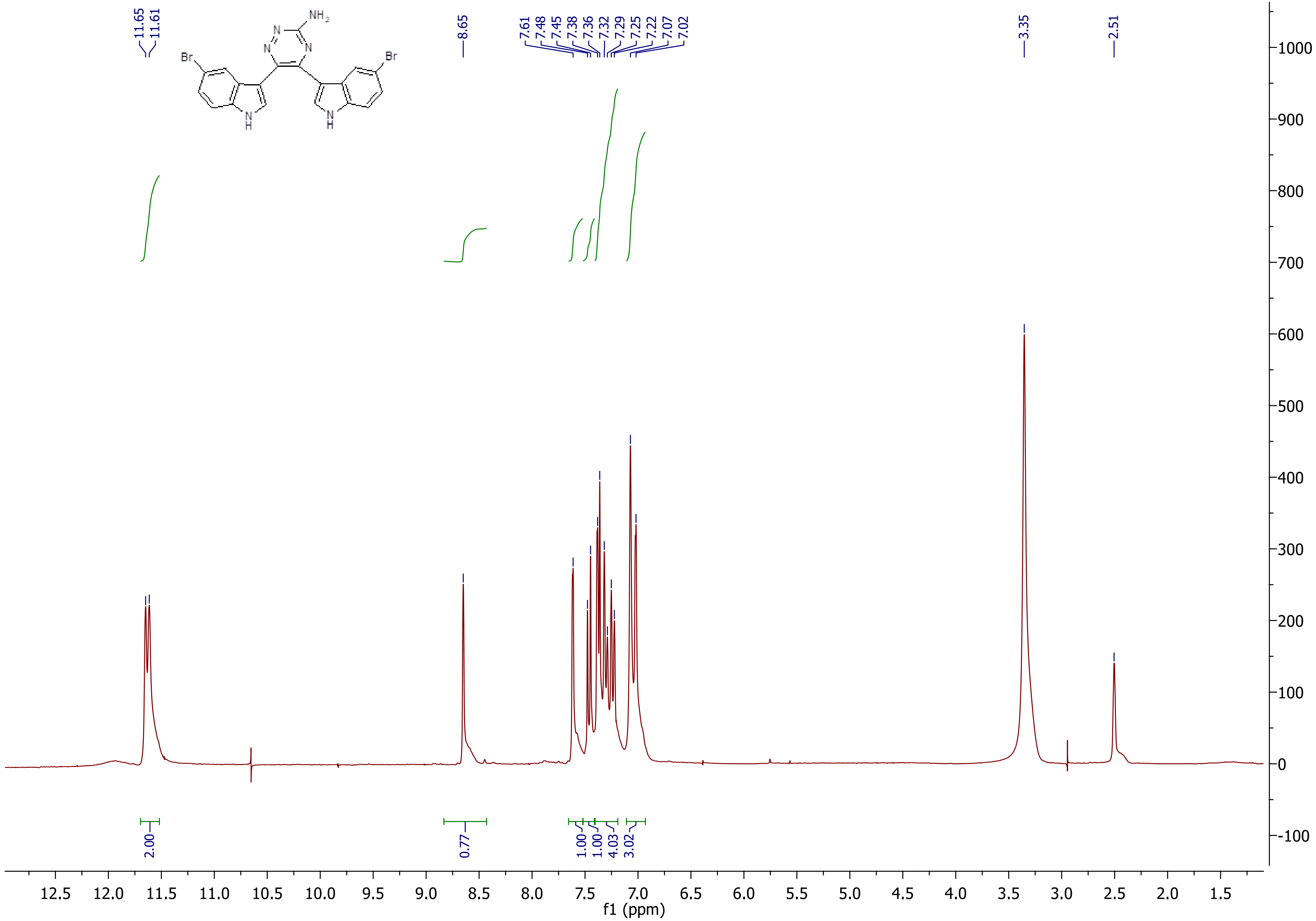

Figuere S4: ^{1}H NMR spectrum of compound **5d**

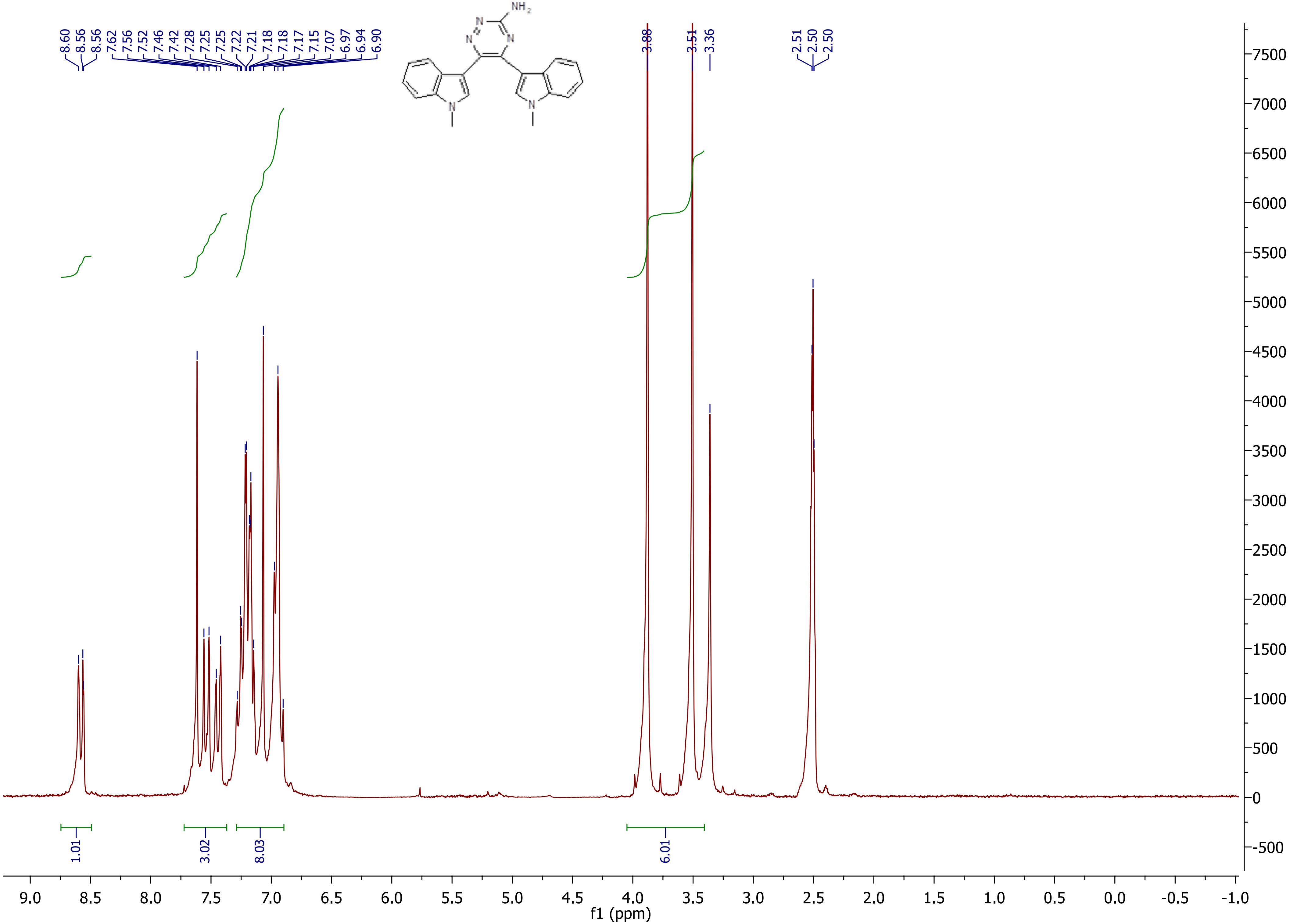

Figure S5: ^{1}H NMR spectra of compound **5e**

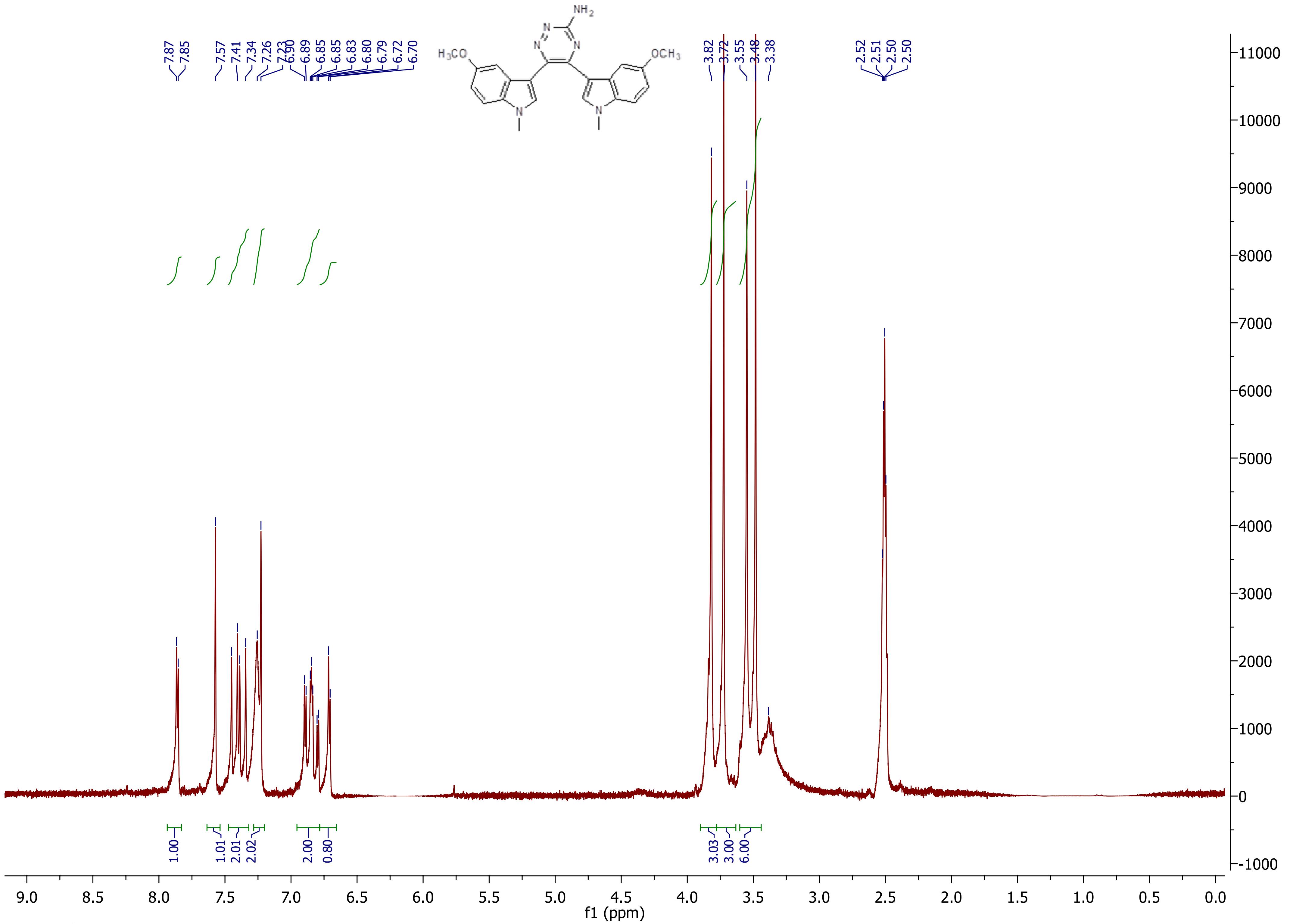

Figure S6: ^{1}H NMR spectrum of compound **5f**

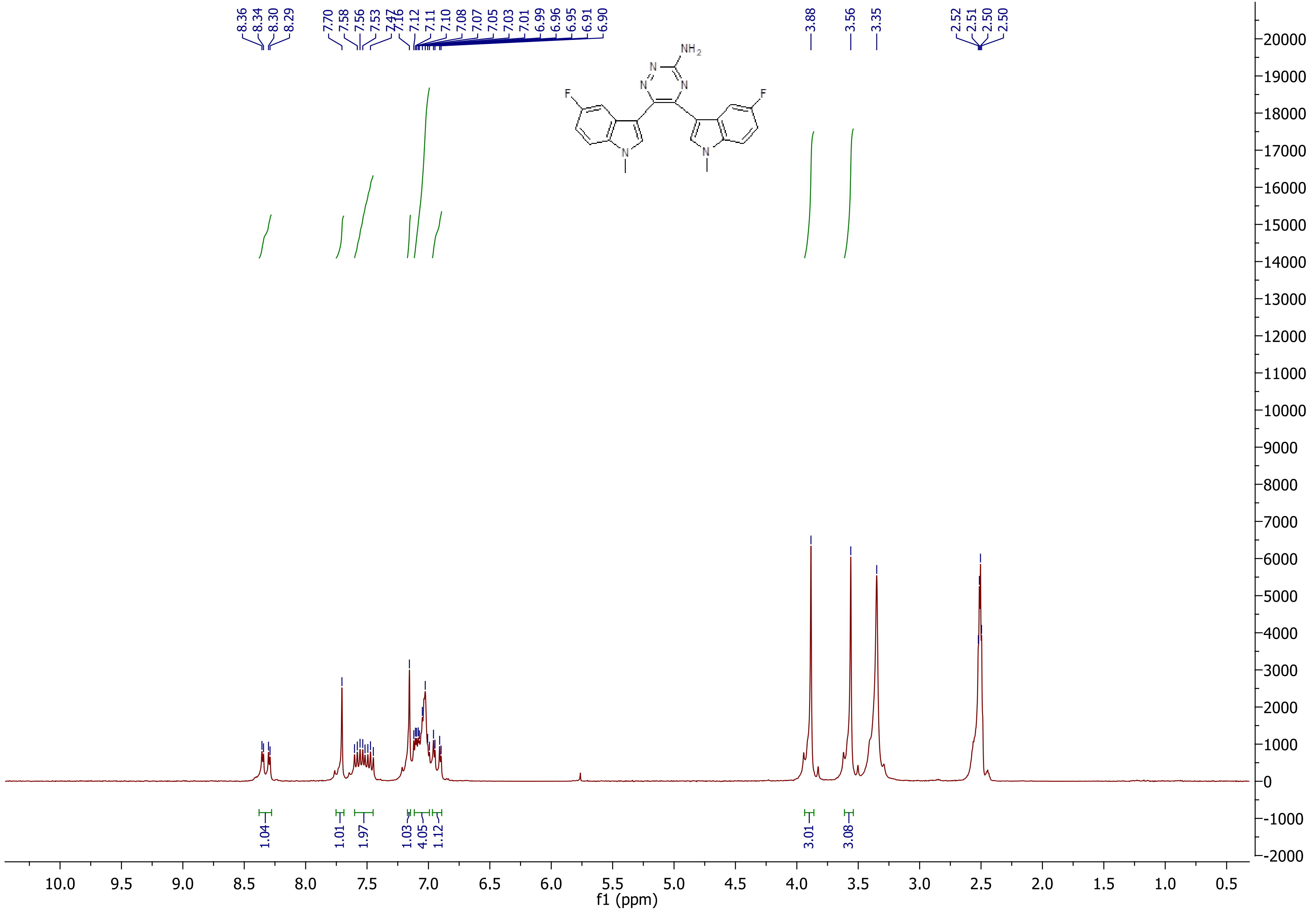

Figure S7: ^{1}H NMR spectrum of compound **5g**

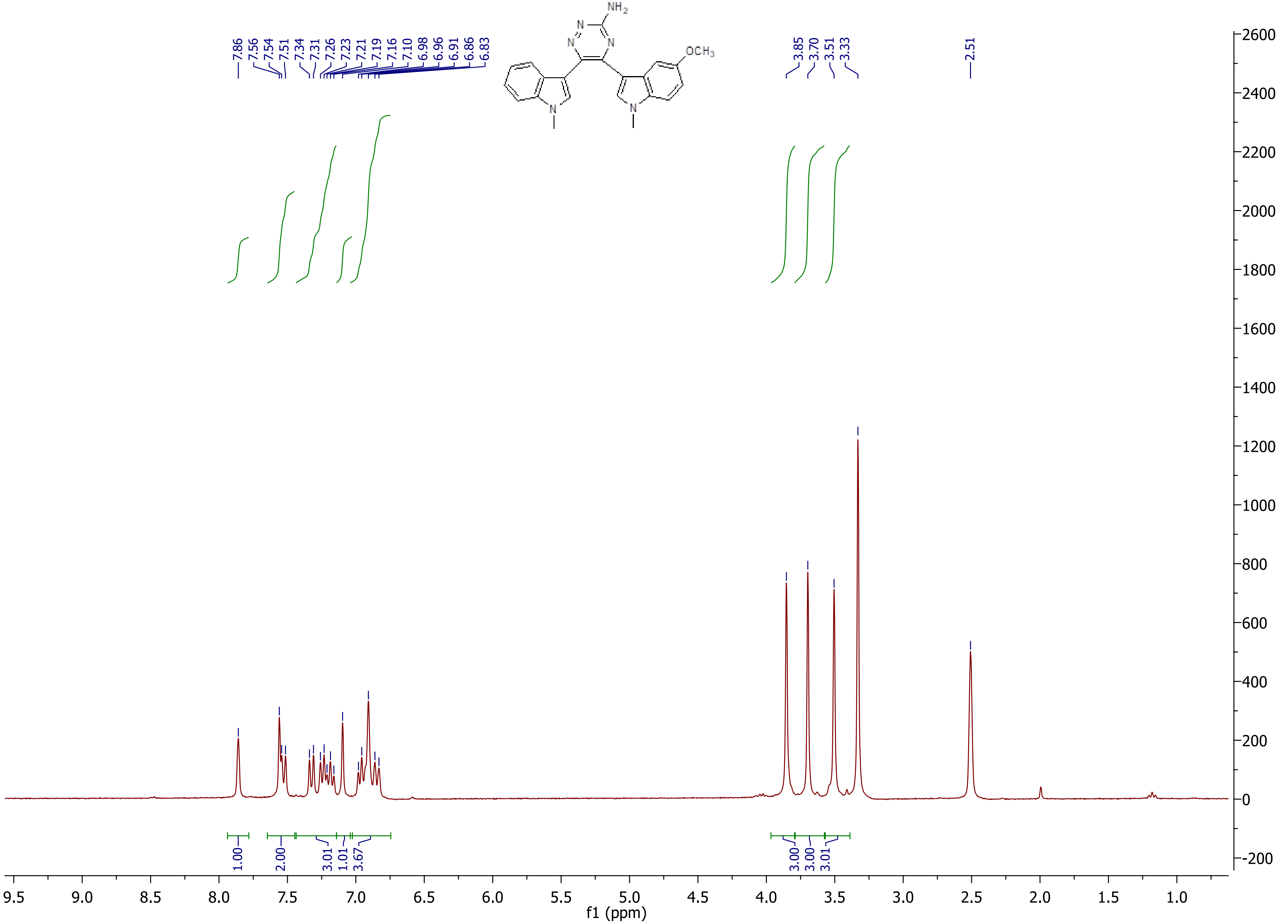

Figure S8: ^{1}H NMR spectrum of compound **5h**

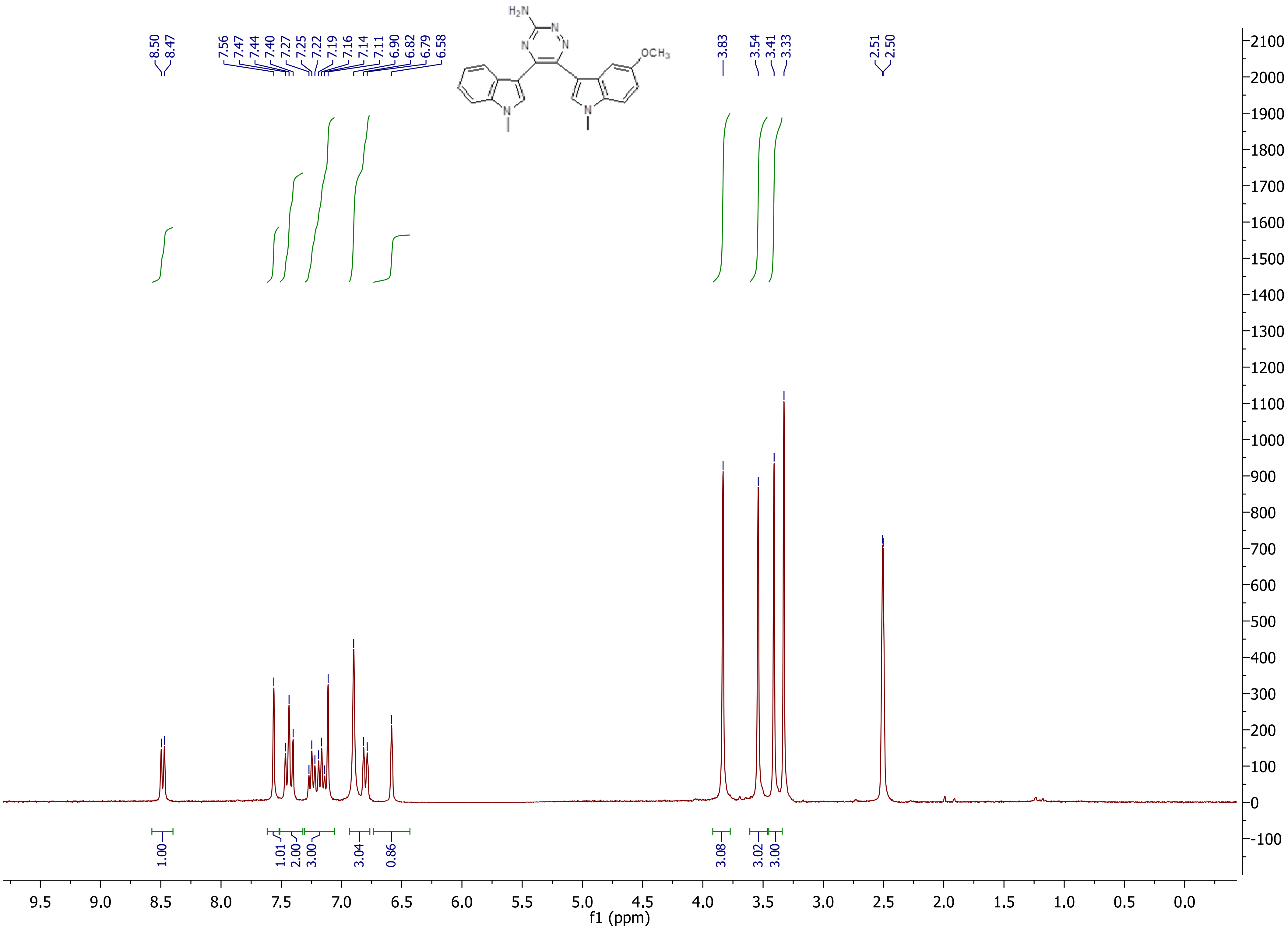

Figure S9: ^{1}H NMR spectrum of compound **6h**

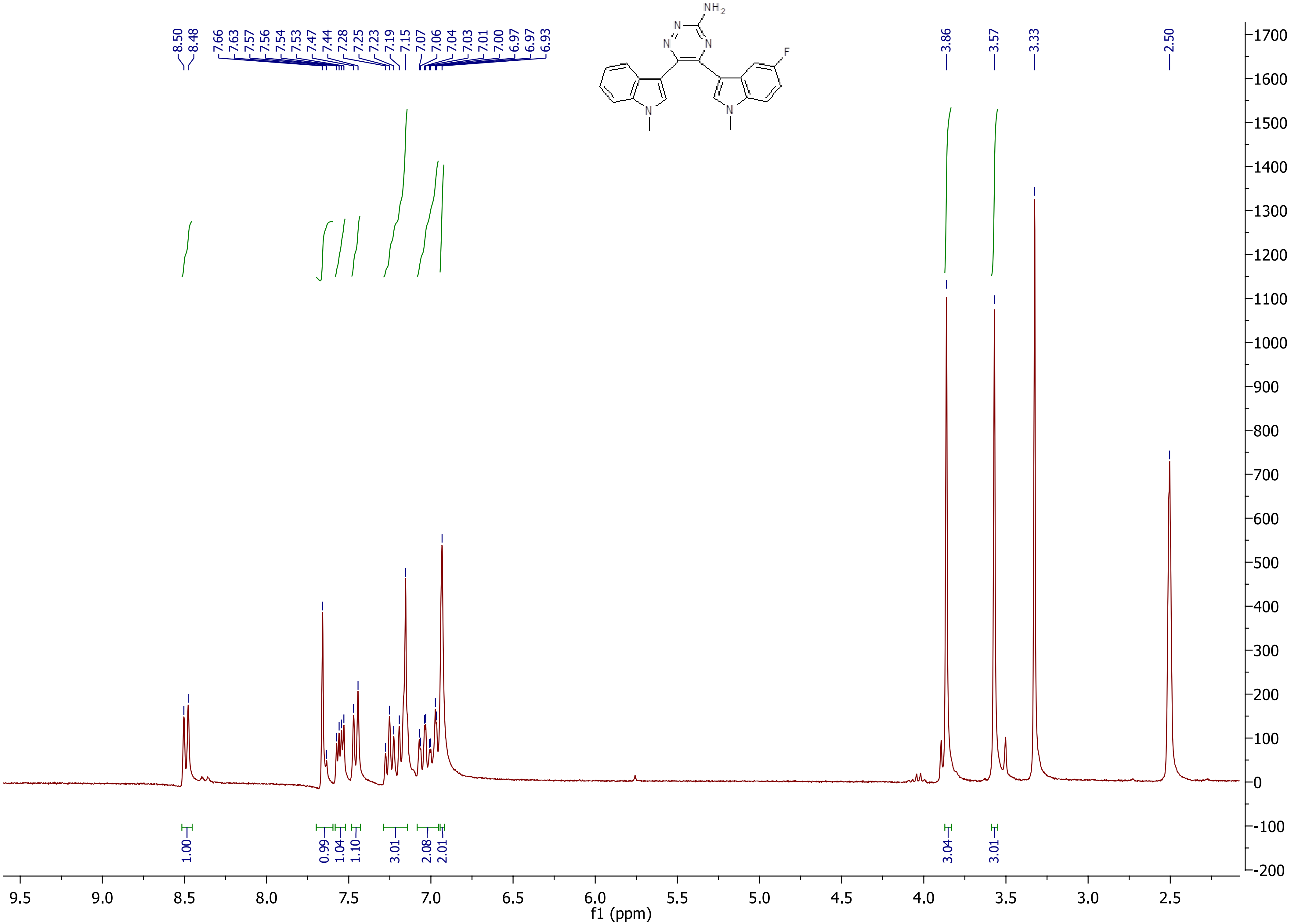

Figure S10: ^{1}H NMR spectrum of compound **5i**

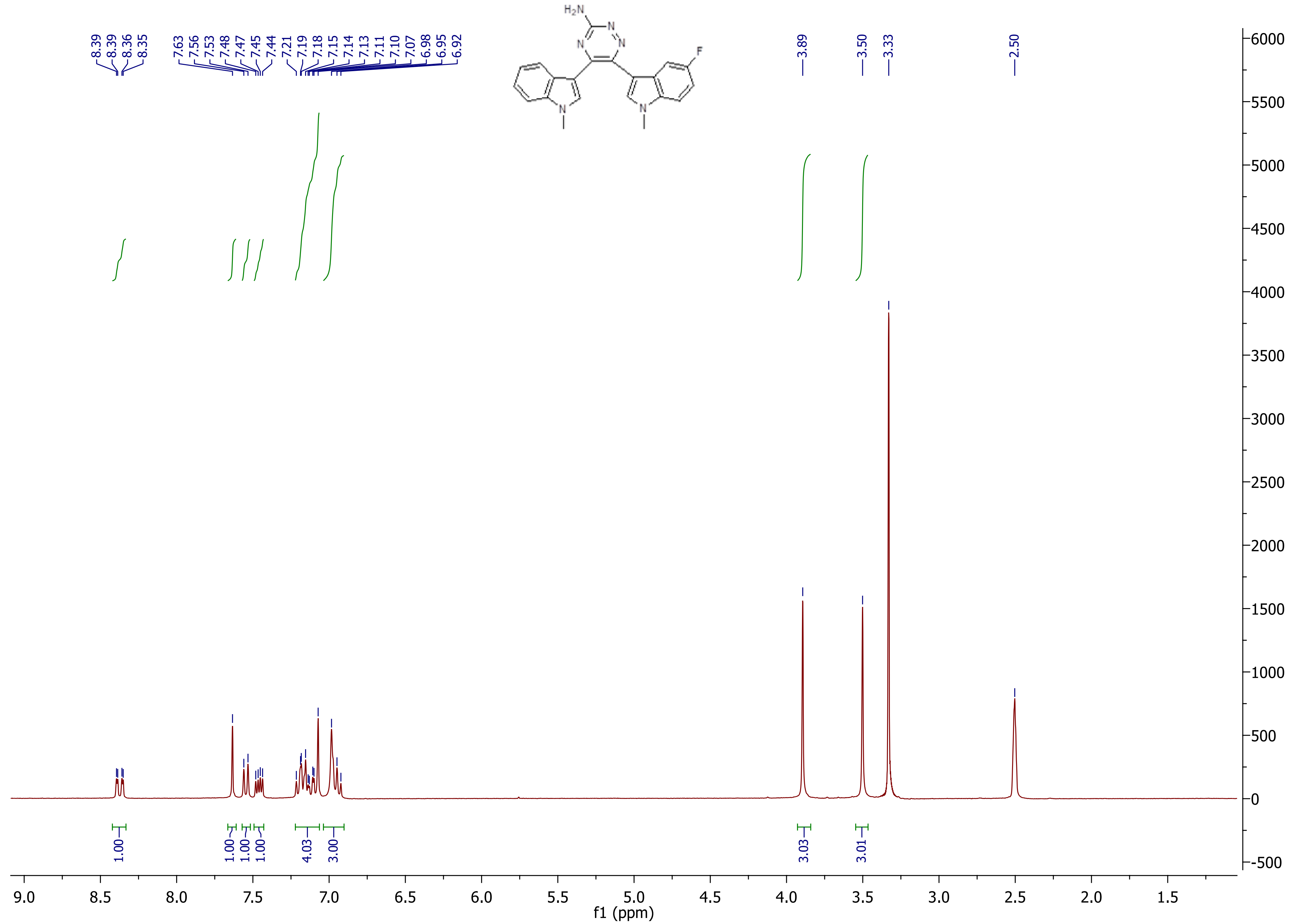

Figure S11: ^{1}H NMR spectrum of compound **6i**

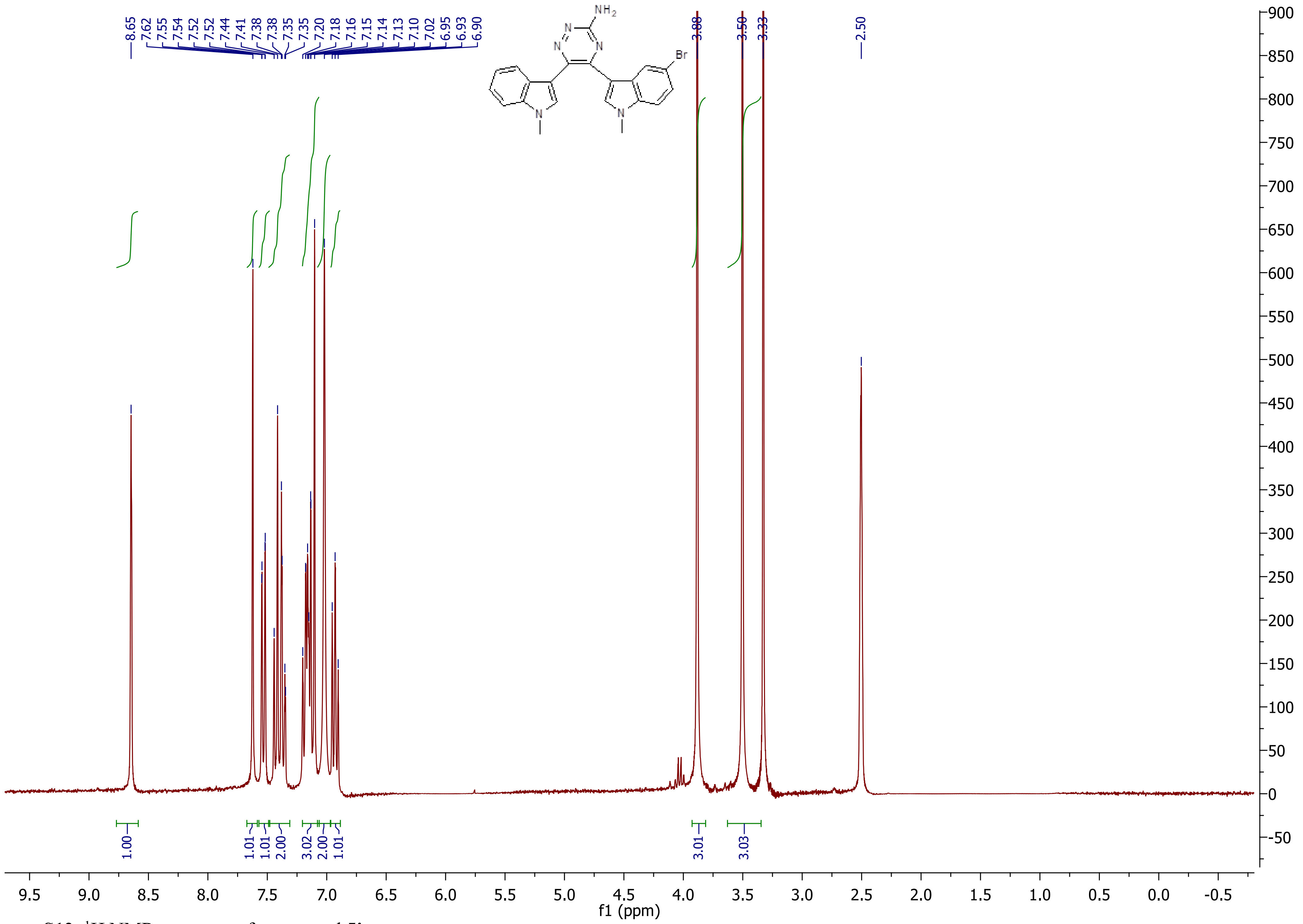

Figure S12: ^{1}H NMR spectrum of compound **5j**

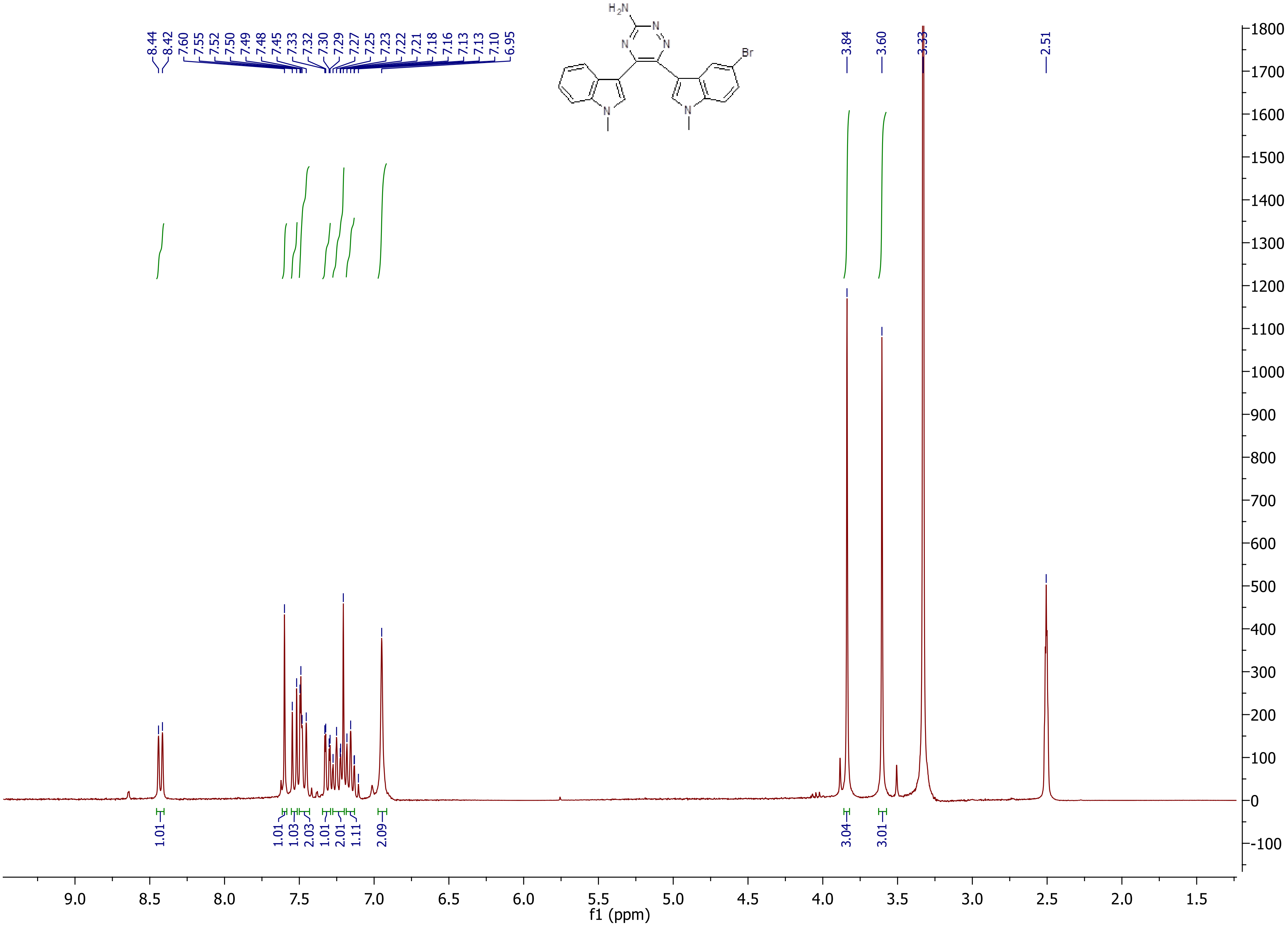

Figure S13: ^{1}H NMR spectrum of compound **6j**

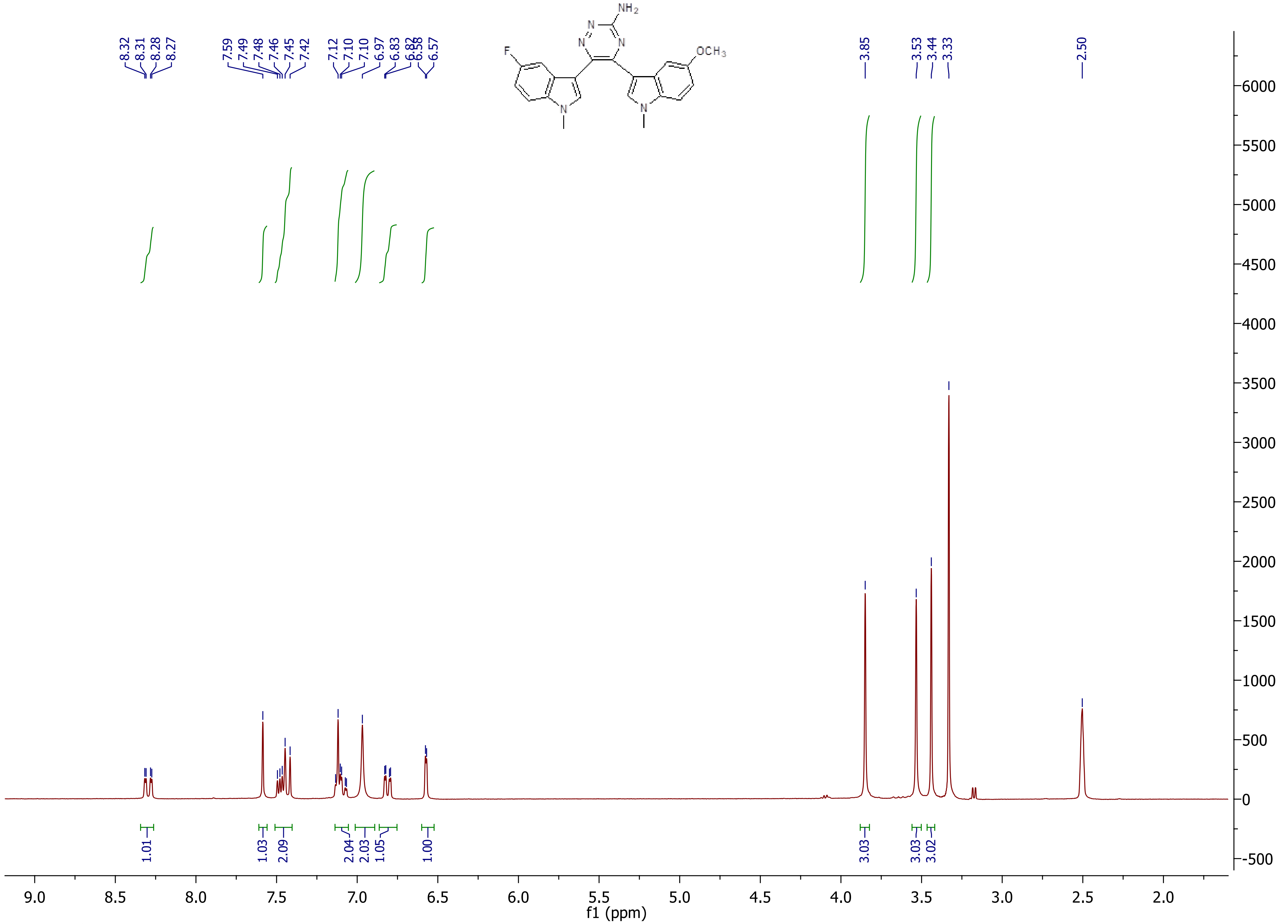

Figure S14: ^{1}H NMR spectrum of compound **5k**

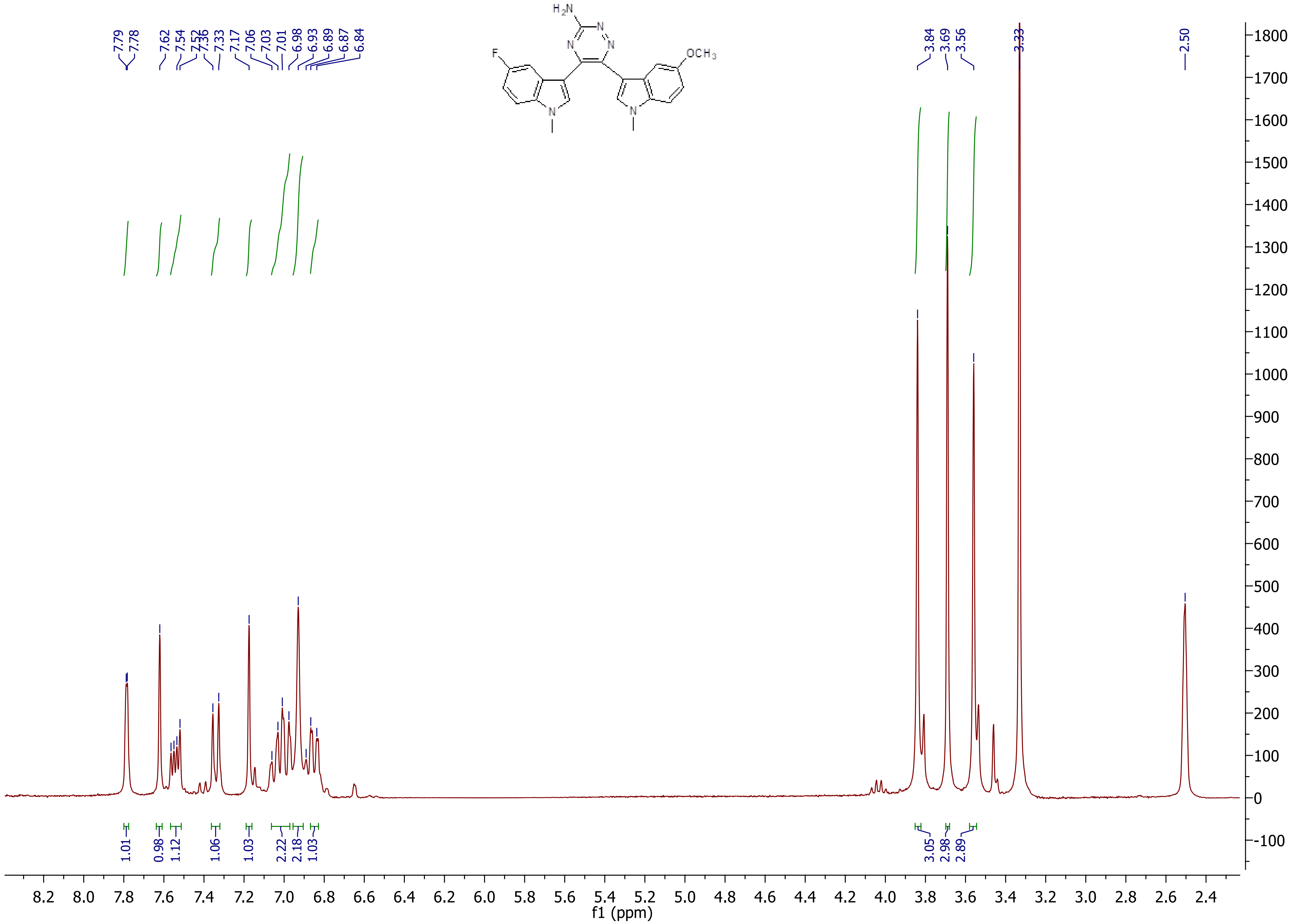

Figure S15: ^{1}H NMR spectrum of compound **6k**

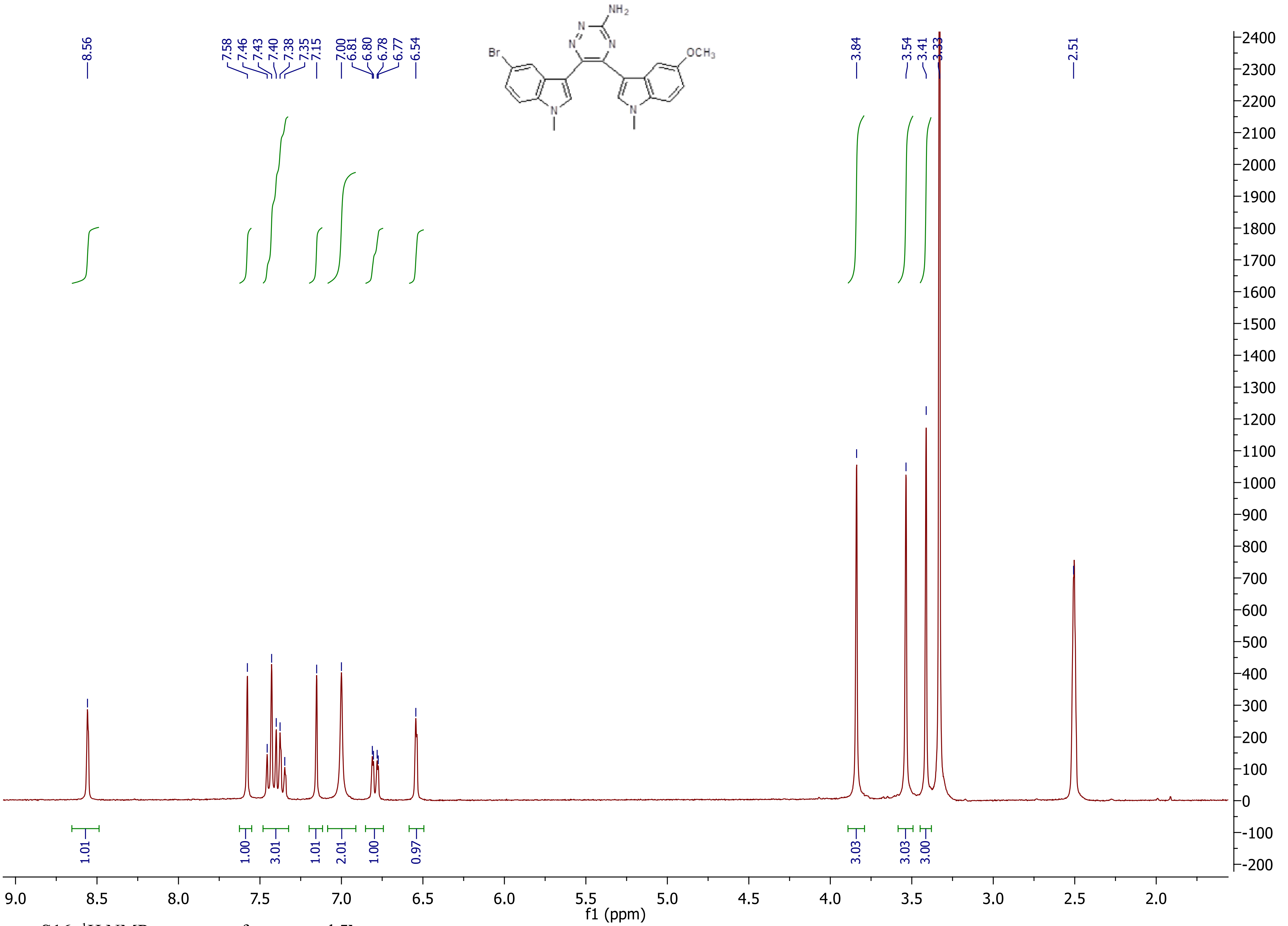

Figure S16: ^{1}H NMR spectrum of compound **5l**

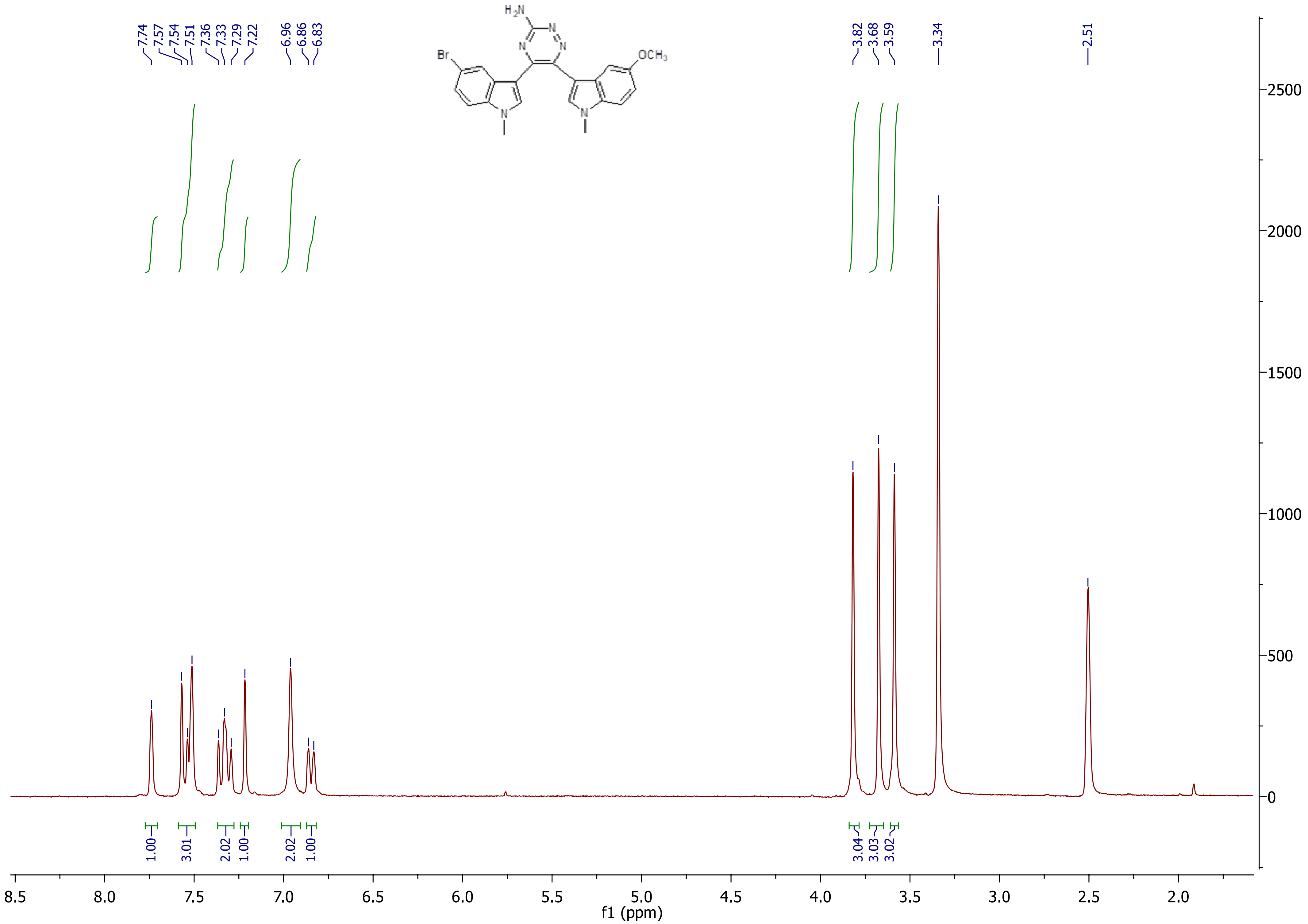

Figure S17: ^{1}H NMR spectrum of compound **6l**

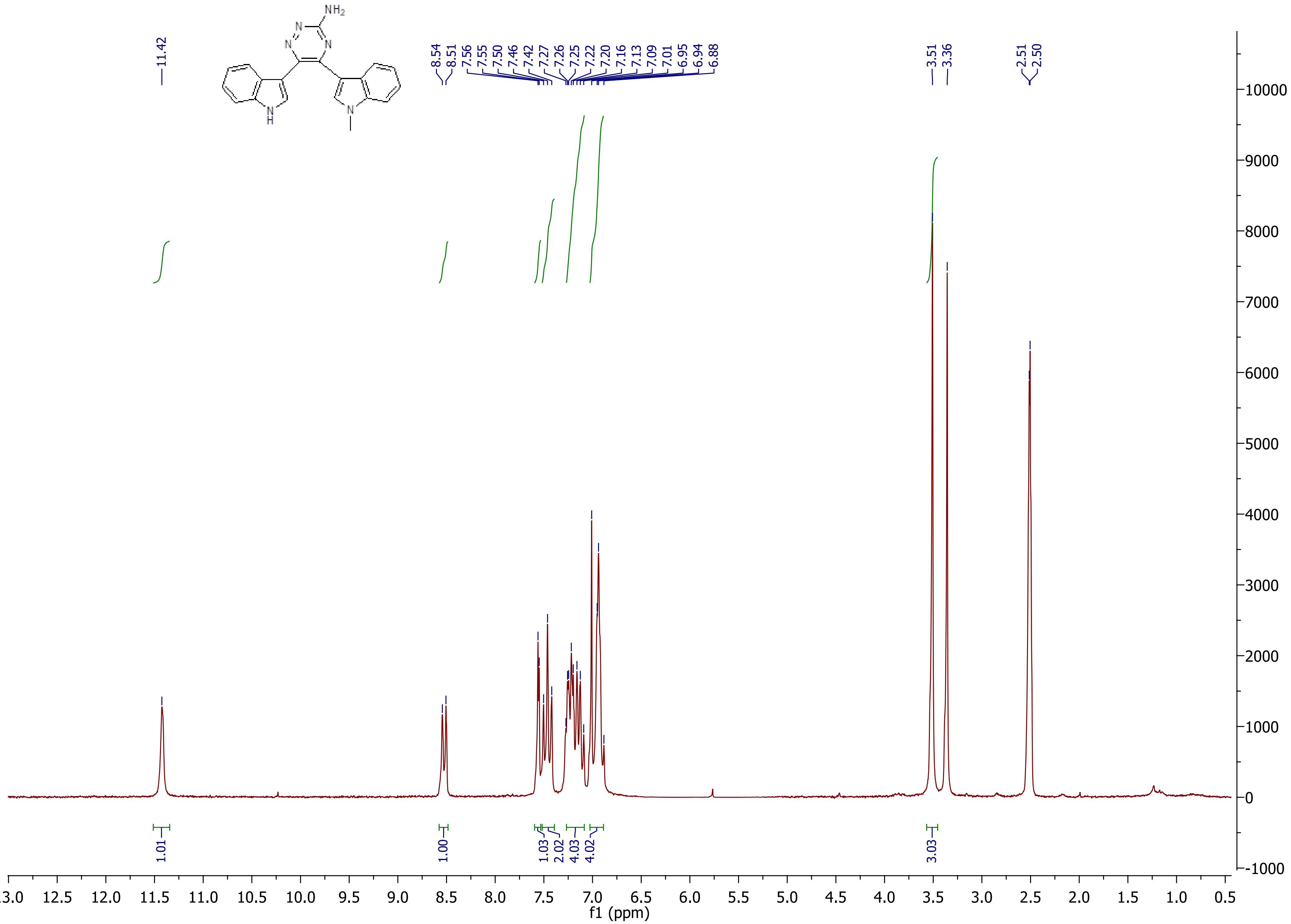

Figure S18: ^{1}H NMR spectrum of compound **5m**

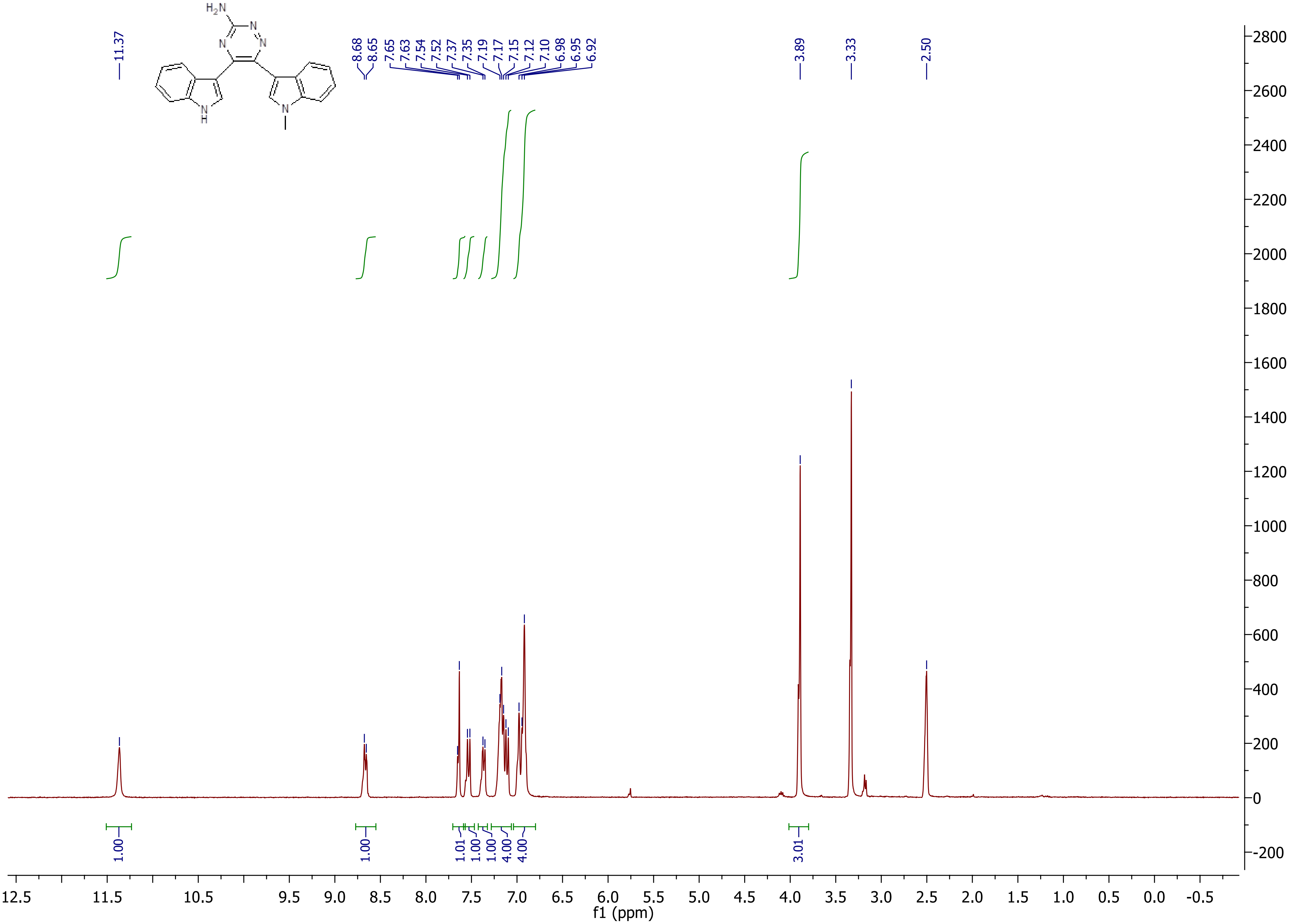

Figure S19: ^{1}H NMR spectrum of compound **6m**

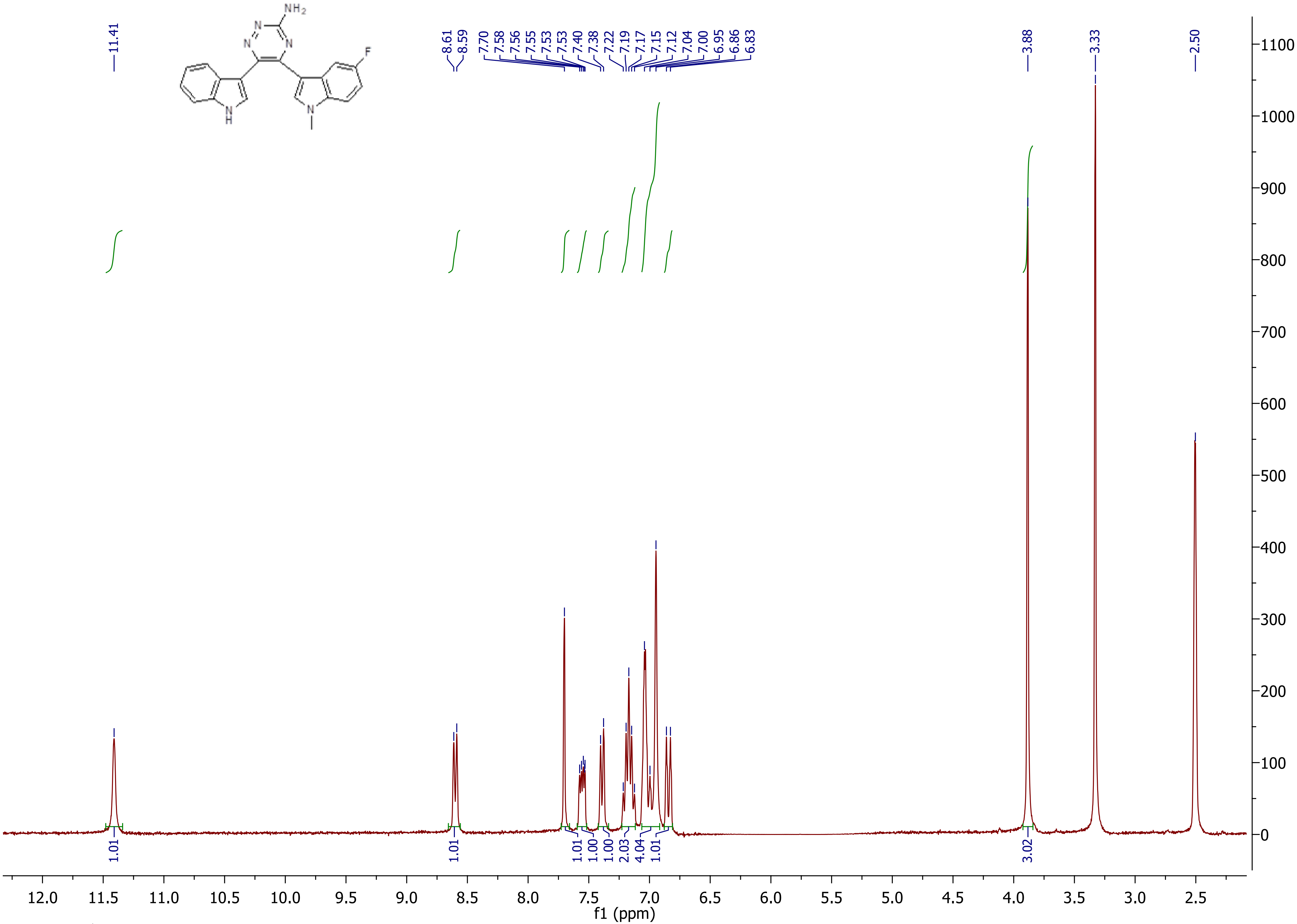

Figure S20: ^{1}H NMR spectrum of compound **5n**

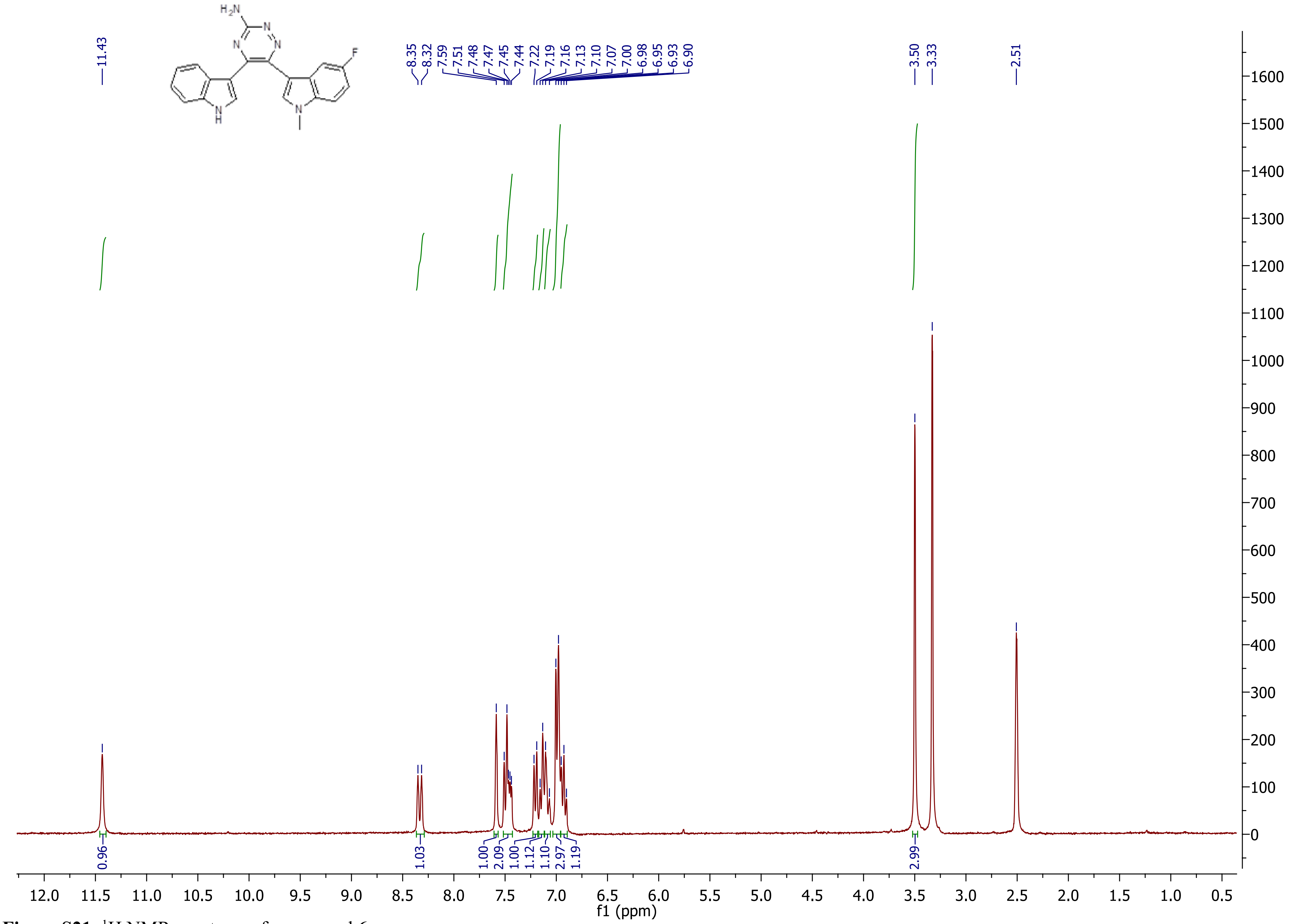

Figure S21: ^{1}H NMR spectrum of compound **6n**

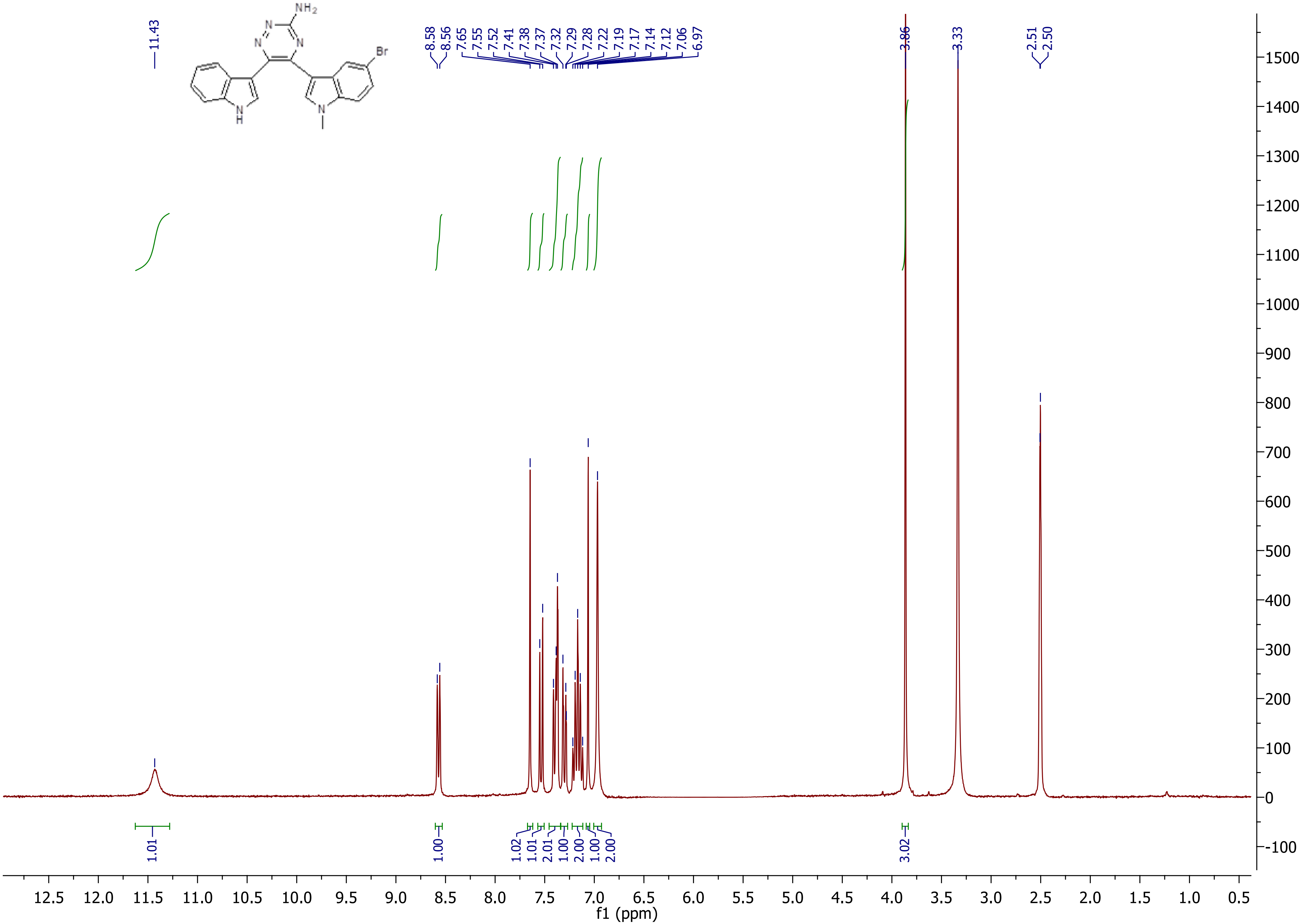

Figure S22: ^{1}H NMR spectrum of compound **5o**

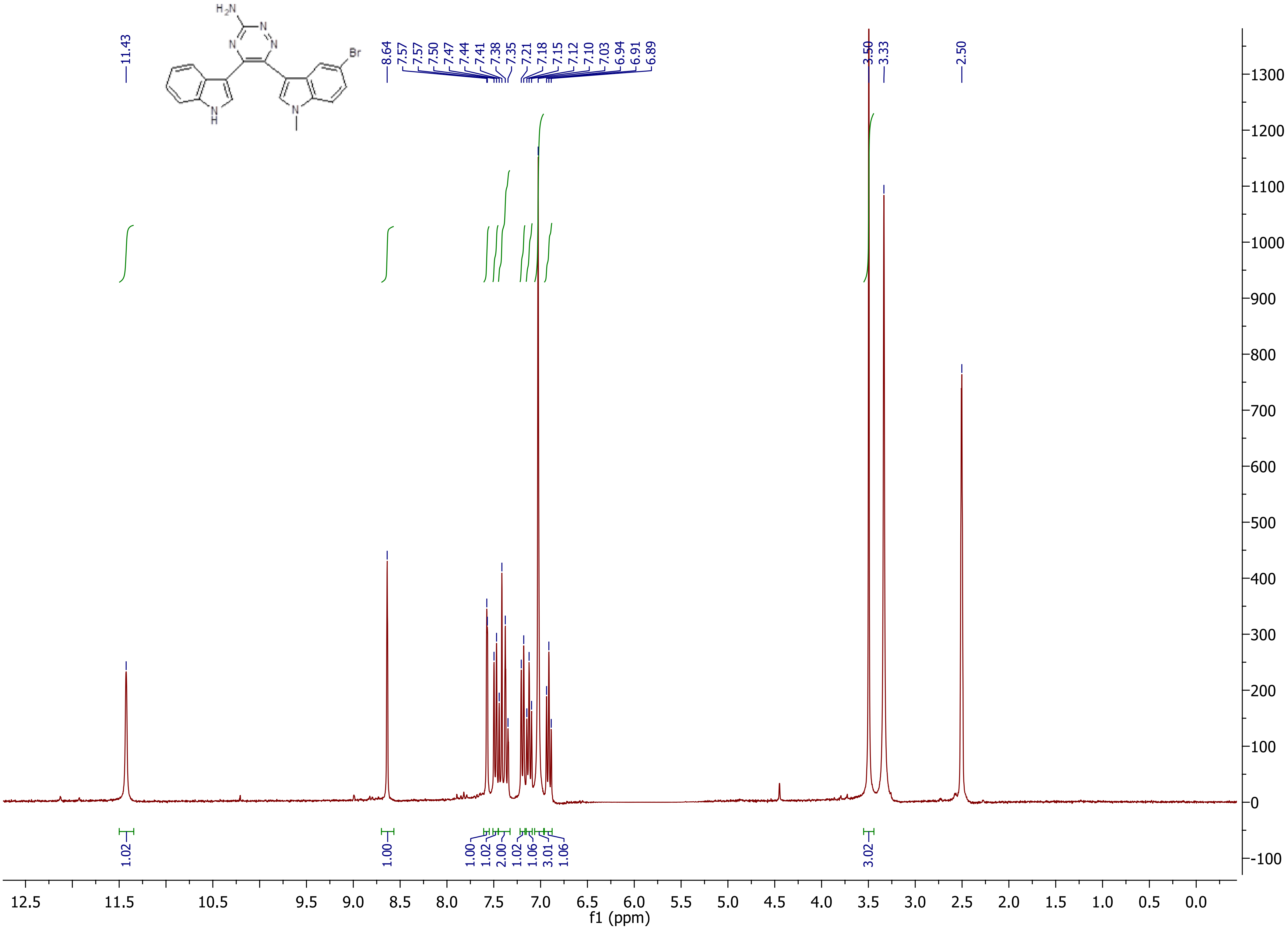

Figure S23: ^{1}H NMR spectrum of compound **6o**

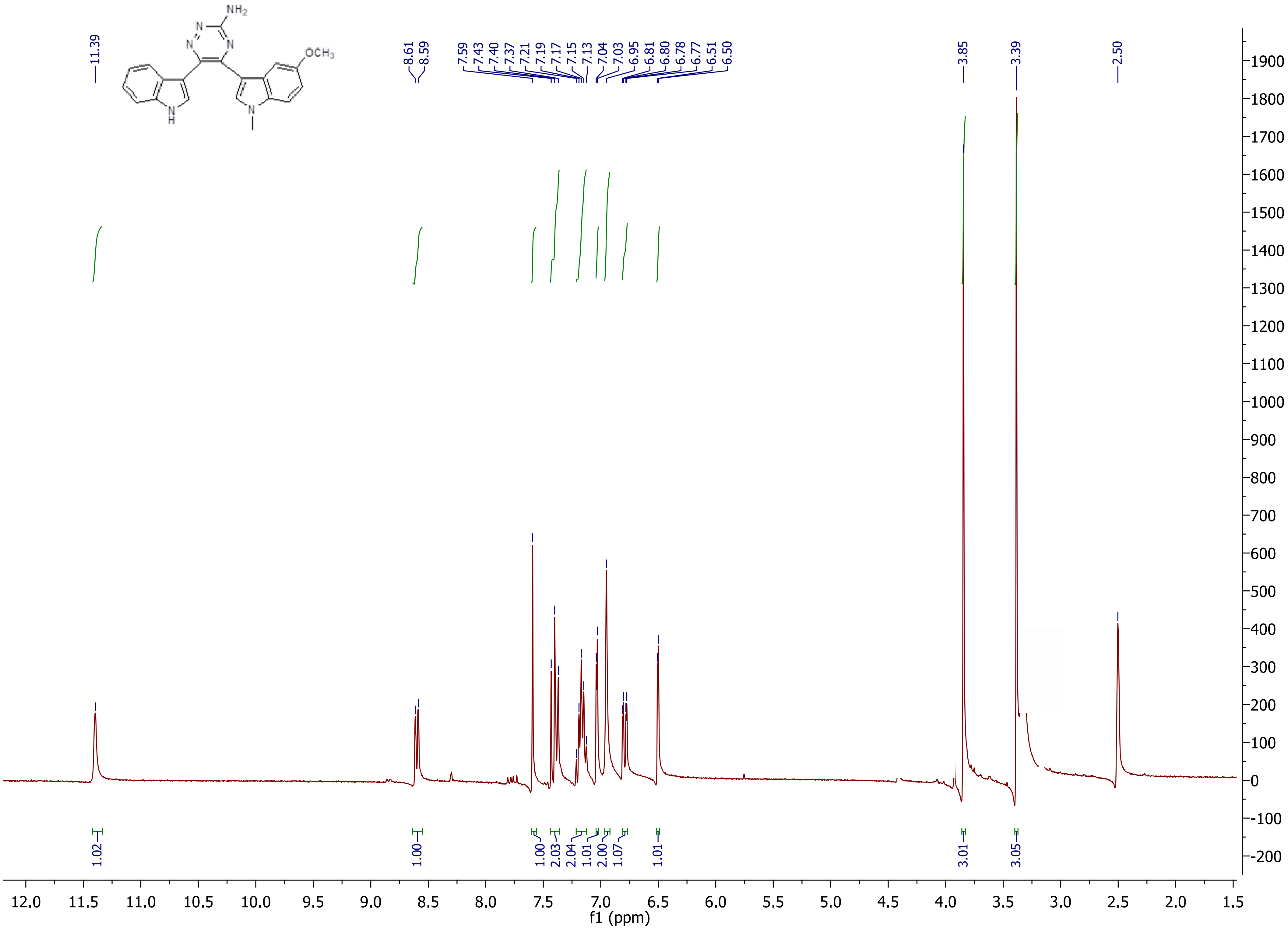

Figure S24: ^{1}H NMR spectrum of compound **5p**

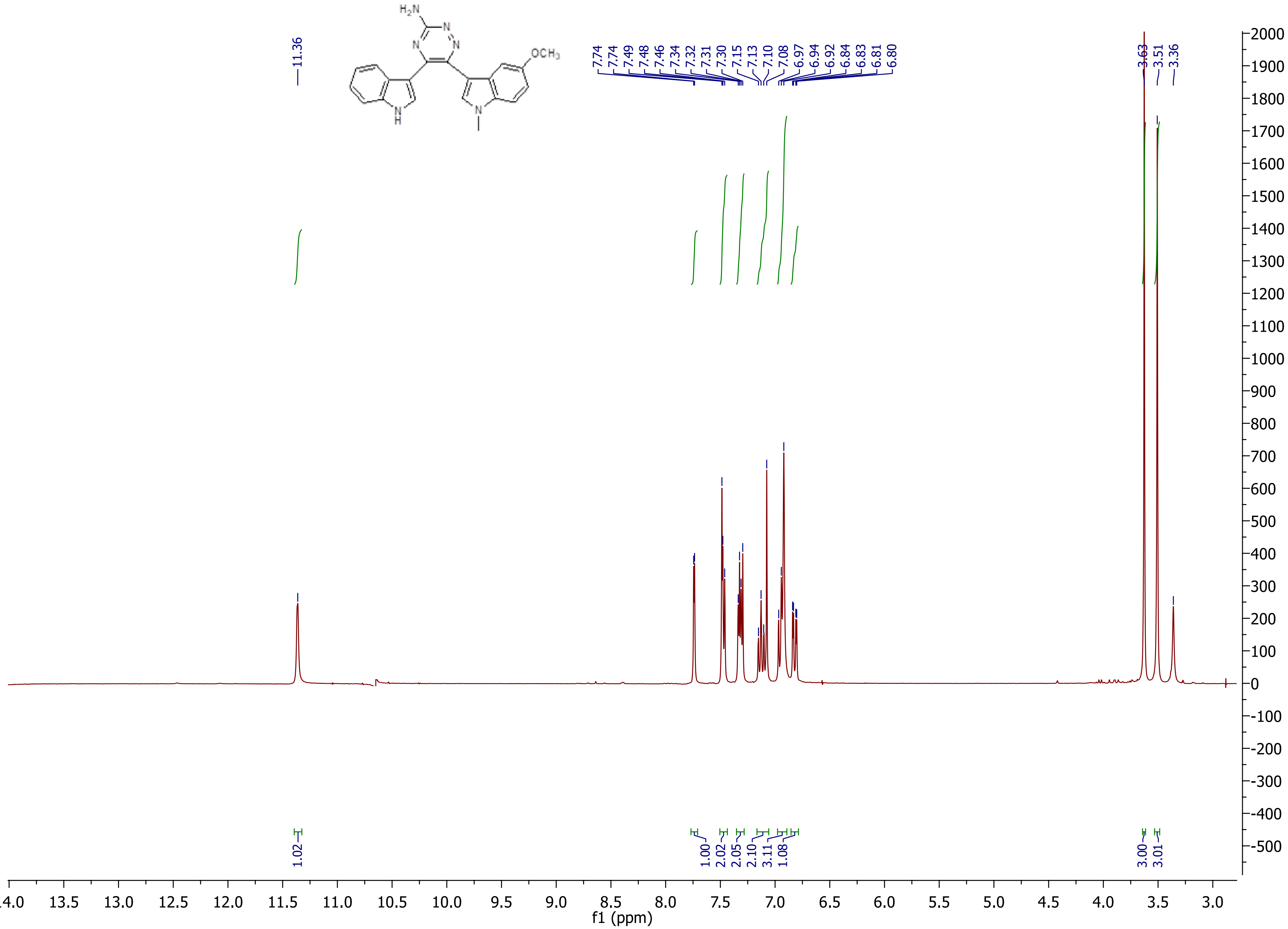

Figure S25: ^{1}H NMR spectrum of compound **6p**

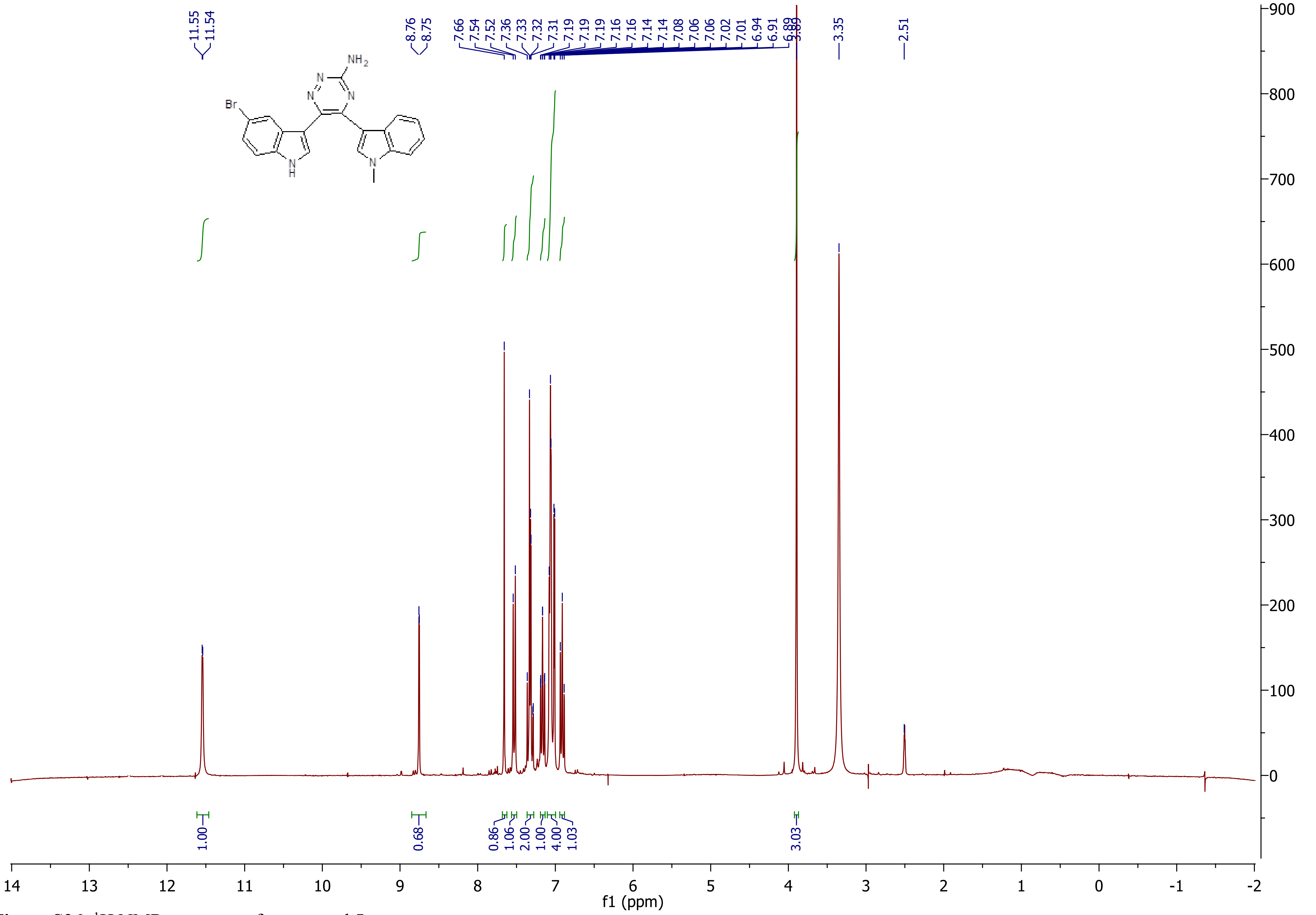

Figure S26: ^{1}H NMR spectrum of compound **5q**

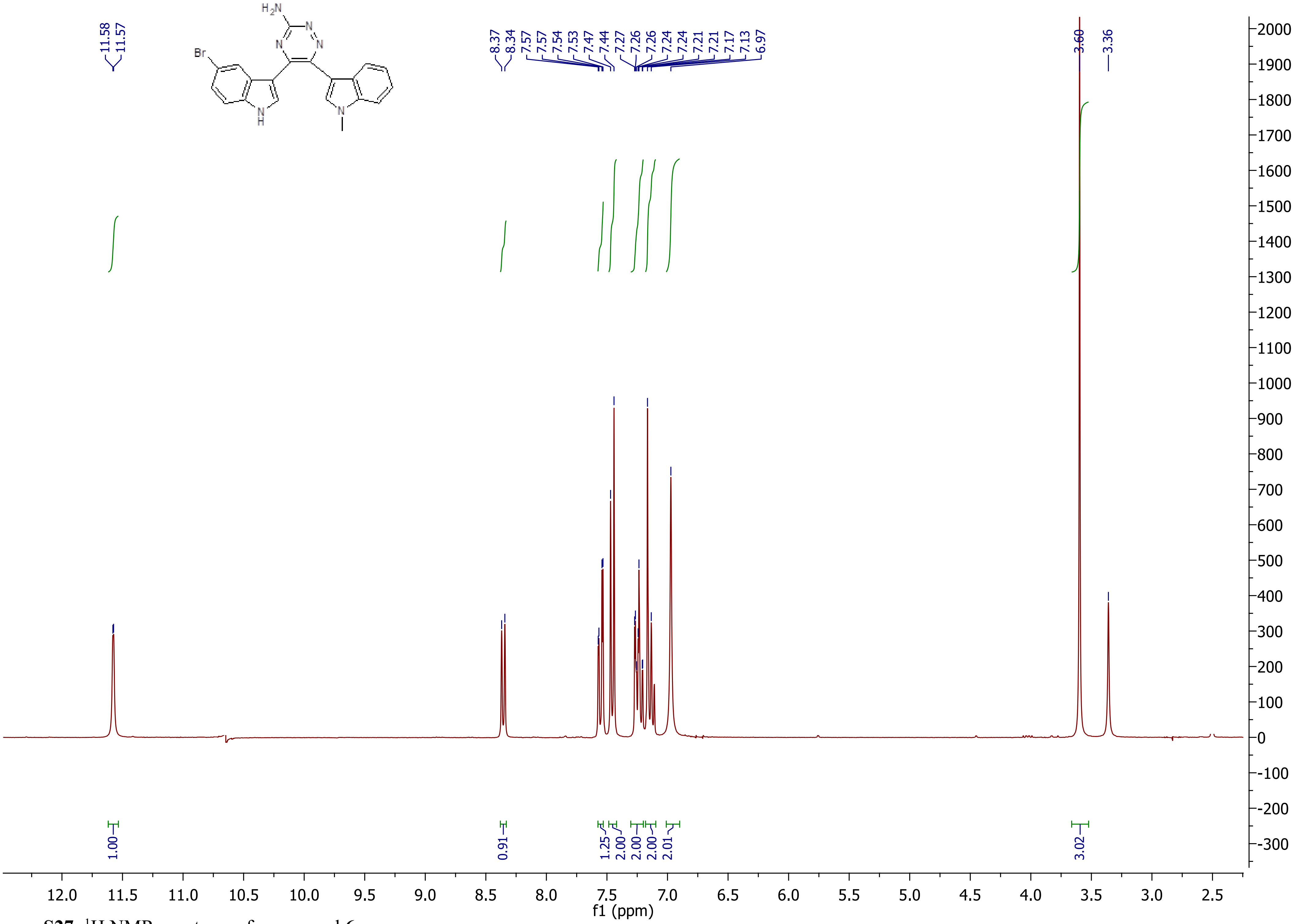

Figure S27: ^{1}H NMR spectrum of compound **6q**

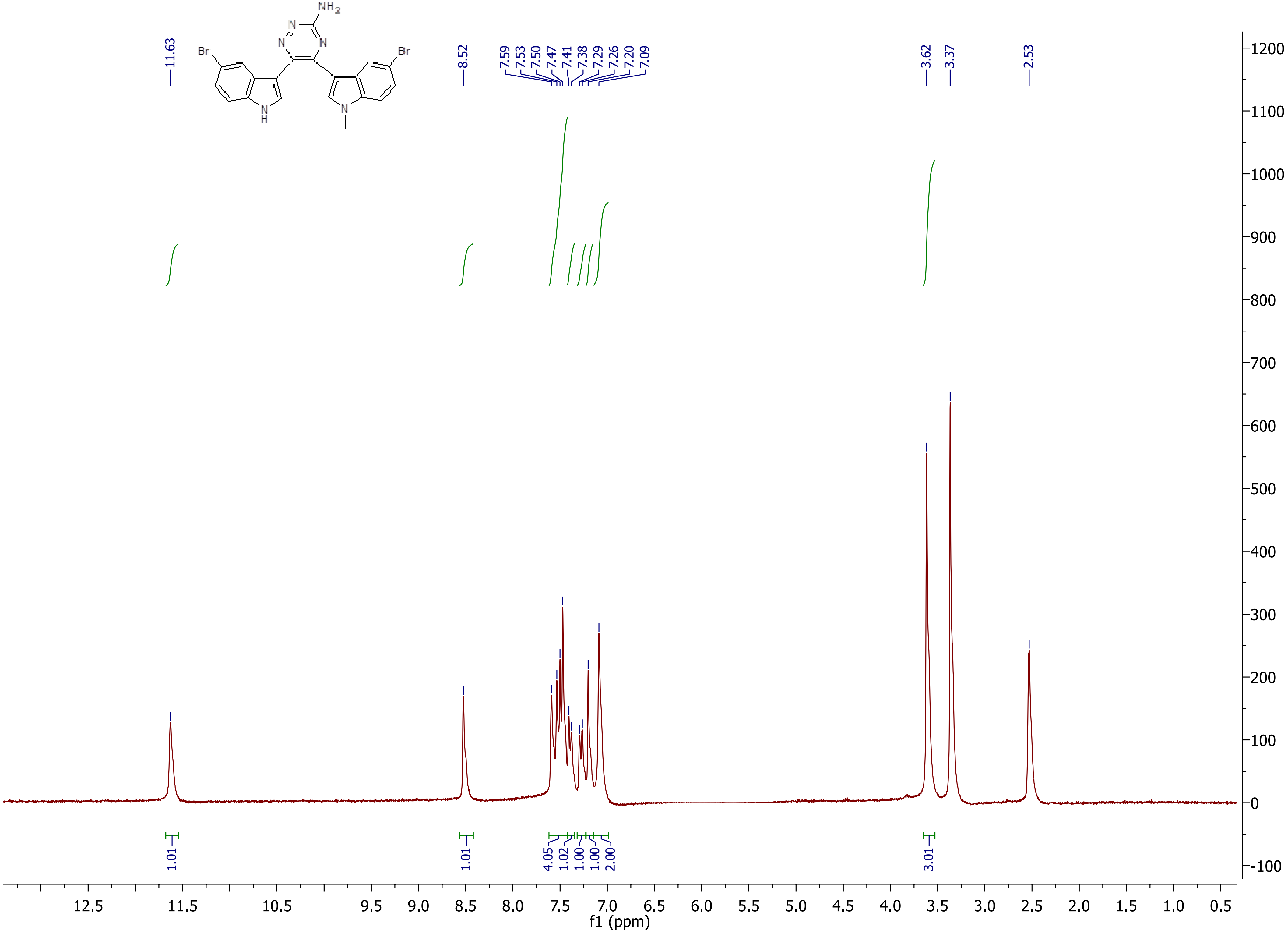

Figure S28: ^{1}H NMR spectrum of compound **5r**

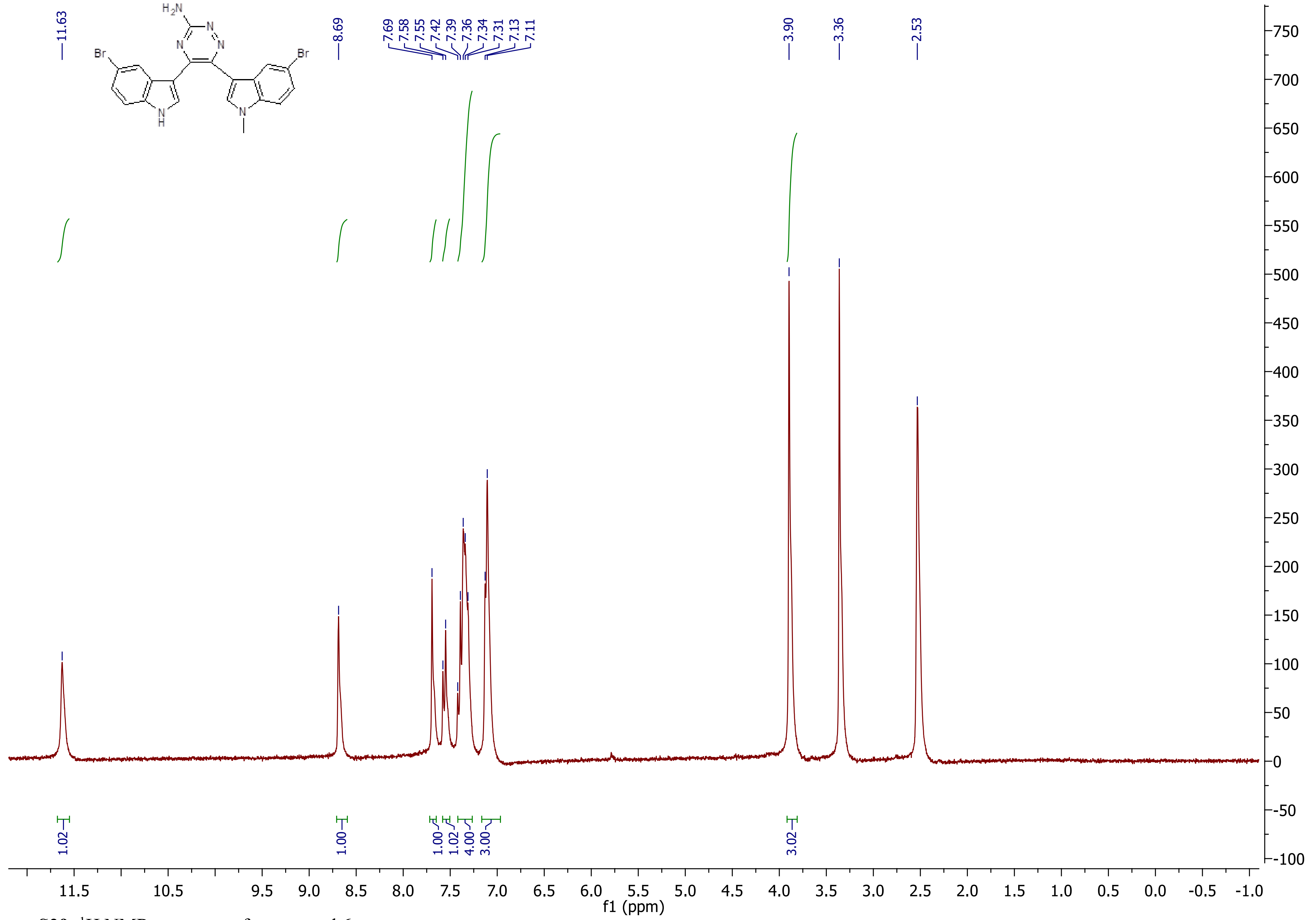

Figure S29: ^{1}H NMR spectrum of compound **6r**

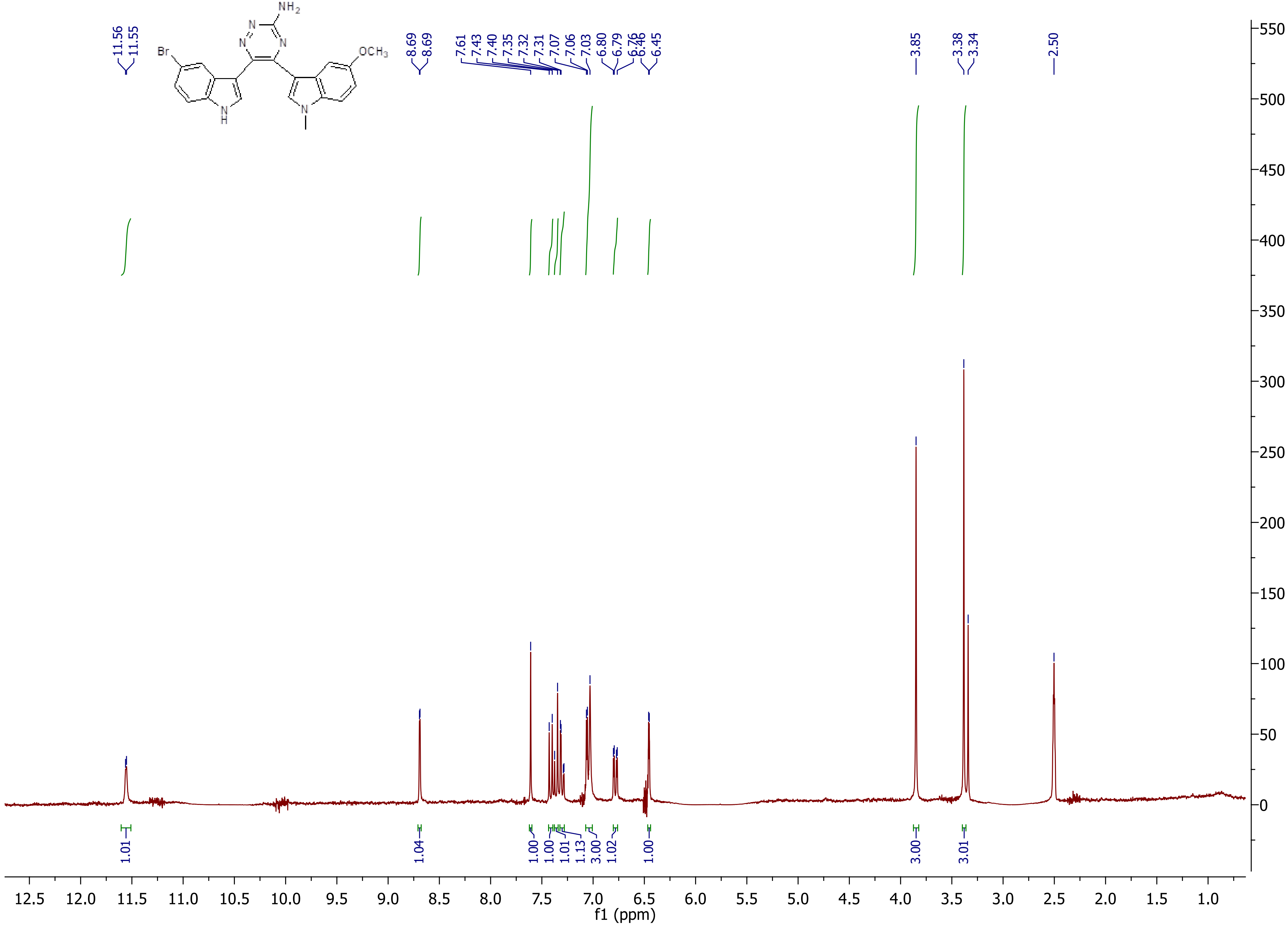

Figure S30: ^{1}H NMR spectrum of compound **5s**

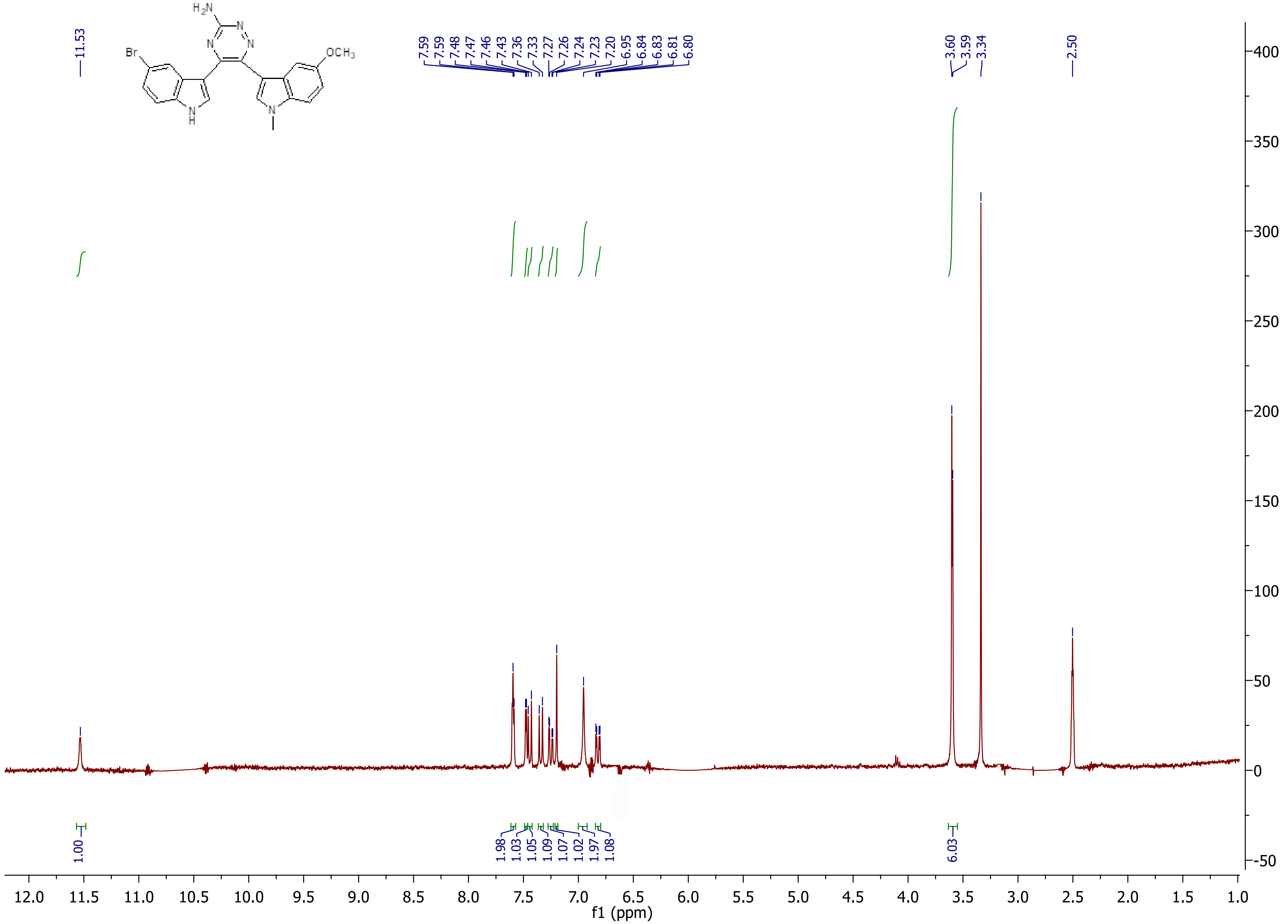

Figure S31: ^{1}H NMR spectrum of compound **6s**

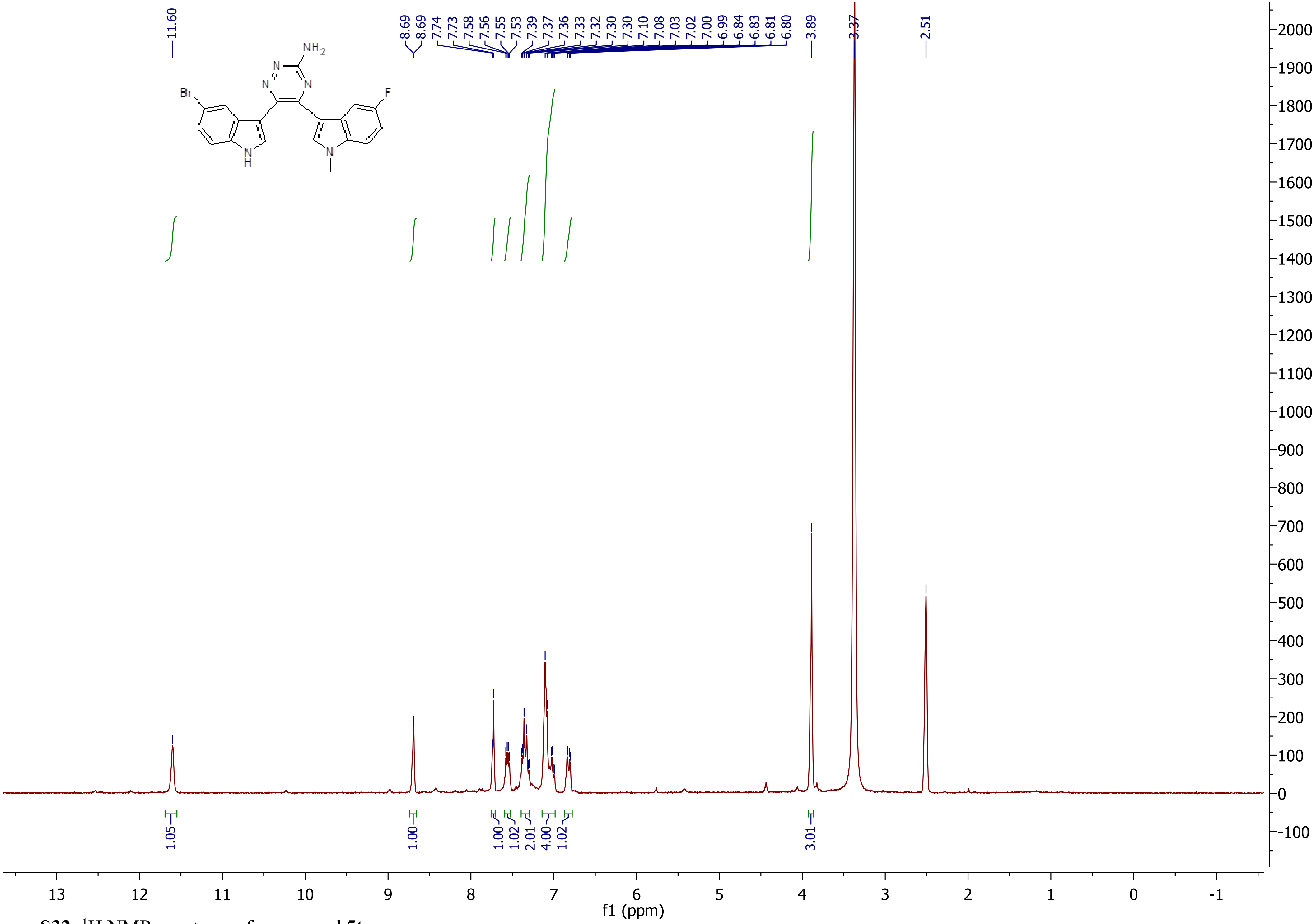

Figure S32: ^{1}H NMR spectrum of compound **5t**

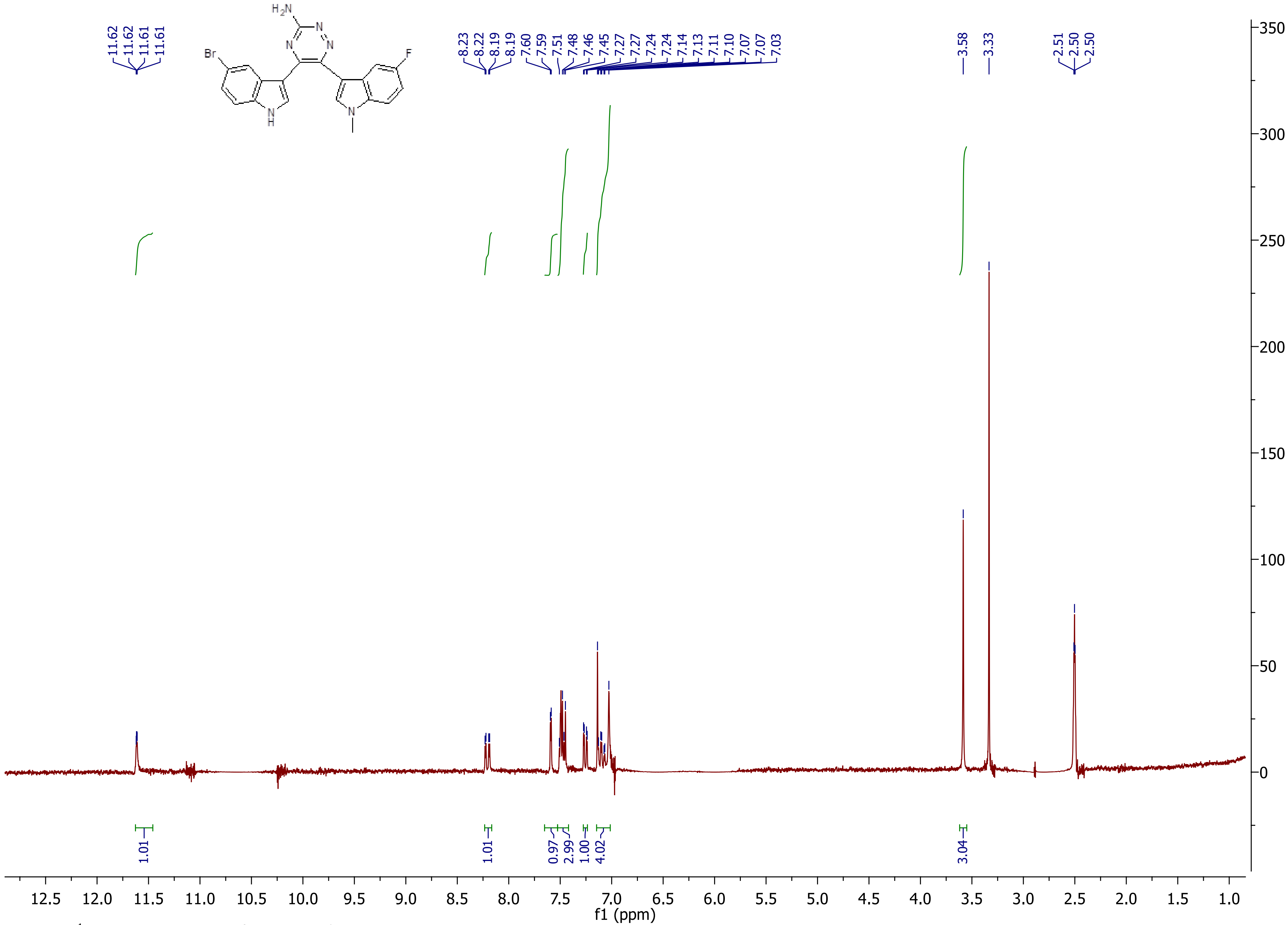

Figure S33: ^{1}H NMR spectrum of compound **6t**

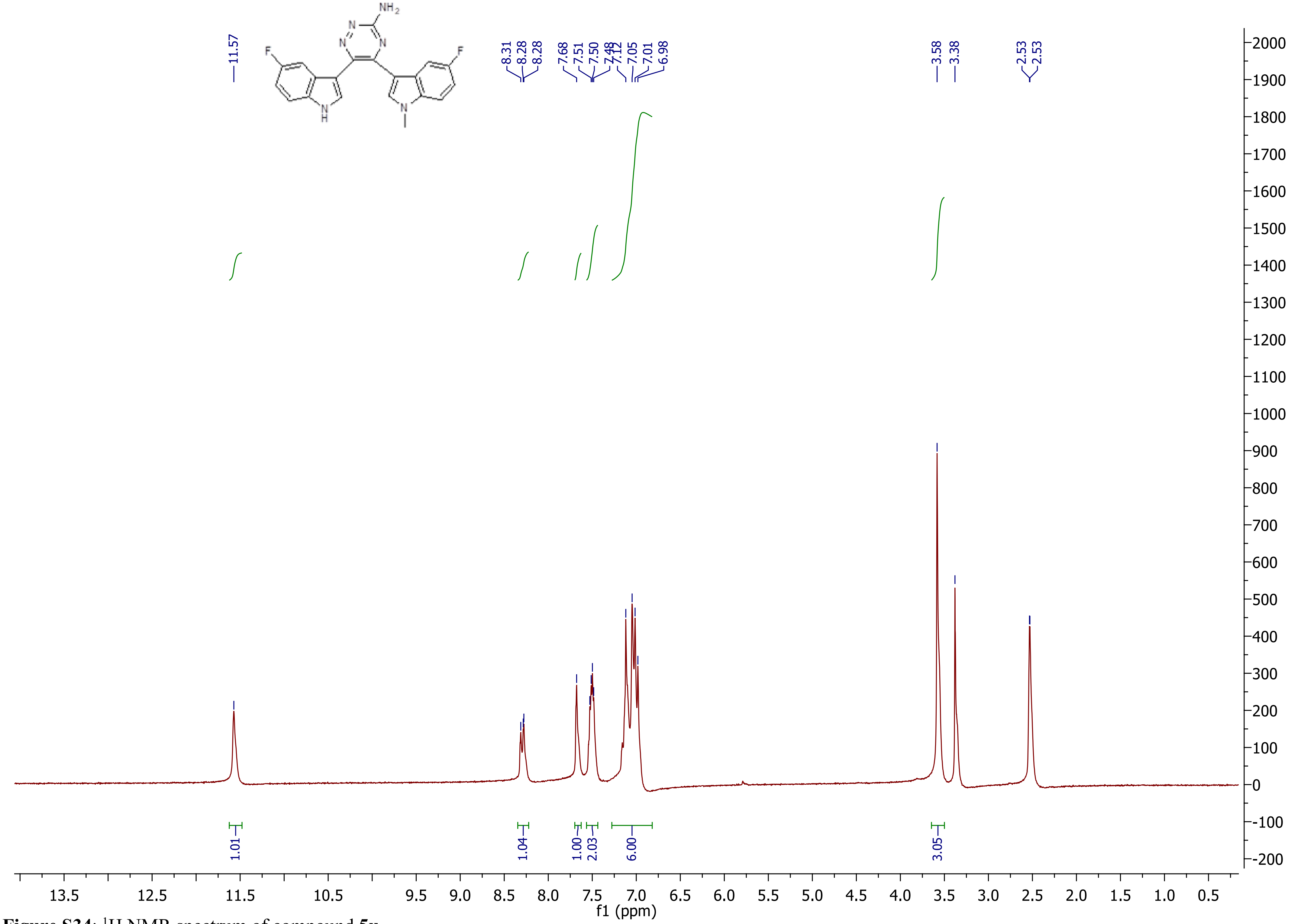

Figure S34: ^{1}H NMR spectrum of compound **5u**

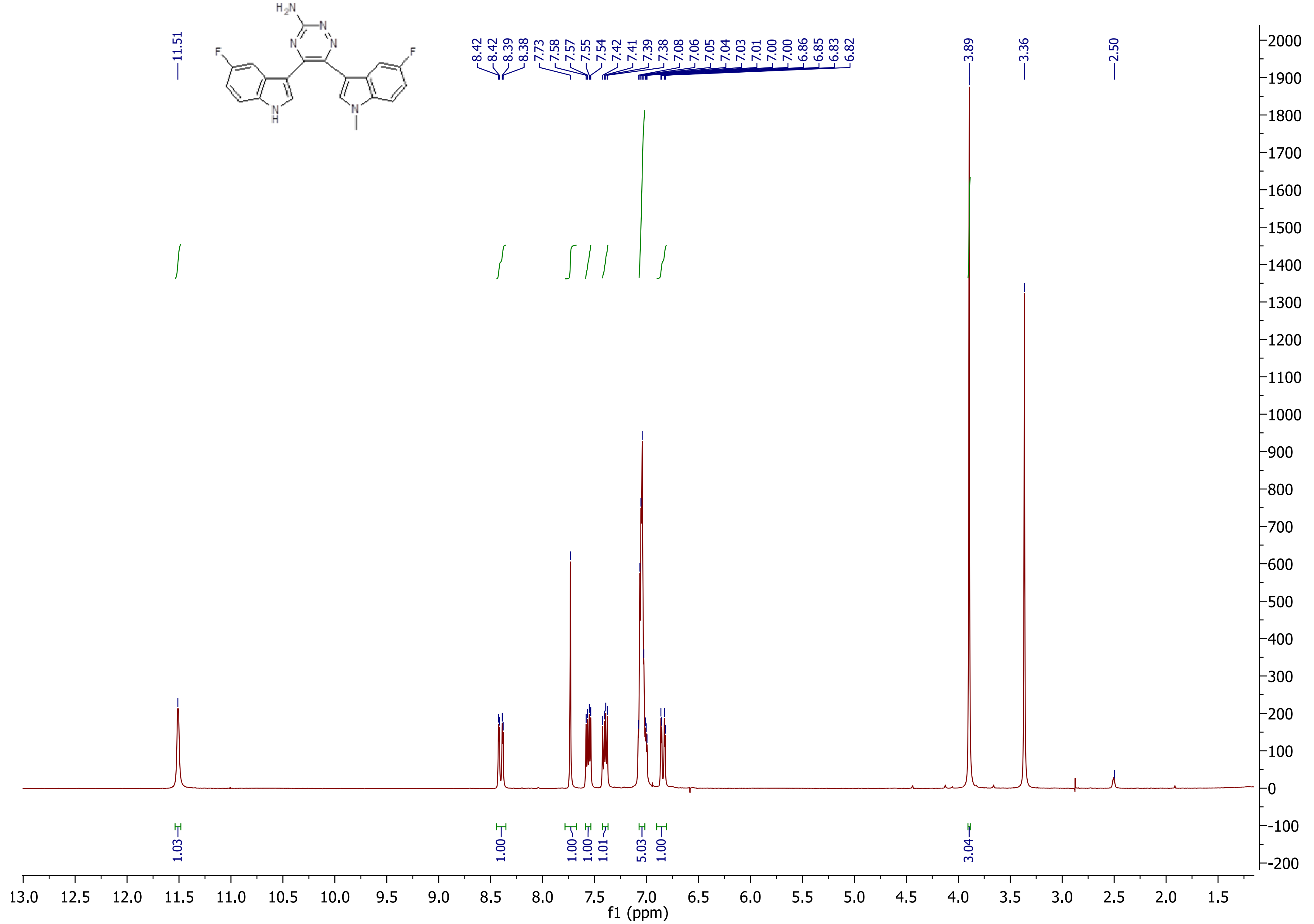

Figure S35: ^{1}H NMR spectrum of compound **6u**

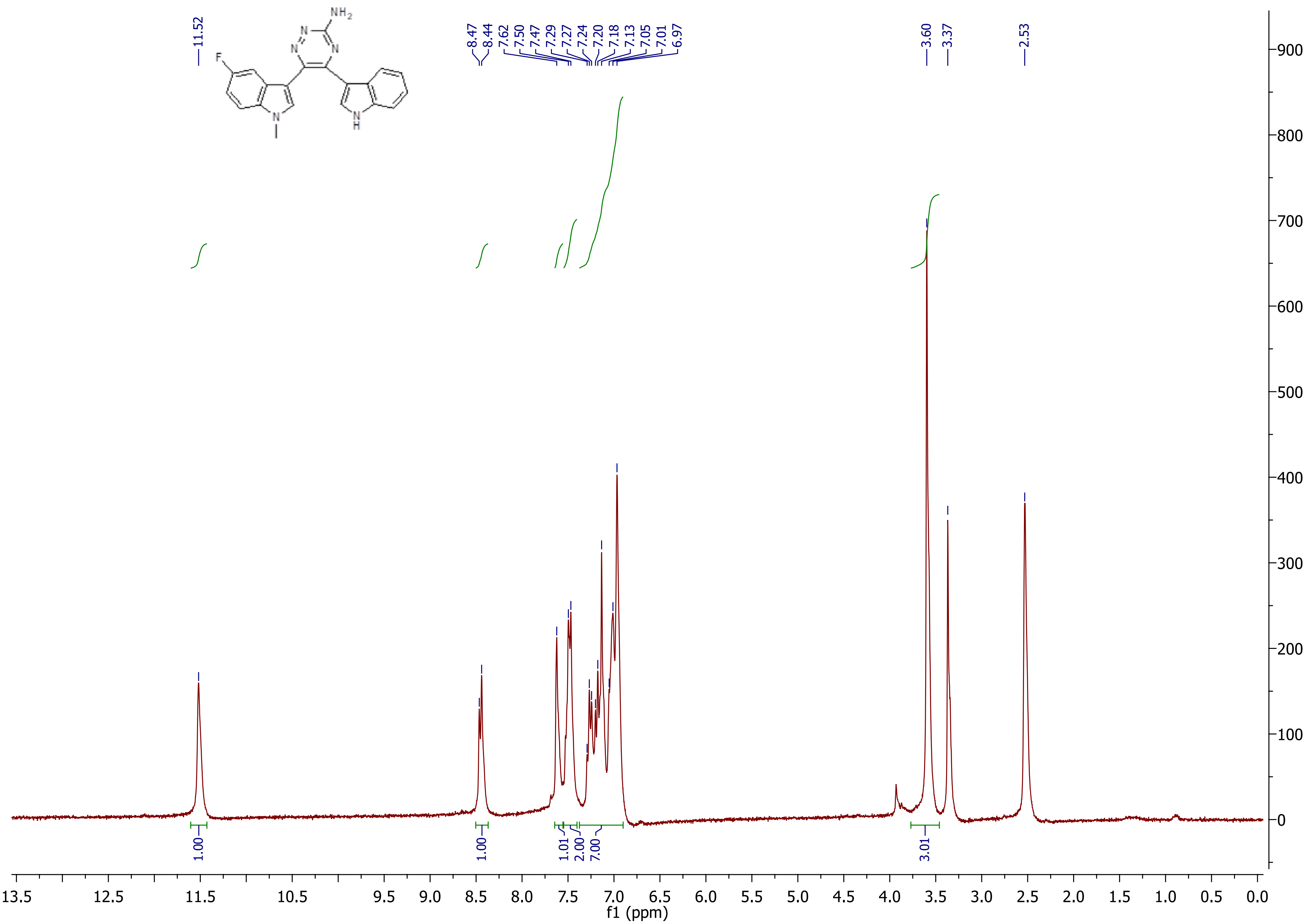

Figure S36 ^{1}H NMR spectrum of compound **5v**

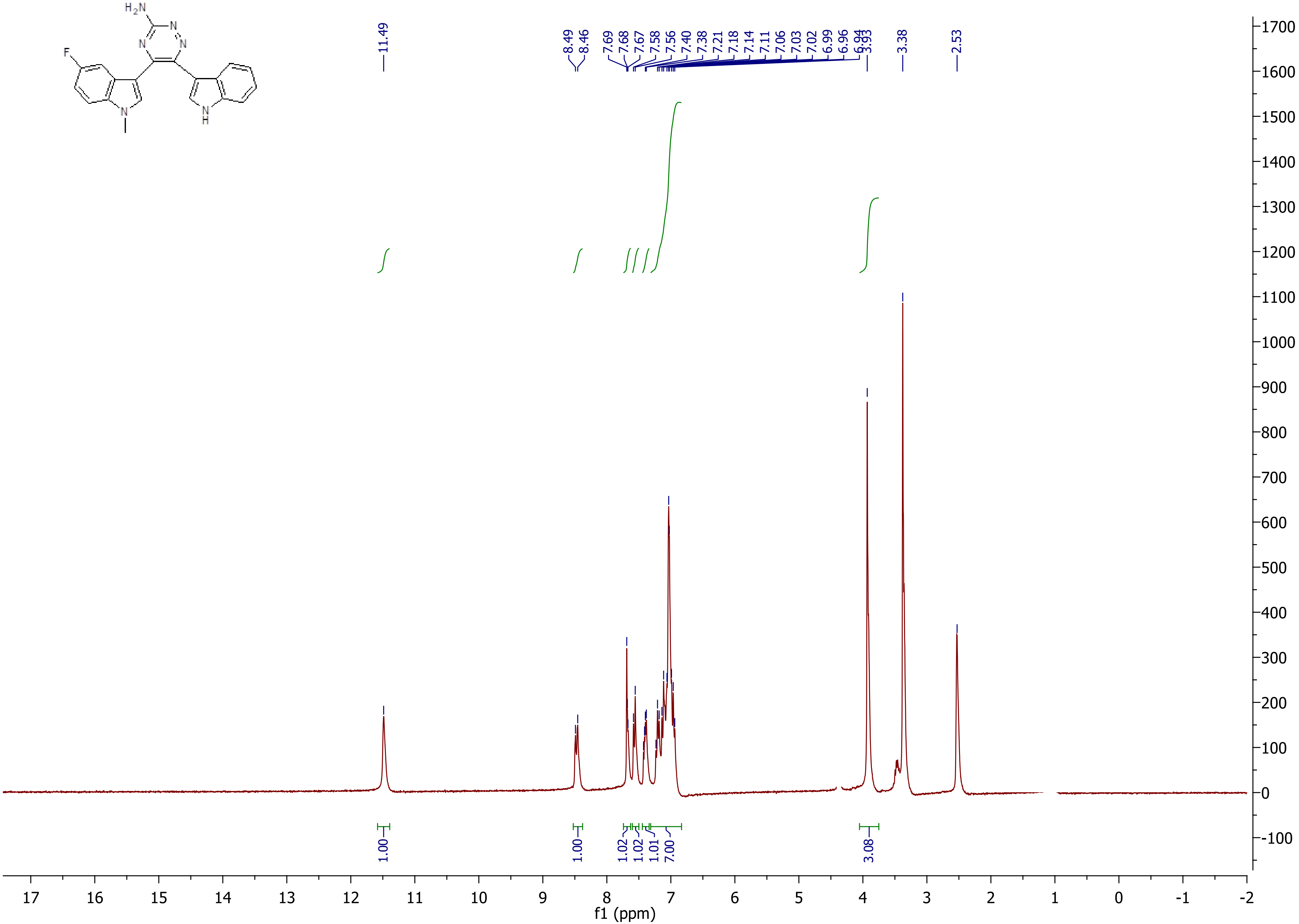

Figure S37: ^{1}H NMR spectrum of compound **6v**

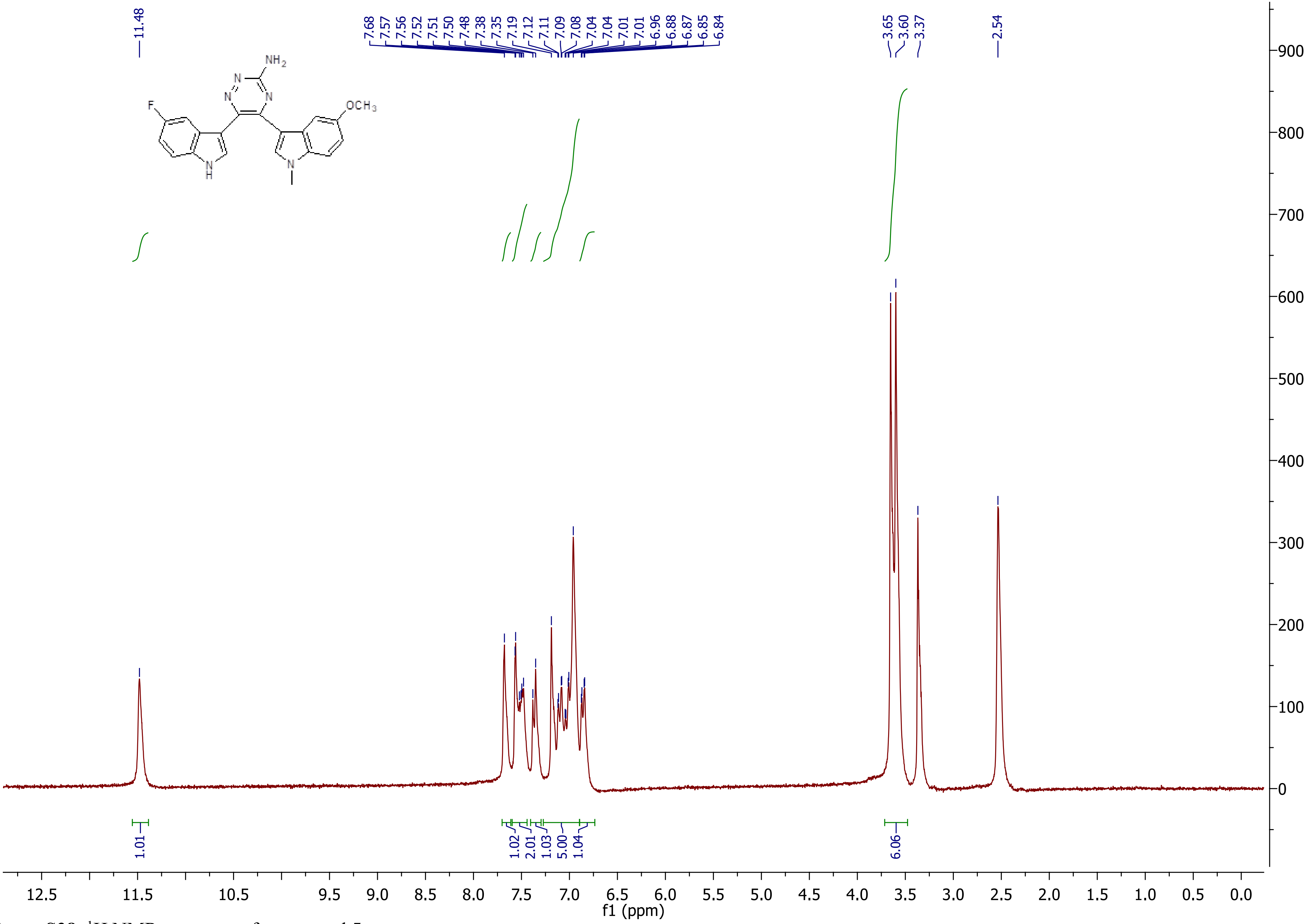

Figure S38: ^{1}H NMR spectrum of compound **5w**

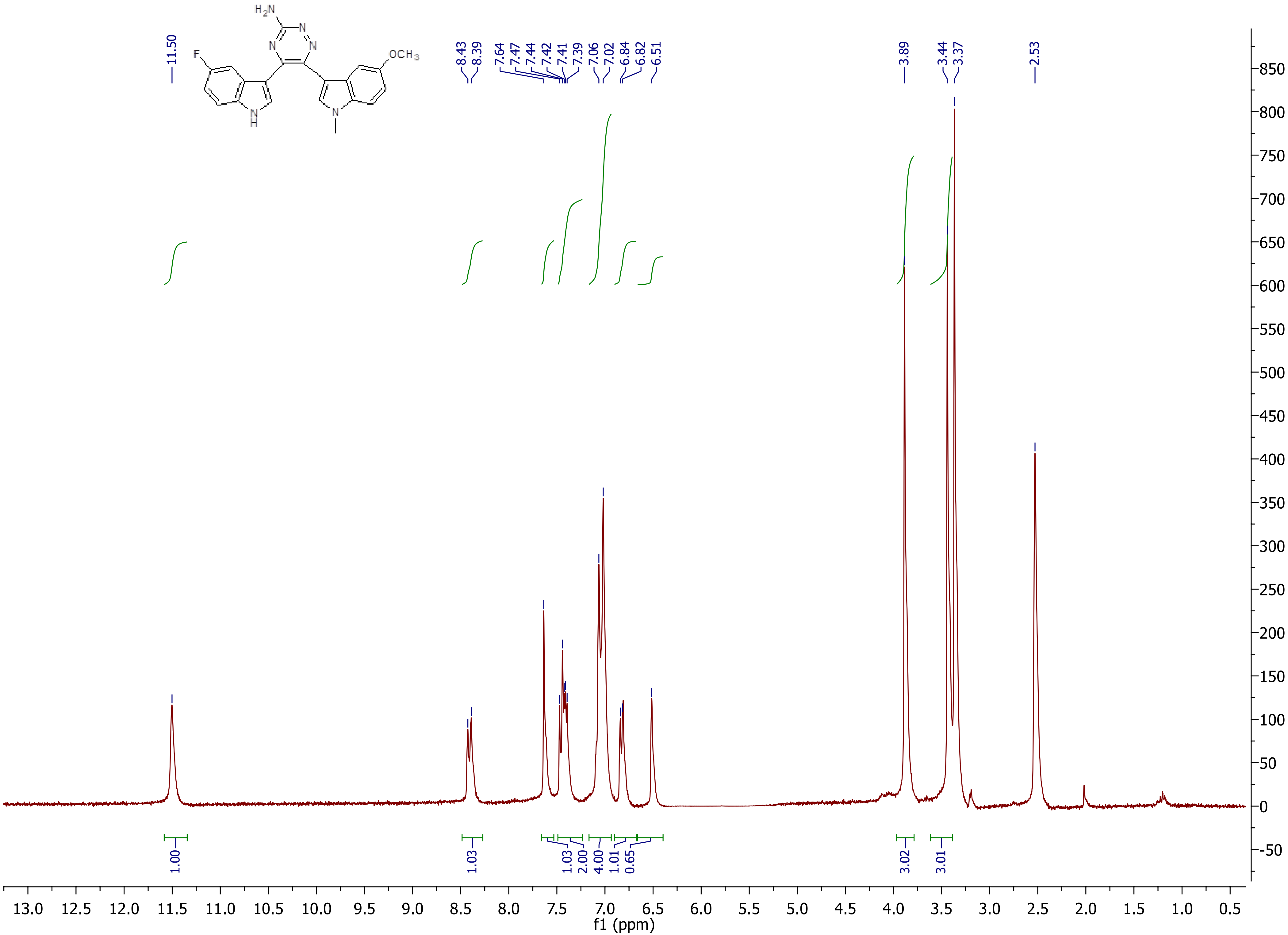

Figure S39: ^{1}H NMR spectrum of compound **6w**

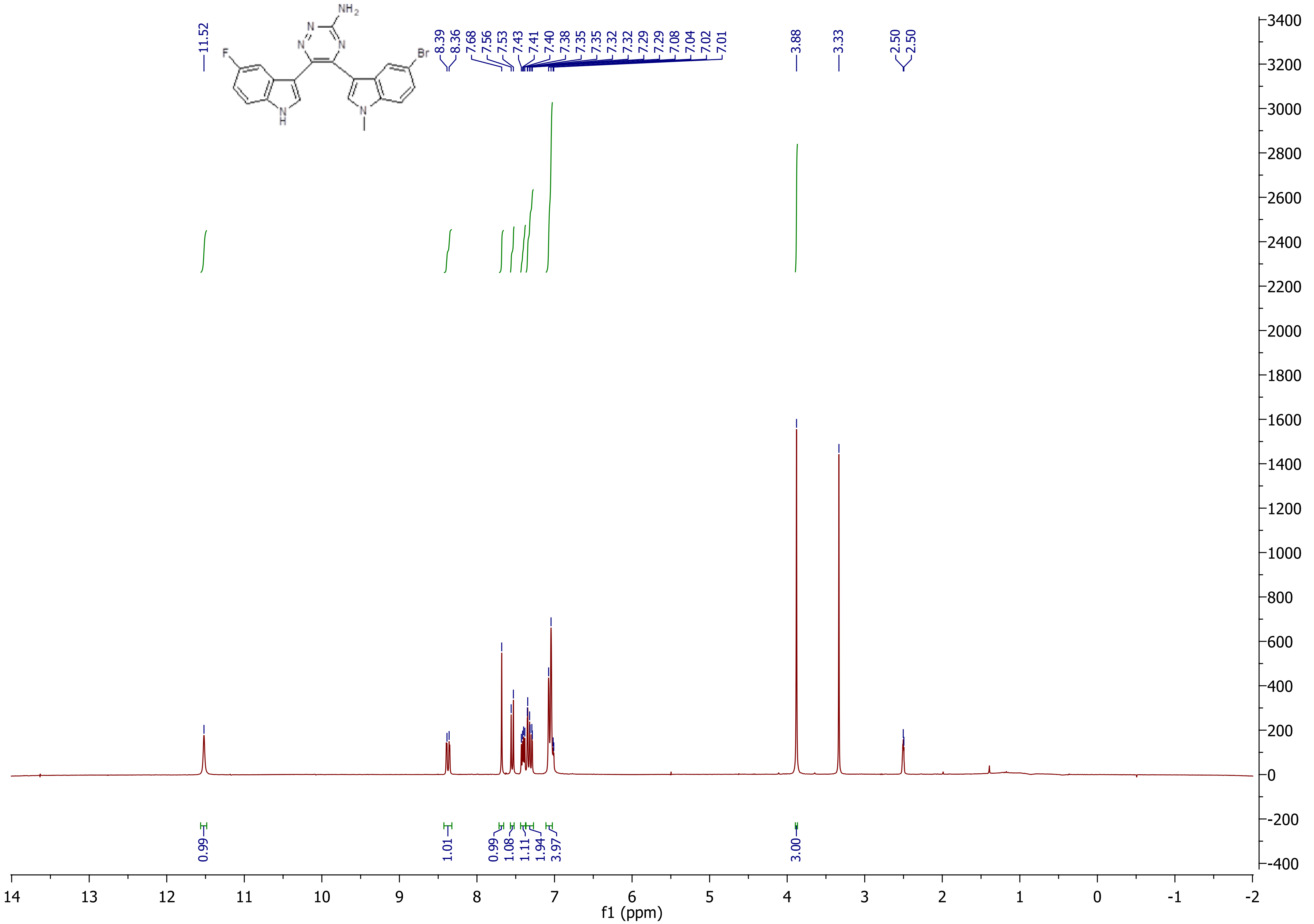

Figure S40: ^{1}H NMR spectrum of compound **5x**

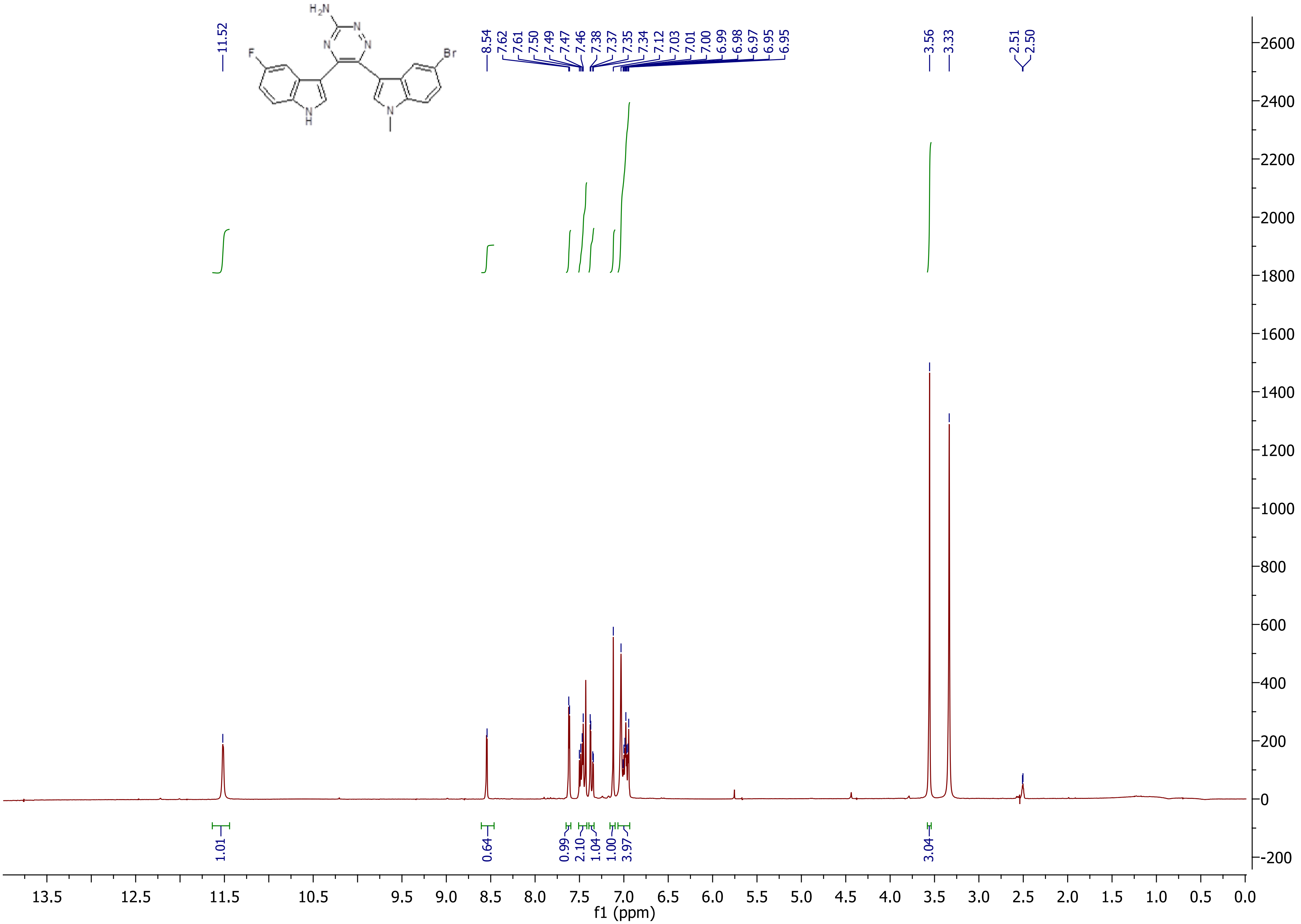

Figure S41: ^{1}H NMR spectrum of compound **6x**

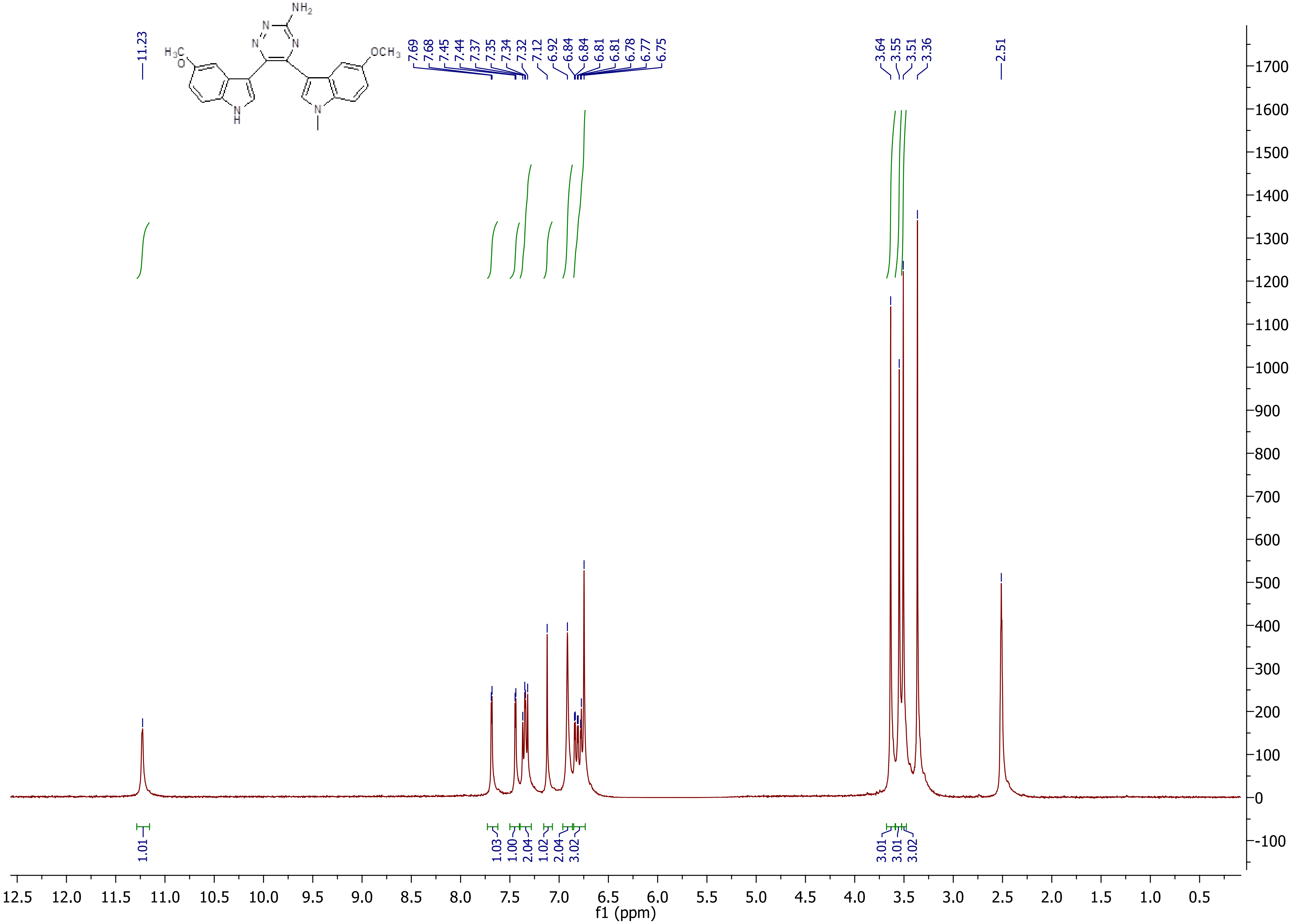

Figure S42: ^{1}H NMR spectrum of compound **5y**

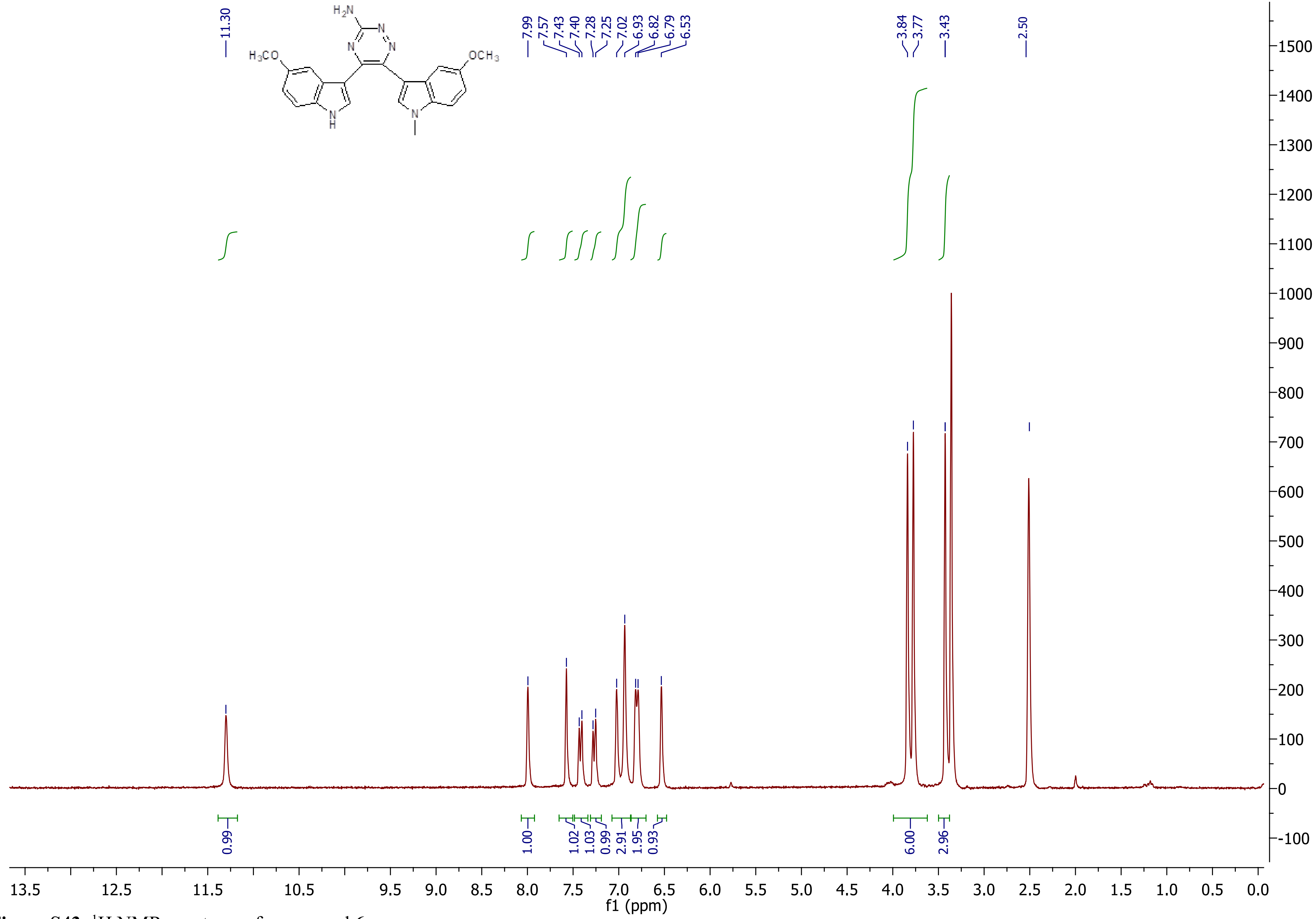

Figure S43: ^{1}H NMR spectrum of compound **6y**

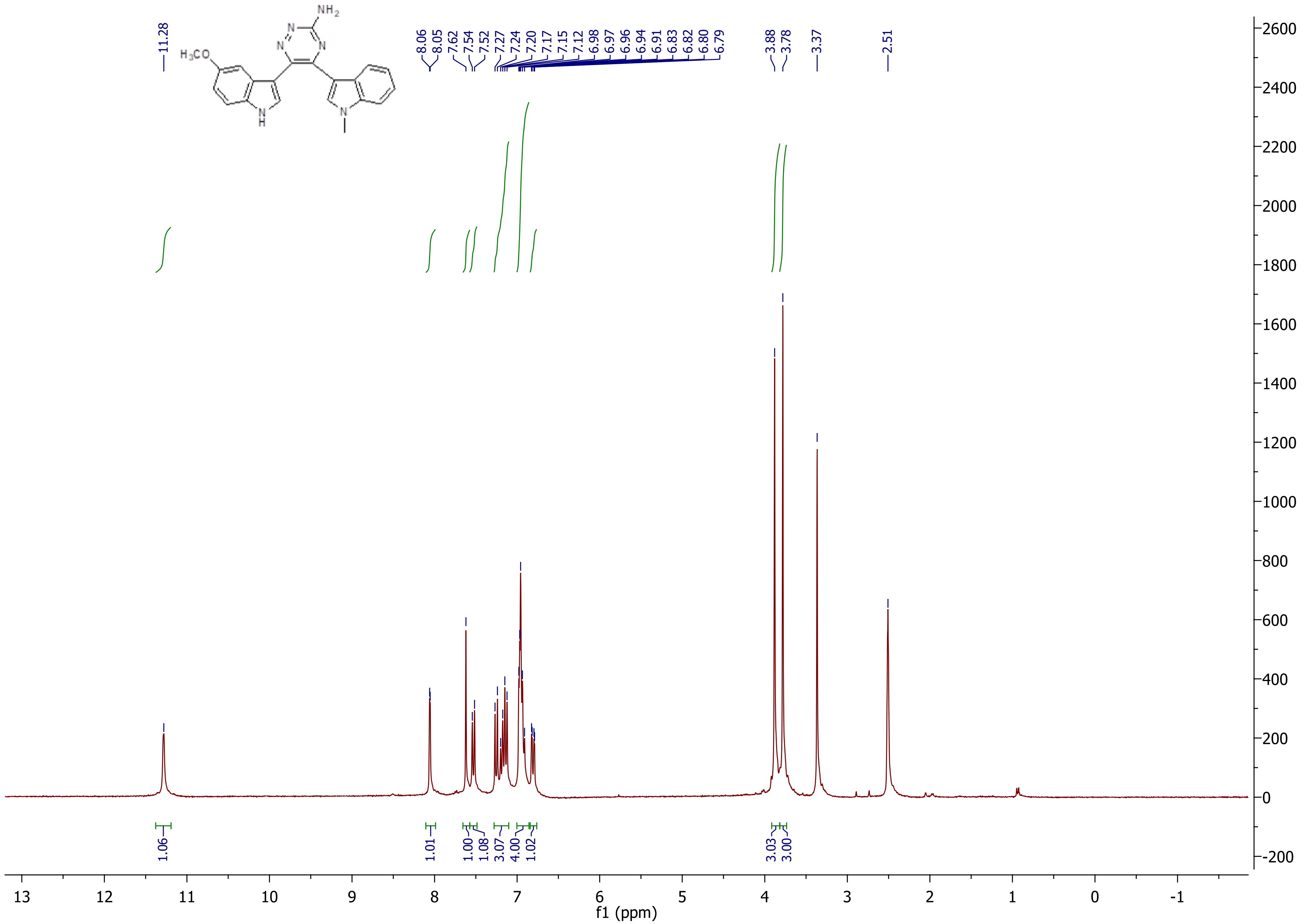

Figure S44: ^{1}H NMR spectrum of compound **5z**

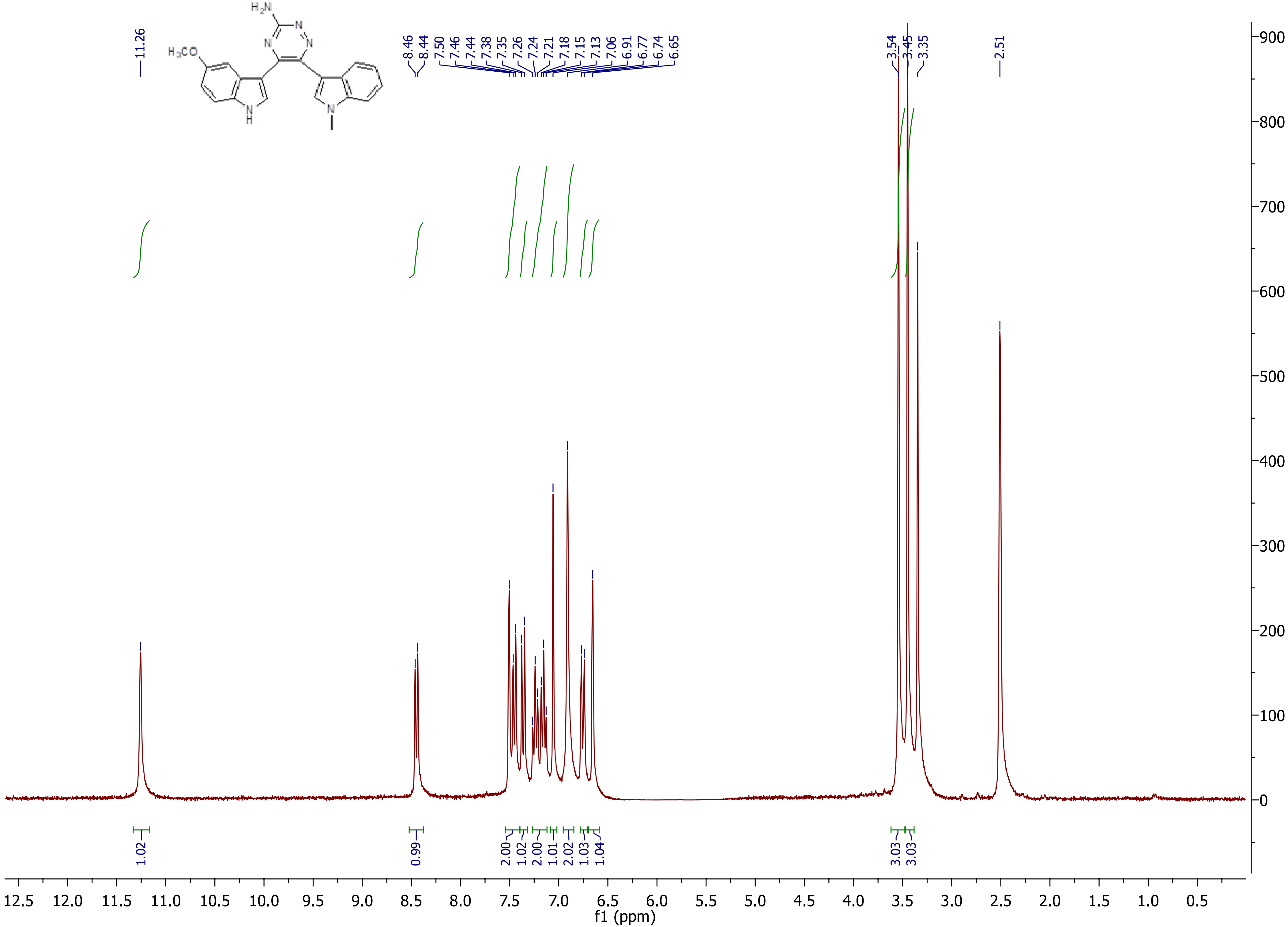

Figure S45: ^{1}H NMR spectrum of compound **6z**

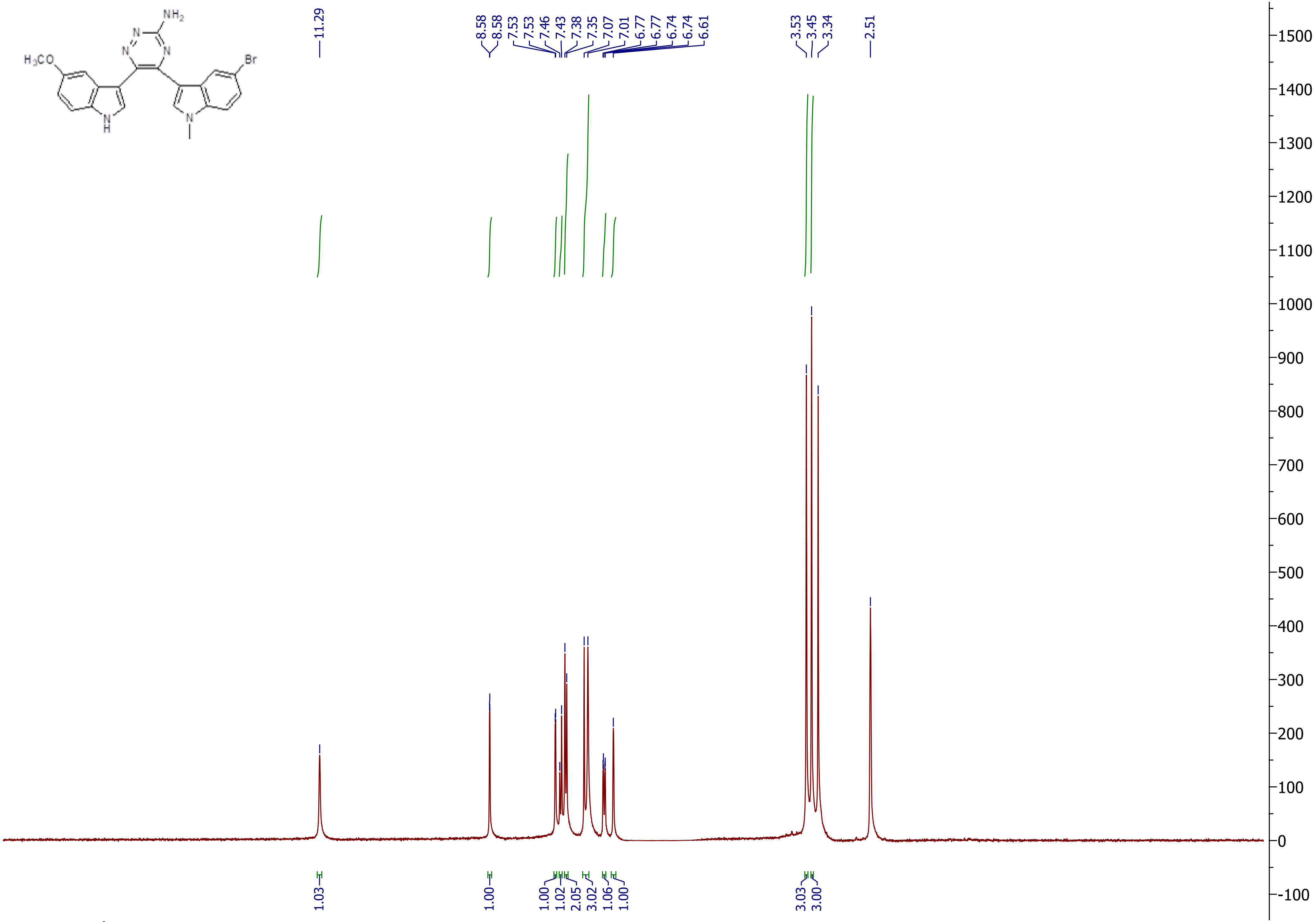

Figure S46: ^{1}H NMR spectrum of compound **5aa**

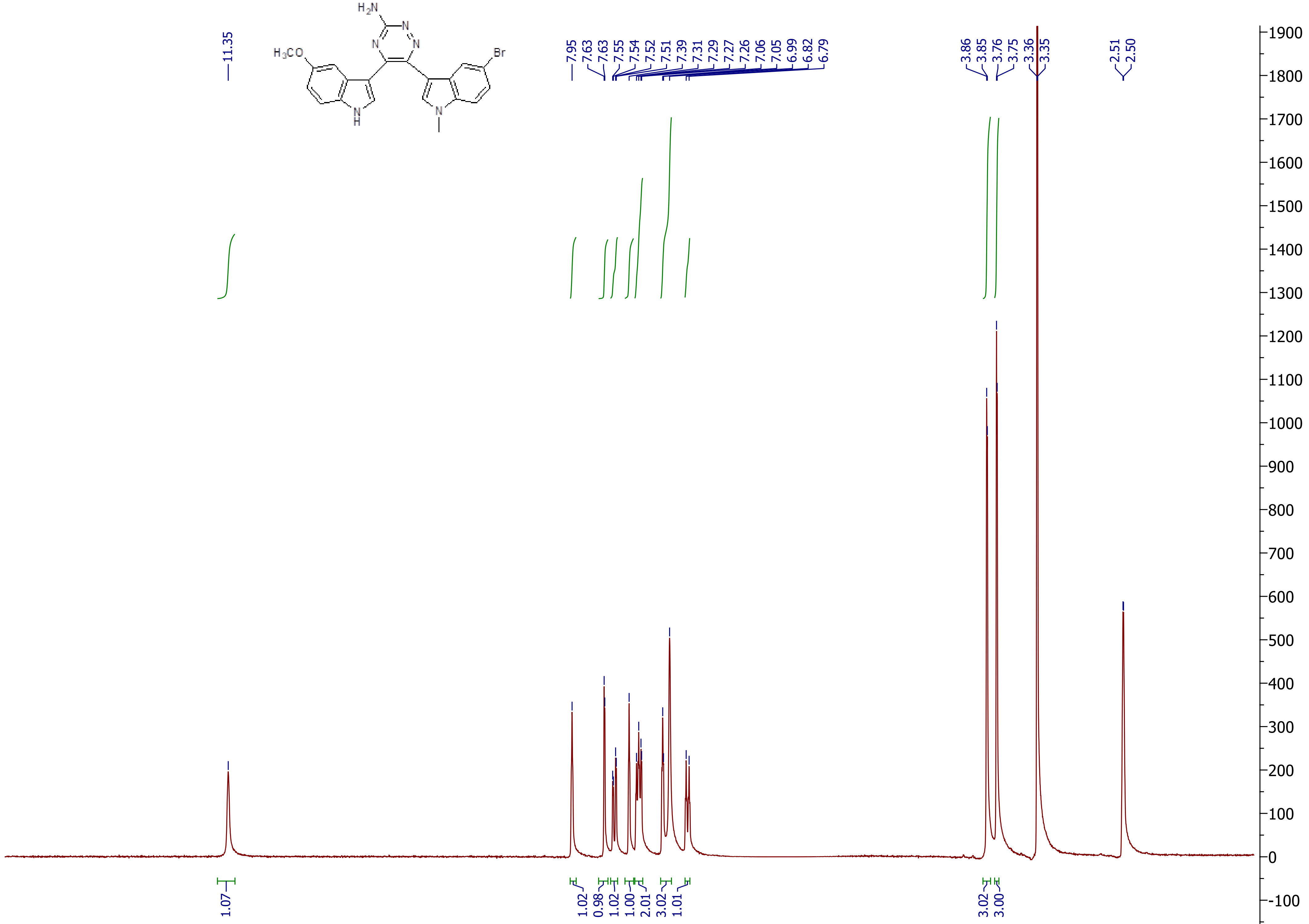

Figure S47: ^{1}H NMR spectrum of compound **6aa**

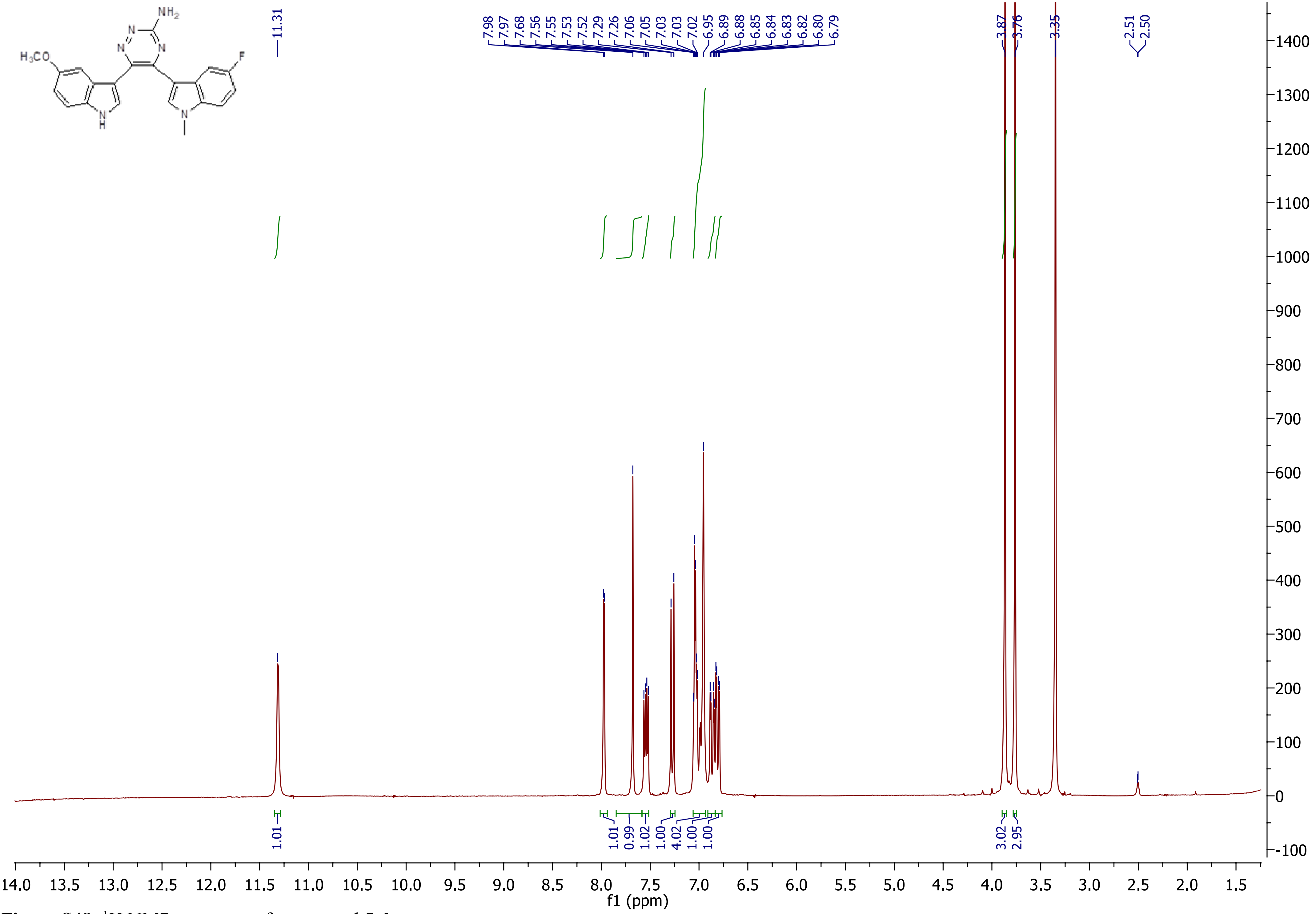

Figure S48: ^{1}H NMR spectrum of compound **5ab**

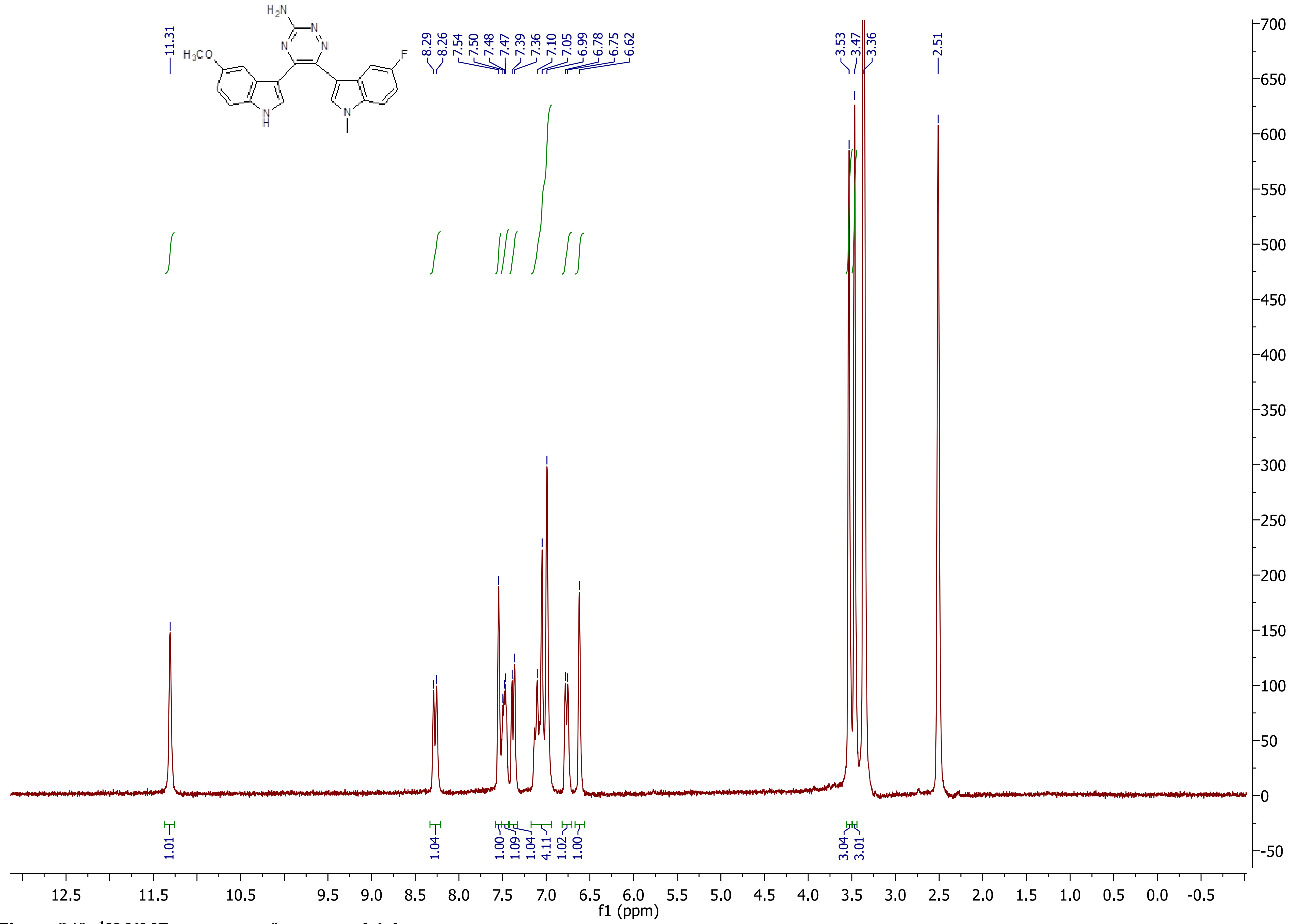

Figure S49: ^{1}H NMR spectrum of compound 6ab

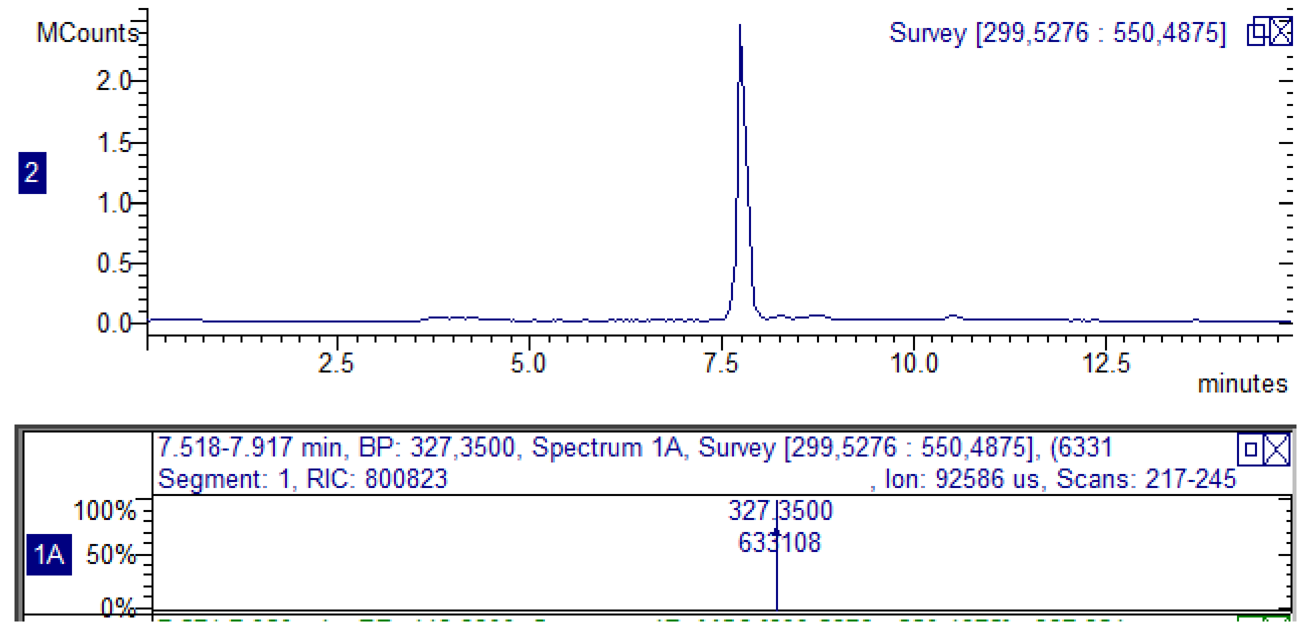

Figure S50: Chromatogram and MS spectrum of compound **5a**.

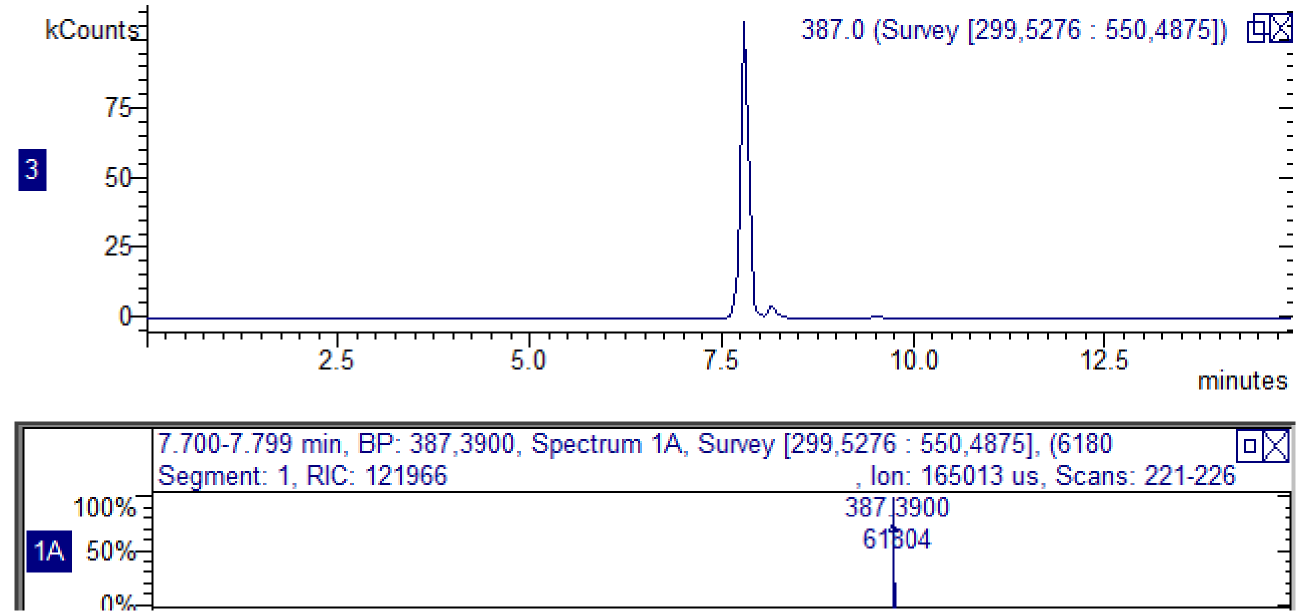

Figure S51: Chromatogram and MS spectrum of compound **5b**.

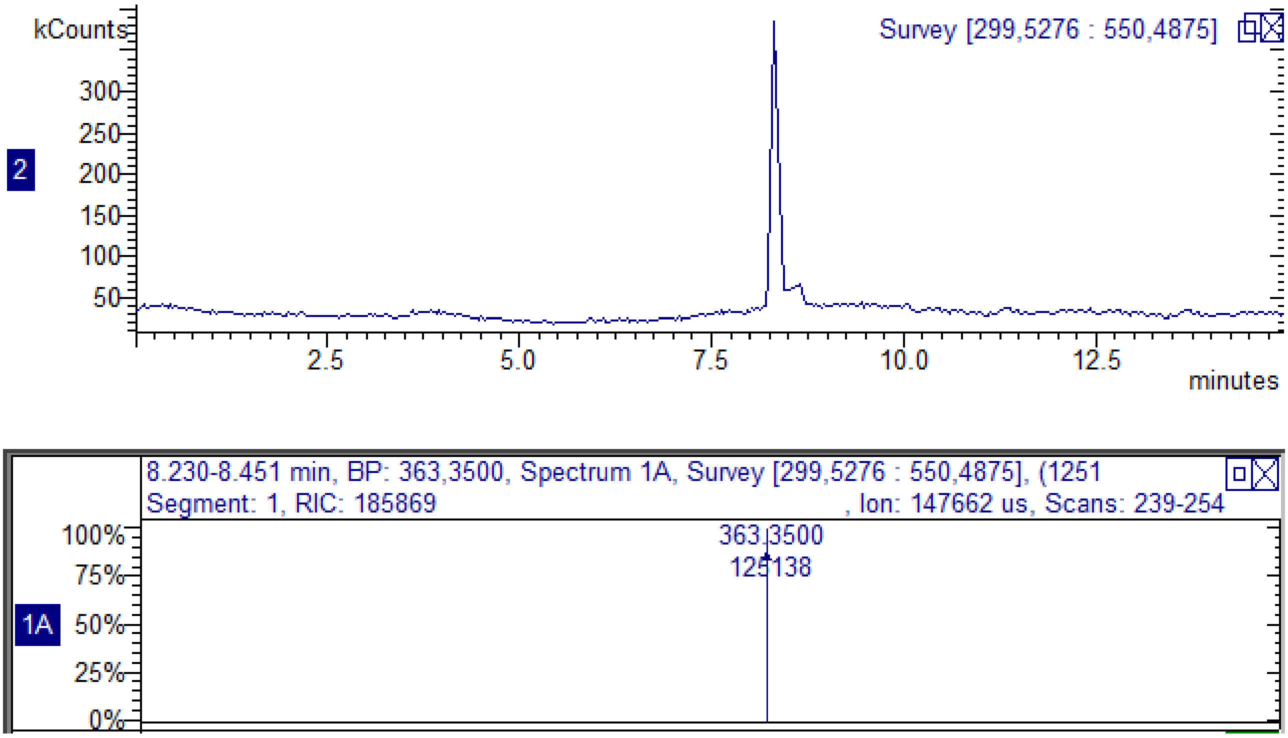

Figure S52: Chromatogram and MS spectrum of compound **5c**.

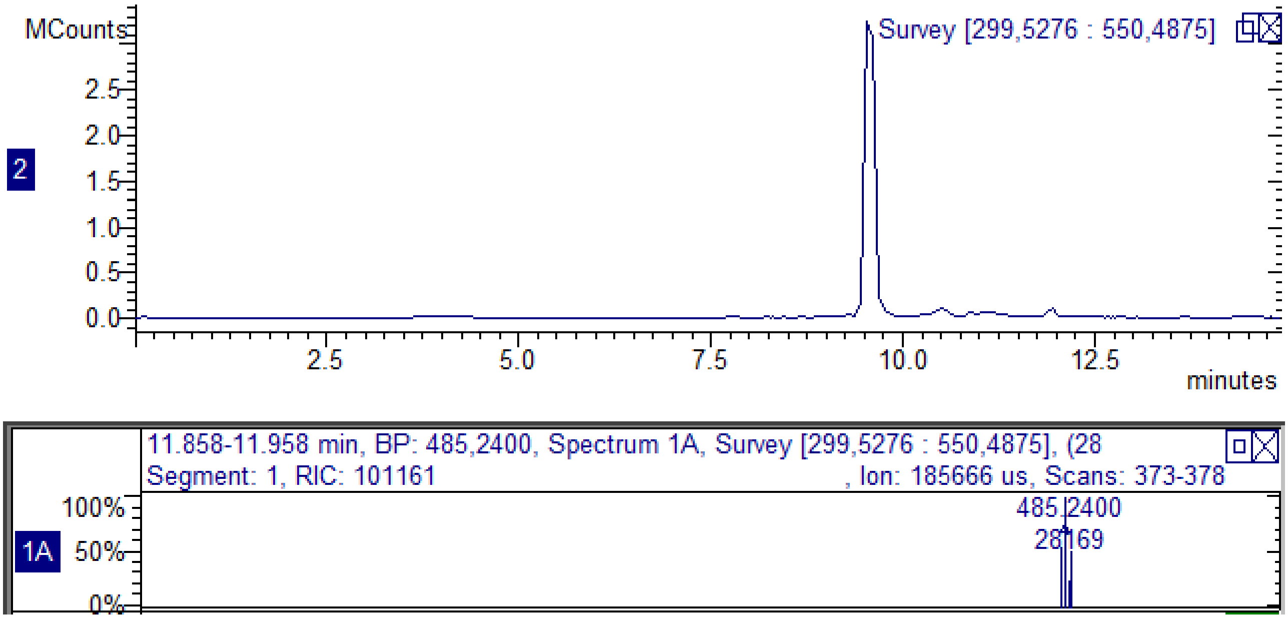

Figure S53: Chromatogram and MS spectrum of the compound **5d**.

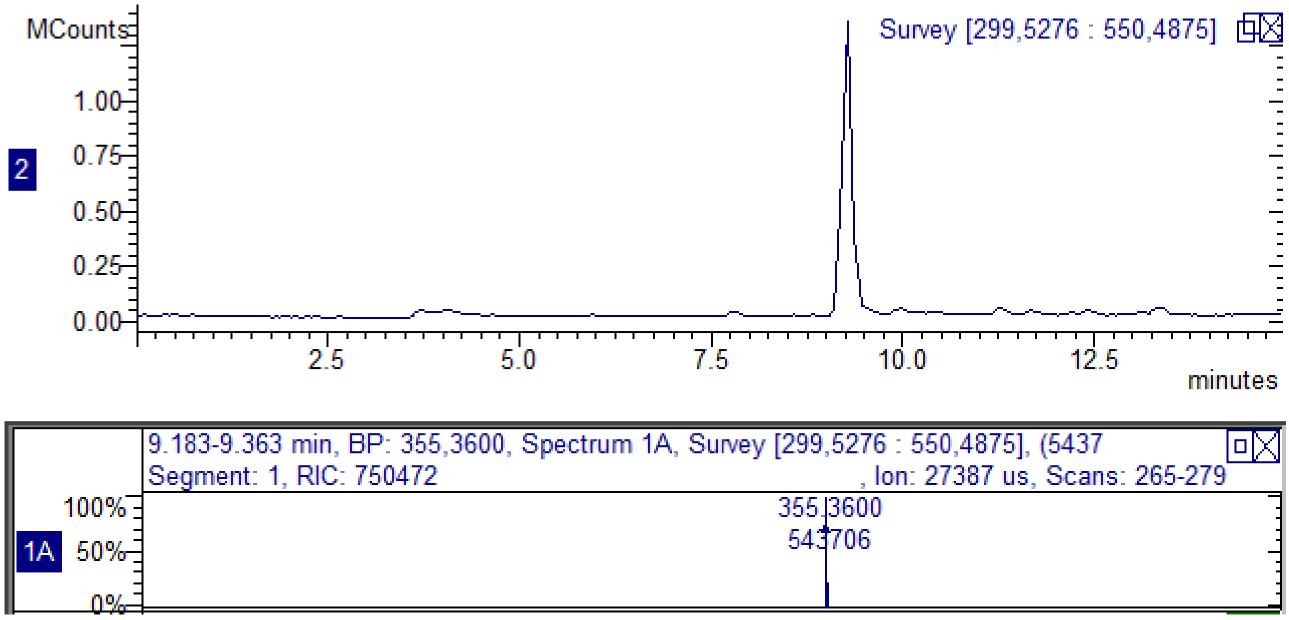

Figure S54: Chromatogram and MS spectrum of compound **5e**.

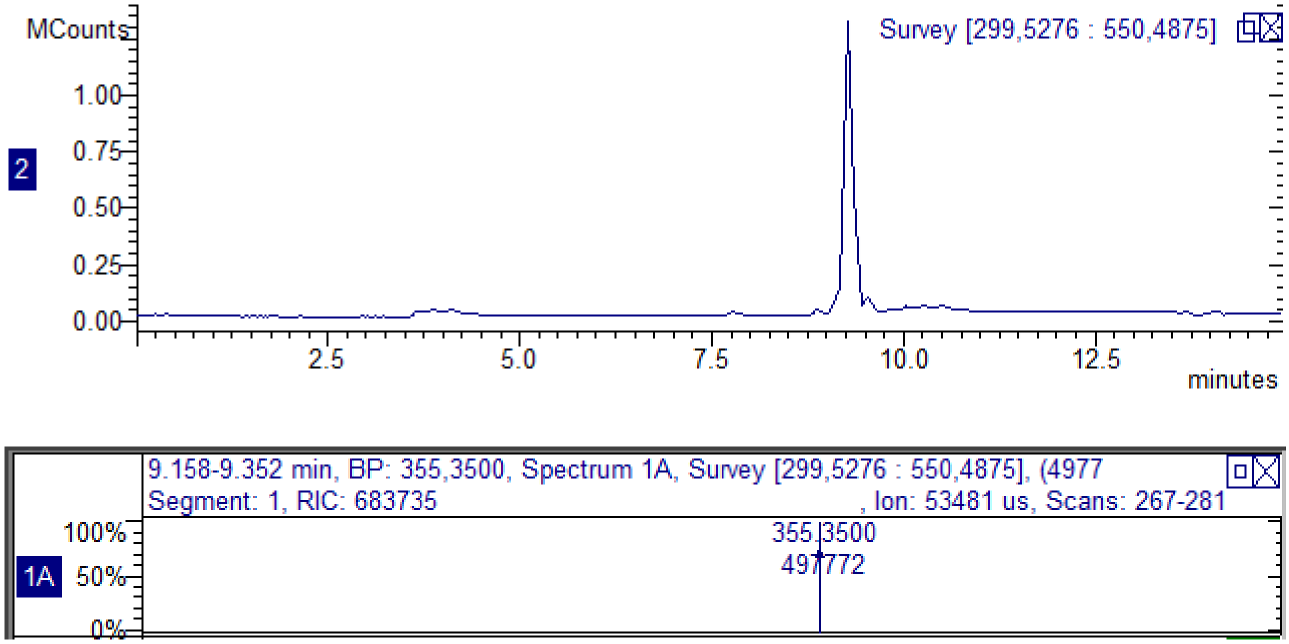

Figure S55: Chromatogram and MS spectrum of compound **5f**.

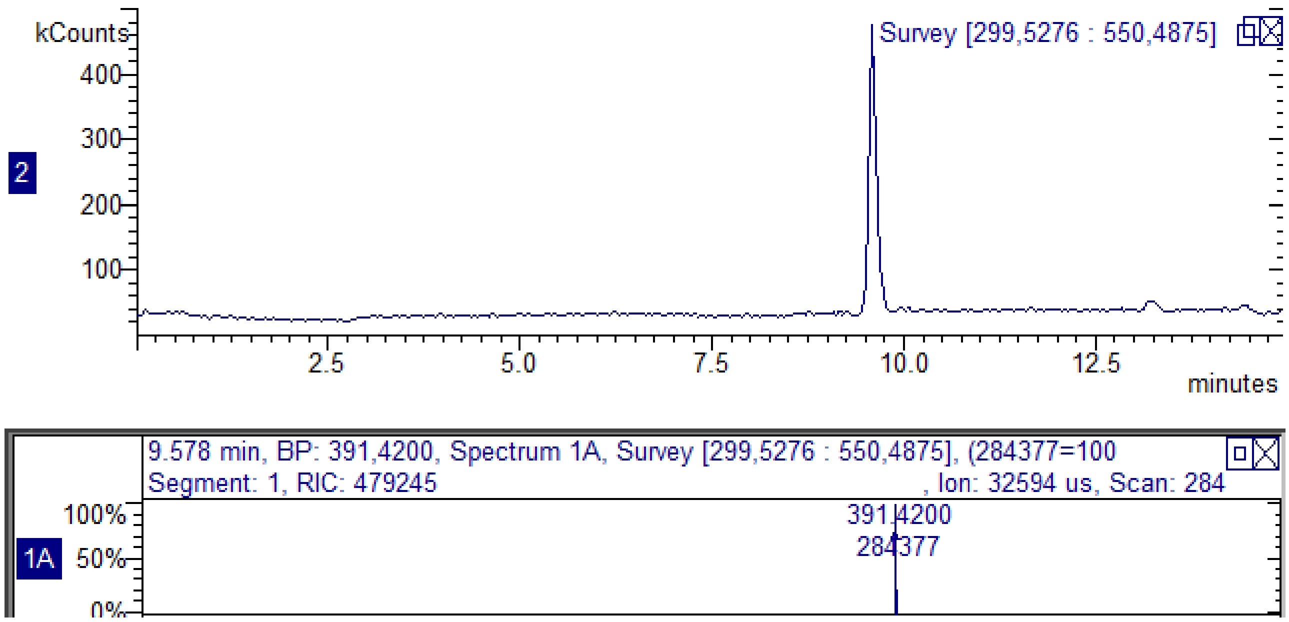

Figure S56: Chromatogram and MS spectrum of compound **5g**.

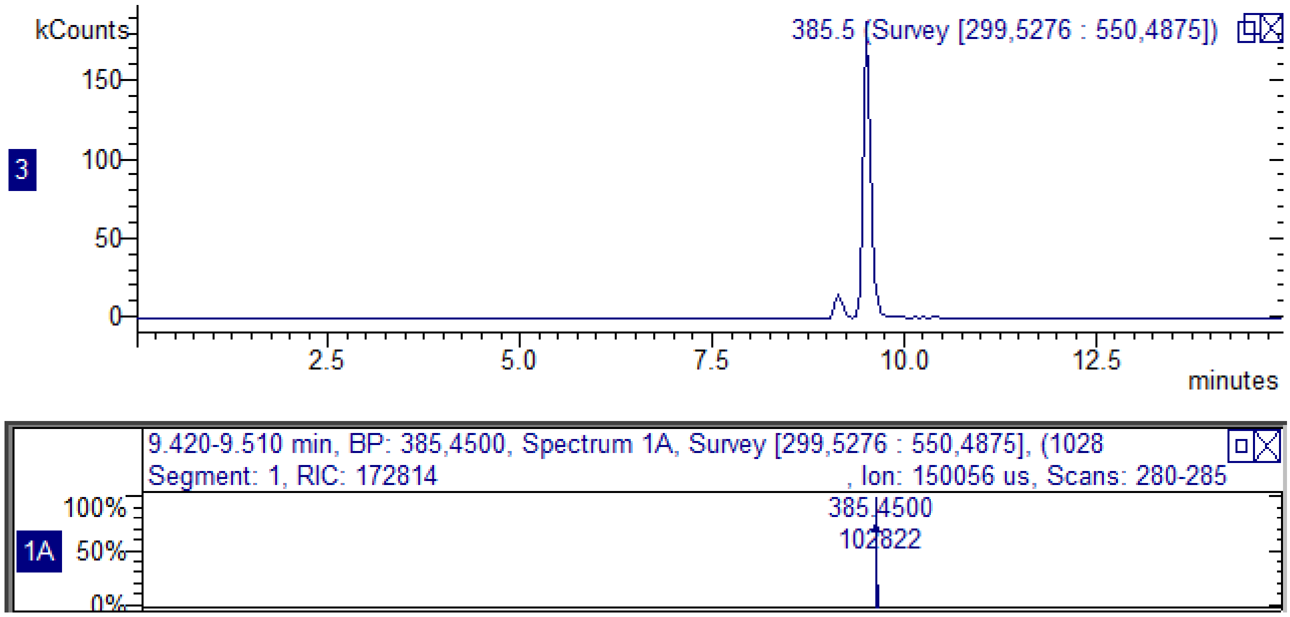

Figure S57: Chromatogram and MS spectrum of compound **5h**.

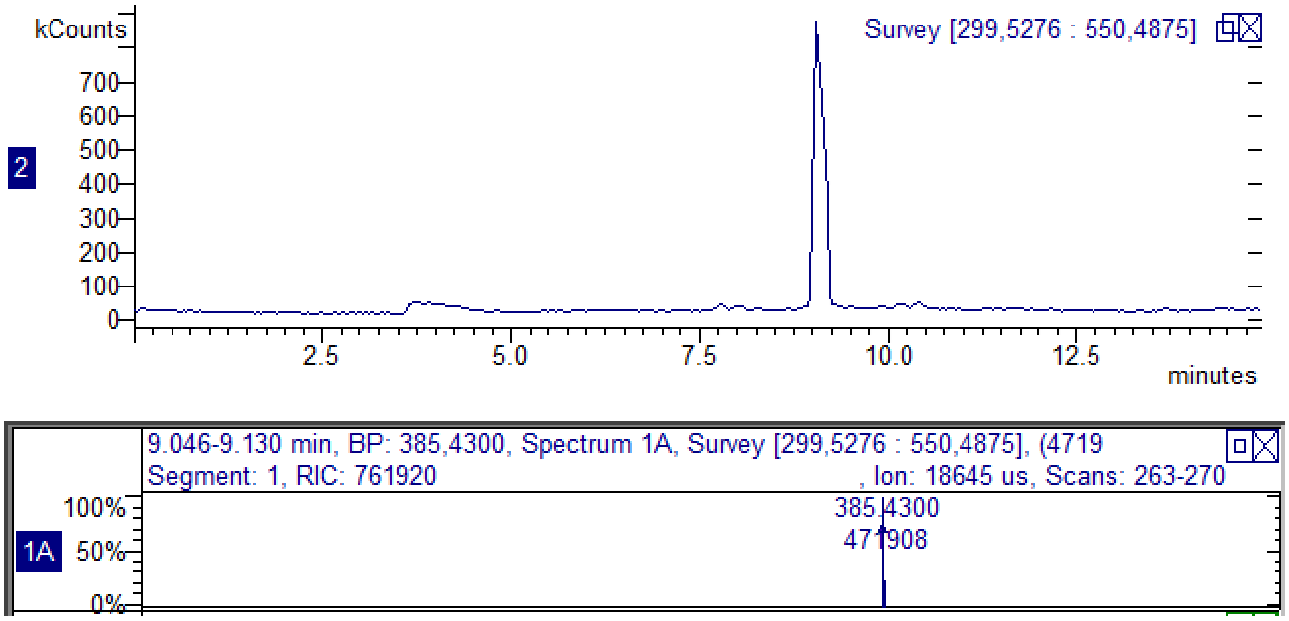

Figure S58: Chromatogram and MS spectrum of compound **6h**.

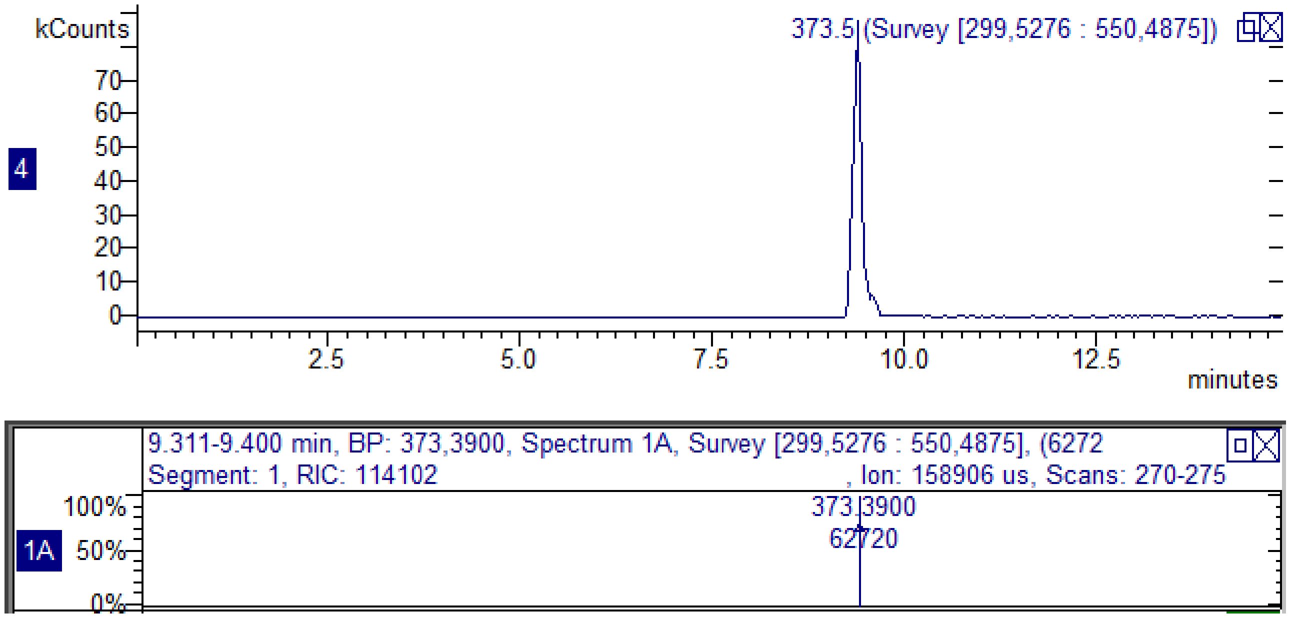

Figure S59: Chromatogram and MS spectrum of compound **5i**.

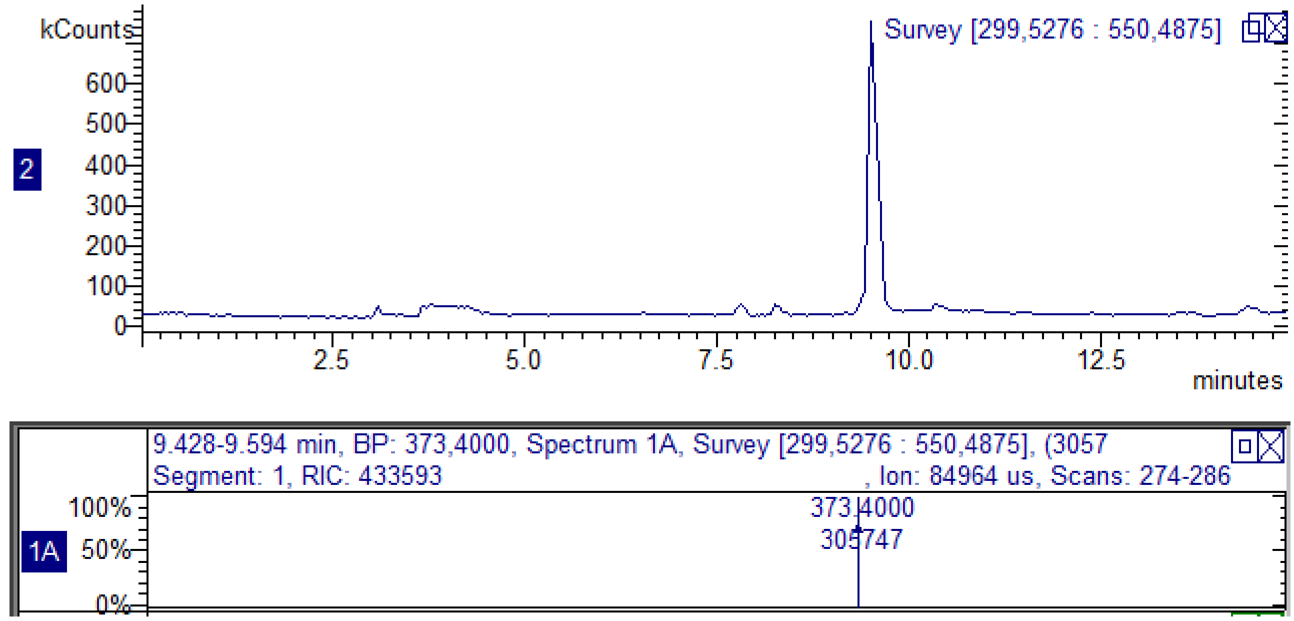

Figure S60: Chromatogram and MS spectrum of compound **6i**.

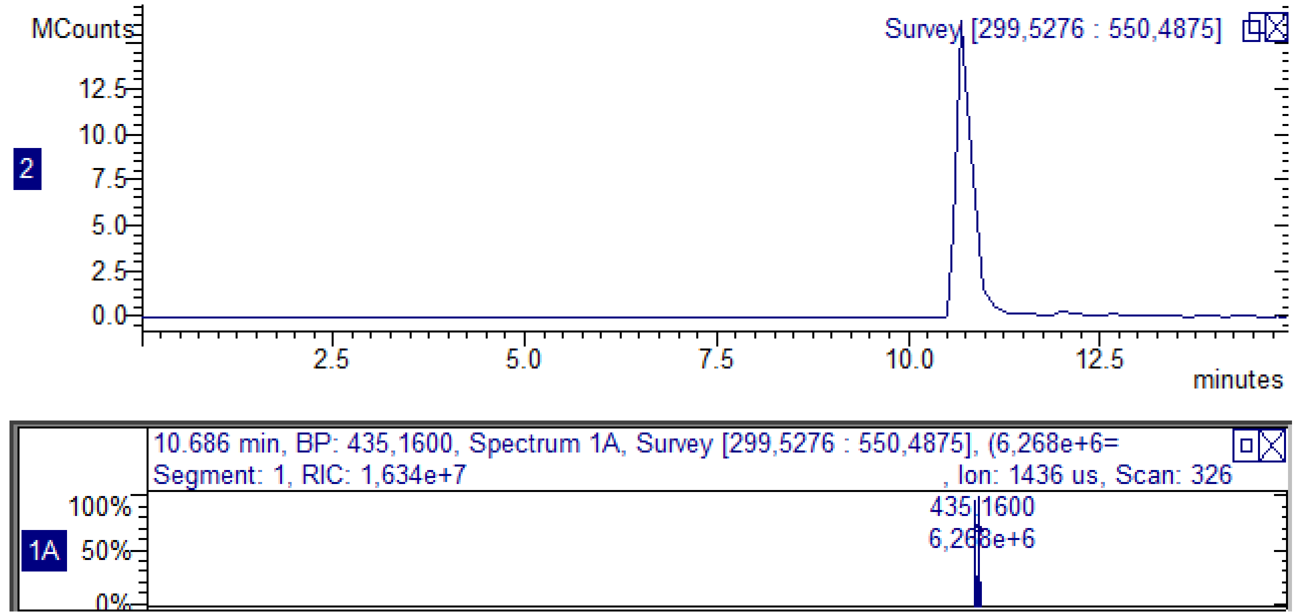

Figure S61: Chromatogram and MS spectrum of the compound **5j**.

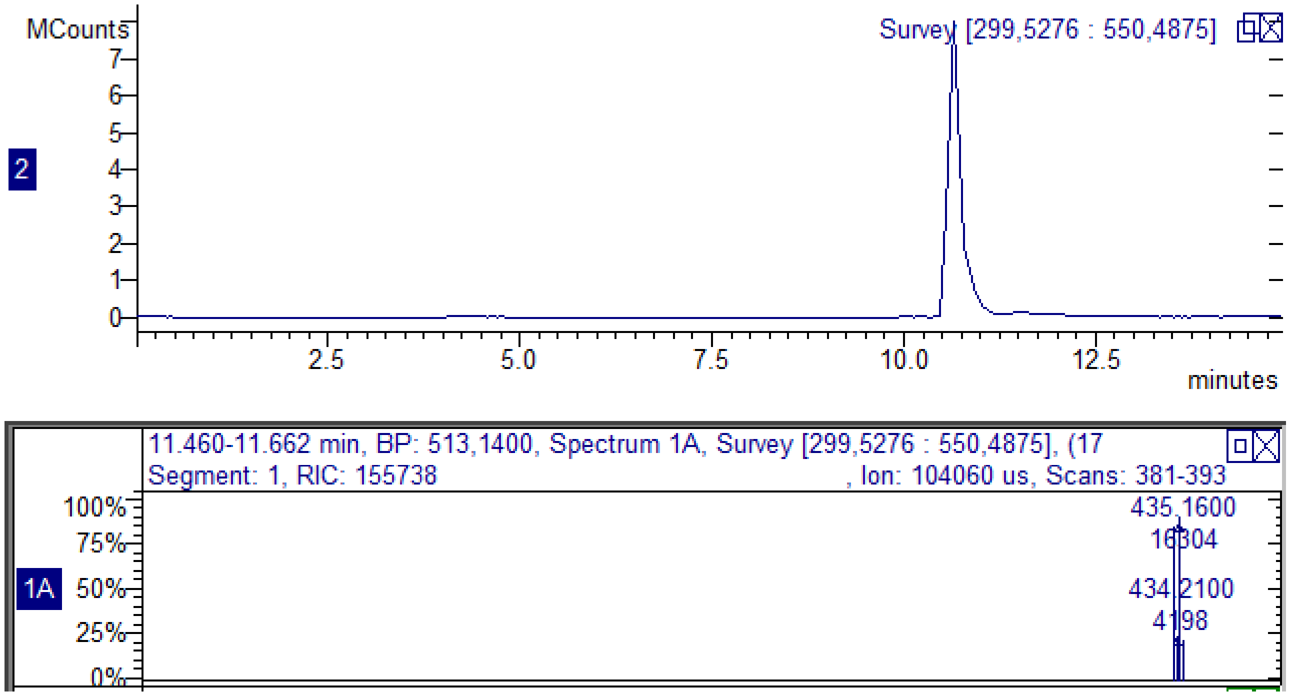

Figure S62: Chromatogram and MS spectrum of the compound **6j**.

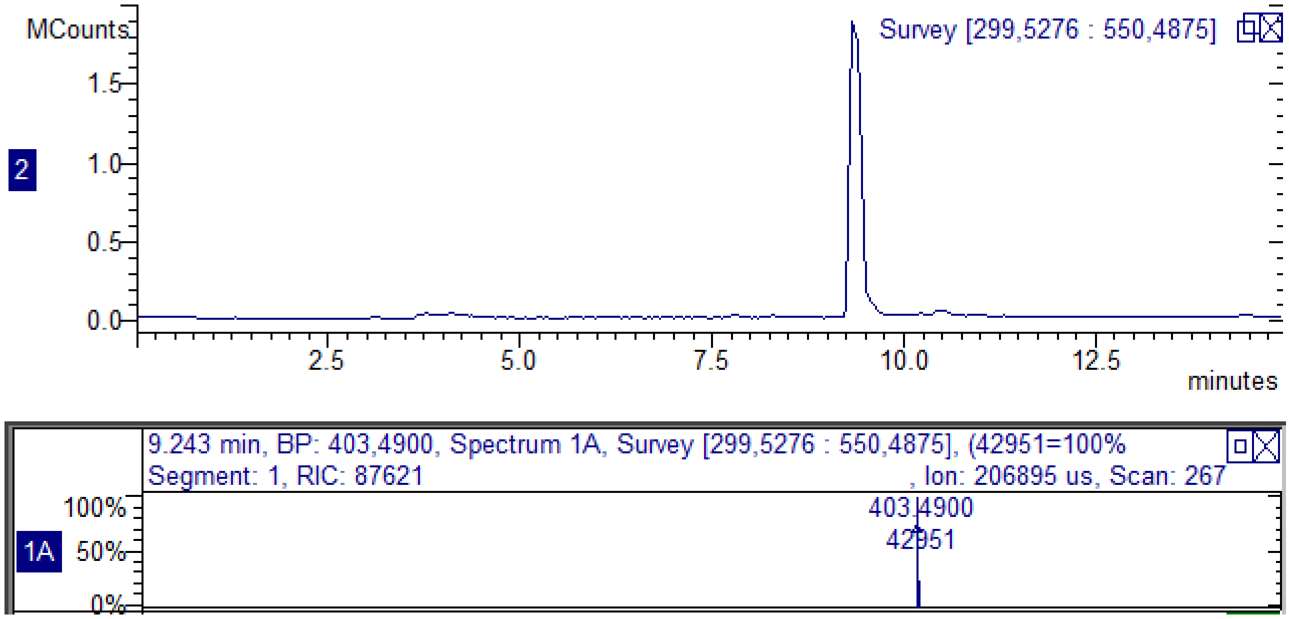

Figure S63: Chromatogram and MS spectrum of compound **5k**.

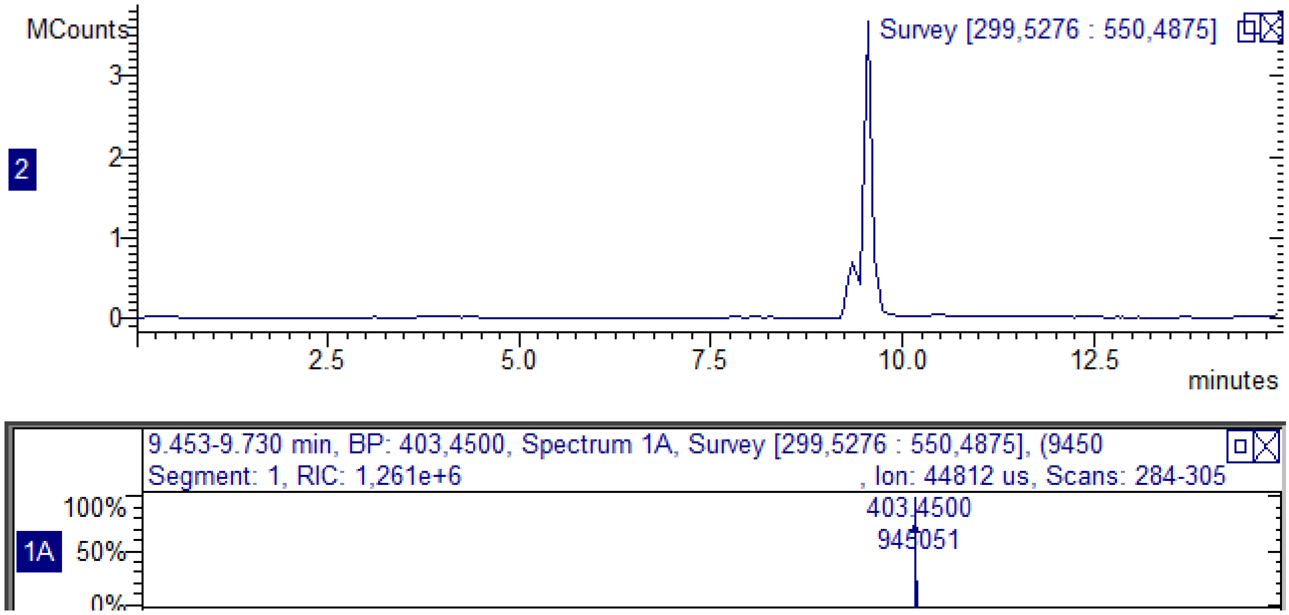

Figure S64: Chromatogram and MS spectrum of compound **6k**.

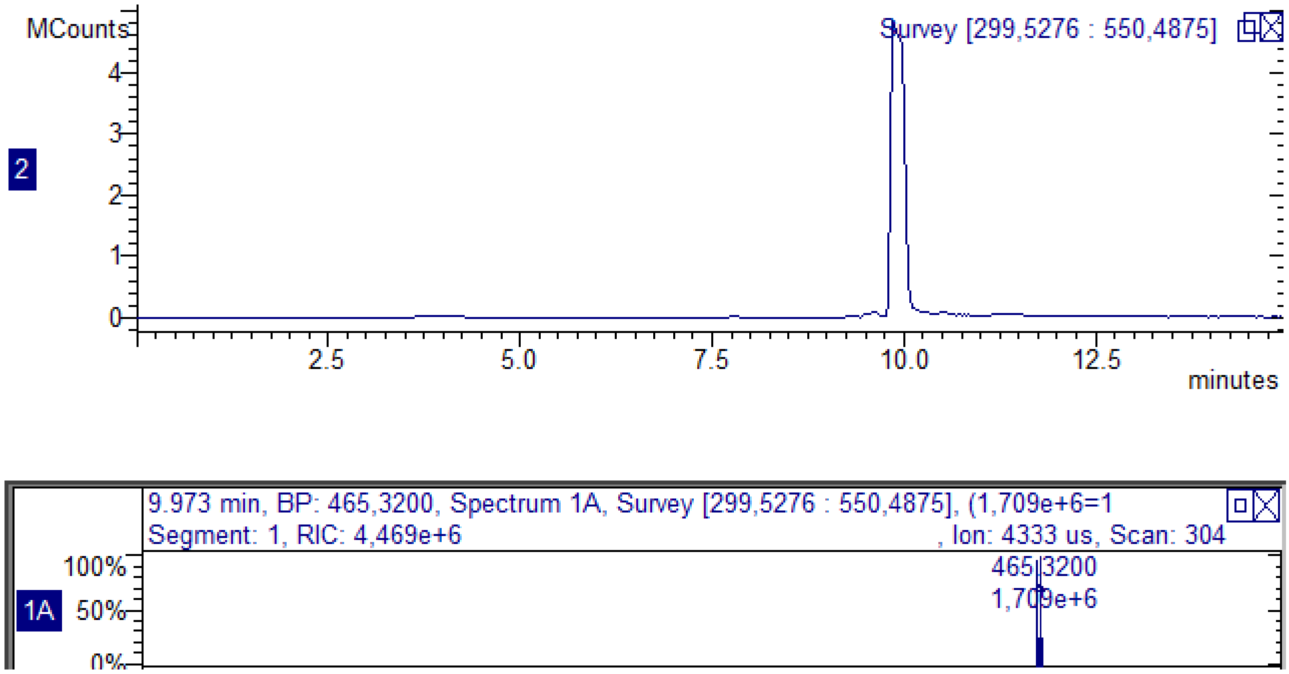

Figure S65: Chromatogram and MS spectrum of the compound **5l**.

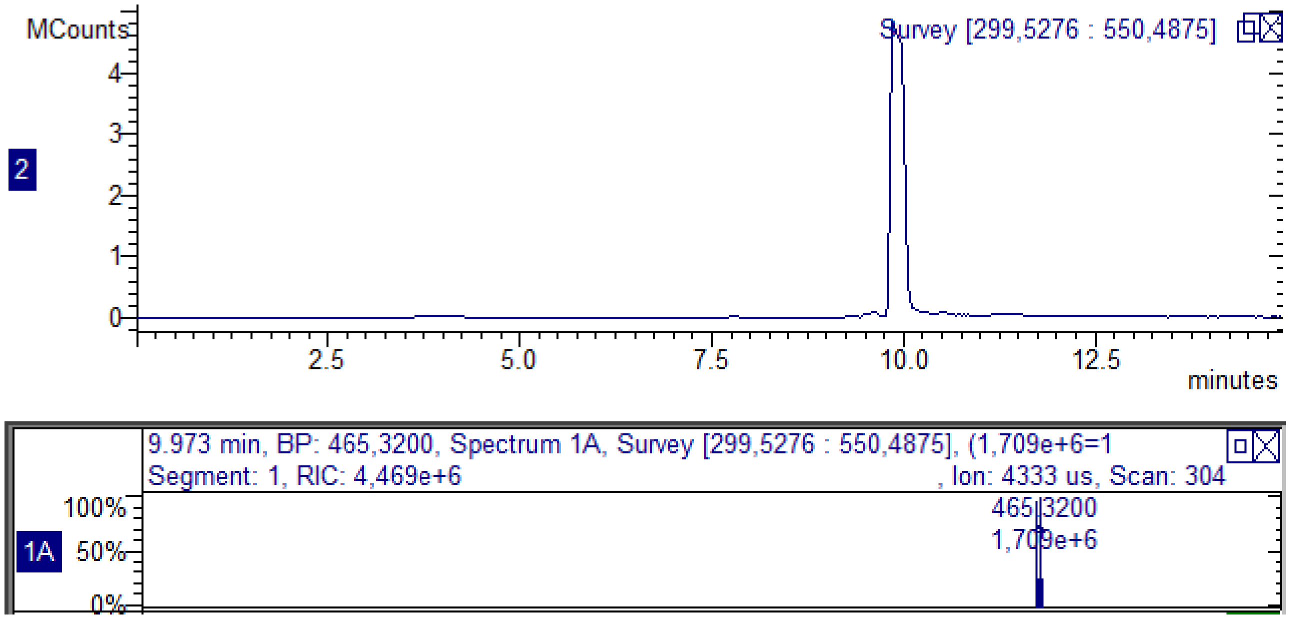

Figure S66: Chromatogram and MS spectrum of the compound **6l**.

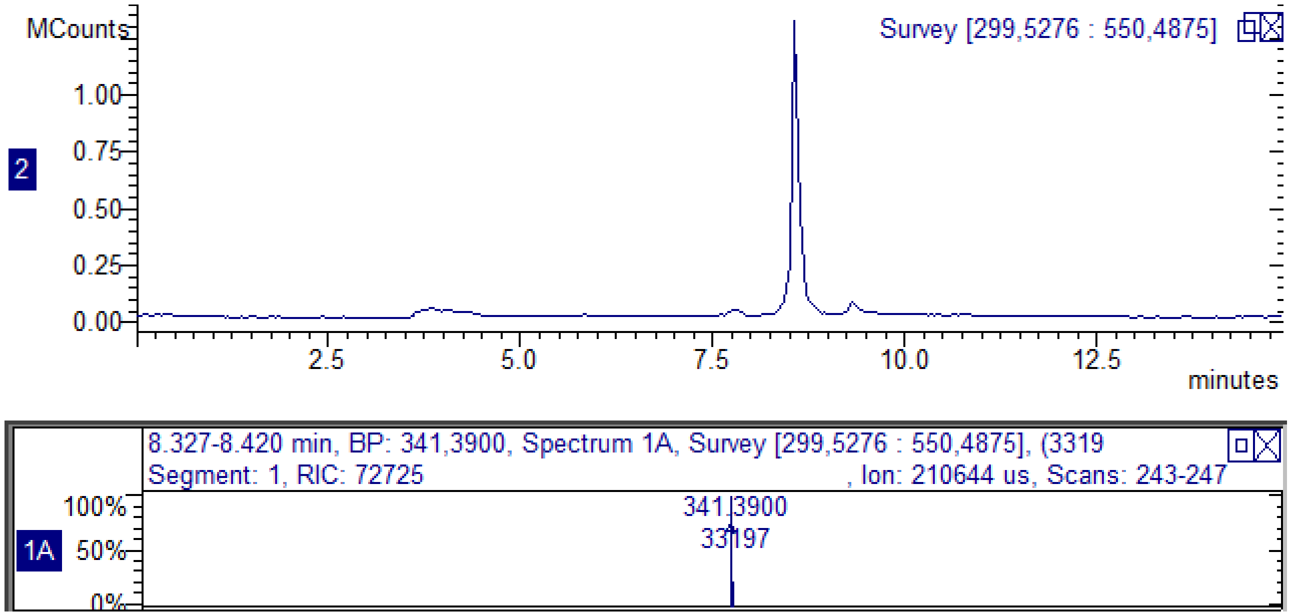

Figure S67: Chromatogram and MS spectrum of compound **5m**.

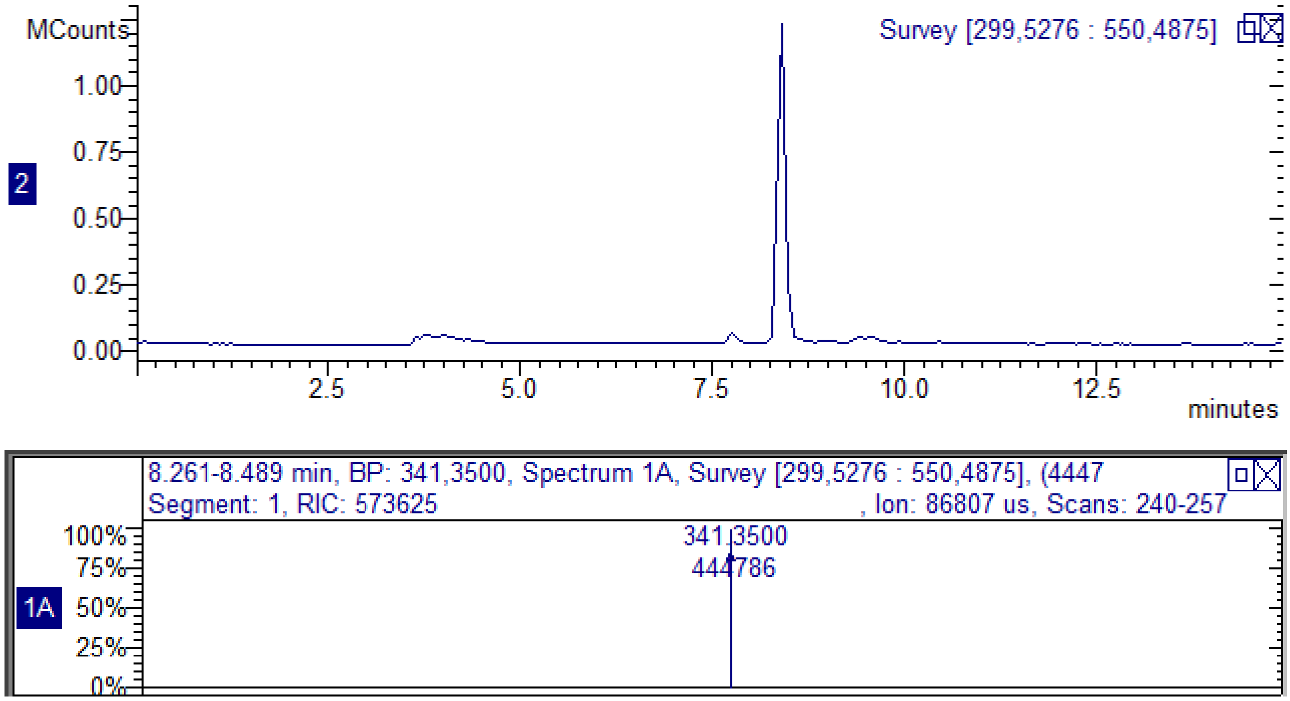

Figure S68: Chromatogram and MS spectrum of compound **6m**.

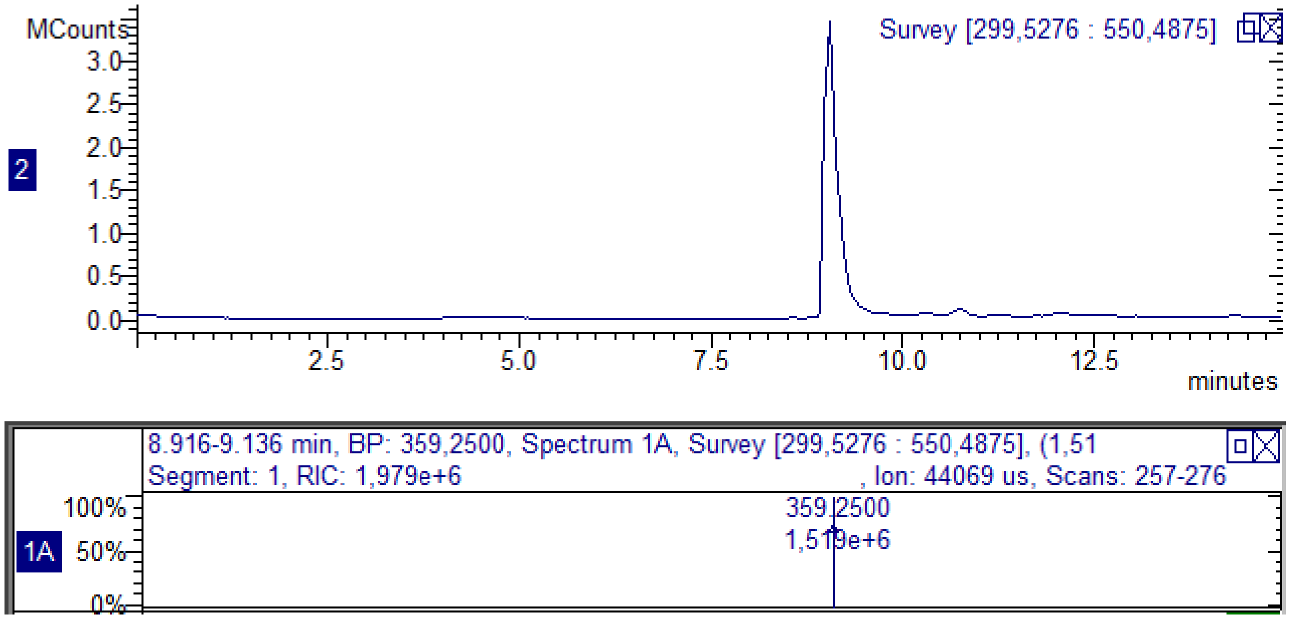

Figure S69: Chromatogram and MS spectrum of compound **5n**.

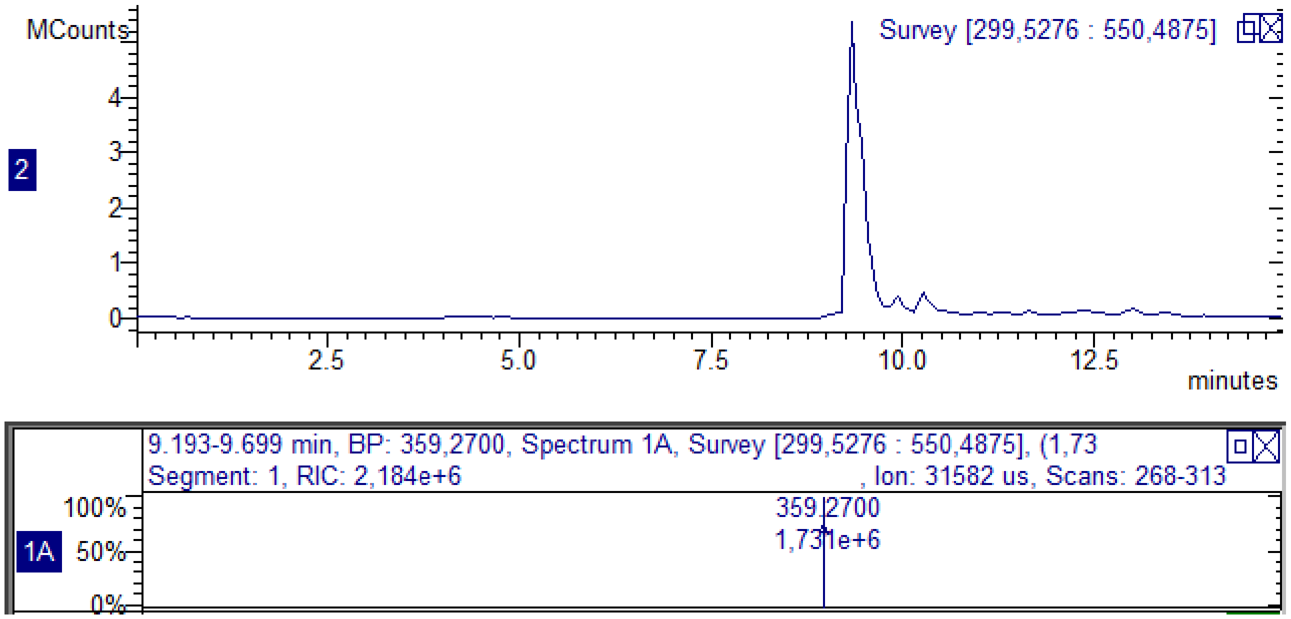

Figure S70: Chromatogram and MS spectrum of compound **6n**.

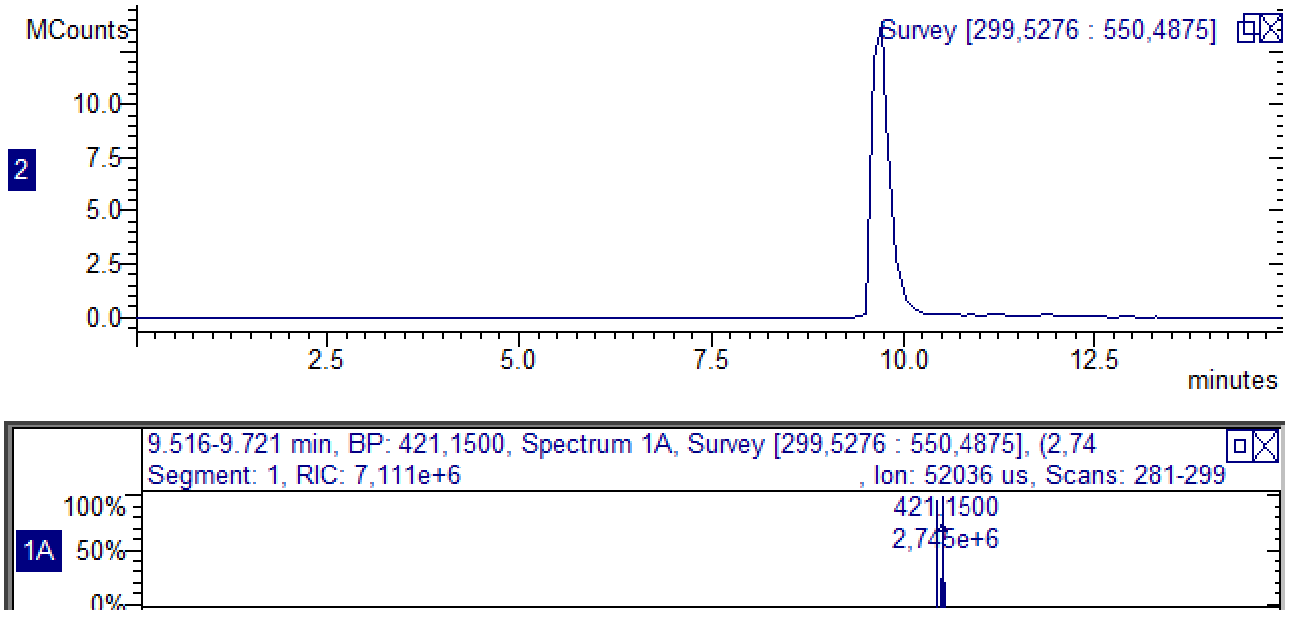

Figure S71: Chromatogram and MS spectrum with detail on the isotopic pattern of the bromine compound **5o**.

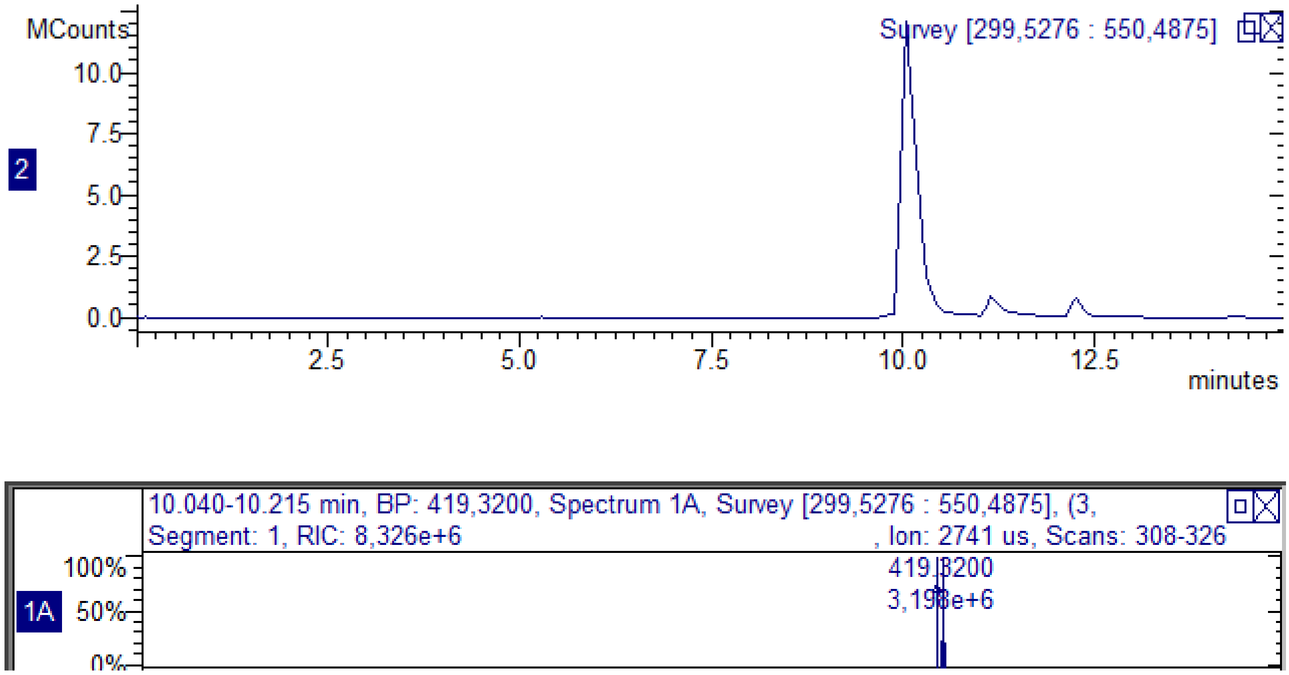

Figure S72: Chromatogram and MS spectrum with detail on the isotopic pattern of the bromine compound **6o**.

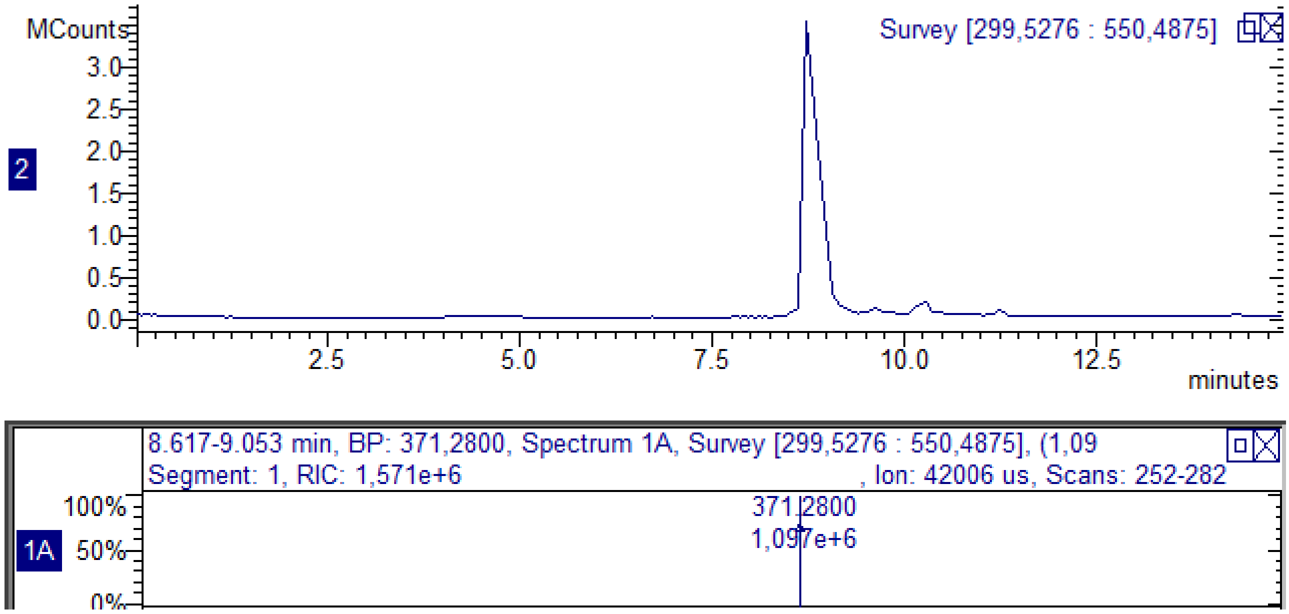

Figure S73: Chromatogram and MS spectrum of compound **5p**.

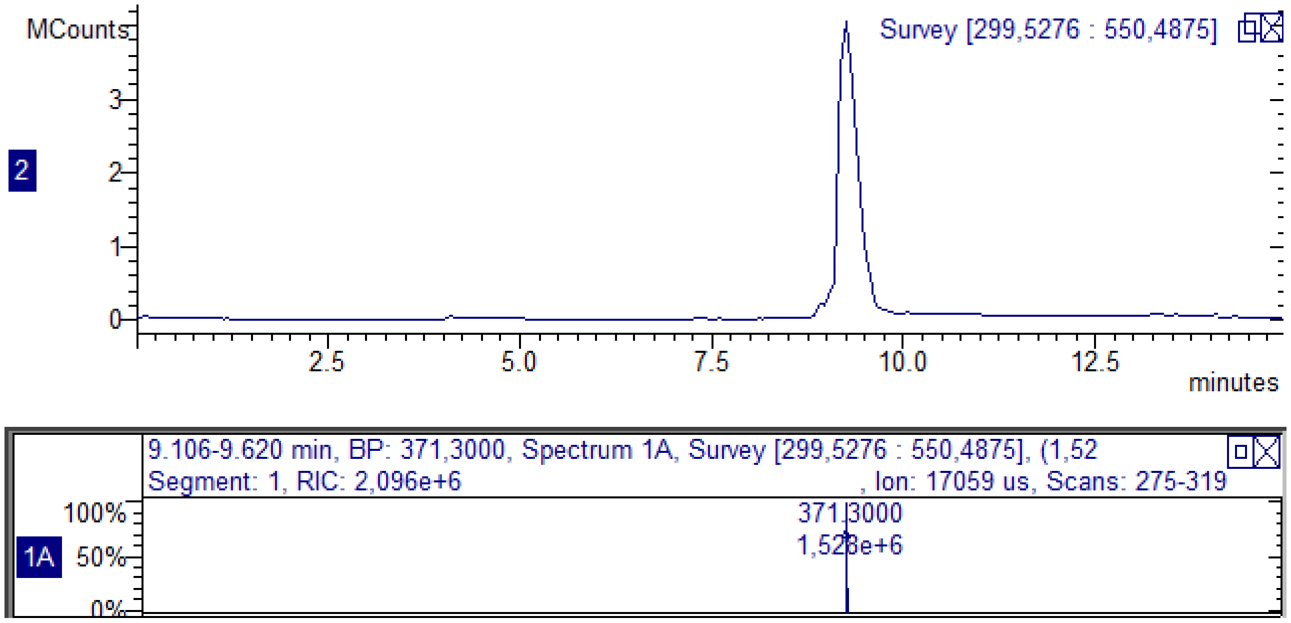

Figure S74: Chromatogram and MS spectrum of compound **6p**.

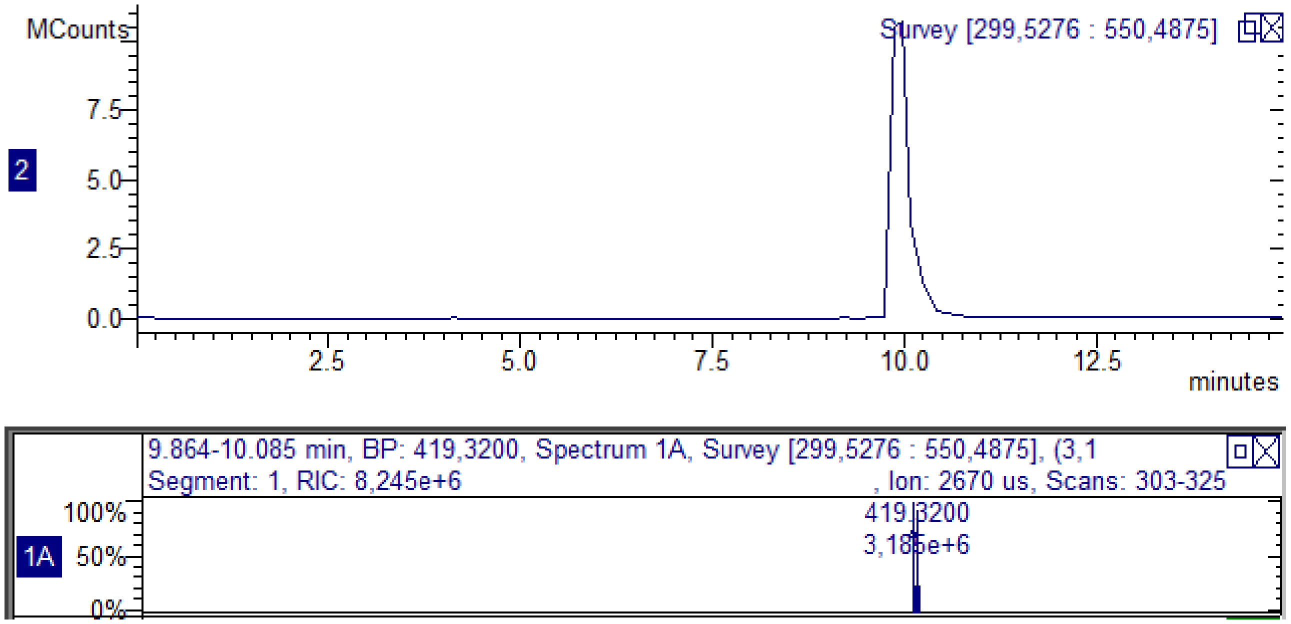

Figure S75: Chromatogram and MS spectrum of the compound **5q**.

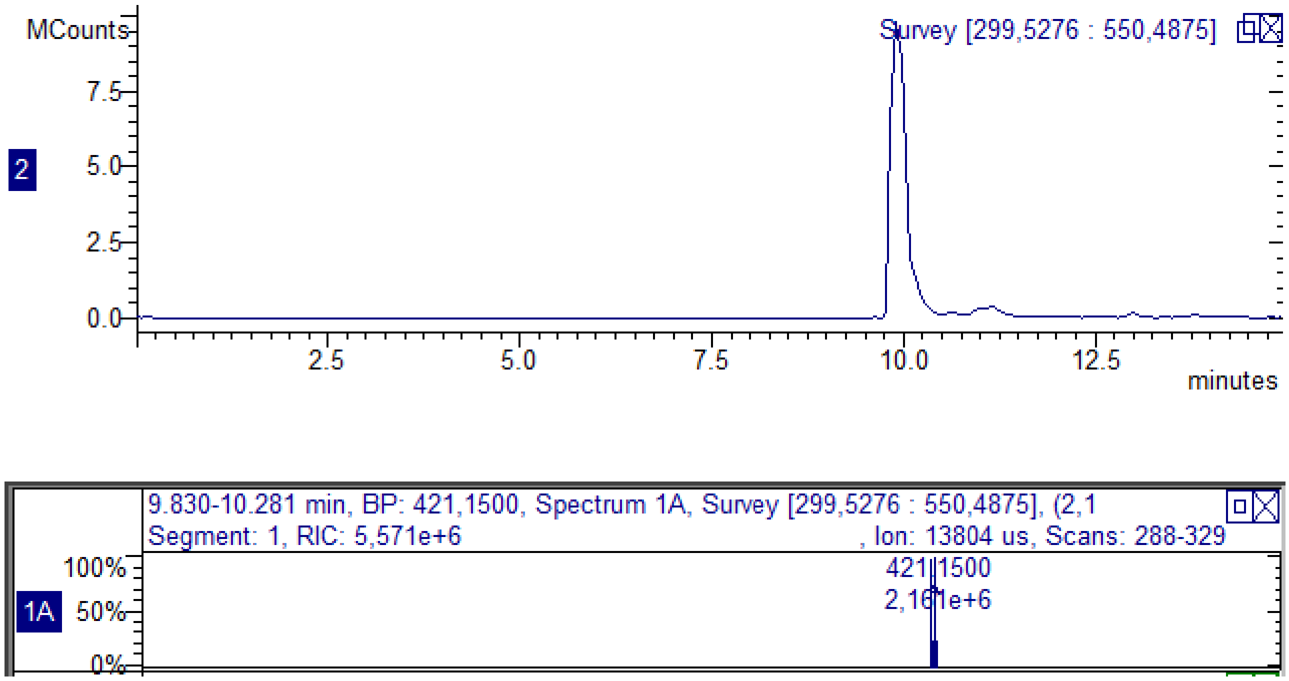

Figure S76: Chromatogram and MS spectrum of the compound **6q**.

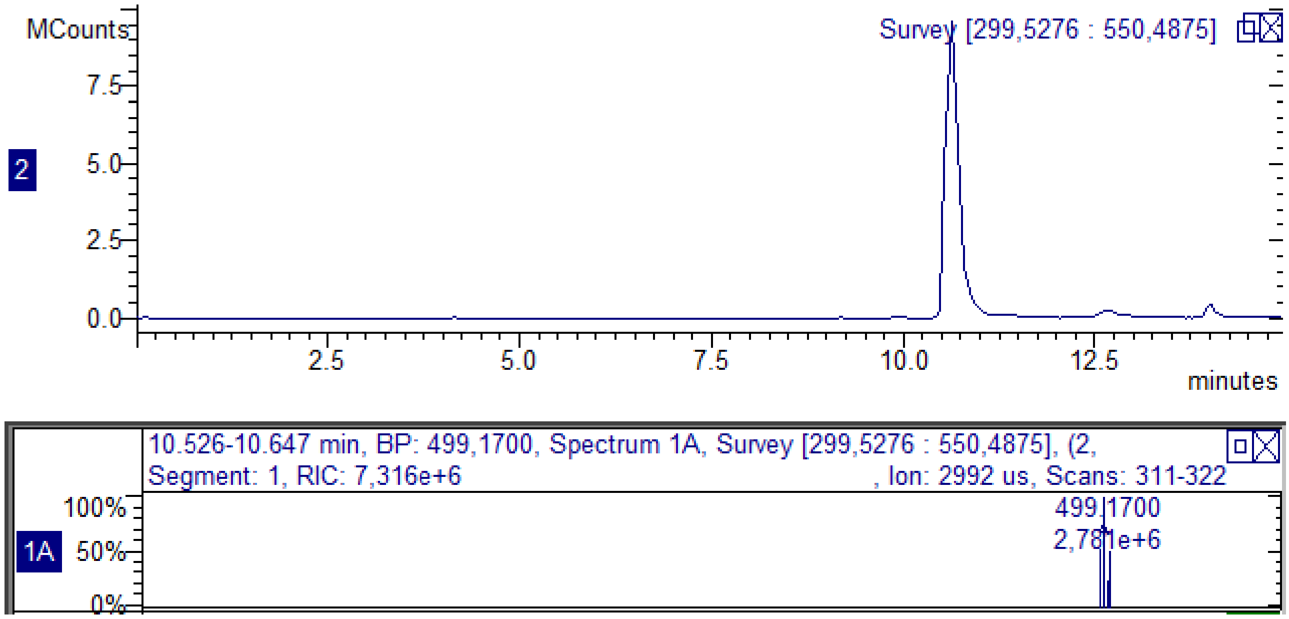

Figure S77: Chromatogram and MS spectrum of the compound **5r**.

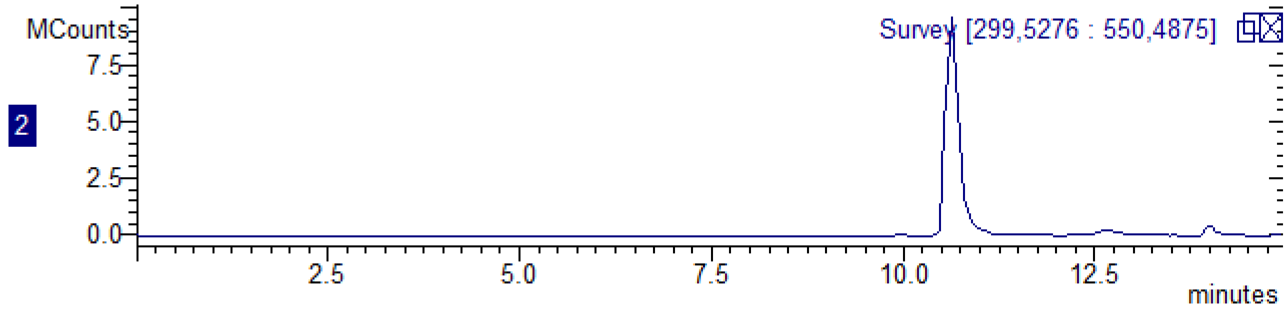

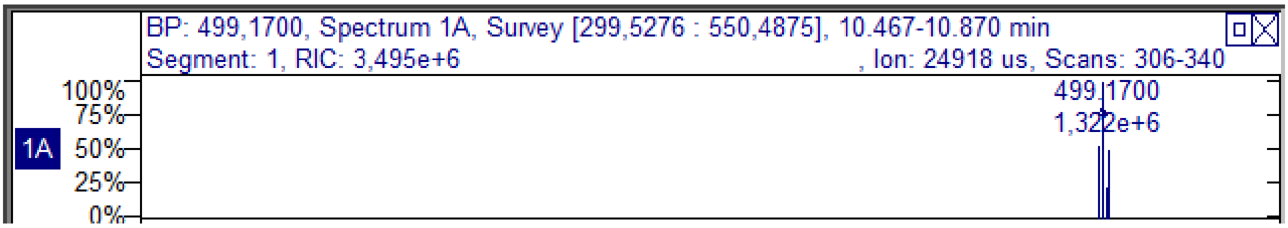

Figure S78: Chromatogram and MS spectrum of the compound **6r**.

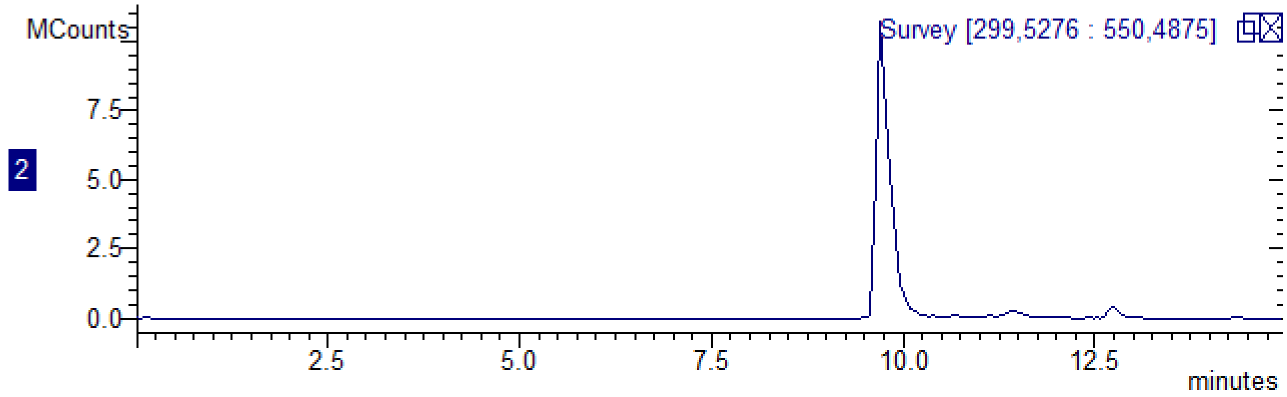

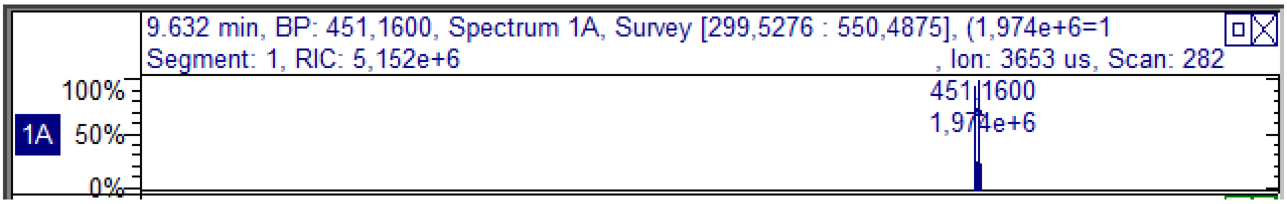

Figure S79: Chromatogram and MS spectrum of the compound **5s**.

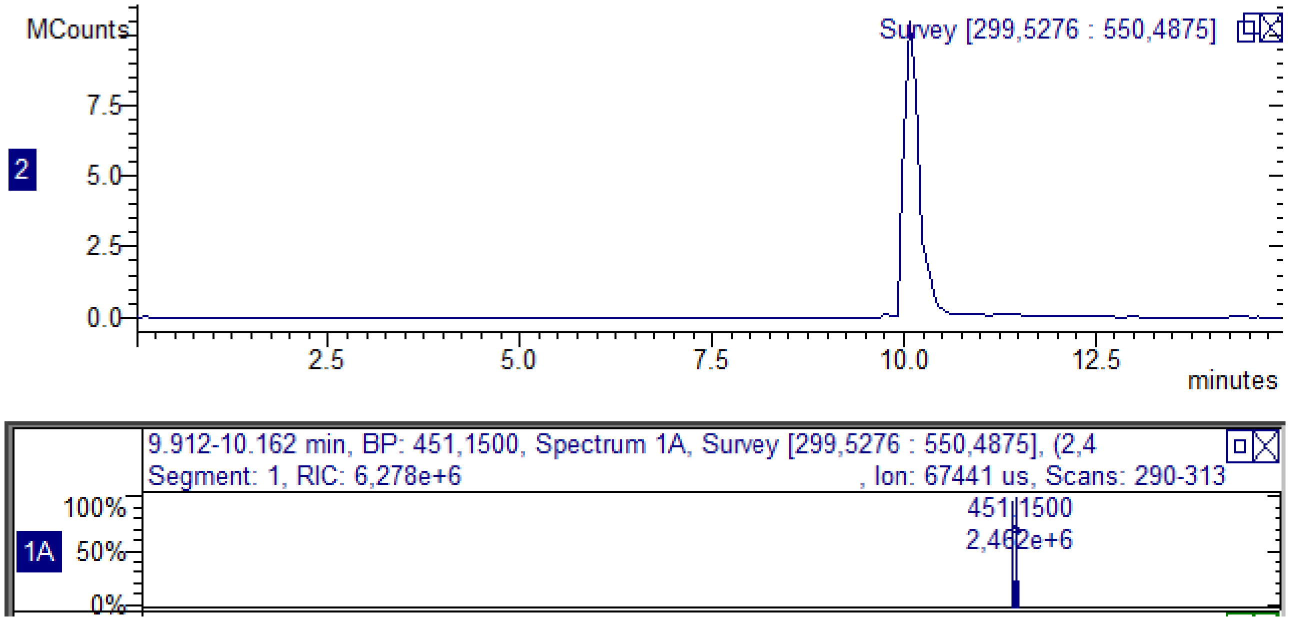

Figure S80: Chromatogram and MS spectrum of the compound **6s**.

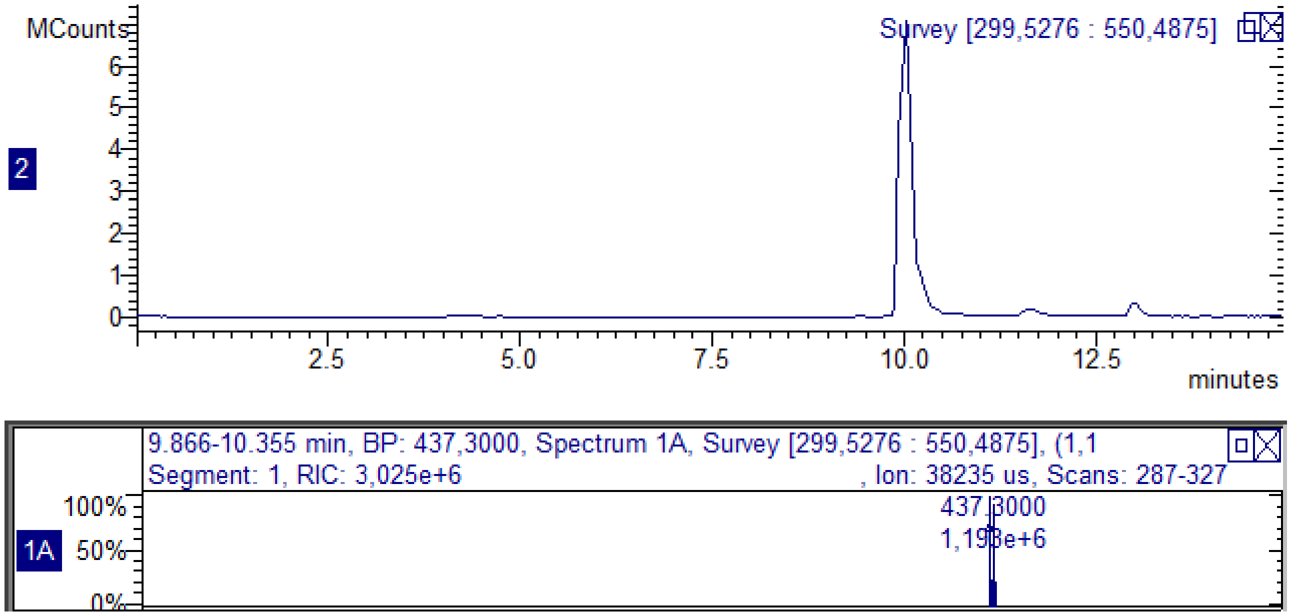

Figure S81: Chromatogram and MS spectrum of the compound **5t**.

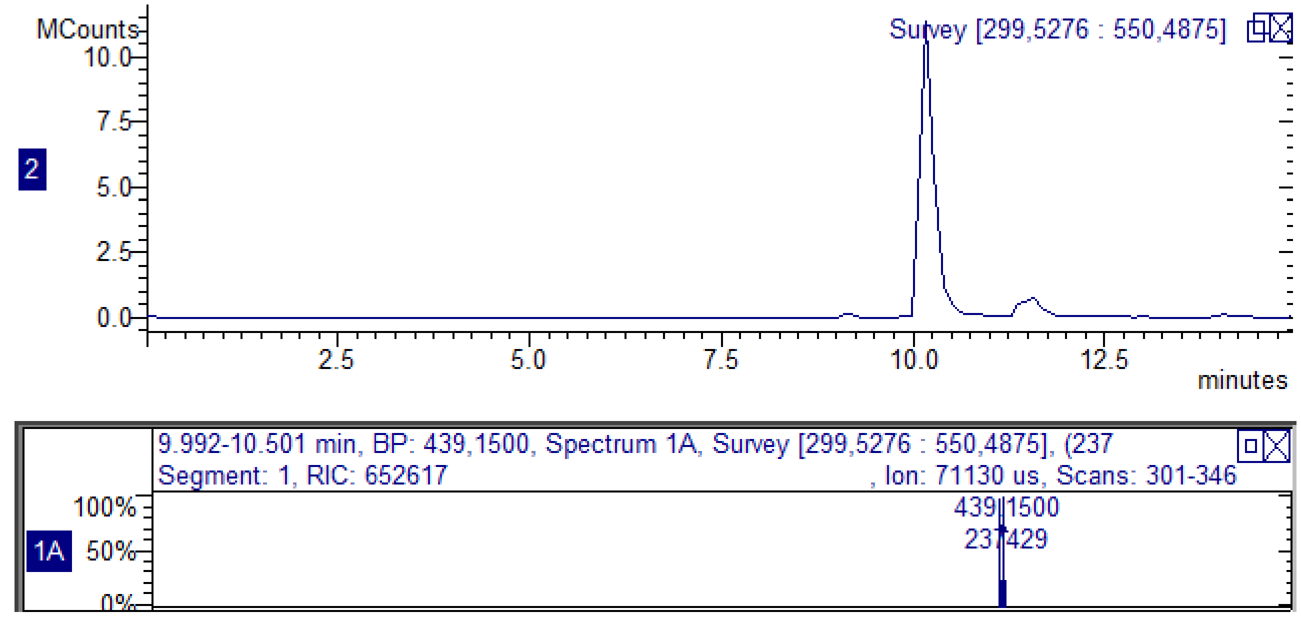

Figure S82: Chromatogram and MS spectrum of the compound **6t**.

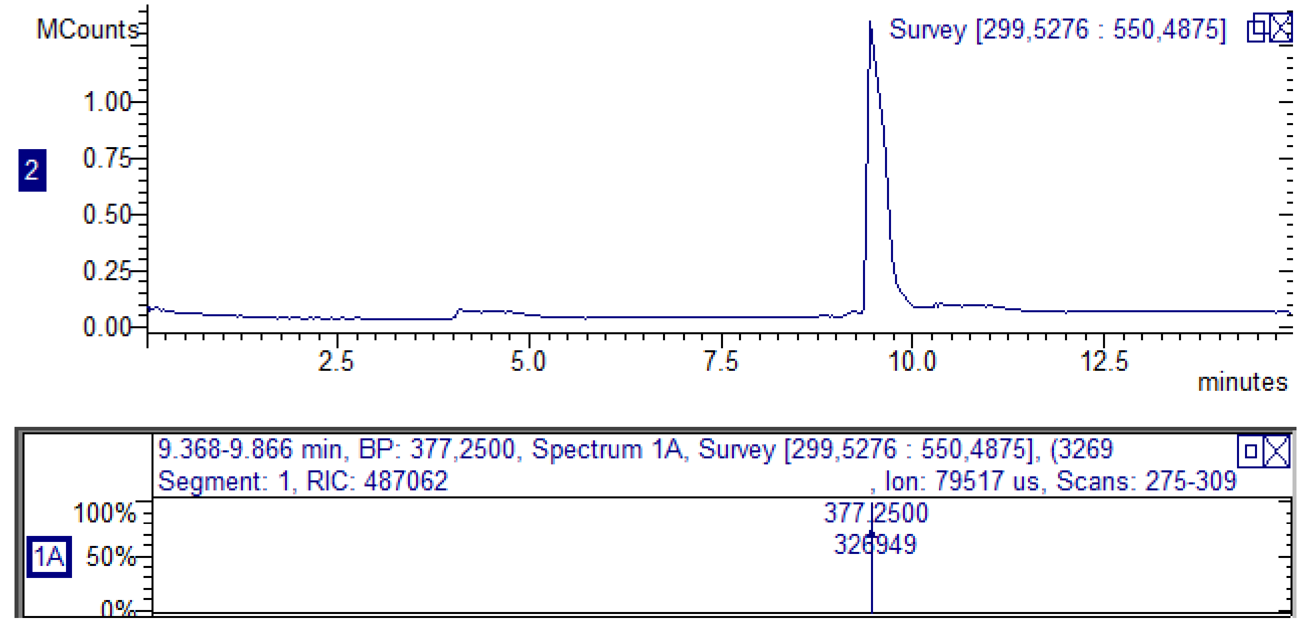

Figure S83: Chromatogram and MS spectrum of compound **5u**.

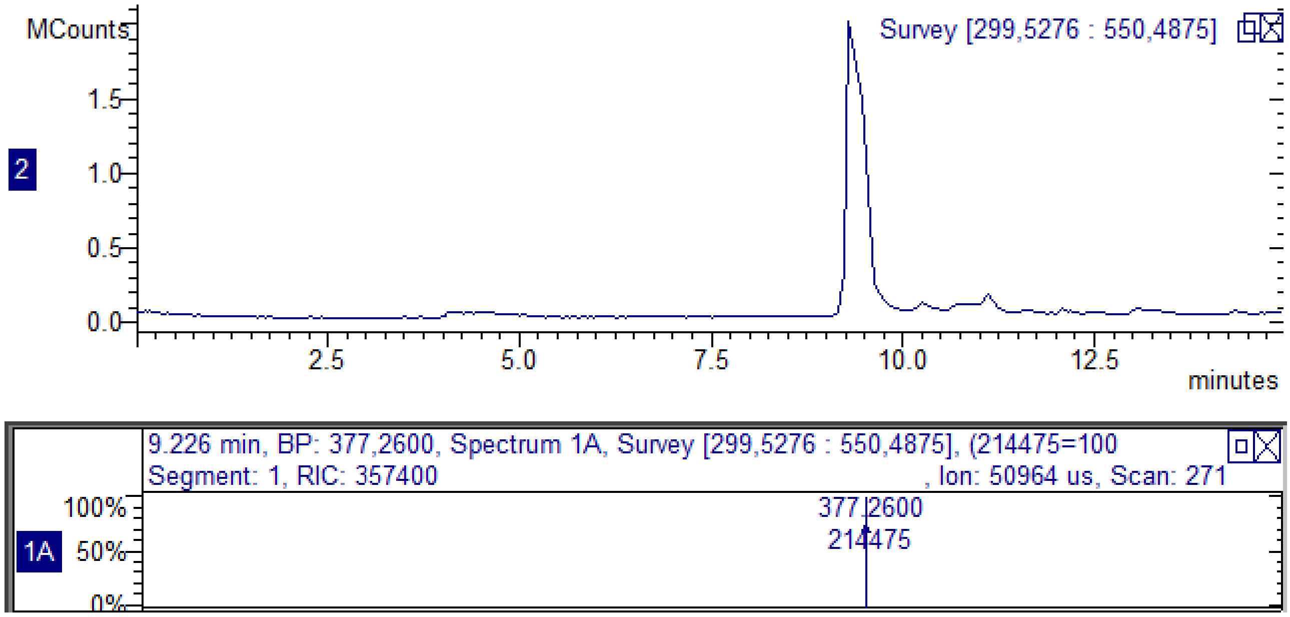

Figure S84: Chromatogram and MS spectrum of compound **6u**.

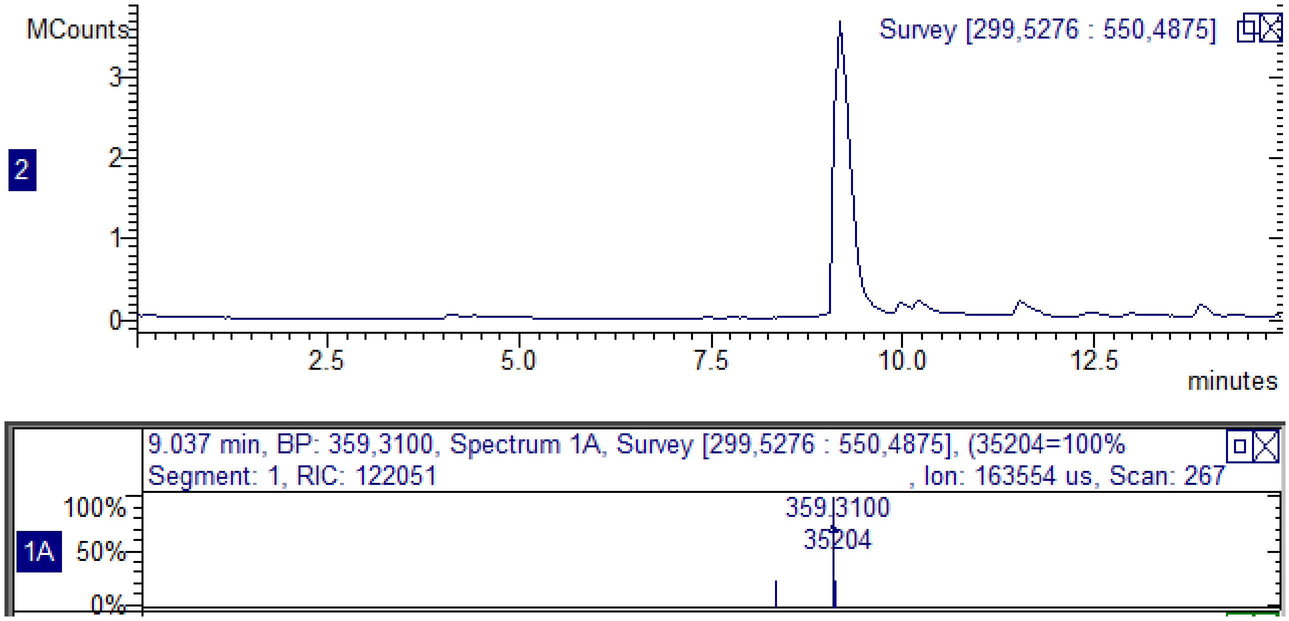

Figure S85: Chromatogram and MS spectrum of compound **5v**.

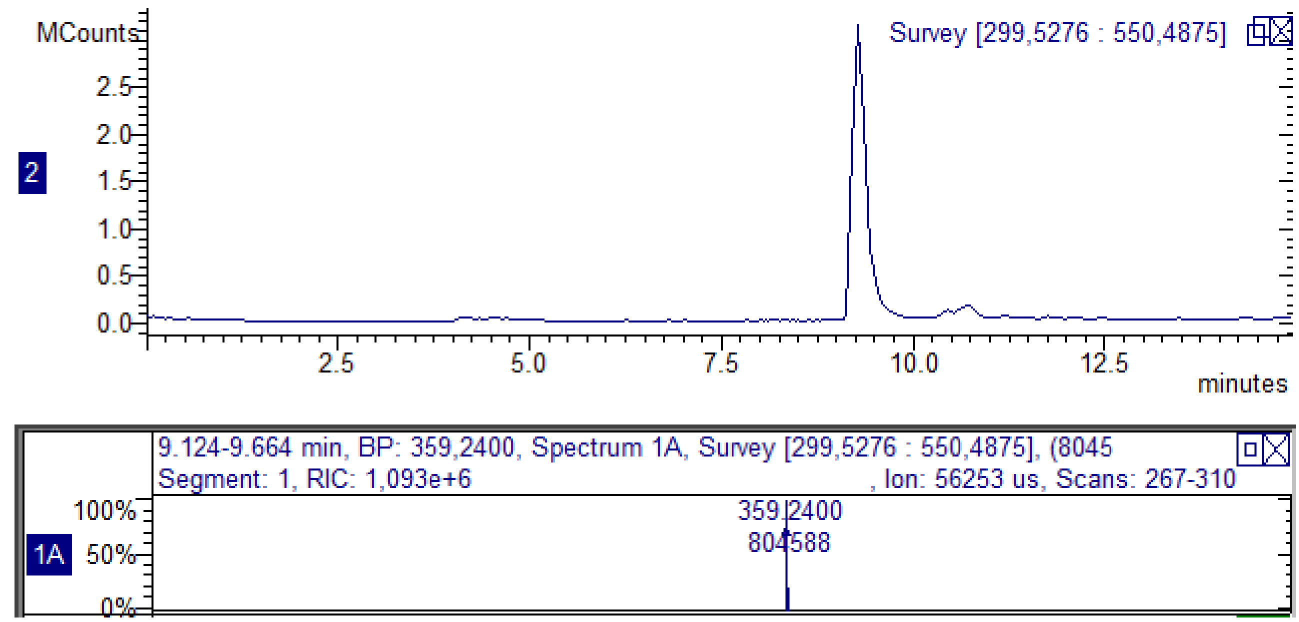

Figure S86: Chromatogram and MS spectrum of compound **6v**.

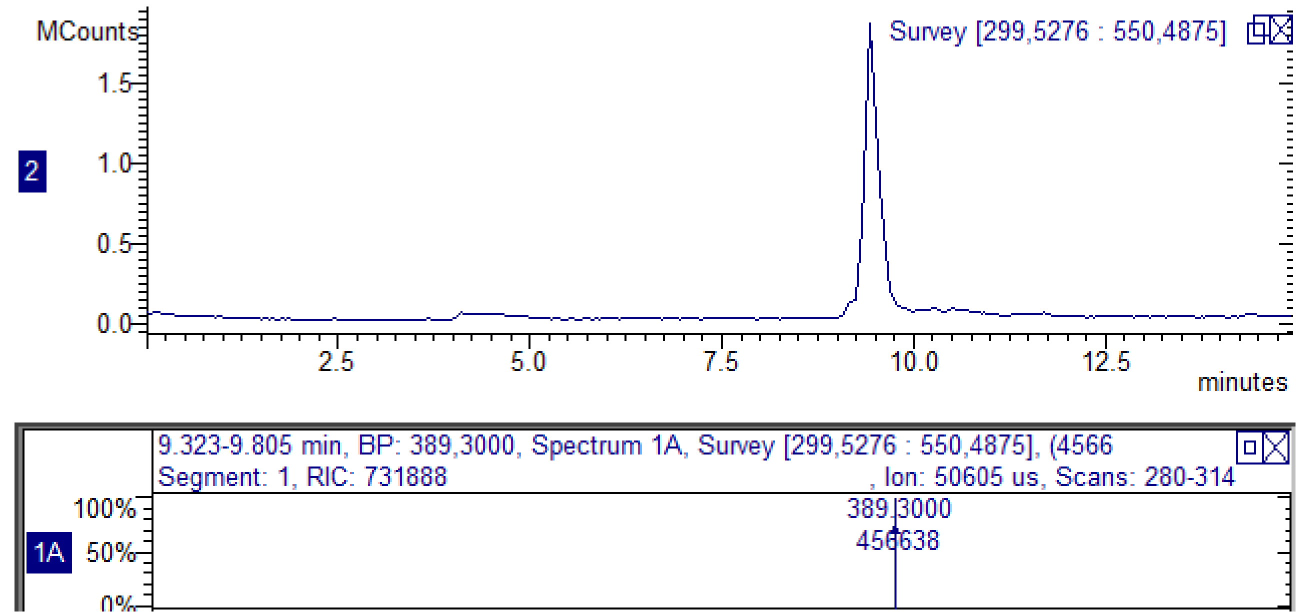

Figure S87: Chromatogram and MS spectrum of compound **5w.**

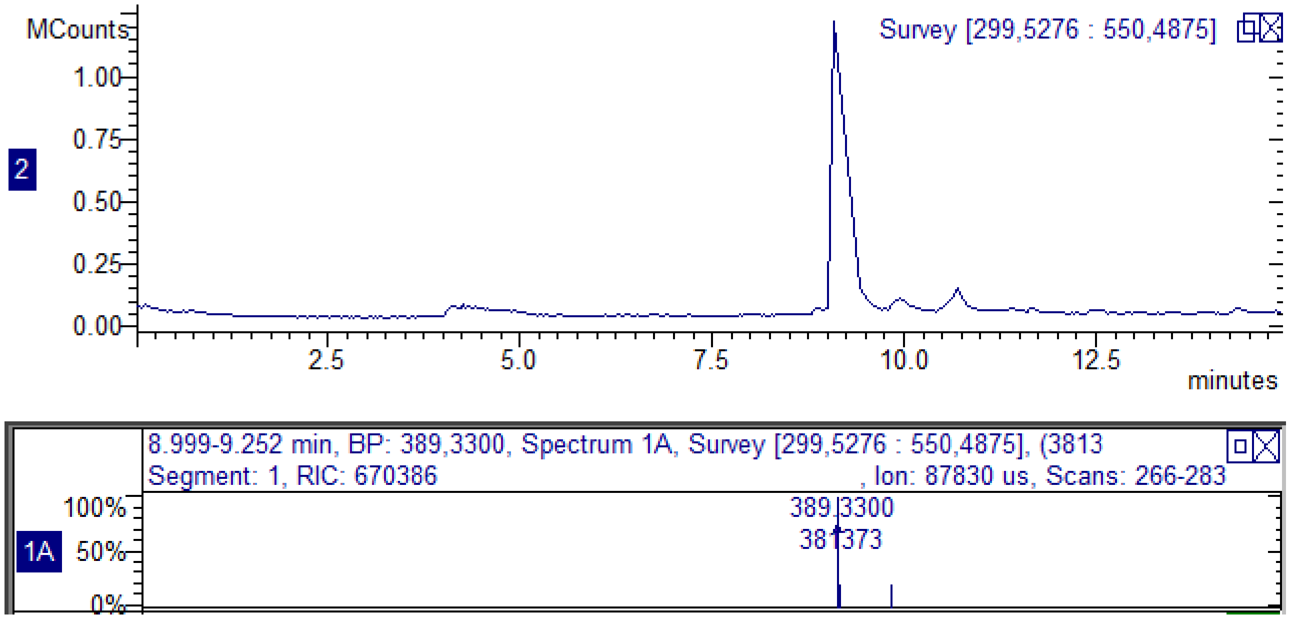

Figure S88: Chromatogram and MS spectrum of compound **6w**.

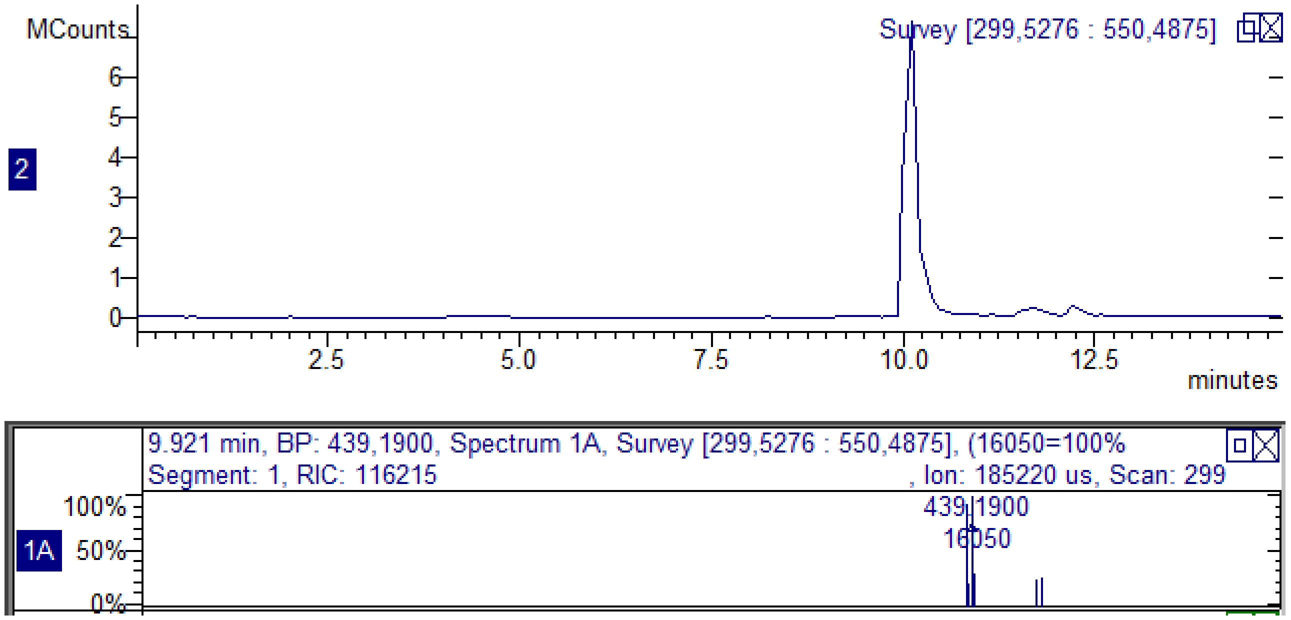

Figure S89: Chromatogram and MS spectrum of the compound **5x**.

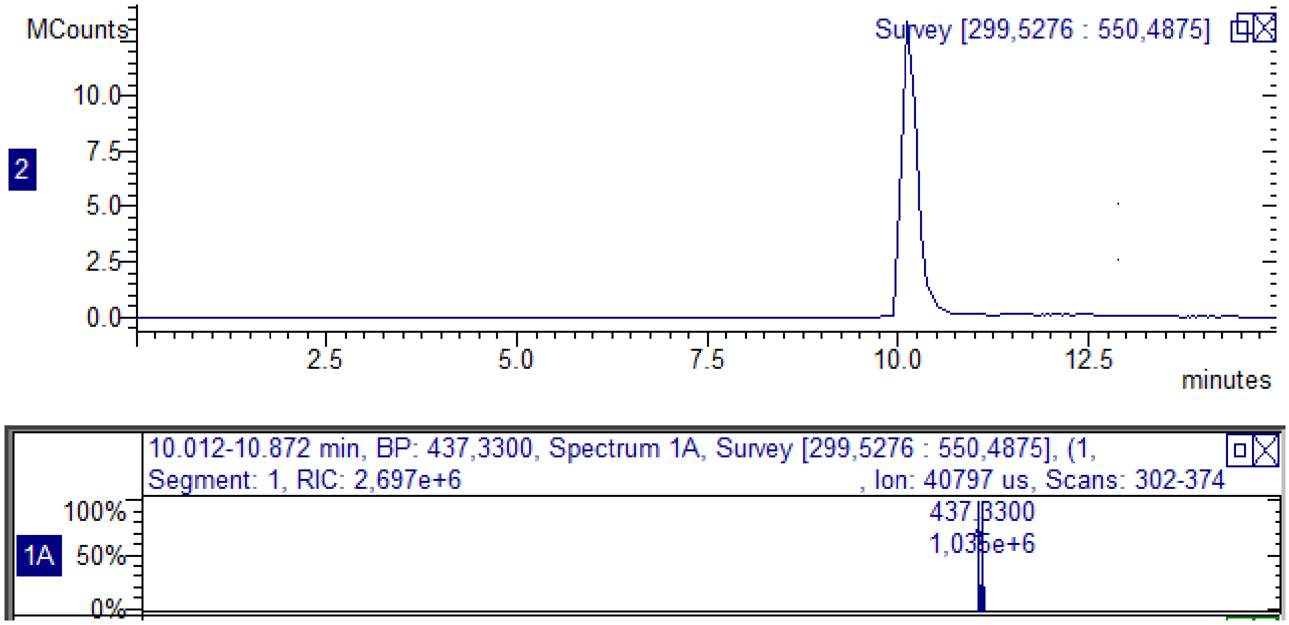

Figure S90: Chromatogram and MS spectrum of the compound **6x**.

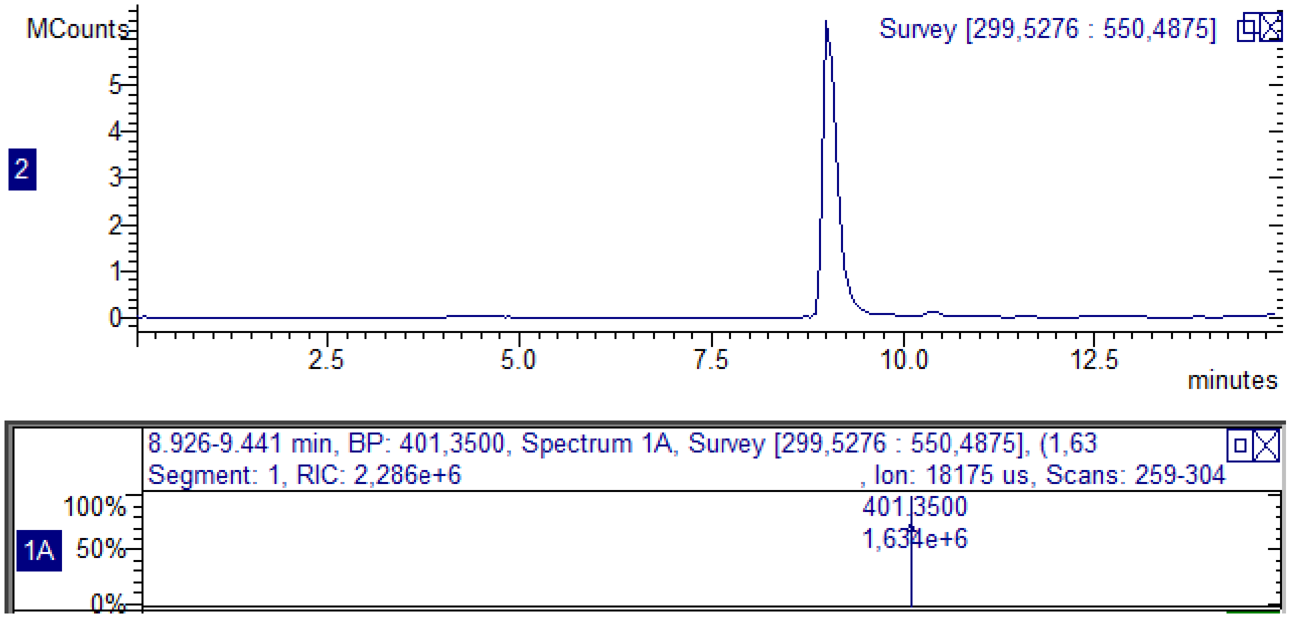

Figure S91: Chromatogram and MS spectrum of compound **5y**.

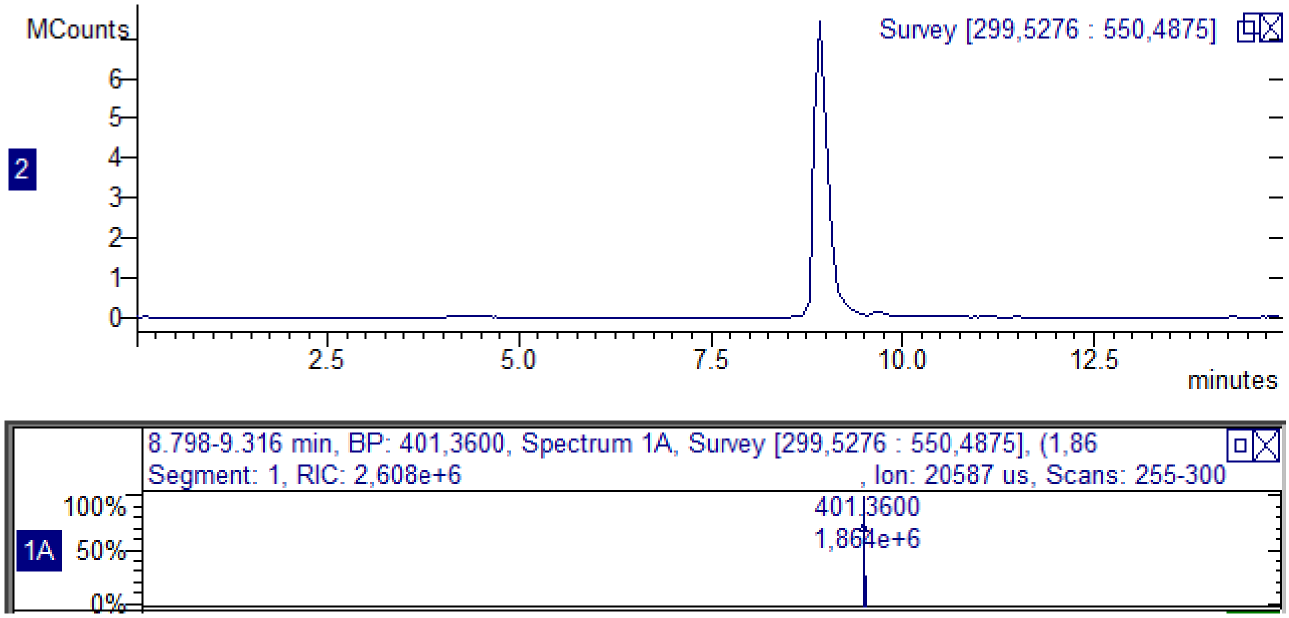

Figure S92: Chromatogram and MS spectrum of compound **6y**.

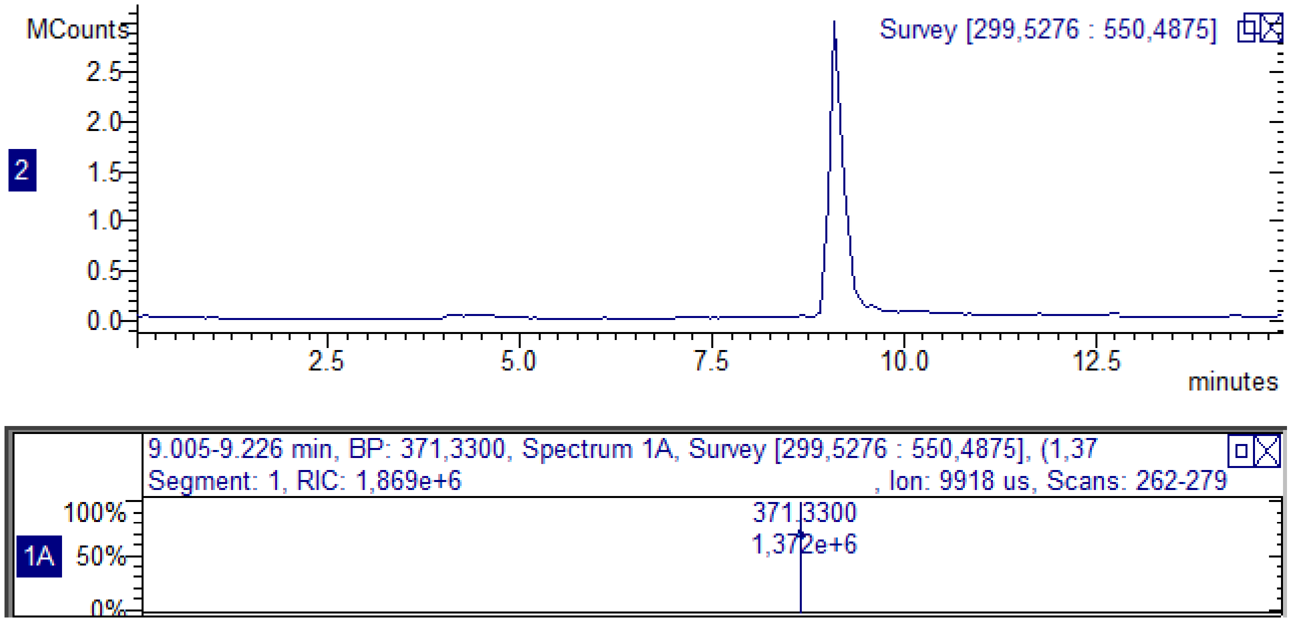

Figure S93: Chromatogram and MS spectrum of compound **5z**.

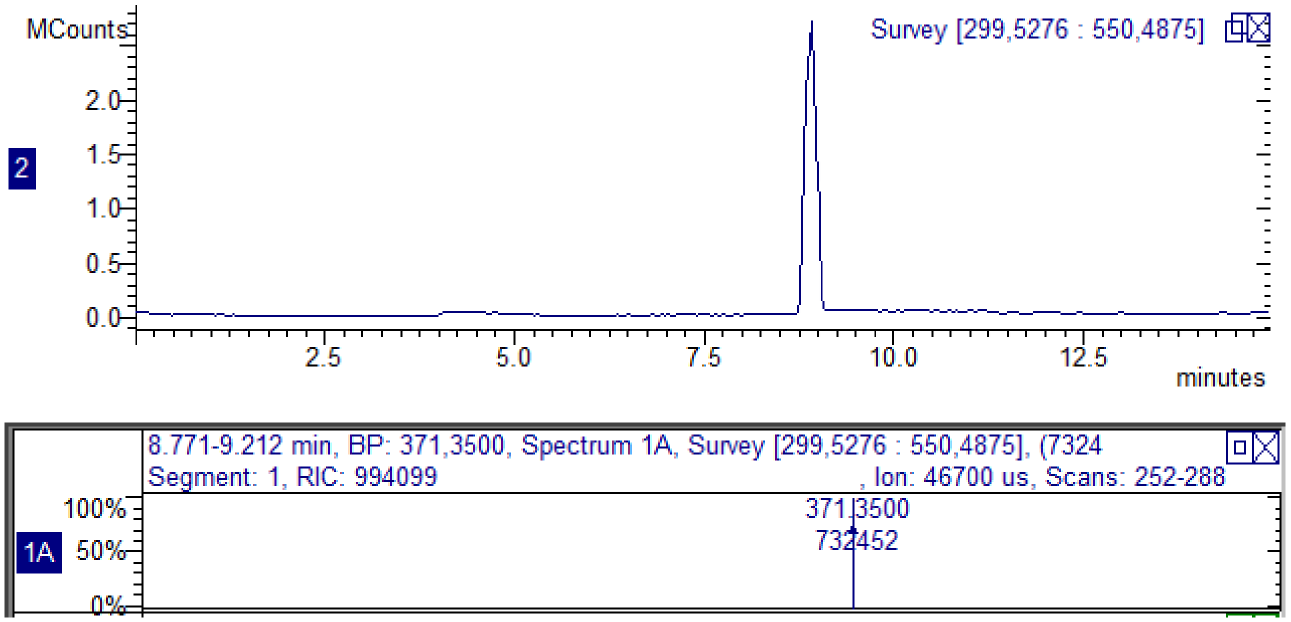

Figure S94: Chromatogram and MS spectrum of compound **6z**.

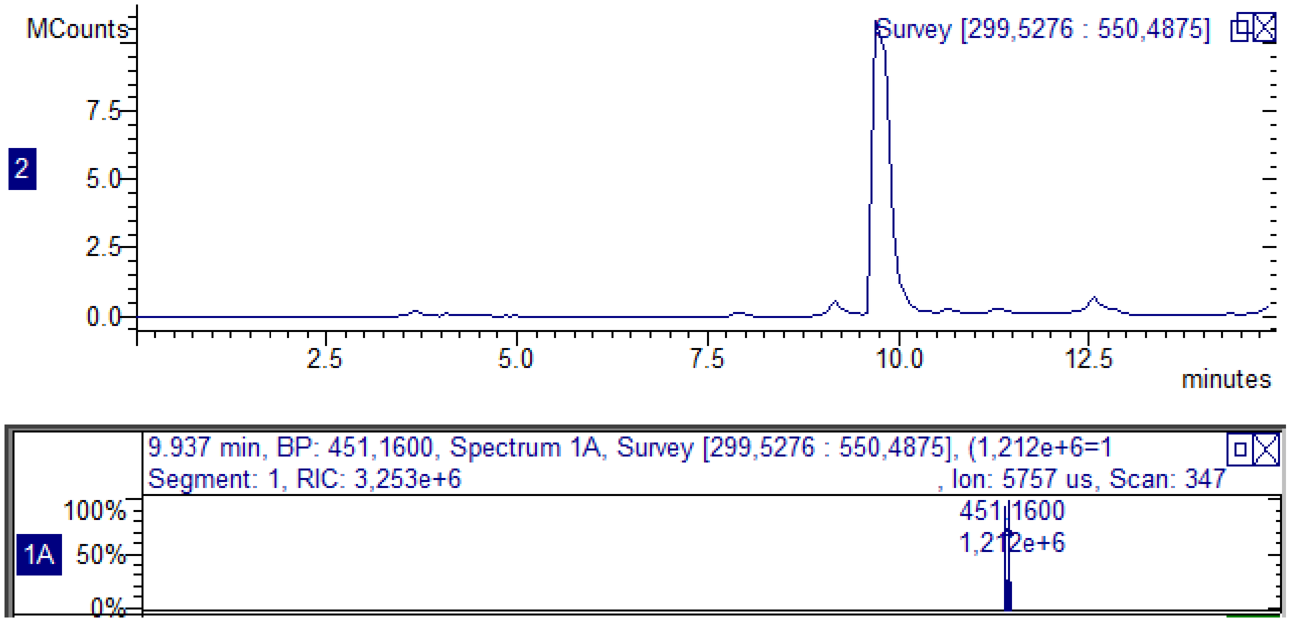

Figure S95: Chromatogram and MS spectrum of the compound **5aa**.

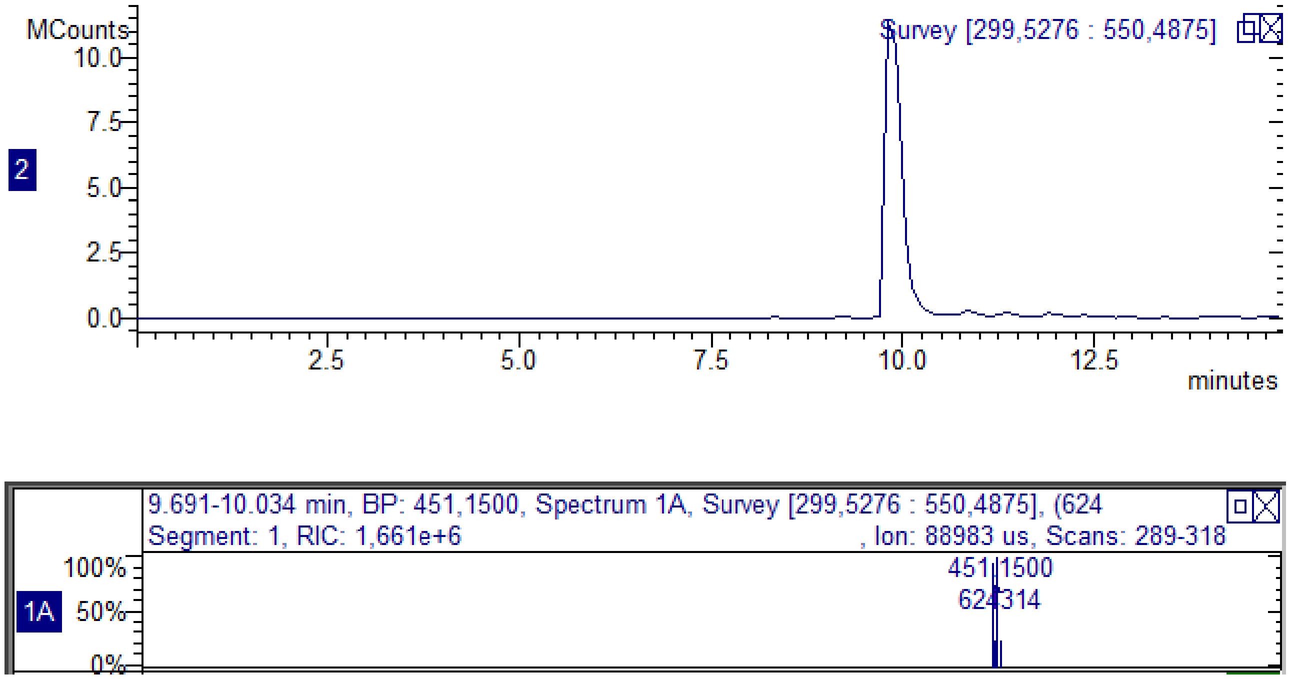

Figure S96: Chromatogram and MS spectrum of the compound **6aa**.

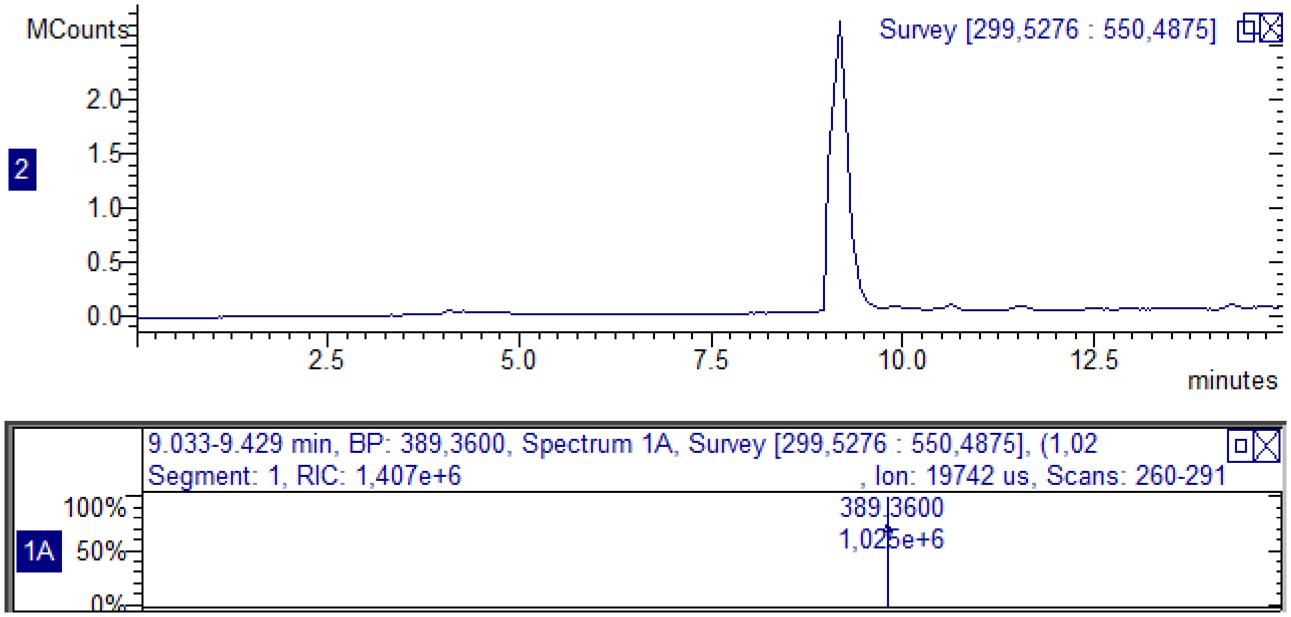

Figure S97: Chromatogram and MS spectrum of compound **5ab**.

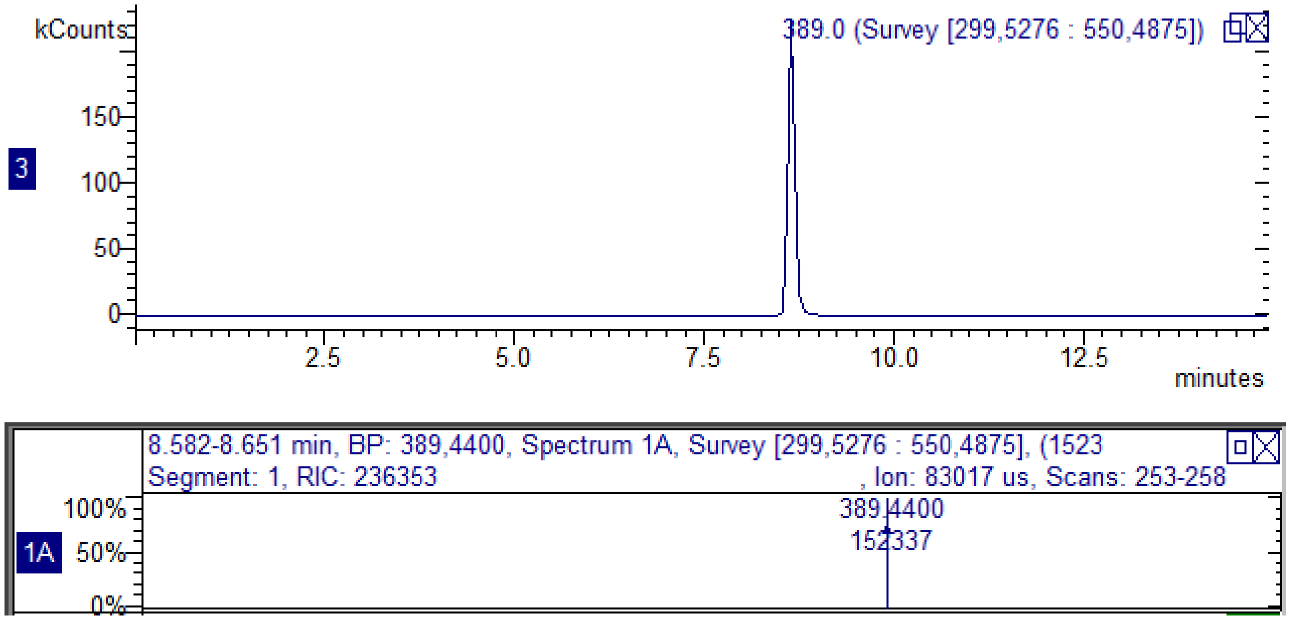

Figure S98: Chromatogram and MS spectrum of compound **6ab**.

Table S2. Antiproliferative activity of compounds **5a-ab** and **6h-ab** against PSN-1 and BxPC3 cancer cells evaluated in a 2D cytotoxicity assay.

Compound	IC_{50} (μM) ± S.D. PSN-1	IC_{50} (μM) ± S.D. BxPC3
5a	1.1±0.6	0.9±0.3
5b	0.5±0.2	0.4±0.1
5c	1.5±0.2	1.1±0.3
5d	0.30±0.04	0.4±0.1
5e	2.8±0.3	2.4±0.6
5f	0.7±0.3	0.5±0.2
5g	0.6±0.1	0.5±0.2
5h	2.7±0.6	2.4±0.5
5l	3.5±0.8	3.1±0.5
5j	0.4±0.1	0.10±0.04
5k	2.8±0.3	2.4±0.5
5l	2.8±0.4	2.3±0.7
5m	4.6±1.2	3.5±0.8
5n	1.2±0.2	2.2±0.7
5o	0.5±0.1	0.20±0.03
5p	1.1±0.1	0.9±0.2
5q	1.1±0.3	1.6±0.4
5r	0.7±0.1	0.50±0.04
5s	0.5±0.2	2.2±0.6
5t	0.4±0.1	1.4±0.2
5u	1.0±0.2	0.80±0.02
5v	0.20±0.04	0.20±0.03
5w	0.8±0.3	0.2±0.03
5x	0.3±0.1	0.2±0.04
5y	0.7±0.1	0.40±0.03
5z	1.4±0.4	1.1±0.3
5aa	1.3±0.3	1.2±0.6
5ab	3.2±0.7	2.8±0.9
Gemcltablne	0.10±0.04	0.020±0.003
DCA	>1000	>1000
DAP	10.2±1.6	16.5±0.9

Compound	IC_{50} (μM) ± S.D. PSN-1	IC_{50} (μM) ± S.D. BxPC3
6h	3.1±0.5	2.7±0.4
6i	1.2±0.2	1.0±0.4
6j	0.6±0.1	0.2±0.04
6k	1.7±0.5	1.0±0.2
6l	1.0±0.1	0.8±0.2
6m	6.5±1.1	4.9±0.7
6n	1.1±0.3	2.6±0.4
6o	0.40±0.04	0.30±0.03
6p	2.3±0.6	1.6±0.5
6q	1.2±0.2	2.5±0.3
6r	0.8±0.1	0.6±0.1
6s	1.1±0.2	1.8±0.2
6t	1.1±0.2	1.7±0.6
6u	5.3±0.4	4.7±0.6
6v	0.20±0.03	0.10±0.02
6w	0.8±0.2	0.30±0.04
6x	0.30±0.04	0.3±0.1
6y	0.60±0.04	0.3±0.1
6z	1.8±0.5	1.5±0.4
5ab	1.6±0.7	1.5±0.6
6ab	1.6±0.7	1.5±0.6
Gemcitabine	0.10±0.04	0.020±0.003
DCA	>1000	>1000
DAP	10.2±1.6	16.5±0.9

Cells (3 × 10^{3}·) were treated for 72 h with increasing concentrations of tested compounds. Cytotoxicity was assessed by MTT test. IC_{50} values were calculated by a four-parameter logistic model ($P < 0.05$). Gemcitabine, DCA and DAP were reported as reference compounds. Data represent mean values from at least 3 independent experiments. S.D.= standard deviation.

Table S3. Antiproliferative activity of compounds **5a-ab, 6h-ab** against PSN-1 and BxPC3 cancer cells evaluated in a 3D cytotoxicity assay.

Compound	IC_{50} (μM) ± S.D. PSN-1	IC_{50} (μM) ± S.D BxPC3
5a	58.2±4.1	99.4±4.2
5b	32.9±10.2	38.2±3.1
5c	43.8±5.1	83.1±4.8
5d	25.9±1.3	33.4±3.1
5e	32.9±0.5	134.5±6.8
5f	32.9±1.7	33.5±4.2
5g	5.8±0.8	19.5±2.1
5h	44.5±3.6	78.4±4.2
5i	4.9±0.8	34.1±1.5
5j	10.4±1.6	16.7±2.0
5k	15.9±2.3	67.9±3.7
5l	8.7±0.5	52.1±3.3
5m	29.8±2.9	96.4±5.2
5n	21.7±3.4	17.6±2.3
5o	10.3±1.3	20.6±1.2
5p	15.8±1.4	76.4±4.2
5q	8.4±0.5	8.6±0.3
5r	36.6±1.5	35.8±2.3
5s	14.7±2.1	56.5±4.7
5t	25.7±1.7	31.1±2.8
5u	9.2±0.7	16.6±0.02
5v	28.1±3.4	56.8±3.9
5w	15.3±2.8	12.2±1.3
5x	26.3±4.3	26.2±2.4
5y	40.1±3.4	35.3±2.3
5z	19.2±3.5	19.2±3.5
5aa	54.4±2.7	49.2±2.6
5ab	131.5±7.6	69.8±3.6
Gemcitabine	159.5±5.6	102.6±6.3
DCA	>1000	>1000
DAP	78.2±6.2	87.4±8.9

Compound	IC_{50} (μM) ± S.D. PSN-1	IC_{50} (μM) ± S.D. BxPC3
6h	24.3±1.4	33.4±3.6
6i	20.9±4.2	29.3±2.8
6j	18.1±2.3	41.2±3.6
6k	9.4±0.2	22.3±2.6
6l	8.0±0.1	8.8±0.4
6m	28.2±2.4	103.1±3.6
6n	12.9±2.1	19.1±2.2
6o	18.9±3.0	27.4±3.3
6p	28.7±2.1	33.6±7.5
6q	14.2±1.1	20.7±2.3
6r	23.5±2.2	29.4±1.6
6s	22.3±2.3	29.2±1.4
6t	21.1±2.5	31.1±2.1
6u	15.2±3.1	34.5±2.6
6v	45.2±4.8	68.1±5.1
6w	9.2±0.5	58.1±2.3
6x	30.6±3.6	28.7±2.1
6y	49.0±2.7	37.7±4.1
6z	19.5±2.6	19.3±3.6
6aa	25.3±1.8	28.3±2.5
6ab	118.2±8.2	61.9±4.2
Gemcitabine	159.5±5.6	102.6±6.3
DCA	>1000	>1000
DAP	78.2±6.2	87.4±8.9

Spheroids ($2.5x10^3$ cells/well) were treated for 72 h with increasing concentrations of tested compounds. The growth-inhibitory effect was evaluated by means of the acid phosphatase (APH) test. IC_{50} values were calculated from the dose–survival curves using a four-parameter logistic model ($p < 0.05$). SD = standard deviation.

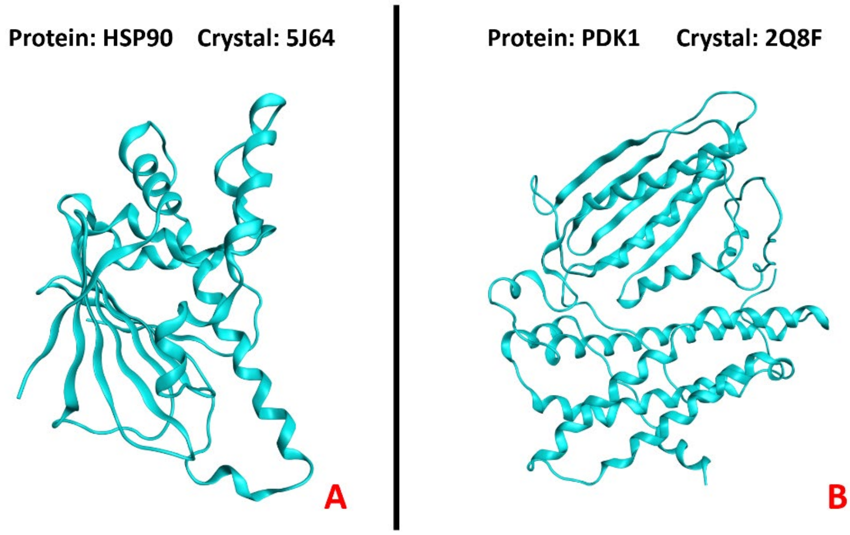

Figure S99. Panel A) Depiction of the HSP90 crystallographic structure with PDB code 5J64, which was the one considered in this study. **Panel B)** Representation of the PDK1 crystallographic structure with PDB code 2Q8F, which was the one considered in this study. The images were created and rendered with MOE.

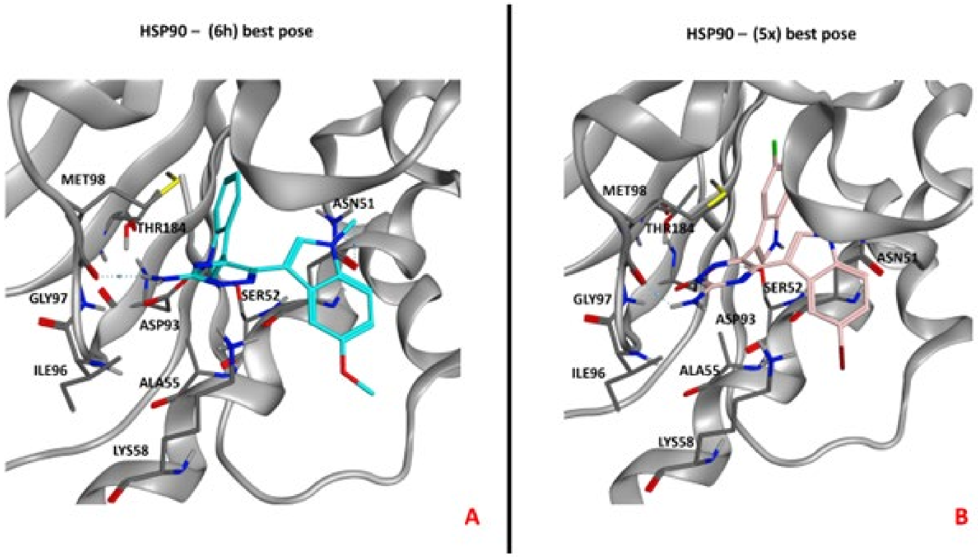

Figure S100. Representation of the binding conformation of the two compounds that we chose as a reference for our computational evaluation in the HSP90 orthosteric binding site. **Panel A)** depiction of the selected pose produced by PLANTS for the compound **6h** (colored in cyan), which passes through the steric, the electrostatic, and the 3D-pharmacophore filtering. **Panel B)** illustration of the selected pose produced by PLANTS for the ligand **5x** (colored in pink) which passes through the steric, the electrostatic, and the 3D-pharmacophore filtering. The images were created and rendered with MOE.

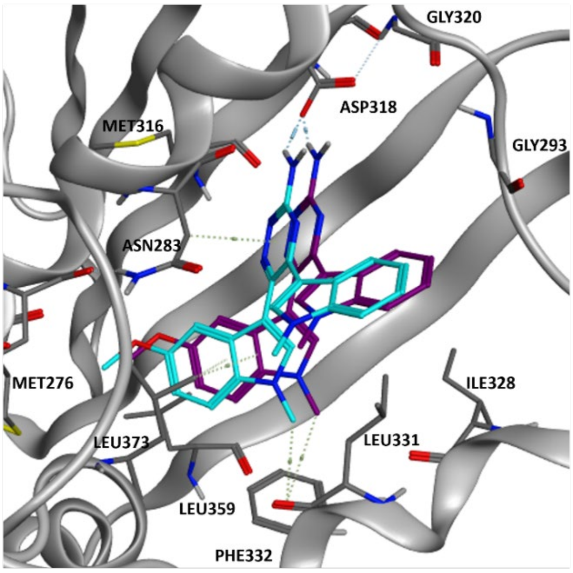

Figure S101. Representation of the superimposition of the best pose obtained for compound **6h** (colored in cyan) and the originally placed conformation (colored in purple), which was used to create the ligand-based homology model. The very low value of RMSD (0.87 Å) gives credit to this binding hypothesis.

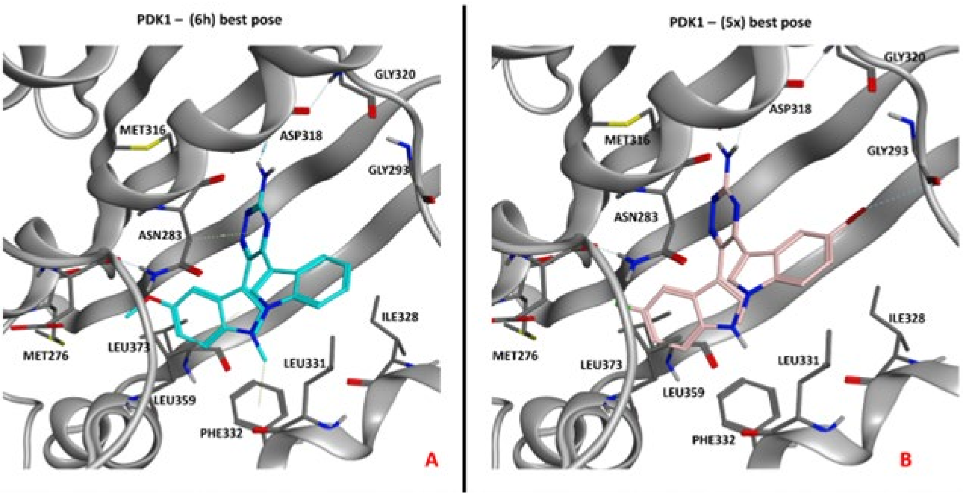

Figure S102. Depiction of the best poses of the two compounds chosen as the reference for our study in the PDK1 ATP-binding site. **Panel A**) representation of the chosen pose produced by PLANTS for the potent ligand **6h**, (colored in cyan), which passes both the steric and electrostatic filtering process. **Panel B**) picture showing the chosen pose produced by PLANTS for the ligand **5x**, (colored in pink) which passes both the steric and electrostatic filtering process. The images were created and rendered with MOE.
